# Photochemical Hetero-cycloadditions

**DOI:** 10.1021/acs.chemrev.6c00681

**Published:** 2026-10-09

**Authors:** Cristian Rosso, Vasco Corti, Giulio Goti, Sara Cuadros, Enrico Marcantonio, Luca Dell’Amico

**Affiliations:** § Department of Chemical Sciences, 9308 University of Padova, Via Marzolo 1, 35131, Padova, Italy; ¶ Instituto de Síntesis Orgánica (ISO) and Departamento de Química Orgánica, 16718 Universidad de Alicante, Apdo. 99, 03080, Alicante, Spain

## Abstract

Hetero-cycloaddition reactions are powerful, atom-economical strategies for the rapid construction of heterocyclic frameworks from readily available building blocks. Over the past two decades, advances in photochemistry, photocatalysis, and modern excitation strategies have driven remarkable developments in the field. By accessing electronically excited states, light enables cycloaddition pathways inaccessible under classical thermal conditions, providing access to reactive radical, ionic, or excited-state intermediates under mild conditions. Technological advances in light sources and photocatalyst design have improved reaction efficiency, site-, regio-, and stereoselectivity, while considerably expanding the accessible chemical space. Beyond established hetero-[2 + 2], hetero-(3 + 2), and hetero-[4 + 2] processes, new reaction classes, including π–σ hetero-cycloadditions exploiting strained small-ring systems, have emerged as powerful approaches to structurally complex saturated scaffolds of increasing relevance in medicinal chemistry. In this Review, we critically examine the evolution of photochemical hetero-cycloadditions from 2000 to 2026, discussing fundamental concepts and terminology, cycloaddition classes, heterocyclic targets, and activation mechanisms. We highlight the conceptual advances that have shaped modern photochemical hetero-cycloaddition chemistry and conclude by discussing current limitations and future directions for the field.

## Graphical abstract

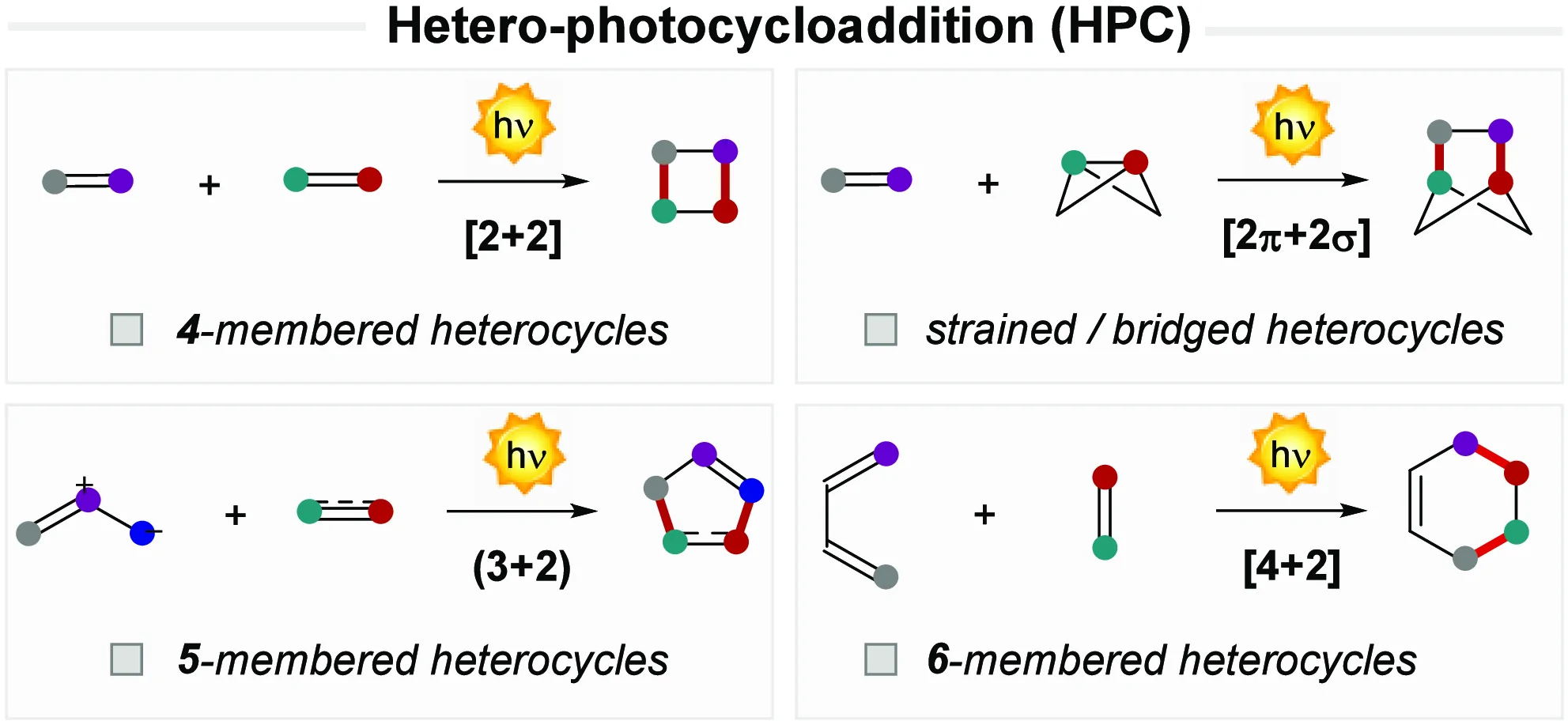

## 1 Introduction

Cycloaddition reactions are a powerful class of transformations in organic synthesis, enabling the rapid construction of cyclic molecular architectures through the formation of multiple covalent bonds. Cycloadditions are classically defined as reactions involving two or more unsaturated moieties leading to the formation of cyclic scaffolds. This event is associated with a net reduction of bond multiplicity.^1^ Due to their intrinsic atom economy and the ability to forge simultaneously two stable bonds in a single synthetic operation, cycloadditions are powerful synthetic tools in organic chemistry.^2–7^ Within this broad family of transformations, hetero-cycloadditions occupy a prominent position (Figure 1a, left).^8–11^ By incorporating one or more heteroatoms into the newly formed cyclic framework, these reactions provide direct access to oxygen-, nitrogen-, or sulfur-containing rings, among the others. These structural motifs are ubiquitous throughout chemistry, biology and material sciences, constituting the privileged scaffolds in a number of natural products, agrochemicals, materials, and most marketed pharmaceuticals.^12–16^

**Figure 1:**
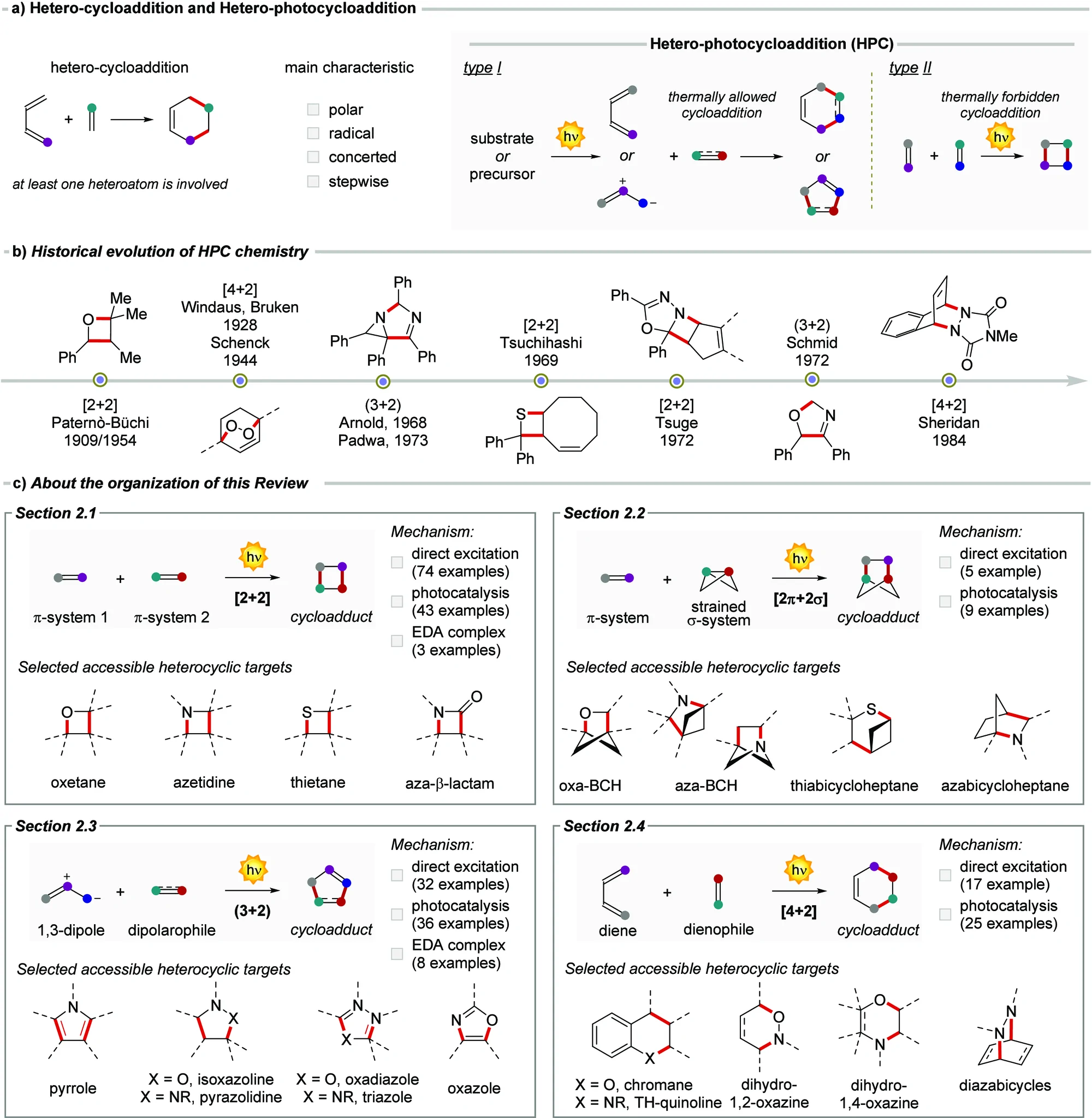
(a) Overview on cycloaddition reactions. (b) Historical evolution of heterophotocycloaddition (HPC) chemistry. (c) Classes of HPCs discussed in this review, selected accessible heterocyclic targets and most common photochemical activation strategies.

In the past few decades, photochemistry and photocatalysis have tremendously boosted the development of hetero-cycloaddition methods. The photoexcitation of molecules alters orbital distributions and the related reaction pathways, often enabling bond formations that are otherwise inaccessible under the thermal domain. Here, photochemical activation provides access to a mechanistic landscape involving reactive species with unique synthetic potential.^17,18^ The combination of photochemistry with hetero-cycloaddition processes leads to a uniquely versatile class of transformations referred as hetero-photocycloadditions (HPC). In contrast to thermal hetero-cycloadditions, which often proceed through concerted mechanisms governed by orbital symmetry constraints,^19^ HPCs exploit the distinctive reactivity of radicals and/or other photochemically generated highly reactive intermediates. Importantly, the role of light in HPC chemistry is not univalent. In some cases, light serves primarily to generate highly reactive intermediates, which subsequently engage in “thermally allowed” cycloaddition events. In others, photoexcitation directly enables cycloaddition pathways that are thermally forbidden, as exemplified by classical [2 + 2] cycloadditions (Figure 1a, right). Therefore, HPCs involve a broad array of different mechanisms, in which the cycloaddition step may proceed in a concerted or stepwise fashion and engage in either polar or radical bond-forming events, depending on the activation mode and the reactive intermediates generated.

Although a variety of diverse HPCs have been developed, [2 + 2], [2π+2σ], (3 + 2), and [4 + 2] are the most prominent and widely investigated reaction classes. Depending on the nature of the reacting partners and the specific activation mode(s), these transformations provide access to four-, five-, and six-membered heterocycles, frequently with high levels of regio-, and diastereocontrol.

It is worth mentioning that two nomenclatures are commonly used to describe cycloaddition reactions: an electronic one, based on the number of interacting electrons and denoted by square brackets (i.e., [*i* + *j*]), and a topological one, based on the number of atoms involved in the cycloaddition and denoted by round brackets (i.e., (*m* + *n*)).^20,21^

The first report of HPC can be traced back to the earliest developments of organic photochemistry (Figure 1b). In 1909, the Italian chemist Emanuele Paternò reported a photochemical [2 + 2] cycloaddition between carbonyl compounds and alkenes,^22^ establishing the foundations of the so-called Paternò-Büchi reaction.^23^ The seminal investigations of Büchi supported these initial observations into a synthetically useful methodology, establishing mechanistic foundation of the process. Thus, this carbonyl-olefin [2 + 2] HPC reaction is the first broadly applicable reaction for the rapid assembly of oxetanes. Following these pioneering reports, the reactivity was expanded significantly to incorporate alternative heteroatoms.^24^ This led to the development of aza-Paterno-Büchi and thia-Paternò-Büchi reactions for the synthesis of azetidines and thietanes, respectively.^25,26^

During the last part of the twentieth century, heterophotocycloaddition chemistry grew significantly through the emergence of [4 + 2] and (3 + 2) HPCs. The earliest precedents of hetero-[4 + 2] cycloadditions date back to the pioneering works of Windaus and Brunken in 1928,^27^ and later Schenck in the 1940s.^28^ Their studies established the sensitized photo-oxygenation of conjugated dienes, demonstrating that photogenerated singlet oxygen (^1^O_2_*) could act as an efficient heterodienophile to provide endoperoxides. Later developments broadened the scope of hetero-[4 + 2] photocycloadditions by exploiting arenophiles^29^ and highly reactive *ortho*-quinone methide and *ortho*-quinodimethane intermediates generated via direct photoexcitation.^30,31^ The historical development of (3 + 2) HPCs can be traced back to 1968, when Arnold and co-workers reported the unexpected formation of an azabicyclopentane derivative via the photolysis of α-azidostyrene.^32^ The conceptualization and generalization of this reactivity was established in the early 1970s. Through independent studies, Padwa^33^ and Schmid^34^ demonstrated that the UV irradiation of 2*H*-azirines induced a selective C–C bond cleavage. This ring-opening event unlocked a transient reactive nitrile ylide intermediate that could be efficiently trapped by various dipolarophiles, such as alkenes, alkynes, and aldehydes, to forge various five-membered heterocycles.

Despite these milestones, the development of heterophotocycloaddition chemistry proceeded relatively slow for decades. While direct photoexcitation enabled a variety of powerful transformations, the discovery of new reaction manifolds was largely dictated by the intrinsic photochemical behavior of the involved substrates. Furthermore, the absence of selective irradiation wavelength was often preventing selectivity in these processes.

A key turning point occurred at the beginning of the twenty-first century with technological progress in selective light-source irradiation (LEDs) and the emergence of visible-light photocatalysis.^35–39^ In particular, the development of photocatalysts capable of mediating single-electron transfer (SET) and energy-transfer (EnT) processes dramatically expanded the synthetic possibilities of photochemistry and created new opportunities for cycloaddition reaction design.^5^ Within the concept of photocycloaddition chemistry, a seminal breakthrough was provided by the pioneering studies of Yoon and co-workers, who demonstrated that visible-light photocatalysis could be successfully integrated with cycloaddition chemistry in the development of a Ru-catalyzed, intramolecular [2 + 2] photocycloaddition.^40^ These discoveries stimulated intense research in the field and contributed to a renewed interest in HPCs. Importantly, although direct photoexcitation remains a widely used and still valuable strategy to trigger HPC reactions, the advent of visible-light photocatalysis has progressively become the dominant approach in the field, leading to a substantial expansion of accessible chemical space and thereby enhancing the applicability, efficiency, and synthetic utility of HPCs. More recently, the combination of photochemical activation with asymmetric catalysis has further enabled enantioselective heterophotocycloadditions, thereby addressing one of the longstanding challenges associated with excited-state reactivity.^41–43^

### 1.1 Aim of This Work

With this review, we aim to provide a comprehensive and critical overview of the developments that have shaped HPC chemistry from the beginning of the new millennium to the present. By collecting, comparing, and critically dissecting the contributions reported during this period we highlight the evolution of the field from the continued development of direct photoexcitation strategies to the rapid expansion of visible-light photocatalysis. Particular attention is devoted to the pioneering studies that first merged photocatalytic activation with HPC chemistry, ultimately leading to the broad range of methodologies available today. In particular, the discussion will be arranged by first addressing methodologies based on direct photoexcitation, followed by photocatalytic approaches, thereby emphasizing their complementary roles in modern heterophotocycloaddition chemistry. Moreover, special attention is devoted to the reaction mechanisms, as this class of transformations encompasses different modes of photochemical and photocatalytic activation.

Over the past years, a number of reviews, accounts, and highlights have appeared in the literature describing selected aspects of HPC chemistry, including specific cycloaddition classes, reactive intermediates, or heterocyclic frameworks.^5,44–54^ However, a comprehensive review encompassing all major classes of HPCs, while critically discussing their photochemical activation modes and mechanistic features, is still lacking. Here, our intention is to fill this gap.

### 1.2 About this Review

The extraordinary growth of photocycloaddition chemistry over the past decades has generated an extensive body of literature concerning the field of HPC, differentiating not only for the type of manifolds, but also for the photochemical activation modes, including direct substrate(s) excitation, electron donor–acceptor (EDA) complex activation, SET and EnT.^17,55^ While this rapid development reflects the vitality of the field, it has also led to a highly heterogeneous landscape in the use of terminology. For example, terms such as cycloaddition and formal cycloaddition are often erroneously employed interchangeably. As discussed above, according to IUPAC, cycloaddition reactions are described as transformations in which two or more unsaturated components combine to form cyclic adducts with a net reduction in bond multiplicity. Importantly, this definition does not imply a specific mechanism. Indeed, cycloadditions may proceed either as pericyclic reactions or through stepwise pathways.^56–58^ In contrast, the expression formal cycloaddition, although frequently encountered in literature, has no standard IUPAC definition and therefore will not be used in this Review. This Review primarily organizes HPCs according to the class of the cycloaddition. In particular, the chapters are organized around hetero-[2 + 2], hetero-[2π+2σ], hetero-(3 + 2) and hetero-[4 + 2] (Figure 1c) Finally, selected cheletropic processes, including representative (n+1)-HPCs, are discussed in a dedicated final section. Throughout this Review we have adopted the IUPAC-recommended notation based on the number of electrons involved in the cycloaddition process, except for 1,3-dipolar and cheletropic cycloadditions for which we have used the more intuitive atom-based notation. During the preparation of this Review, we noticed a surprisingly large number of inconsistencies in the literature, with the two nomenclature systems often being used interchangeably or incorrectly. We hope that the discussion presented herein will help clarify their proper usage and encourage a more consistent terminology in future studies. Importantly, the scope of this Review embraces HPCs, while does not include cascade annulation processes involving nonconjugated systems, or those where the reactive center is not directly conjugated with the π-system within the substrates. These topics fall beyond the scope of the present Review and have been comprehensively covered elsewhere.^44,45,59–61^

Collectively, these classes encompass the majority of contemporary developments in HPC chemistry, thereby providing access to a broad spectrum of heterocyclic molecular architectures. Although organized according to cycloaddition classes, the examples discussed throughout the Review often reveal distinct mechanistic features. Hetero-[2 + 2] photocycloadditions remain largely rooted in the reactivity of electronically excited carbonyl, imine, and alkene derivatives, and constitute the area in which photocatalytic energy-transfer strategies have experienced some of their most significant developments. These methods have emerged as powerful tools for the construction of four-membered heterocycles, including oxetanes, azetidines, azetines, and thietanes. Hetero-[2π+2σ] photocycloadditions represent a comparatively less numerous, yet fast growing and conceptually distinct, class of transformations. This class of reactions often exploits the photochemical activation of strained σ-bonds embedded within bicyclobutanes and related small-ring systems, providing access to unprecedented bicyclic scaffolds that have recently emerged as valuable arene bioisosteres.^48^ Hetero-(3 + 2) photocycloadditions encompass the broadest mechanistic landscape, with direct photoexcitation, photoredox catalysis, and EDA complex activation enabling the generation of a diverse array of reactive 1,3-dipoles and dipolarophiles. As a result, they constitute an exceptionally versatile platform for the synthesis of a wide variety of five-membered nitrogen- and oxygen-containing heterocycles. Finally, hetero-[4 + 2] photocycloadditions are typically enabled by the photochemical generation of highly reactive heterodienes together with dearomative activation strategies, providing efficient access to structurally complex six-membered heterocycles. Importantly, given the still limited number of enantioselective HPCs reported to date, special attention is devoted to these examples.

As these cycloaddition classes are not associated with a single mechanistic landscape, particular emphasis is placed on the photochemical activation mode and mechanistic features of each transformation. This approach allows the reader to navigate the field simultaneously from both synthetic and mechanistic perspectives, highlighting how similar bond-forming outcomes can arise from different reactive intermediates and excited-state activation pathways. Although mechanistic aspects are discussed throughout all chapters, the overall organization varies depending on the reaction class. The chapters referring to hetero-[2 + 2], hetero-[2π+2σ], and hetero-[4 + 2] reactions are primarily structured around the heterocyclic targets, while the hetero-(3 + 2) chapter is organized according to the photochemical activation mode.

For the convenience of the reader, Table 1 provides an overview of the photocatalysts employed throughout this Review, allowing a rapid comparison of the catalytic systems utilized across the various classes of HPCs.

**Table 1:**
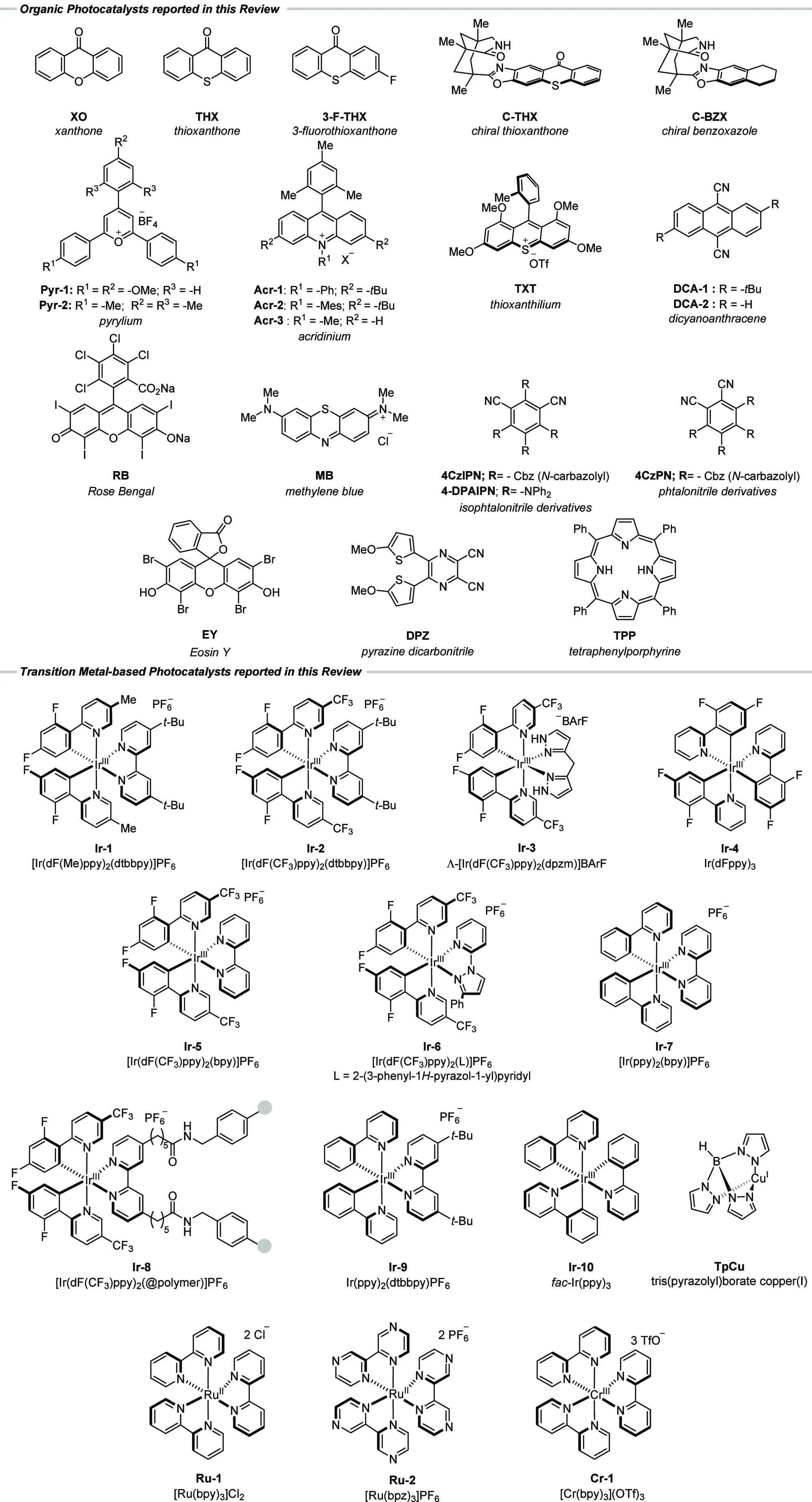
Overview of the Photocatalysts Used in HPCs Discussed in This Work

## 2 [2 + 2]-Cycloadditions

Photochemical hetero-[2 + 2] cycloadditions provide a direct and versatile approach to strained four-membered heterocycles through the combination of two unsaturated two-atom components. Among these, at least one contains a heteroatom that becomes incorporated into the newly formed ring. Depending on the nature of the reacting π-systems, these transformations enable access to oxetanes, azetidines, azetines, oxetenes, thietanes, and related heterocyclic frameworks (Scheme 1).^5,19^ The Paternò–Büchi (PB) reaction between carbonyl compounds and alkenes represents the archetypal member of this family and provided an early conceptual foundation for the development of related processes involving imines, alkynes, and other heteroatom-containing unsaturated components.^23^ As mentioned in the Introduction, rather than revisiting the extensive early photochemical literature, the discussion presented herein begins with representative modern reports from 2000 and progresses through subsequent developments in the field.

**Scheme 1:**
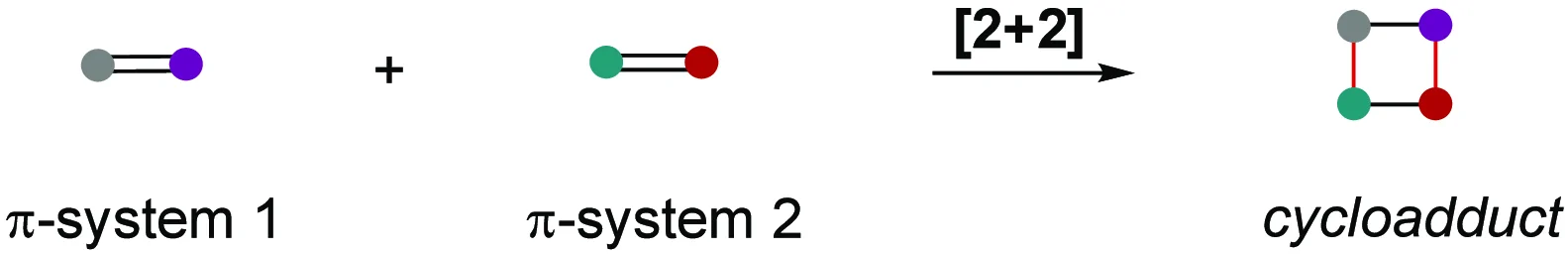
General [2 + 2]-Cycloaddition of Two π-Systems To Form Four-Membered Heterocycles

The mechanistic landscape of hetero-[2 + 2] photocycloadditions is diverse and strongly dependent on the electronic properties of the reacting partners and the mode of photoactivation. Many reactions proceed stepwise following excitation of one component, generating open-shell intermediates that undergo sequential bond formation, intersystem crossing (ISC), and radical recombination. Alternative reaction mechanisms involve exciplexes, charge-transfer complexes, or radical-ion pairs generated by single-electron transfer. Accordingly, modern hetero-[2 + 2] photocycloadditions may be initiated through direct excitation of a reacting substrate, triplet sensitization by EnT, or photoredox activation through SET.^62,63^

Technological advancements have allowed the development of these complementary activation modes and enabled the use of visible light, with improved chemo- and stereoselectivity, and broadened the range of heteroatom-containing π-components suitable for four-membered-ring construction.

Consistent with the scope of this Review, only transformations in which the heteroatom is directly involved in at least one of the newly formed ring bonds are considered. Photocycloadditions occurring exclusively across a C–C π-bond of a heterocyclic structure are therefore excluded. Both intra- and intermolecular reactions are included, together with processes involving alkenes or alkynes and cases in which the initially generated hetero-[2 + 2] cycloadduct undergoes subsequent rearrangement or fragmentation, provided that the connectivity arising from the primary cycloaddition remains clearly identifiable. The material is organized according to the nature of the heterocyclic framework generated in the primary photochemical event, with each class further subdivided by activation mode, including direct excitation, EDA-complex activation, photosensitized EnT, and photoredox SET pathways.

### 2.1 Oxetanes

#### 2.1.1 Direct Irradiation Methods

Direct excitation of carbonyl compounds represents the classical activation mode for PB reactions and provides a direct route to oxetane frameworks. This strategy is governed by the intrinsic absorption properties of the carbonyl substrate: aliphatic and aromatic ketones typically absorb in the UV region, whereas only selected classes, including quinones and 1,2-dicarbonyl compounds, show appreciable absorption in the visible range. Following n−π* excitation, carbonyl compound **1** is promoted to a singlet excited state and generally undergoes rapid ISC to the corresponding triplet state **1-T**_**1**_. Addition to alkene **2** then generates a triplet 1,4-diradical, which undergoes intersystem crossing and ring closure to furnish oxetane **3** (Scheme 2). In some cases, particularly with aliphatic ketones under UV irradiation, cycloaddition may also occur directly from the singlet excited state when intersystem crossing is comparatively slow.

**Scheme 2:**
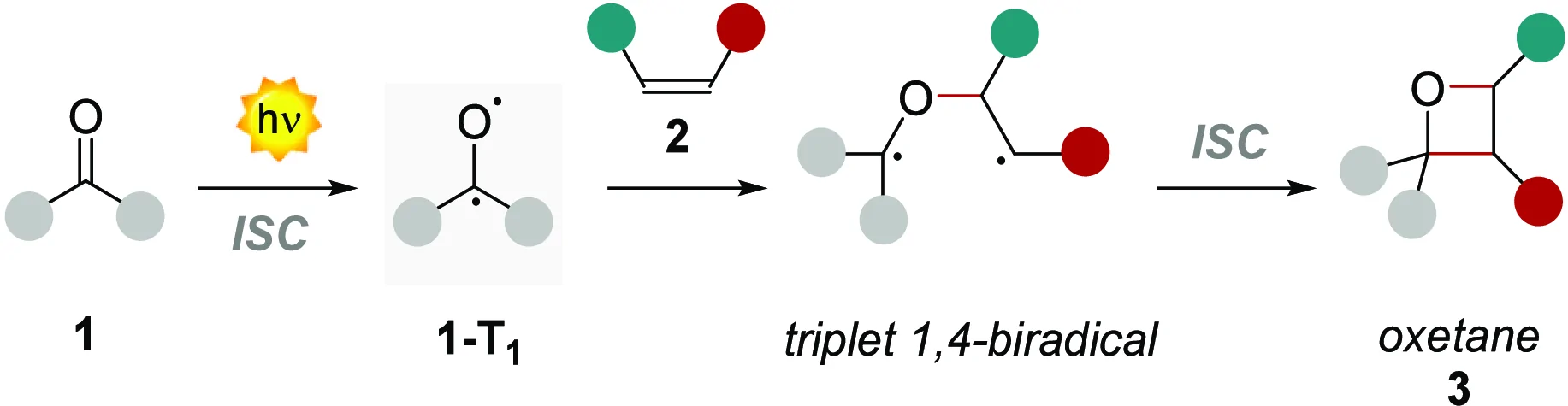
General Direct-Excitation Paternò–Büchi Pathway from Carbonyl Compounds and Alkenes to Oxetanes

Abe and co-workers reported in 2000 a representative example of modern hetero-[2 + 2] photocycloaddition involving 2-siloxyfurans **4** and carbonyl compounds **5**.^64^ Upon direct UV irradiation (λ > 290 nm), these substrates underwent PB cycloaddition to furnish the bicyclic oxetanes **6–7** in high yield (Scheme 3). This study highlighted the pronounced influence of both the furan **4** substitution pattern and the carbonyl **5** excited-state multiplicity on the regioselectivity of the process. While aldehydes generally provided nearly statistical mixtures of regioisomers **6** and **7**, triplet-excited ketones preferentially delivered oxetane **7**. Notably, high *exo* selectivity was observed throughout the study, indicating that stereoselectivity is less sensitive to these electronic and structural variations than regioselectivity. Mechanistically, the reaction proceeds through the addition of the excited carbonyl species (e.g., **5-T**_**1**_) to the electron-rich furan **4**, followed by formation of a 1,4-diradical intermediate **8** whose regio- and stereochemical outcome is governed by productive intersystem crossing and cyclization to the *exo*-oxetane products **6** or **7**.

**Scheme 3:**
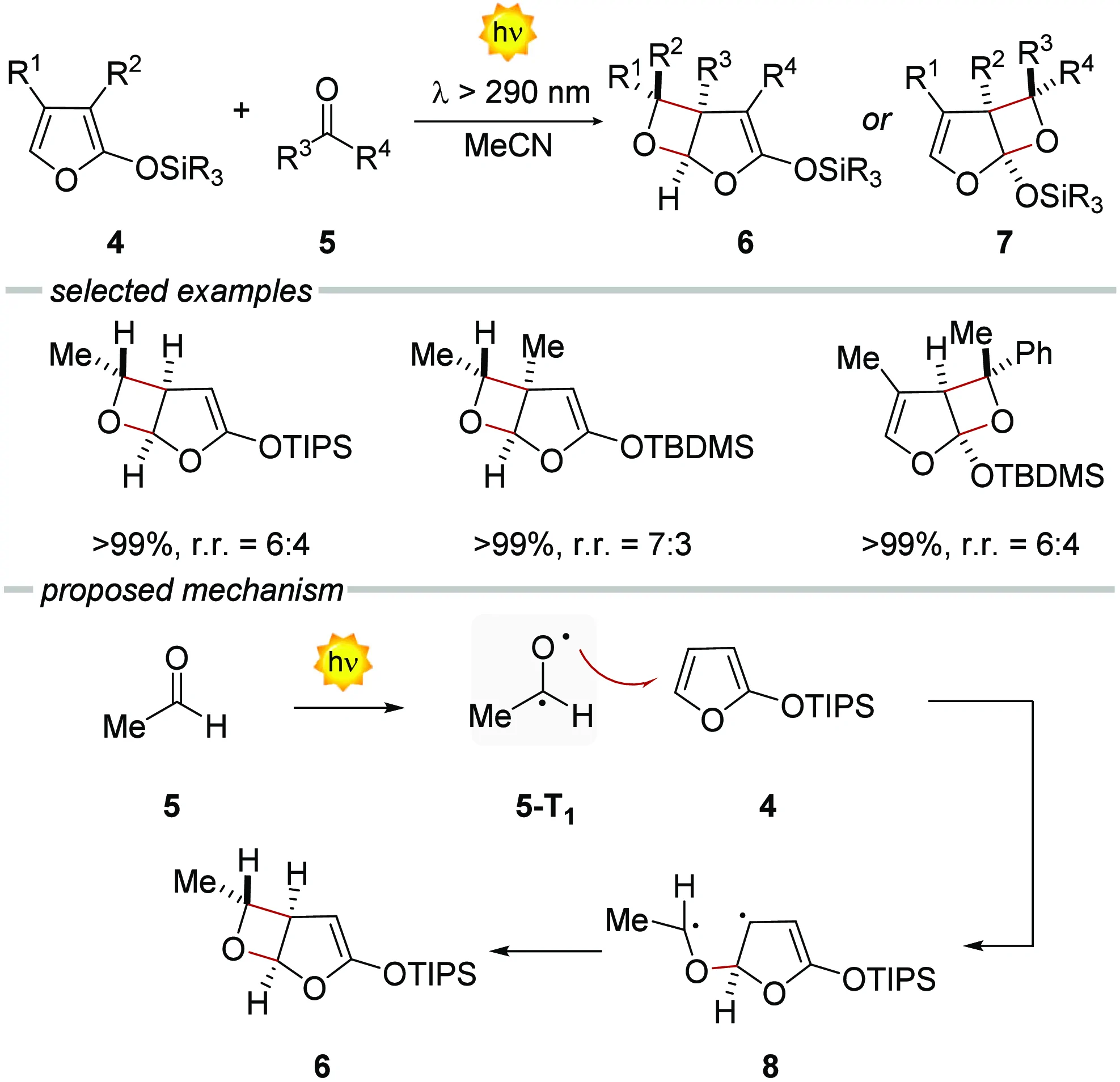
Direct PB Cycloadditions of 2-Siloxyfurans with Carbonyl Compounds To Access Bicyclic Oxetanes

Related PB reactions of heteroatom-substituted electron-rich alkenes were later explored in both synthetic and mechanistic contexts. Booker-Milburn and co-workers used a flow-photochemical PB reaction between a *D*-isosorbide-derived bicyclic enol ether and benzaldehyde to access oxetane intermediates on multigram scale en route to (+)-goniofufurone, illustrating the scalability of otherwise slow and dilute enol ether photocycloadditions.^65^ Complementary process-oriented studies by Kakiuchi and co-workers further showed that slug-flow microreactor conditions, using water as an inert segmented phase, can improve the efficiency and productivity of model PB reactions, underscoring the practical value of flow technologies for light-limited hetero-[2 + 2] photocycloadditions.^66^ D’Auria and co-workers investigated 5-oxazolylmethanol derivatives, showing that the hydroxyl directing effect previously observed with allylic alcohols and furylmethanols can also operate with oxazoles, leading to unusual endoselective oxetane formation with aromatic aldehydes.^67^ Finally, Abe and co-workers extended their siloxyheterocycle studies from furans to 2-siloxy-1*H*-pyrroles, where benzophenone afforded regioselective bicyclic oxetanes; laser flash photolysis and computational studies supported a triplet-diradical pathway in which product formation is governed by the distribution of productive diradical conformers.^68^

In a complementary report, Adam and Stegmann disclosed an initial hydroxy-directed variant of the PB reaction between chiral allylic alcohols **9** and benzophenone **10**.^69^ Upon UV irradiation (λ = 350 nm), the excited aromatic ketone **10** underwent regio- and diastereoselective hetero-[2 + 2] cycloaddition with the allylic alcohols **9**, affording oxetanes **11** with high preference for the *syn* diastereomer (Scheme 4). The observed selectivity was rationalized through a hydrogen-bonded exciplex, in which the allylic hydroxy group directs the approach of the excited carbonyl partner **10**, while steric interactions within the allylic substrate **9** favor a reactive conformation leading to the *syn* oxetane **11**. Consistently, disruption of hydrogen bonding in protic solvent or masking of the hydroxy group as a silyl ether led to a pronounced erosion of stereoselectivity. In a subsequent expanded study, the same authors extended this hydroxy-directed strategy to a broader set of chiral and achiral allylic substrates **9** and aromatic carbonyl compounds **10**, including acetophenone and benzaldehyde, enabling the preparation of side-chain-functionalized oxetanes **11** bearing up to three stereogenic centers.^70^

**Scheme 4:**
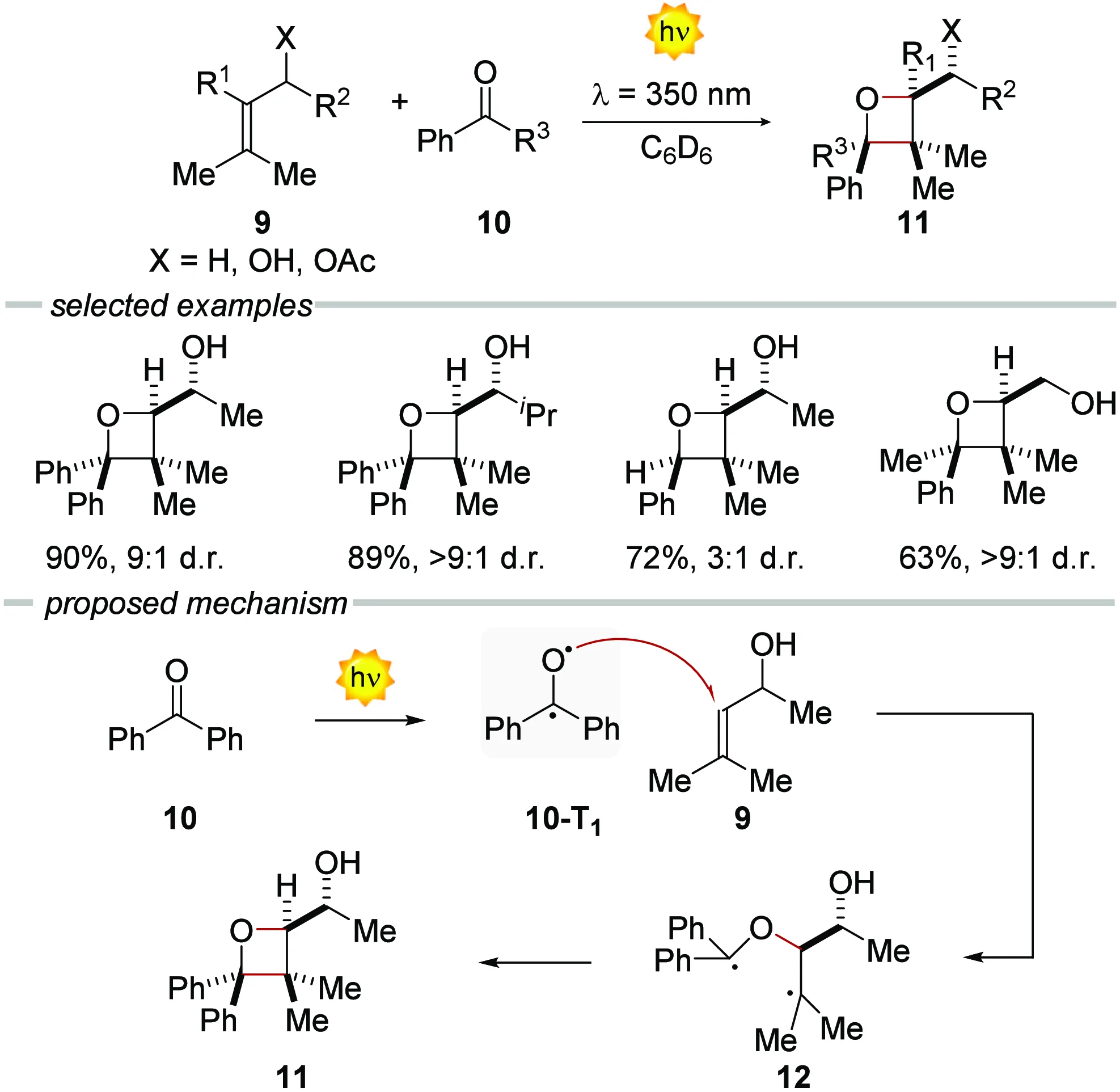
Hydroxy-Directed PB Cycloadditions of Allylic Alcohols with Aromatic Carbonyl Compounds

During the same period, Bach and co-workers developed a closely related series of PB reactions involving nitrogen-containing electron-rich alkenes as a photochemical carbohydroxylation strategy.^71^ In their initial study, *N*-protected 2,3-dihydropyrroles **13** were reacted with aromatic carbonyl compounds **14** under irradiation to afford bicyclic amino-oxetanes **15**, which could be further converted by hydrogenolysis into pyrrolidinol derivatives, including intermediates relevant to the synthesis of preussin-type alkaloids (Scheme 5). Mechanistically, these transformations follow the typical carbonyl–alkene hetero-[2 + 2] pathway, in which the excited carbonyl compound **14** adds to the electron-rich enecarbamate **13** double bond to generate a triplet 1,4-diradical species **16** that undergoes intersystem crossing and cyclization to the oxetane product **15**. This platform was subsequently expanded in two complementary directions: acyclic α-alkyl enecarbamates were shown to undergo regioselective photocycloaddition with benzaldehyde to provide 3-alkyl-3-aminooxetanes, while 3,4-dihydro-2-pyridones enabled access to bicyclic lactam-derived oxetanes that could be opened to 2-arylmethyl-3-piperidinol scaffolds. Together, these studies established PB photocycloaddition as a versatile entry to nitrogen-containing oxetanes and amino alcohol derivatives.^72,73^ This chemistry was later revisited by Bach and co-workers in a practical synthetic procedure, which summarized the use of silyl enol ethers and enamides as heteroatom-substituted alkene partners in PB reactions, providing direct access to 3-silyloxy- and 3-aminooxetanes that can be further elaborated through ring-opening chemistry.^74^

**Scheme 5:**
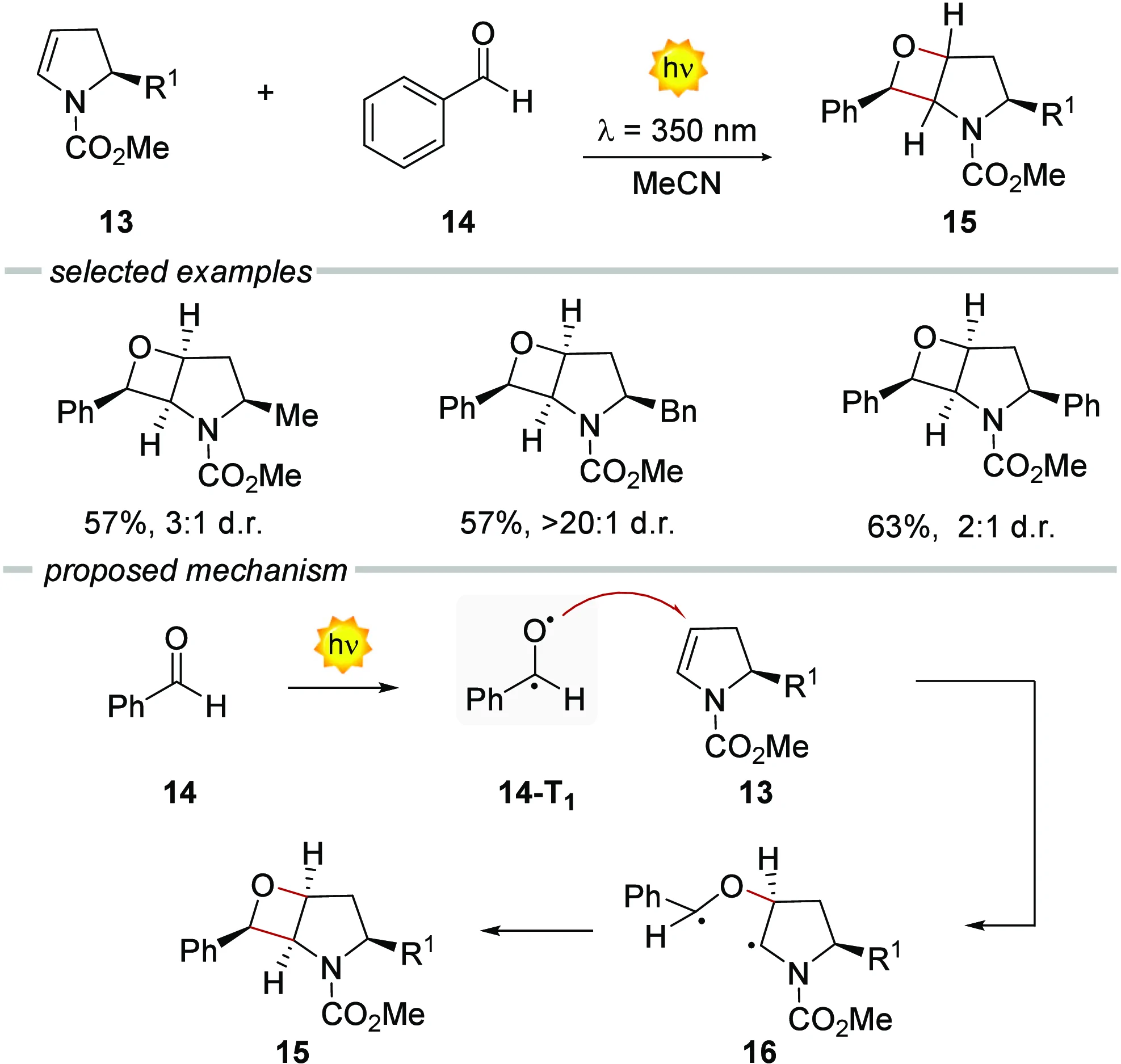
PB Cycloadditions of Nitrogen-Substituted Electron-Rich Alkenes with Aromatic Carbonyl Compounds

The synthetic relevance of this approach was later illustrated by Aitken, Boddaert and co-workers in a concise synthesis of all four stereoisomers of oxetin.^75^ Glyoxylate esters were reacted with *N*-vinylformamide or protected vinyl carbamate derivatives under UV irradiation to afford 3-amino-2-oxetanecarboxylate intermediates with complete regioselectivity but limited *cis*/*trans* control. Subsequent separation and resolution strategies enabled gram-scale access to enantiomerically pure oxetin and epi-oxetin stereoisomers, highlighting the utility of PB chemistry in the synthesis of strained β-amino acid derivatives.

Besides these reports, several additional studies expanded the mechanistic and substrate scope of PB-type hetero-[2 + 2] photocycloadditions. Scheffer and co-workers reported an unusual solid-state variant, in which aryl cyclopentyl ketones furnished oxetanes in the crystalline state rather than the Norrish type I products observed in solution.^76^ Adam and Stegmann investigated the temperature-dependent *cis*/*trans* selectivity in ketone–cyclooctene photocycloadditions, highlighting the role of preoxetane diradical conformations, cyclization, and cleavage.^77^ Further substrate-specific investigations described the regio- and diastereoselective formation of spiroindoline oxetanes from 1-acetylisatin and enol ethers, chiral phenylglyoxylate–furan photocycloadditions, and captodative olefin additions to monosubstituted benzils.^78–80^ A later study by Xu and co-workers further highlighted the mechanistic flexibility of 1,2-dicarbonyl compounds in reactions with oxazoles, where [2 + 2], [4 + 2], and [4 + 4] pathways could compete depending on the carbonyl partner and oxazole substitution pattern; notably, benzil afforded only PB-type [2 + 2] oxetane products.^81^ Related reactivity was observed in the photochemical coupling of para-quinones with bicyclopropylidene, where benzoquinones initially furnished spirooxetanes that could undergo further irradiation-induced rearrangement, while naphtho- and anthraquinones displayed substrate-dependent competition between oxetane formation and alternative polycyclic pathways.^82^ Zhang, Xu and co-workers subsequently showed that strained bicycloalkylidenes undergo PB spirooxetane formation with isatin and isoquinolinetrione, although competing 1,6-diradical pathways led to unusual rearranged products and higher-order cycloadducts.^83^ Together, these reports emphasized how reaction medium, substrate organization, excited-state character, and diradical stability govern selectivity in carbonyl–alkene hetero-[2 + 2] photocycloadditions.

Later mechanistic and stereochemical studies further clarified how oxetane-forming hetero-[2 + 2] photocycloadditions can be controlled by subtle changes in reaction conditions and excited-state pathways. Song and co-workers investigated PB reactions of dimethyluracil and dimethylthymine with benzophenone derivatives, showing that temperature, substituent effects, and solvent composition strongly influence regioselectivity through changes in the stability and conformational behavior of triplet 1,4-diradical intermediates.^84–86^ Abe and co-workers similarly highlighted temperature-dependent stereoselectivity in the reaction of 2,3-dihydrofuran-3-ol derivatives with benzophenone.^87^ Inoue and co-workers later demonstrated that the stereochemical outcome of chiral cyanobenzoate–alkene PB reactions can be switched by changing the irradiation wavelength, distinguishing direct excitation from charge-transfer-complex excitation.^88,89^ Related studies by Mariano, Yoon and Sivaguru further emphasized, respectively, the competition between SET and oxetane formation, and the role of substrate preorganization and axial-to-point chirality transfer in intramolecular PB reactions.^90,91^

In 2020, Dell’Amico and co-workers reported a modern visible-light variant of direct PB chemistry through the dearomative hetero-[2 + 2] photocycloaddition of indoles **17** with aromatic ketones **18**.^92^ Irradiation with 405 or 465 nm LEDs enabled oxetane formation across the indole C2–C3 π-bond, affording highly diastereoselective oxetane-fused indolines **19** (Scheme 6). For benzophenone-derived partners, excitation at the weak visible absorption tail was sufficient to promote the reaction while limiting benzopinacol formation. Quenching studies with the indole, inhibition by oxygen or DABCO, the absence of detectable substrate association, and unfavorable photoinduced electron transfer (PET) energetics supported a triplet diradical pathway involving addition of the excited ketone **18** to the indole π-system and cyclization of intermediate **20** to the oxetane product **19**. By contrast, benzil-derived partners could also operate through a PET manifold, consistent with their more favorable redox properties. This study expanded noncatalytic visible-light PB chemistry to the dearomative functionalization of indoles.

**Scheme 6:**
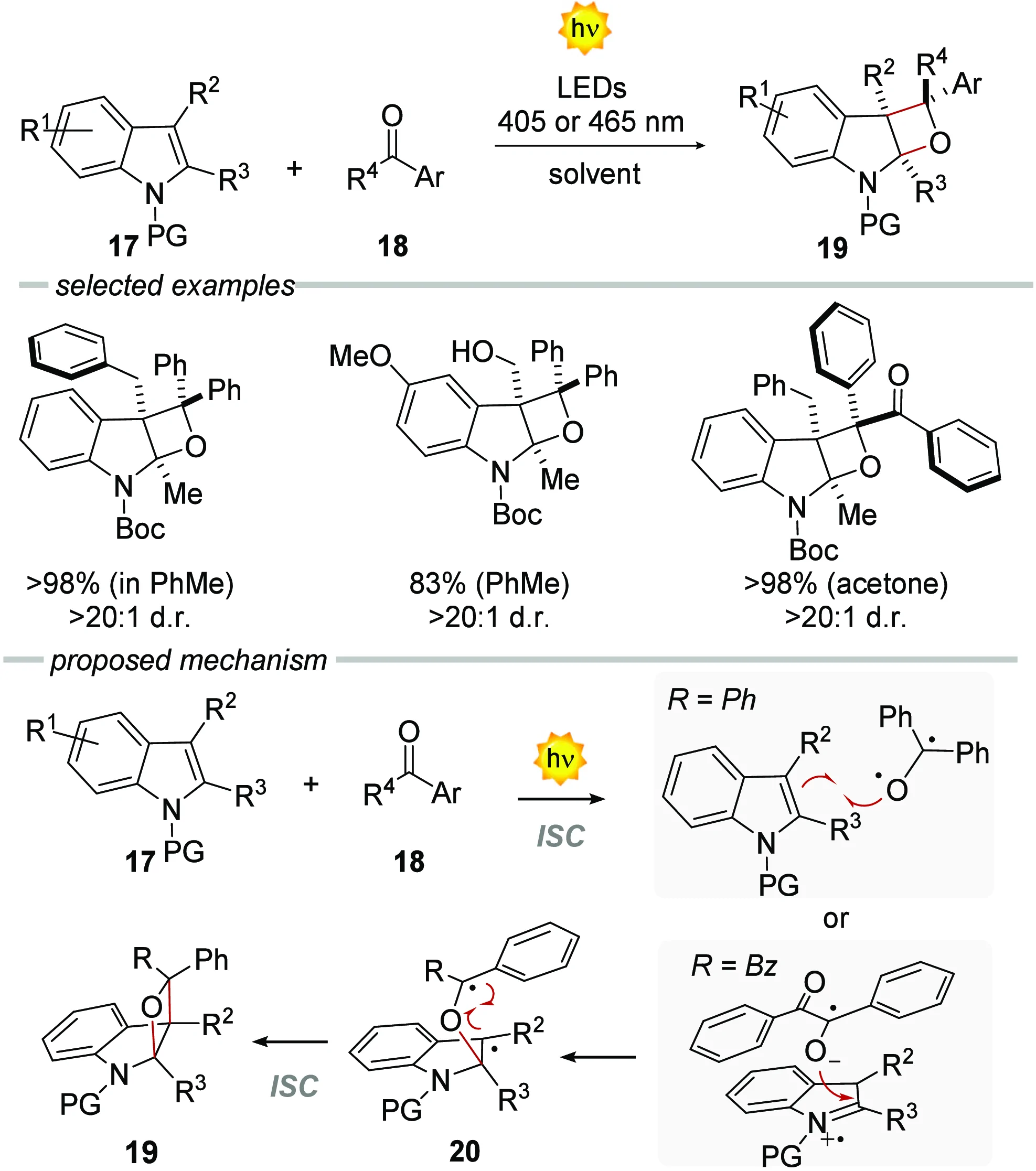
Visible-Light-Induced Dearomative PB Cycloaddition of Indoles with Aromatic Ketones

In a follow-up study, the same group extended this visible-light PB strategy to indole enol ethers under flow conditions.^93^ Oxindole-derived enol ethers were engaged with substituted benzophenones to afford oxetane-fused indoline products with high chemoselectivity, with no competing addition to pendant allylic or furan double bonds. The use of flow irradiation markedly improved reaction efficiency, enabling near-quantitative product formation within minutes compared with much lower conversion under batch conditions. Mechanistic experiments again supported direct excitation of the benzophenone partner followed by a triplet diradical pathway, with no evidence for ground- or excited-state substrate association and an energetically disfavored PET process.

Between 2022 and 2024, several catalyst-free visible-light protocols further broadened the scope of direct PB chemistry beyond indole-based substrates. Capaldo, Ravelli and co-workers showed that benzils can undergo blue-light-induced cycloadditions with simple cyclic and acyclic olefins, with wavelength and temperature playing a key role in suppressing benzil decomposition and competing [4 + 2] or hydrogen-atom-transfer pathways.^94^ Zhu and co-workers reported a more general white-light-mediated reaction of dicarbonyl compounds with styrenes, benzofurans, cyclic styrenes, and aliphatic alkenes, providing multisubstituted oxetanes through direct carbonyl excitation.^95^ In a complementary study, Su and co-workers exploited the visible-light absorption of anthraquinones to access anthraquinone-based spirocyclic oxetanes with high regioselectivity under mild conditions.^96^ Finally, Basso and co-workers extended this logic to β-lactone synthesis by combining visible-light Wolff rearrangement of α-diazoketones with excitation of benzils, enabling a doubly photochemically activated PB-type reaction with photogenerated ketenes under flow conditions.^97^ Together, these studies illustrate how direct visible-light excitation of suitable carbonyl partners can expand classical PB reactivity without relying on external photocatalysts.

Fluorinated alkenes have also been exploited as distinctive partners in direct PB reactions and related cascade processes. In 2021, Hoffmann and co-workers investigated the irradiation of aromatic ketones **18** with fluorinated alkenes **21**, leading to fluorinated oxetanes **22** and **23** as the main products together with minor amounts of monofluorinated alkenes **24** (Scheme 7).^98^ The latter were proposed to arise from secondary fragmentation of the initially formed oxetanes, corresponding overall to a photo-Wittig-type carbonyl–olefin metathesis process. Computational studies indicated that the unusual regioselectivity of the cycloaddition could not be rationalized solely from ground-state alkene polarization, but rather reflected the relative stability of the possible 1,4-diradical intermediates. Building on this concept, Shen and co-workers later developed a one-pot photothermal cascade between aromatic ketones **25** and aromatic *gem*-difluoroalkenes **26**.^99^ In this case, initial PB cycloaddition furnishes *gem*-difluorooxetane intermediate **28**, which undergoes thermally promoted retro-[2 + 2] fragmentation to triarylalkene **29**, followed by photoinduced 6π electrocyclization and collidine-assisted dehydrogenative aromatization to phenanthrenes **27**. Although the final products are no longer heterocyclic, this cascade is relevant in the context of hetero-[2 + 2] photochemistry because the oxetane-forming PB step constitutes the key photochemical bond-forming event.

**Scheme 7:**
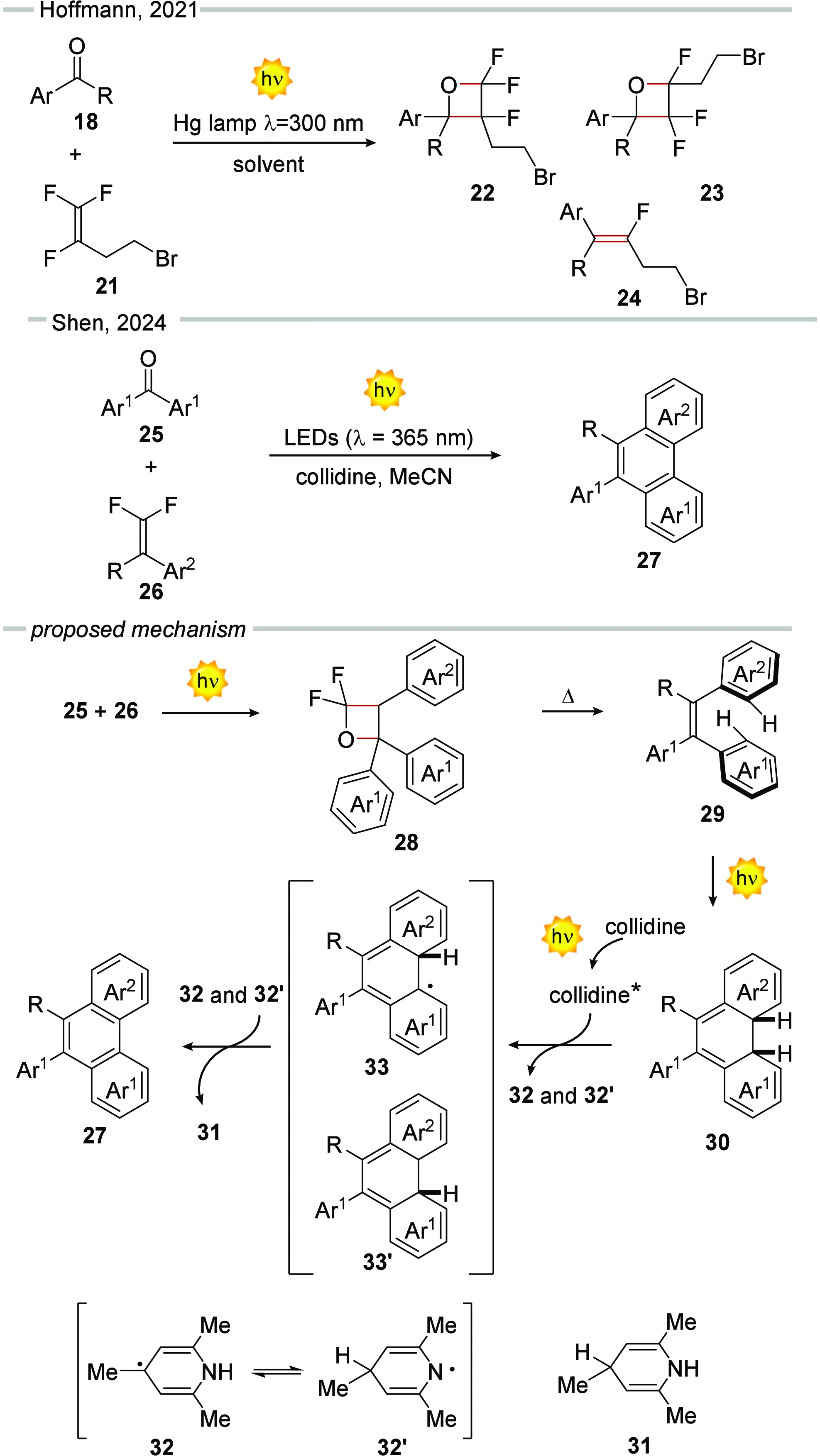
Direct PB Reactions of Fluorinated Alkenes and Gem-Difluoroalkenes in Oxetane-Forming Cascade Processes

Beyond preparative synthesis, direct PB photochemistry has also found important applications in structural lipidomics. In this context, UV-induced reactions between carbonyl reagents and lipid C = C bonds generate oxetane adducts that undergo characteristic retro-PB fragmentation under tandem MS conditions, enabling localization and quantification of double-bond positional isomers.^100^ Since the development of PB-MS/MS workflows, including shotgun lipidomics, imaging, single-cell analysis, and optimized carbonyl reagents, has been extensively reviewed elsewhere, these analytical applications are not discussed in detail here.^101^

Through a distinct mechanistic strategy, the Paternò–Büchi reaction can also be triggered by the selective excitation of the alkene partner instead of the carbonyl chromophore. Besides obviating to the need of a carbonyl compound able to absorb light, such a switch in the excited state nature of the substrate from an n,π* to a π,π* triplet might help avoiding side reactions that are typically associated with the excited-state chemistry of carbonyl chromophores such as hydrogen abstraction, disproportionation, and pinacol coupling. Following this approach, in 2017 the Sivaguru group reported the first example of what they termed a “transposed Paternò–Büchi” reaction.^102^ Here, they applied this concept to the intramolecular cycloaddition of atropoisomeric enamides **34** to give complex oxetane **35** (Scheme 8). The reaction occurs through the sensitization of the enamide moiety under UV-light irradiation using either acetone both as solvent and photosensitizer or xanthone **XO** as photocatalyst. Importantly, performing the reaction with atropoisomerically enriched enamides enables axial to point chirality transfer, giving access to enantioenriched oxetane. A combination of laser flash photolysis experiments and DFT calculations enabled to ascertain the role of the enamide moiety as chromophore.

**Scheme 8:**
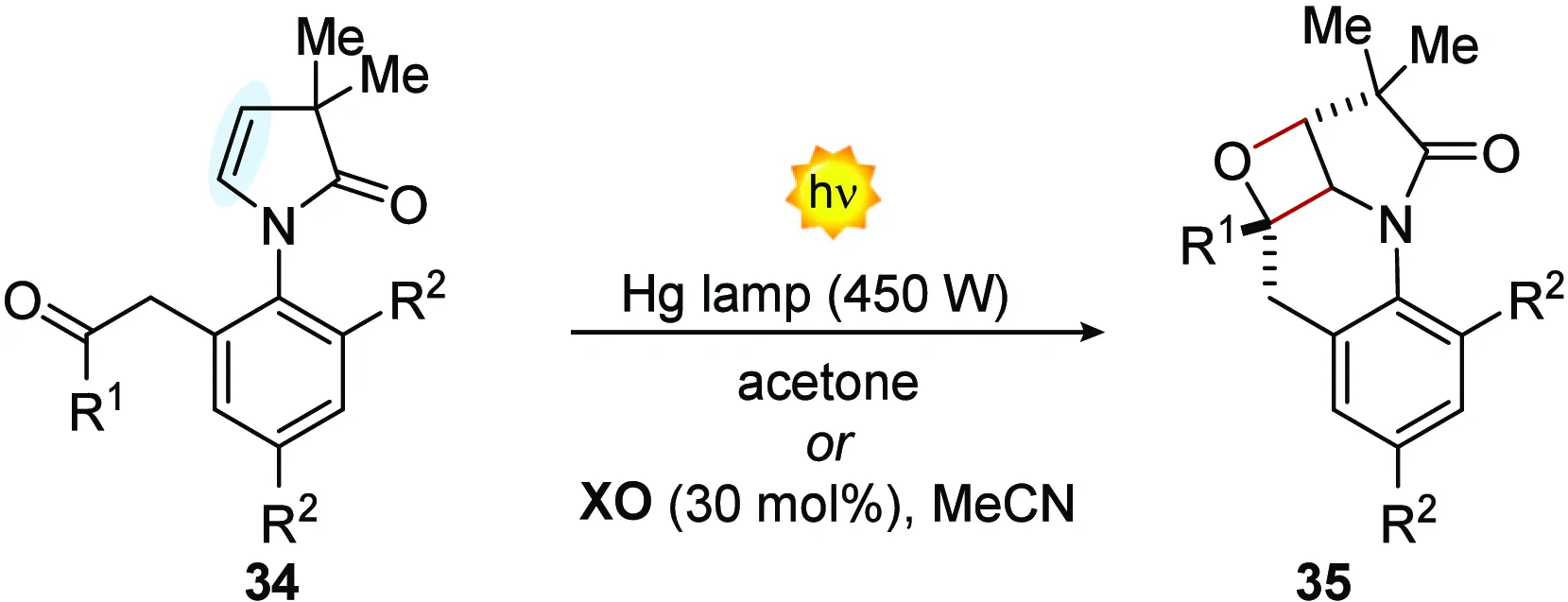
Intramolecular Transposed Paternò–Büchi Reaction of Atropoisomeric Enamides

A step further in the generalization of this approach was marked a couple of years later by Schmidt and co-workers.^103^ Inspired by seminal works on Cu-catalyzed photodimerization of alkenes,^104–107^ they reported the use of a Cu catalyst to enable the PB reaction between norbornene **36** and unactivated carbonyls **5**, whose photoexcitation would normally require highly energetic UV–C light (Scheme 9). In contrast, irradiation with a 100 W Hg lamp in the presence of a Cu­(I) tris­(pyrazolyl)­borate (Tp) complex allowed to engage in [2 + 2] cycloadditions a variety of both cyclic and linear aliphatic ketones, giving the corresponding cycloadduct **37** with exquisite *exo*-selectivity. Through a series of spectroscopic studies, the authors proved that the reaction is unlocked by the formation of Cu-norbornene complex **TpCu-36**, a photoactive species that effectively absorbs UV–B light. Therefore, excitation between 280–300 nm results in a metal-to-ligand charge transfer (MLCT) to generate a Cu­(II) metal center and an alkene radical anion. Then, such excited state complex TpCu-36* reacts in a [2 + 2] carbonyl–olefin cycloaddition to give product **37** and restore the ground state Cu­(I) catalyst. The authors proposed that this final step may involve a Cu­(III) oxametallacycle intermediate, a mechanistic hypothesis later corroborated by DFT calculations from the Cui group, who also elucidated the origin of the *exo*-selectivity observed in the reaction.^108^

**Scheme 9:**
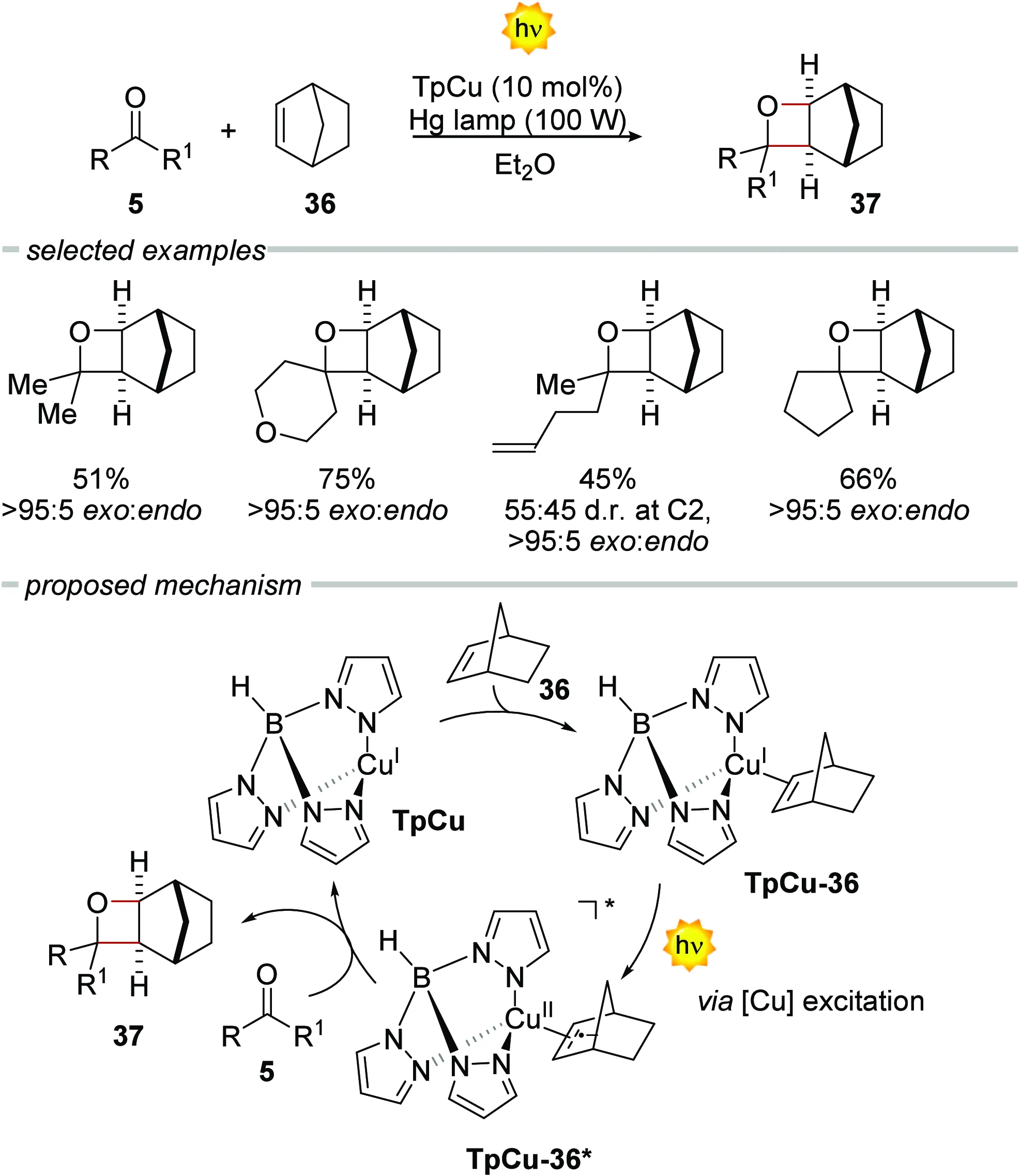
Cu-Catalyzed Transposed PB Reaction of Norbornene and Ketones

A related PB reaction involving norbornene was described a few years later by the Pan group, who used a catalytic aryl ketone as photosensitixer.^109^

#### 2.1.2 EDA-Complex Photochemistry

A distinct visible-light activation mode was recently introduced by Ouyang and co-workers, who exploited electron donor–acceptor (EDA) complex photoexcitation for PB-type oxetane formation.^110^ In this analytical platform, benzophenone **38** associates with unsaturated lipids **39** to generate an EDA complex **42**, whose irradiation with visible light triggers photoinduced electron transfer to give radical-ion pair **43** and, ultimately, oxetane regioisomers **40** and **41** (Scheme 10). Although not developed as a preparative method, this transformation proved valuable for lipid structural analysis: collision-induced dissociation of the oxetane adducts induces retro-PB fragmentation, producing diagnostic ions **44** and **45** that enable localization of the original C=C bond within the lipid chain. This example highlights how noncatalytic visible-light activation of substrate complexes can expand hetero-[2 + 2] photochemistry beyond conventional direct UV excitation. Related visible-light PB-MS/MS approaches based on anthraquinone reagents have also been developed for structural lipidomics, further illustrating the analytical value of visible-light hetero-[2 + 2] oxetane formation.^111^

**Scheme 10:**
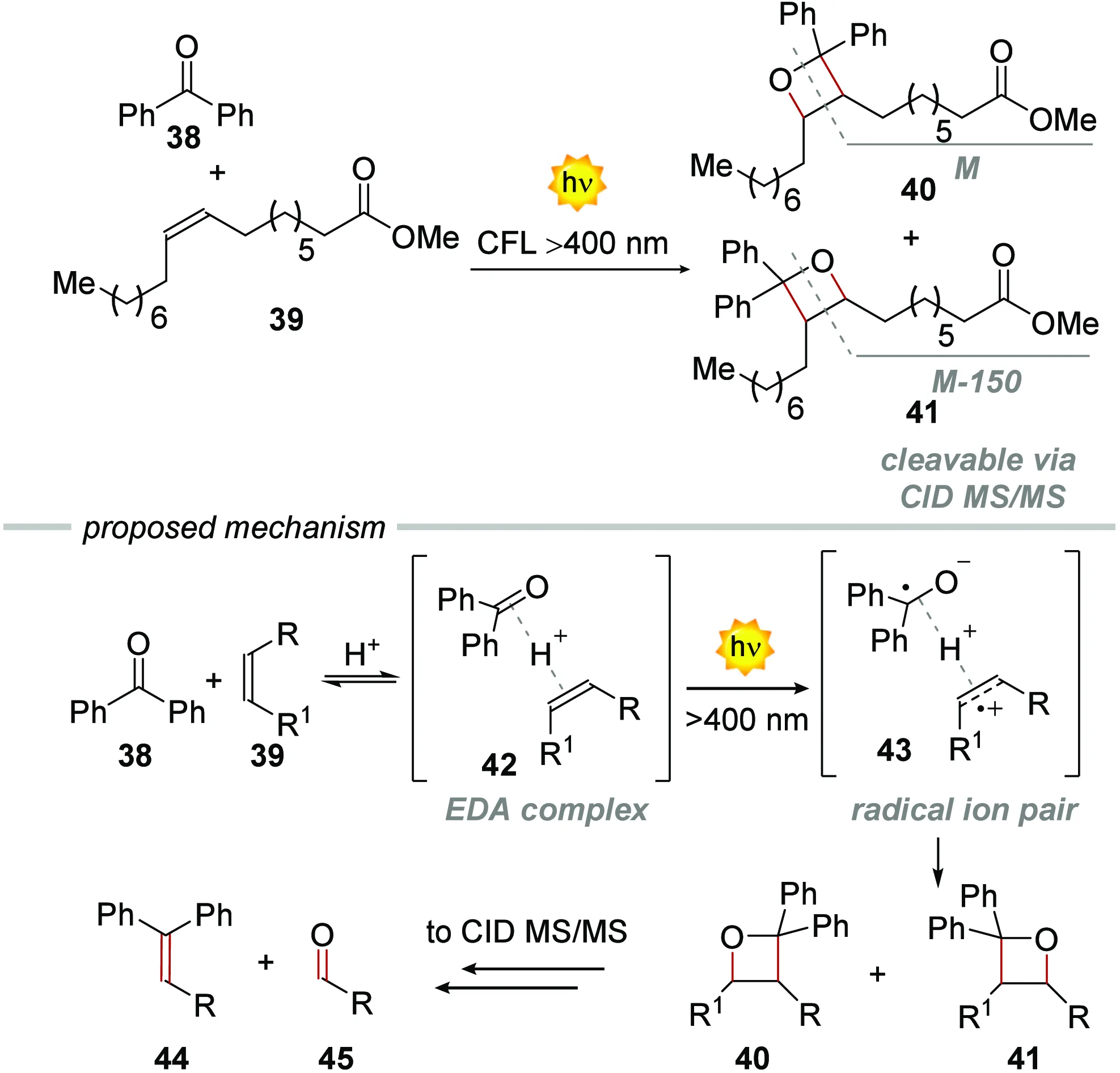
Visible-Light Activation of Benzophenone–Lipid EDA Complexes for Oxetane Formation and Double-Bond Localization

In 2023, Dell’Amico and co-workers further showed that substrate-complex photoactivation can be used to control the stereochemical outcome of visible-light PB reactions.^112^ Biorelevant indole derivatives **46** were reacted with prochiral α-dicarbonyl compounds **47** to afford oxetane-fused indolines **48** as *endo* or *exo* cycloadducts depending on the operative excited-state pathway (Scheme 11). Mechanistic investigations combining DFT calculations, UV–vis and emission spectroscopy, and transient absorption spectroscopy revealed two competing manifolds. Under 370 nm irradiation, direct excitation of the carbonyl partner promotes formation of an exciplex **49**, in which an n−π interaction enforces a nearly perpendicular arrangement of the two substrates and leads selectively to the endo cycloadduct. By contrast, irradiation at longer wavelength selectively activates the EDA complex **50**, where π–π association places the indole and dicarbonyl components in a different relative geometry, strongly altering the exo/endo outcome. This study demonstrated that subtle changes in light wavelength and substrate structure can switch between exciplex- and EDA-controlled PB pathways, providing a rare example of stereochemical control through selective excitation of distinct substrate assemblies.

**Scheme 11:**
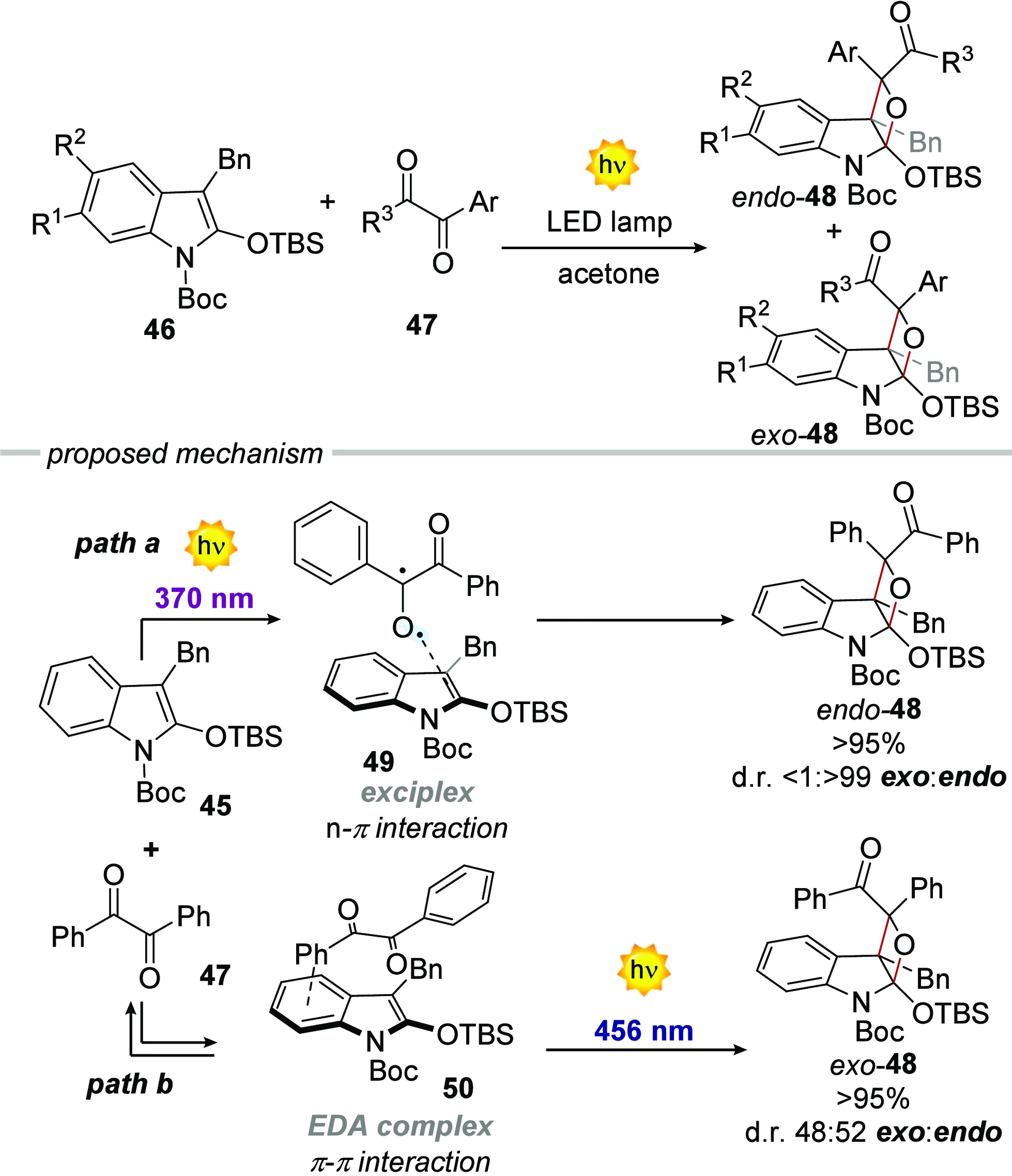
Wavelength-Controlled PB Cycloadditions of Indoles with α-Dicarbonyl Compounds through Direct and EDA-Complex Excitation Pathways

#### 2.1.3 Photocatalytic Methods

In 2020, Schindler and Yoon independently reported triplet-sensitized PB reactions of α-ketoesters **51** with alkenes **52** under visible-light photocatalysis.^113,114^ Using the high-energy iridium photocatalysts under blue LED irradiation, energy transfer to the α-ketoester component generated the corresponding triplet carbonyl species, which engaged a range of electronically diverse alkenes to afford oxetanes **53** (Scheme 12). This activation mode avoids the need for direct excitation of the carbonyl substrate, provided that its triplet energy is compatible with that of the photocatalyst. Mechanistically, the reaction was proposed to proceed through triplet α-ketoester addition to the alkene, initial C–O bond formation, and generation of 1,4-diradical intermediate **54**, followed by intersystem crossing and radical recombination to deliver the oxetane product **53**. In a related study, Yoon and co-workers showed that the same photocatalytic platform could be diverted under Lewis acid conditions toward allylic alcohol products, highlighting the sensitivity of α-ketoester–alkene photochemistry to substrate activation and reaction environment.

**Scheme 12:**
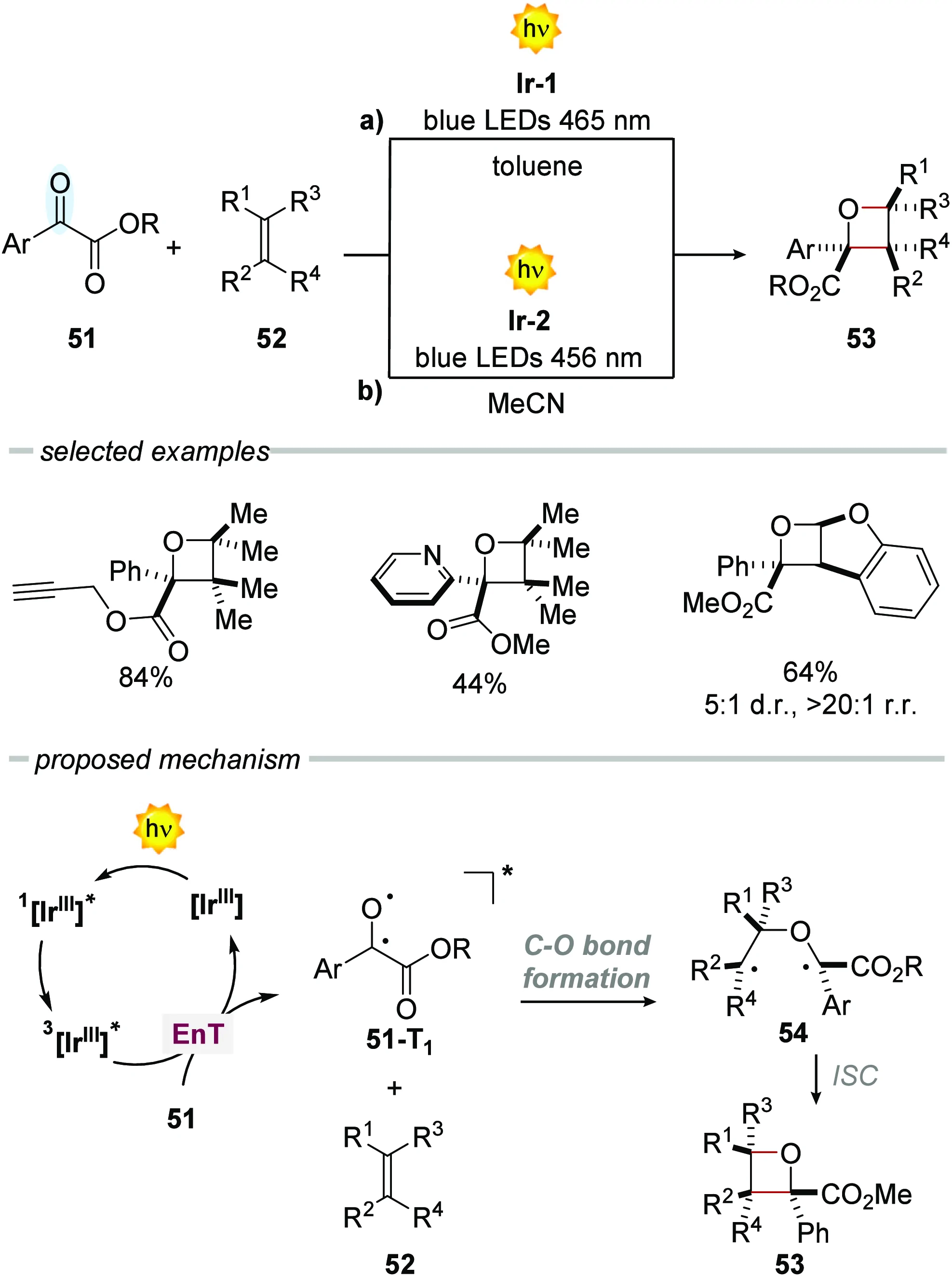
Triplet-Sensitized PB Reactions of α-Ketoesters with Alkenes

In 2024, Yoon and co-workers addressed the long-standing challenge of catalytic enantioselective PB reactions through a triplet-rebound strategy.^115^ Using a chiral hydrogen-bonding iridium photosensitizer **Ir-3** under blue-light irradiation, adamantyl benzoylformate derivatives **55** were coupled with hydrogen-bonding quinolones **56** to afford oxetane-fused products **57** with high diastereo- and enantioselectivity (Scheme 13). Mechanistic studies showed that the chiral Ir catalyst binds the quinolone partner **56** through hydrogen bonding, while the α-ketoester **55** is activated by Dexter energy transfer without ground-state association to the catalyst. The resulting triplet carbonyl species **55-T**_**1**_ can then react within the initial solvent cage with the catalyst-bound quinolone, enabling stereocontrol through a triplet rebound pathway. Slow addition of the quinolone and careful control of solvent and oxygen effects minimized unselective background pathways, allowing access to enantioenriched oxetanes with excellent selectivity. This study demonstrated that asymmetric control over native, unbound excited carbonyl species can be achieved without perturbing their intrinsic n,π* PB reactivity.

**Scheme 13:**
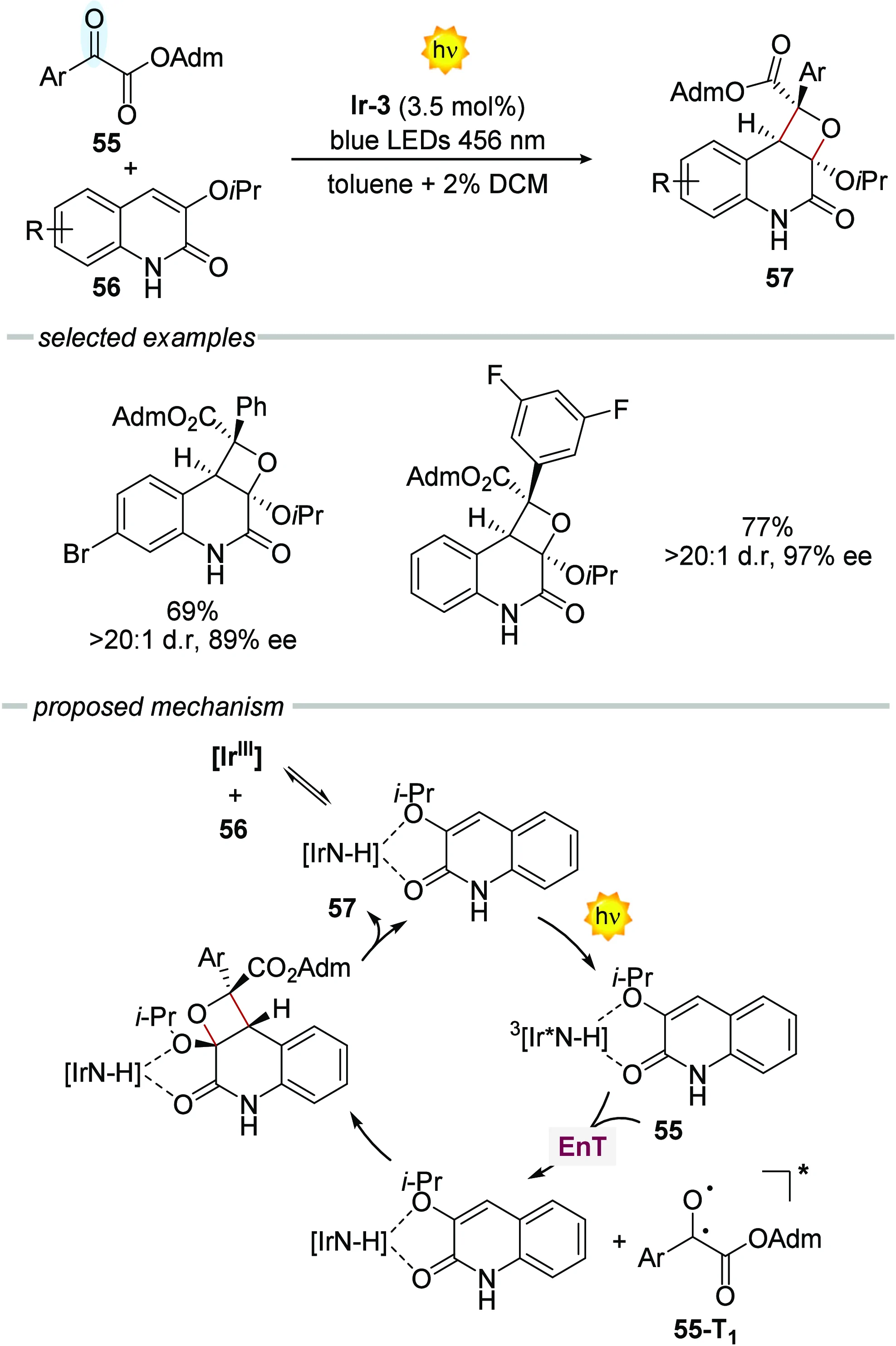
Enantioselective Energy-Transfer PB Reaction Controlled by a Chiral Hydrogen-Bonding Photosensitizer

Finally, Yoon and co-workers showed that 3-alkoxyquinolin-2­(1*H*)-ones **58**, indenes **59**, and 1-(hetero)­aryl cyclohexenes **60**, all compounds with relatively low lying triplet energies, underwent transposed PB cycloaddition with aromatic aldehydes and ketones under 427 nm irradiation using **Ir-2** as photosensitizer (Scheme 14).^116^ The triplet energy of this photocatalyst lies between that of the alkenes and the aldehydes, which enables the selective sensitization of the alkene partner as corroborated by Stern Volmer quenching studies and by the detection of alkene dimerization side-products. While this study significantly expands the plethora of alkene available for the transposed PB photocycloaddition, the sensitivity of the reaction to the substitution of the substrates remains pronounced, with best results obtained combining electron rich alkenes with electron poor aldehydes and ketones.

**Scheme 14:**
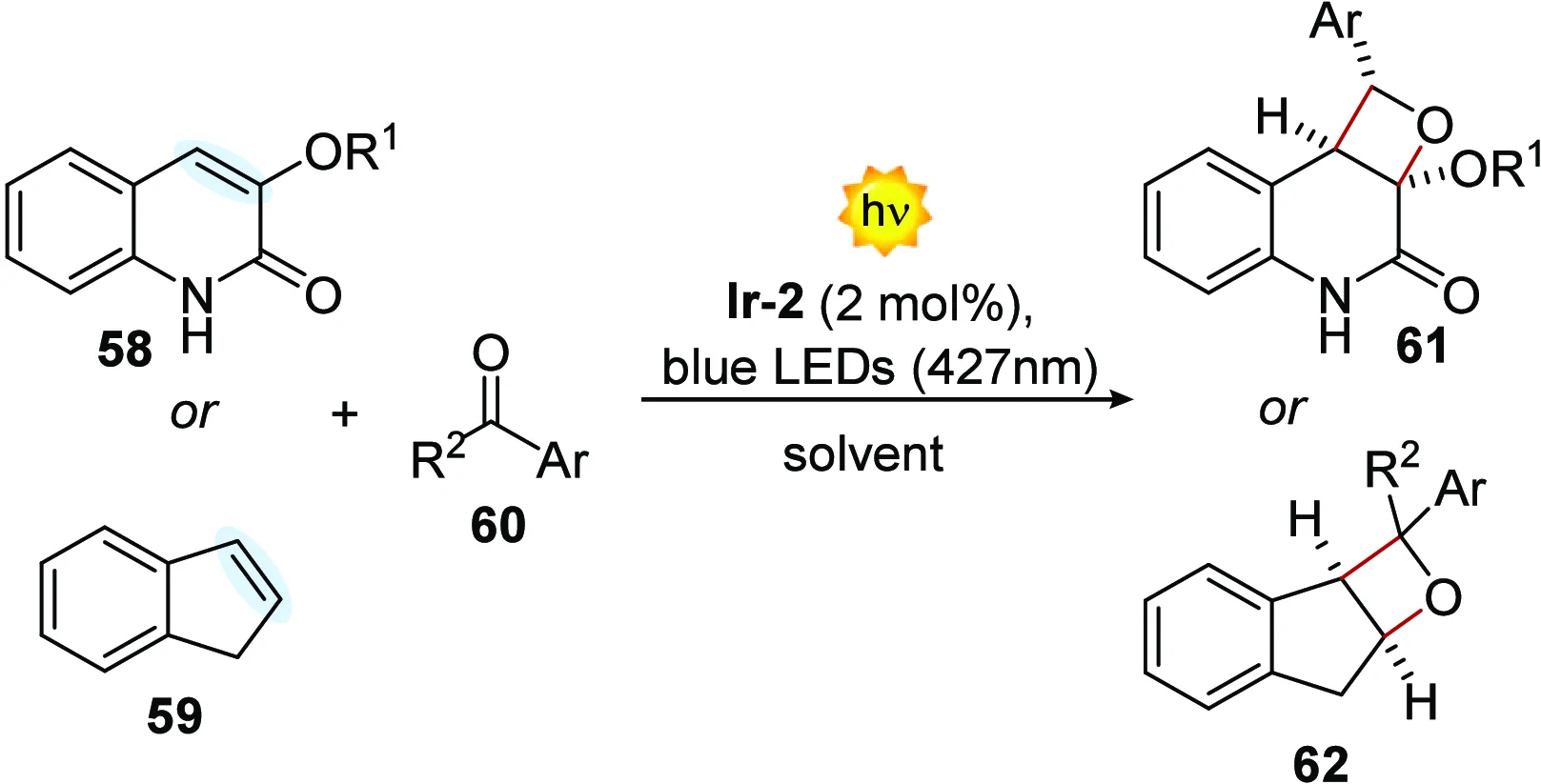
Transposed PB Reaction of 2-Quinolinones and Indenes

In 2026, Katayev and co-workers further expanded visible-light EnT-mediated PB chemistry to the synthesis of trifluoromethylated oxetanes.^117^ Using **Ir-2** under blue-light irradiation, trifluoropyruvate esters **63** were coupled with a range of alkenes **64** in dimethyl carbonate to afford CF_3_-containing oxetanes **65** and **66** (Scheme 15). The method tolerated terminal and β-substituted styrenes, cyclic alkenes, cinnamyl-alcohol-derived substrates, and selected alkynes, providing direct access to fluorinated oxetane building blocks of potential medicinal-chemistry relevance. Mechanistic experiments supported an energy-transfer manifold rather than a redox pathway: both the alkene and the CF_3_-carbonyl partner can be sensitized, but computational studies indicated that the productive cycloaddition preferentially proceeds from the triplet excited state of the trifluoropyruvate. The reaction was proposed to occur through initial C–O bond formation to give a 1,4-diradical intermediate, followed by intersystem crossing and radical recombination to furnish the oxetane products.

**Scheme 15:**
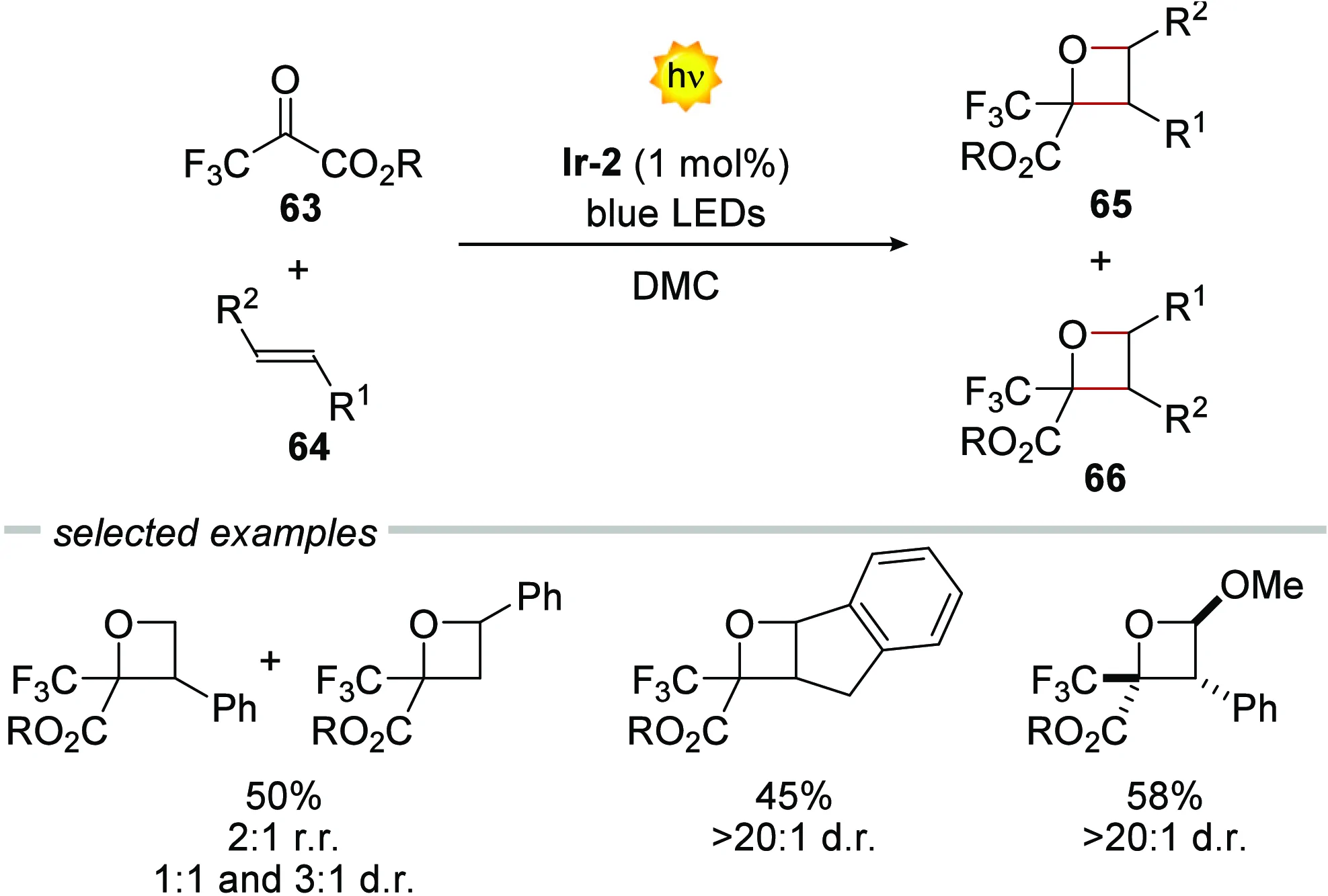
Energy-Transfer-Mediated Synthesis of Trifluoromethylated Oxetanes from Trifluoropyruvates and Alkenes

### 2.2 Oxetenes

#### 2.2.1 Direct Irradiation Methods

Carbonyl compounds **5** can also undergo PB-type hetero-[2 + 2] photocycloadditions with alkynes **67**, generating oxetenes **68** rather than oxetanes. Owing to their combined ring strain and unsaturation, these intermediates are generally short-lived and undergo rapid 4π-electrocyclic ring opening to α,β-unsaturated carbonyl compounds **69** or enter subsequent cascade reactions.^118^ Early studies established this reactivity through photochemical addition of *p*-benzoquinones to diphenylacetylene, competitive alkyne addition to the C=C and C=O bonds of *p*-quinones, and direct detection of a transient oxetene in the photoreaction of benzaldehyde with 2-butyne.^119–121^ A key antecedent was reported in 1998 by Kochi and co-workers, who showed that direct excitation of 2,6-dichlorobenzoquinone or charge-transfer excitation of its EDA complexes with diarylacetylenes converged on regioselective quinone methide formation through an oxetene-forming pathway.^122^ Xu and co-workers subsequently extended this reactivity to 1-acetylisatin and arylalkynes, obtaining alkylideneoxindoles or more elaborate dispiro products through transient spirooxetenes.^123^ Related photoreactions of 1,3,4­(2*H*)-isoquinolinetriones with diphenylacetylenes were reported in the same year and were likewise interpreted through transient oxetene formation.^124^ A later study expanded the alkyne scope and related the observed regioselectivity to the stability of the intervening triplet 1,4-diradicals (Scheme 16, selected examples **70**–**72**).^125^ Although the oxetene intermediates are typically not isolated, these transformations are included here because the carbonyl oxygen is directly incorporated into the primary hetero-cycloaddition product and the connectivity of the initial C=O/C≡C bond-forming event remains identifiable.

**Scheme 16:**
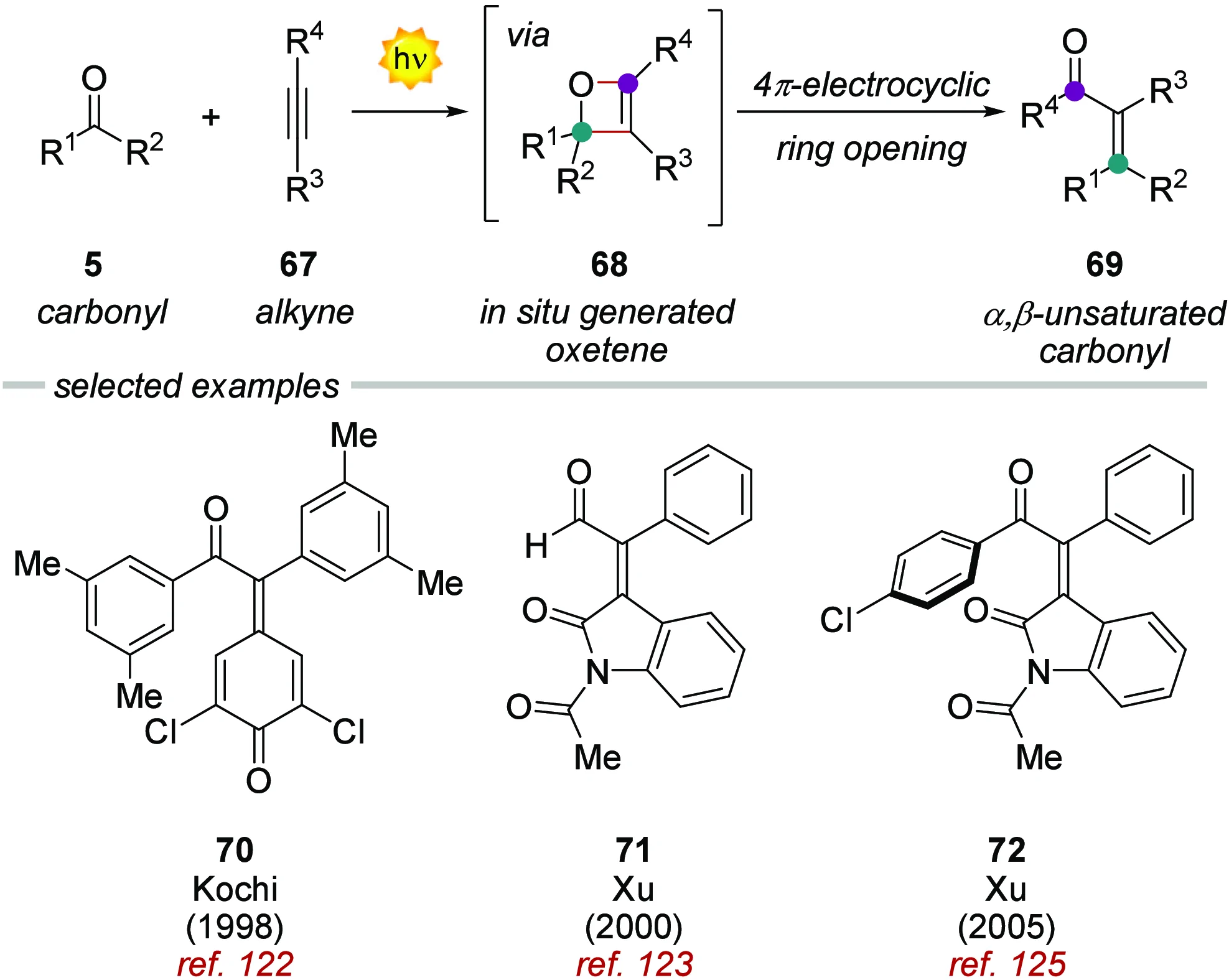
Representative Carbonyl–Alkyne Photocycloadditions Proceeding through Transient Oxetene Intermediates

The visible-light-induced photocycloaddition of *p*-benzoquinones with alkynes, followed by oxetene ring opening to *p*-quinone methides, has recently emerged as a versatile platform for alkyne difunctionalization. In 2021, Jain and co-workers exploited this sequence to generate *p*-quinone methides as synthetically useful intermediates that could be intercepted by suitable nucleophiles (Scheme 17).^126^ Irradiation at 444 nm in the presence of catalytic Zn­(OTf)_2_ enabled trapping with indole, aniline, and thiol nucleophiles, affording products **75** bearing a newly formed quaternary carbon center. On the basis of literature precedents and mechanistic experiments, the reaction was proposed to proceed through photoexcitation of *p*-benzoquinone **73** to its singlet excited state **73-S**_**1**_. Addition to alkyne **67** generates singlet 1,4-biradical intermediate **76**, which undergoes ring closure to furnish spirooxetene **77**. Subsequent 4π-electrocyclic ring opening affords the more stable *p*-quinone methide **78**, whose coordination to Zn­(OTf)_2_ promotes 1,6-addition of nucleophile **74**.

**Scheme 17:**
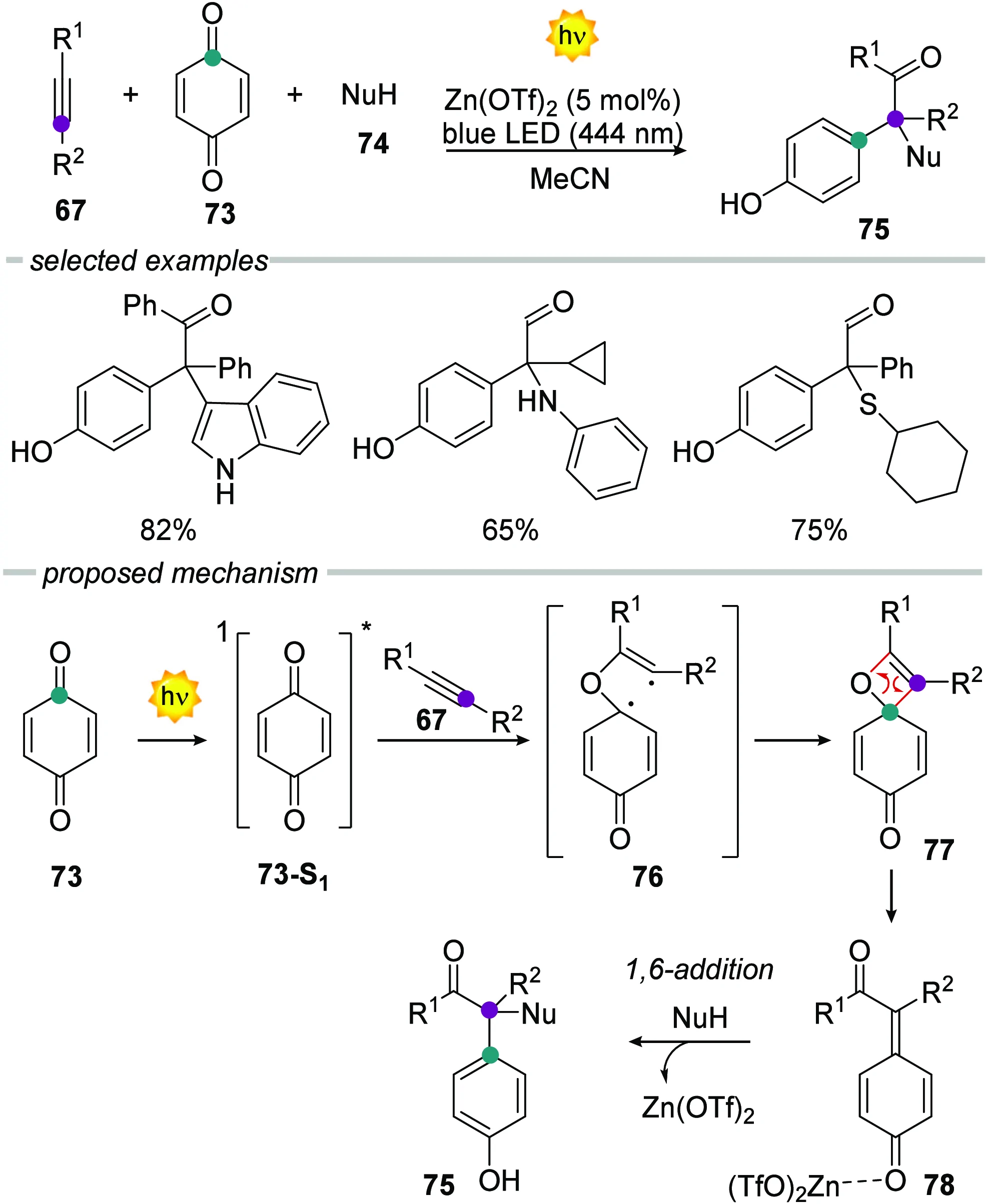
Nucleophilic Trapping of *p*-Quinone Methides Generated by Visible-Light Benzoquinone–Alkyne Photocycloaddition

Following this initial report, the scope of nucleophilic trapping progressively expanded. Rizvi, Shah and co-workers extended the process to alcohol nucleophiles using CuCN to promote trapping of the photogenerated *p*-quinone methide.^127^ Hwang and co-workers subsequently developed a broader CuCl-catalyzed protocol involving alkyl or aryl alcohols and both terminal and internal alkynes. Although the overall transformation was described as a photoredox process, the oxetene-forming pathway depicted by the authors involves direct photoexcitation of the benzoquinone, followed by addition to the alkyne, spirooxetene formation, and ring opening to the corresponding *p*-quinone methide; CuCl subsequently coordinates to this intermediate and promotes nucleophilic alcohol addition.^128^ Jain and co-workers also employed *ortho*-phenylenediamines as dinucleophiles under basic conditions, enabling the synthesis of 2,2-disubstituted dihydroquinoxalines.^129^

More recently, Yamaguchi, Itoh and co-workers developed a metal-free protocol applicable to both terminal and internal alkynes, allowing minimally substituted *p*-quinone methides **73** to be intercepted directly by alcohol nucleophiles and providing quaternary-carbon products such as **80**.^130^ Beyond the expansion of the nucleophile scope, combination of this oxetene-forming photocycloaddition with chiral Brønsted acid catalysis enabled asymmetric trapping of the in situ generated *p*-quinone methides **79**. In 2022, Lu and co-workers and, independently, Gao, Zhang and co-workers employed SPINOL-derived chiral phosphoric acids to control the addition of indole derivatives, furnishing enantioenriched products bearing α-quaternary stereocenters.^131,132^ The latter protocol provided acyclic all-carbon quaternary stereocenters, as illustrated by product **81**. In 2023, Dai, Lu and co-workers further diversified this platform through enantioselective transfer hydrogenation with Hantzsch esters, providing highly enantioenriched α,α-diaryl ketones such as **82**.^133^ Lu and co-workers subsequently developed a diastereo- and atroposelective oxo-diarylation with *N*-arylpyrroles, simultaneously establishing central and C–N axial chirality in products such as **83**.^134^ A related alkyne–carbonyl metathesis/transfer-hydrogenation sequence was independently disclosed by Gao, Zhang and co-workers.^135^ Dai, Lu and co-workers later extended this strategy to terminal alkynes, enabling the catalytic asymmetric preparation of α,α-diaryl aldehydes such as **84**.^136^ Beyond these intermolecular and asymmetric variants, intramolecular trapping of the same photogenerated **79** also enabled access to strained heterocycles such as **85**, as discussed below. Taken together, direct nucleophilic trapping products such as **80**, asymmetric intermolecular functionalization products **81**–**84**, and intramolecular cyclization products such as **85** illustrate the broad synthetic diversification accessible from a common photogenerated *p*-quinone methide intermediate **80** (Scheme 18).

**Scheme 18:**
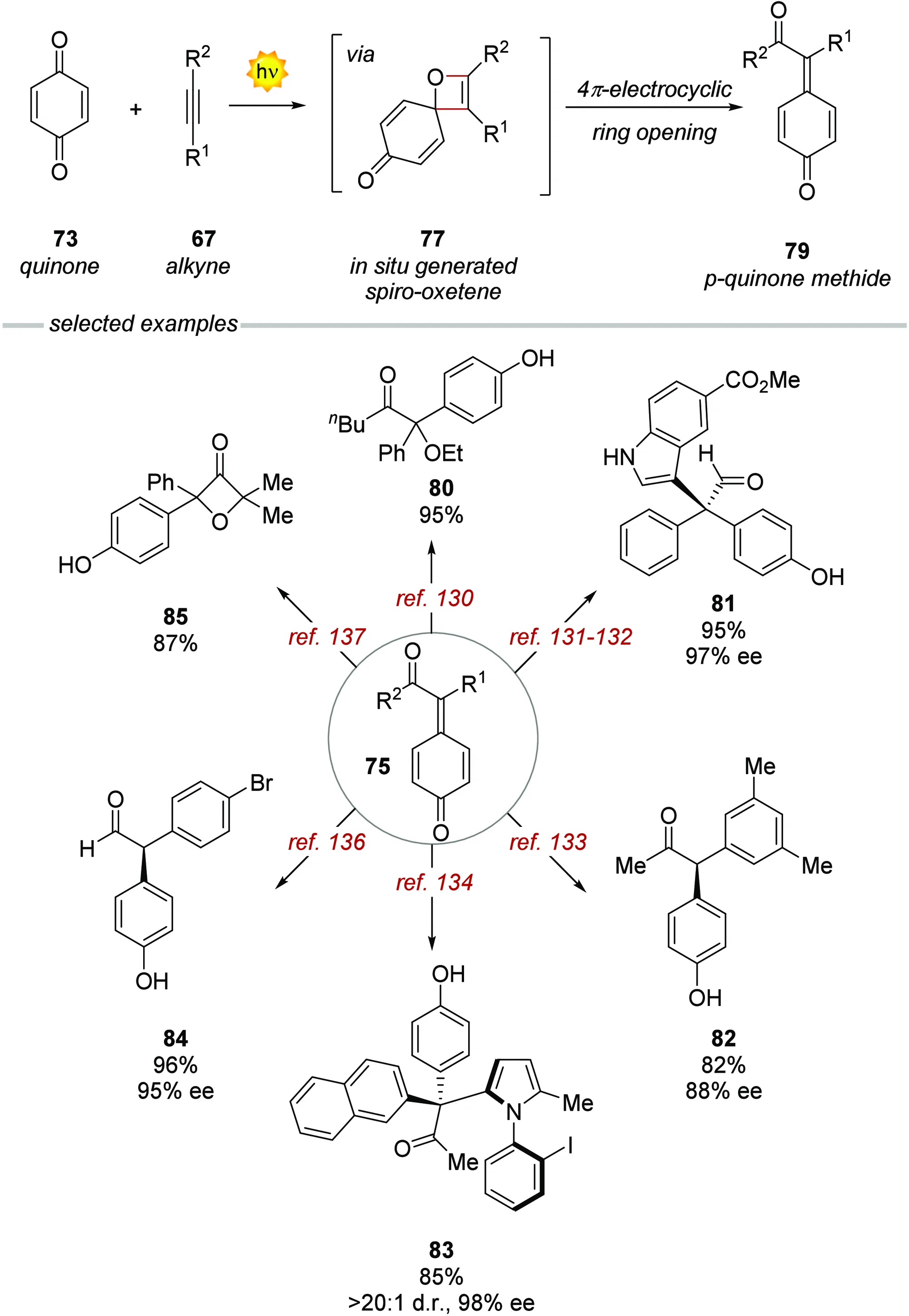
Synthetic Diversification of Photogenerated *p*-Quinone Methides from Oxetene-Forming Benzoquinone–alkyne Photocycloaddition

Among these downstream applications, intramolecular trapping of photogenerated *p*-quinone methides represents a distinct subclass that merits separate consideration. In 2024, Kim and co-workers employed propargylic alcohols and amines, for which visible-light photocycloaddition with *p*-benzoquinones, oxetene ring opening, and *p*-quinone methide formation were followed by a rearomatization-driven 4-*exo*-trig cyclization, affording oxetan-3-ones and azetidin-3-ones, such as **85**.^137^ Reddy and co-workers independently developed a catalyst- and reagent-free annulative process involving propargylic alcohols and amines, providing oxetane and azetidine derivatives through intramolecular trapping of the photogenerated *p*-quinone methide.^138^ More recently, Guan, Huang, He and co-workers extended this strategy to alkynylanilides, in which intramolecular attack of the amide oxygen on the photogenerated *p*-quinone methide furnished acylated 4*H*-3,1-benzoxazine derivatives (Scheme 19).^139^

**Scheme 19:**
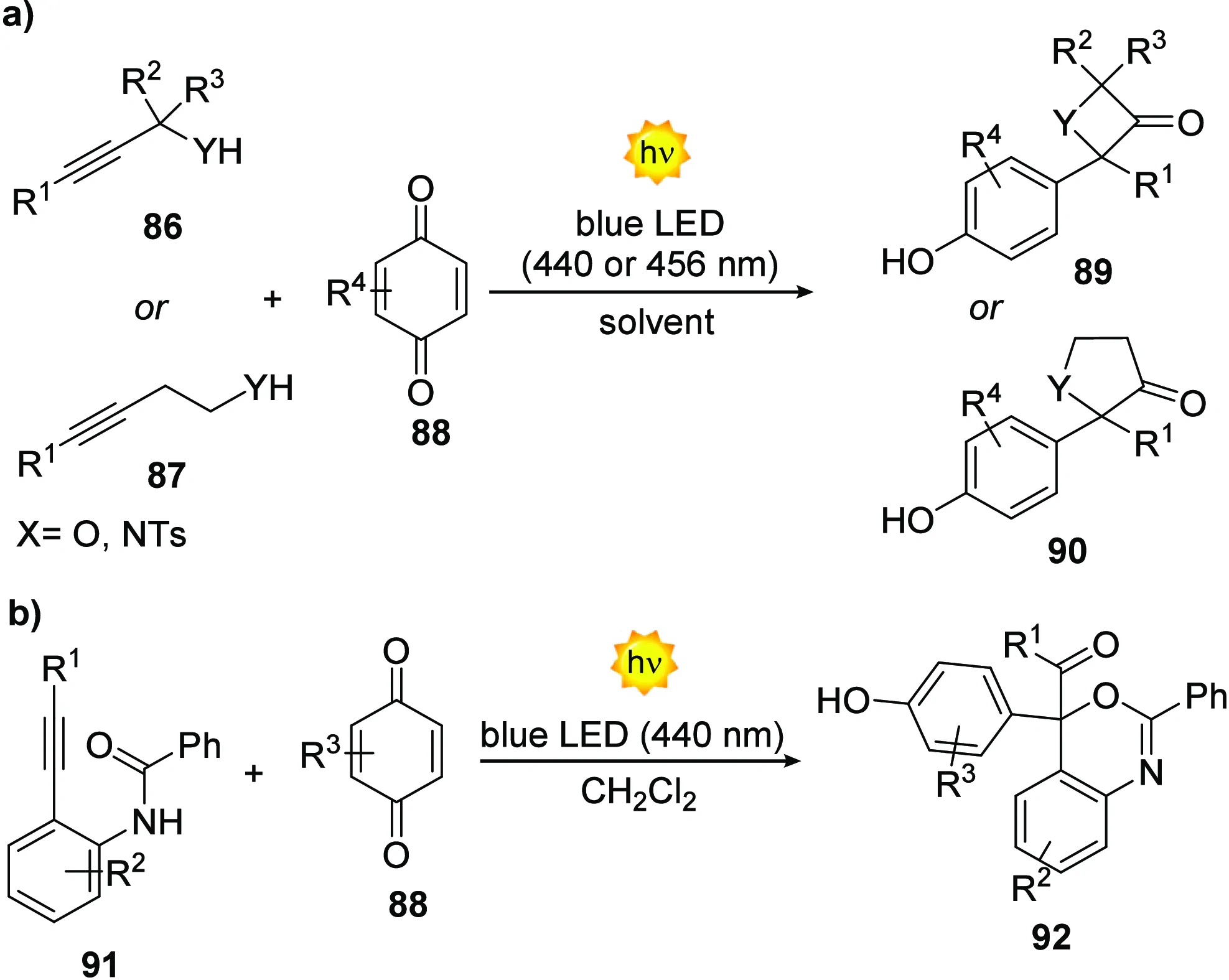
Intramolecular Trapping of *p*-Quinone Methides Generated through Benzoquinone–Alkyne PB Chemistry

Importantly, in these intramolecular variants, the isolated heterocycle is formed during the downstream trapping of the *p*-quinone methide rather than directly during the primary hetero-[2 + 2] photocycloaddition. Nevertheless, the initial light-induced step remains fully consistent with the scope of this Review, because it proceeds through an oxetene intermediate in which the carbonyl oxygen participates directly in the formation of the four-membered ring. Overall, these studies establish oxetene ring opening as an effective gateway from simple alkynes to structurally and stereochemically diverse products.

#### 2.2.2 Photocatalytic Methods

In contrast to the direct excitation processes discussed above, oxetene-forming photocycloadditions can also be embedded within photoredox cascades initiated by excitation of a catalytic species. In these cases, single-electron transfer generates reactive metal-bound or radical intermediates that subsequently engage a carbonyl component in the oxetene-forming step.

An alternative photocatalytic oxetene-forming manifold was reported in 2017 by Hwang and co-workers.^140^ Under visible-light irradiation in the presence of O_2_ and catalytic CuCl, phenols **93** underwent oxidative coupling with terminal alkynes **94** to afford aryl ketones **95** (Scheme 20). Unlike the directly excited benzoquinone–alkyne processes described above, this transformation was proposed to be initiated by excitation of an in situ generated Cu­(I) acetylide **96**, followed by single-electron transfer to O_2_ to furnish Cu­(II) species **97**. Concurrent Cu-mediated aerobic oxidation of phenol **93** generates benzoquinone **73**, which reacts within the copper-acetylide manifold to form the proposed Cu­(II)-bound spirooxetene **98**. Subsequent ring opening affords coordinated quinone methide **99**, whose fragmentation generates Cu­(I) and acyl radical intermediate **100**. Trapping by molecular oxygen, followed by rearrangement with CO_2_ extrusion and tautomerization, ultimately delivers aryl ketone **95**.^141^ Although the isolated product arises from a multistep oxidative cascade, the transformation is relevant to the present discussion because the proposed photocycloaddition generates a transient oxetene in which the carbonyl oxygen directly participates in formation of the four-membered ring.

**Scheme 20:**
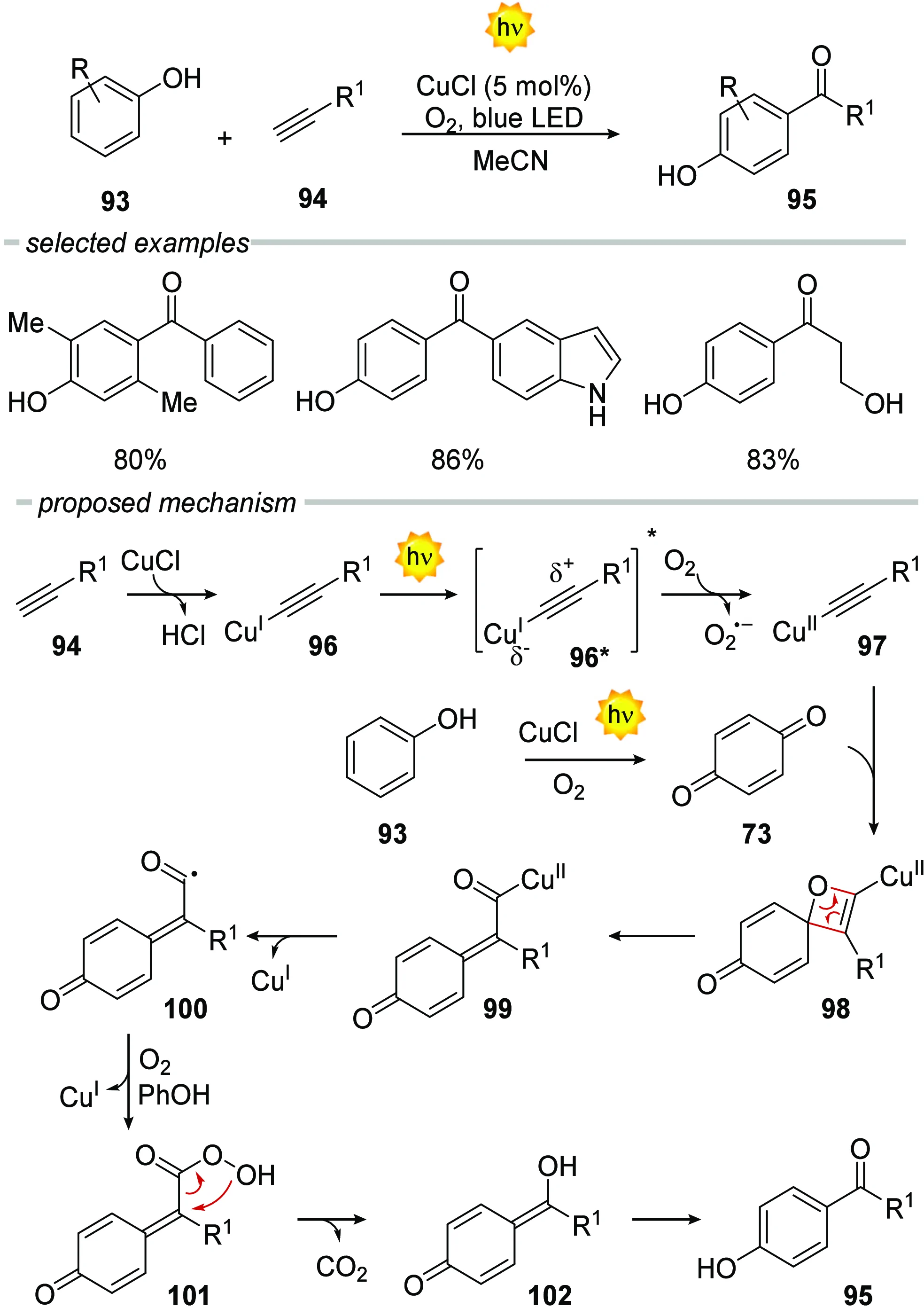
Copper-Photoredox Oxidative Coupling of Phenols and Alkynes through a Proposed Spirooxetene Intermediate

### 2.3 Dioxetanes

#### 2.3.1 Photocatalytic Methods

Dioxetanes represent a distinct class of peroxide-containing four-membered heterocycles that can arise from the hetero-[2 + 2] cycloaddition of singlet oxygen (^1^O_2_) with alkenes.^142–145^ In photochemical settings, ^1^O_2_ is typically generated by photosensitized energy transfer from an excited photosensitizer (PS) to ground-state triplet oxygen (^3^O_2_).^146–148^ The resulting ^1^O_2_ can add across electron-rich C=C bonds to form 1,2-dioxetanes, in which both oxygen atoms are directly incorporated into the newly formed ring. Although many dioxetanes are highly unstable and undergo rapid fragmentation to carbonyl products, their decomposition can generate electronically excited carbonyl species and result in chemiluminescence, a property that has found applications in sensing and bioimaging. More substituted or conformationally constrained examples can be sufficiently long-lived for isolation or downstream synthetic and mechanistic applications (Scheme 21).^142,143,149^ For this reason, the present section includes transformations in which photogenerated singlet oxygen forms a dioxetane as the primary hetero-[2 + 2] cycloadduct, even when this intermediate subsequently decomposes or is exploited mainly as a transient precursor to oxygenated products.

**Scheme 21:**
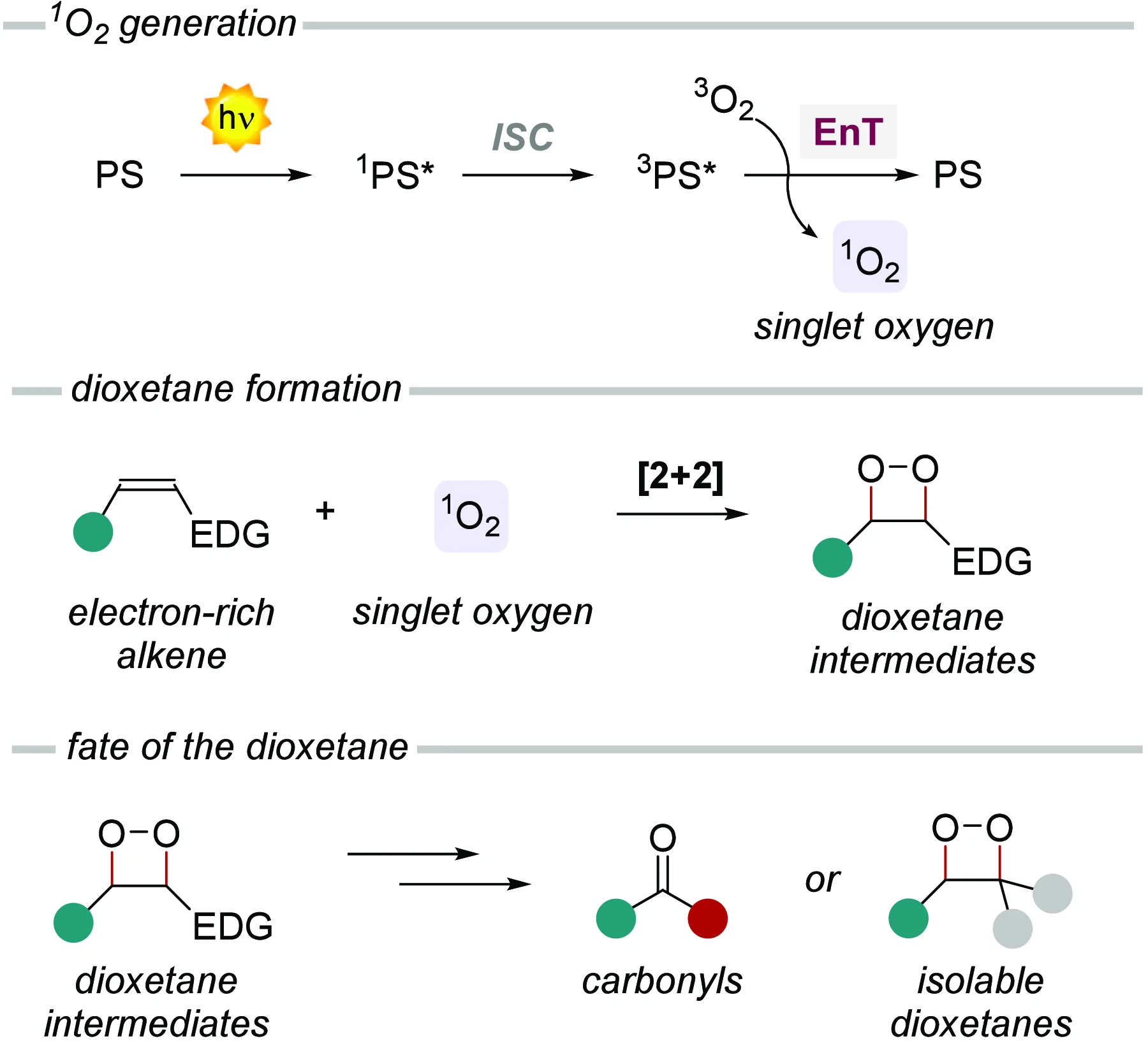
Photosensitized Generation of Singlet Oxygen and Hetero-[2 + 2] Cycloaddition to Electron-Rich Alkenes To Form Transient or Isolable 1,2-Dioxetanes, Followed in Many Cases by Fragmentation to Carbonyl Compounds (PS = Photosensitizer; EDG = Electron-Donating Group)

The competition between dioxetane formation and alternative singlet-oxygen pathways is strongly substrate-dependent. Dioxetane formation is favored for electron-rich alkenes in which allylic hydrogen abstraction is unavailable or geometrically disfavored, or when substrate organization directs ^1^O_2_ addition to a face lacking accessible allylic hydrogens. In these cases, the hetero-[2 + 2] pathway can compete effectively with other oxygenation modes, leading either to isolable dioxetanes or to short-lived intermediates whose decomposition products reveal the initial cycloaddition event.^145^ This behavior parallels other transient hetero-[2 + 2] intermediates discussed in this Review, such as oxetenes and thietanes, for which the primary cycloadduct may be synthetically relevant even when not isolated. From a synthetic perspective, singlet-oxygen dioxetane chemistry has often been used less as a general route to isolable heterocycles and more as a controlled oxidative cleavage manifold or as a mechanistic entry to high-energy peroxide intermediates. Accordingly, only representative cases are highlighted here, with emphasis on transformations in which the dioxetane connectivity is experimentally supported or synthetically exploited. Broader applications of photogenerated ^1^O_2_ in organic synthesis, including preparative oxygenations beyond dioxetane formation, have been reviewed extensively elsewhere.^142,144,148^

Classical photooxygenation studies established the fundamental features of this manifold, including stereospecific dioxetane formation from electron-rich alkenes such as substituted enol ethers.^150^ Subsequent work showed that substrate structure can strongly redirect singlet-oxygen reactivity toward the hetero-[2 + 2] pathway: in oxazolidinone-derived enecarbamates, steric and conformational effects favored dioxetane formation over competing oxygenation modes, making this family a useful platform for probing chemoselectivity and stereoselectivity in singlet-oxygen additions.^151,152^ Related studies on heteroatom-substituted cyclic alkenes further illustrated how transient dioxetanes can undergo fragmentation or intramolecular oxygen-transfer processes, so that the isolated products may be carbonyl compounds or rearranged oxygenated structures rather than the primary peroxide cycloadduct.^153^ More complex electron-rich alkenes, including polyphenolic natural-product motifs,^154^ have also been shown to undergo ^1^O_2_ [2 + 2] cycloaddition, highlighting the relevance of dioxetane intermediates beyond simple model substrates. A preparative natural-product example is provided by the synthesis of melohenine B, in which methylene-blue-sensitized photooxygenation of indole epimers was proposed to proceed through dioxetane cleavage and aldehyde intermediates before stereospecific ring reclosure to the alkaloid framework.^155^

### 2.4 Azetidines and Azetines

The [2 + 2] photocycloaddition of imines and π-systems, known as aza-PB reaction, constitutes one of the most direct and efficient approaches toward the synthesis of four-membered nitrogenated heterocycles.^156^ Particularly, photocycloaddition of imines with alkenes and alkynes gives functionalized azetidines and azetines, that are of particular interest in medicinal chemistry for their ability to enhance physicochemical properties of drug candidates.^157^ However, despite the relevance of these *N*-heterocycles, advancements it the development of general aza-PB protocols has long lagged behind. Compared to the classical Paternò–Büchi reaction, the development of its aza-variant poses additional synthetic challenges. (Scheme 22). Above all, after visible light absorption, the triplet excited state imine preferentially undergoes *E*/*Z* isomerization of the C=N bond, a nonradiative relaxation pathway that brings it back to the ground state. In addition to that, homolysis of the σ-bond at the N atom and SET reduction constitute further deactivation pathways that have long hampered the successful implementation of [2 + 2] photocycloaddition protocols with imines.^158,159^ Indeed, for long time aza-PB reactions have remained largely underdeveloped, with the few synthetically useful methods reported relying on the use of high-energy UV light and being limited in scope to configurationally locked cyclic imines, such as azauracils and isoindolones.^160–165^ Similarly, the elusive [2 + 2] cycloaddition of azo compounds with alkenes to afford 1,2-diazetidines has been documented only in constrained polycyclic systems.^166–168^ Formation of 1,2-diazetidines via [2 + 2] cycloaddition has otherwise only been invoked in the photochemical dimerization of imines and in the oxidative coupling of hydrazines with styrenes.^169,170^

**Scheme 22:**
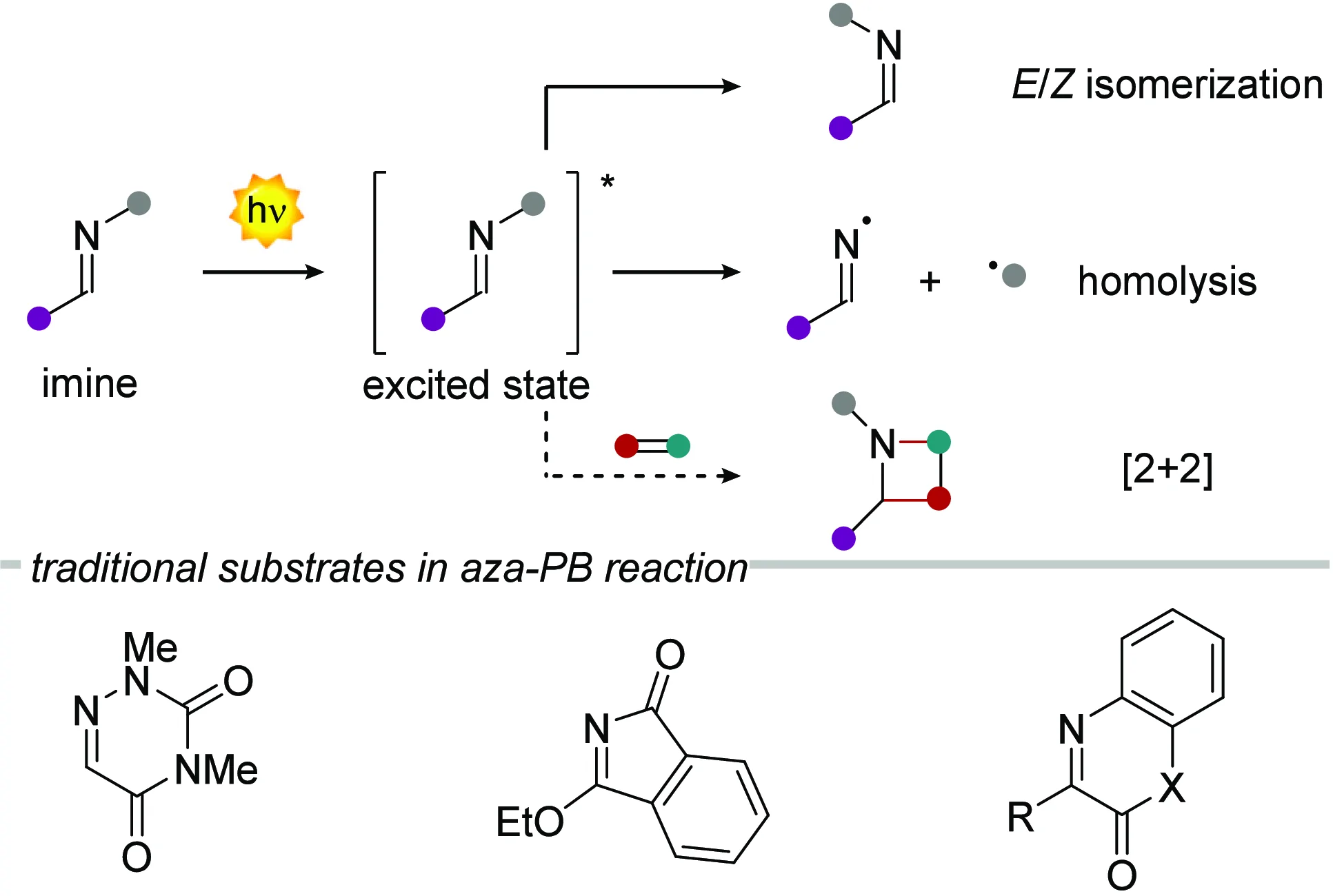
Synthetic Challenges to the Development of Aza-PB Reactions

Not surprisingly, the resurgence of synthetic photochemistry has fostered new discoveries also in this research area and the past ten years have seen the flourishing of methods toward more general aza-PB reactions.

#### 2.4.1 Direct Irradiation Methods

A milestone in the field was marked in 2016 by Maruoka and co-workers, who reported on the use of *N*-aryl sulfonyl imines to access complex azetidine scaffolds under 365 nm irradiation.^171^ Under these conditions, *N*-tosylimine of 2-naphthaldehyde **103** and *N*-(2-naphthylsulfonyl)­imine of *p*-anisaldehyde reacted with substituted benzofurans **104** and styrenes **105** (Scheme 23). A limitation of this approach was the need to use a high-pressure Hg lamp for substrates whose absorption profiles were shifted toward the deep-UV region. Nevertheless, this work represents the first example of acyclic imines participating in [2 + 2] photocycloaddition to alkenes. From a mechanistic point of view, the authors proposed a concerted pathway involving singlet exciplex with a π-π interaction between the aryl moieties of the imine and the olefin. The formation of a singlet exciplex intermediate is supported by the stereospecificity observed in the addition to 1,2-substituted styrenes.

**Scheme 23:**
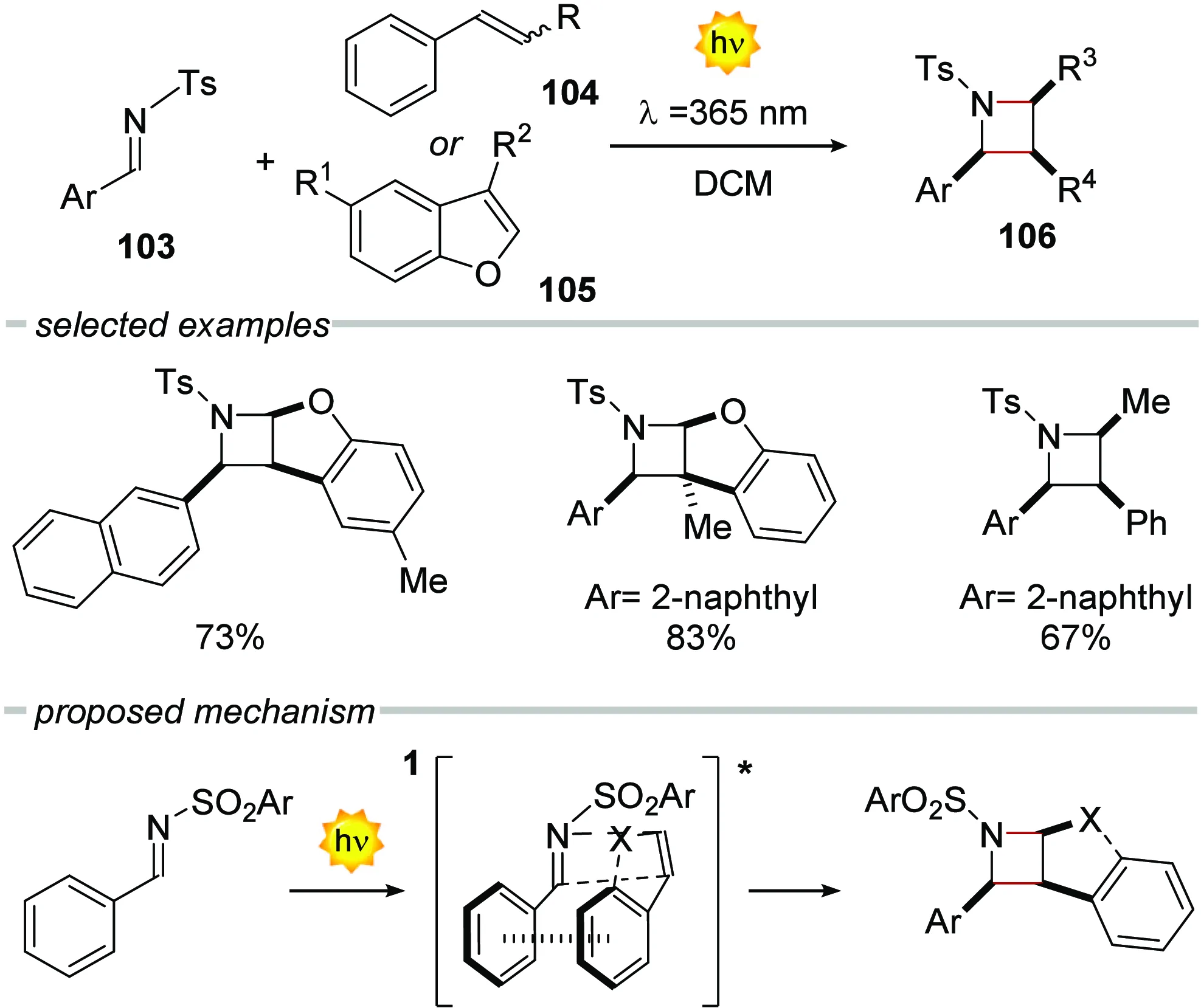
Intramolecular Transposed Paternò–Büchi Reaction of Atropoisomeric Enamides

From a mechanistic point of view, the authors proposed a concerted pathway involving a singlet exciplex featuring a π–π interaction between the aryl moieties of the imine and the olefin. The stereospecificity observed in the addition to 1,2-disubstituted styrenes supports the involvement of this intermediate and highlights the importance of π–π interactions in promoting the cycloaddition.

Capitalizing upon their Cu-catalyzed PB reaction,^103^ the Schmidt group reported an aza-PB variant in 2022.^172^ Irradiation of acyclic imines **107** in the presence of norbornene **108** using a Hg lamp with catalytic TpCu resulted in the formation of azetidines **110** (Scheme 24). Importantly, acyclic *N*-alkyl aldimines and ketimines, and oximes were all competent substrates. Mechanistic studies confirmed that the reaction is driven by the photoexcitation via MLCT of the *in situ* formed Cu-alkene complex. As an intrinsic limitation of the method, beyond norbornenes **108**, the reaction is applicable only to specific cyclic alkenes, such as maleic anhydride and maleimide **109**.

**Scheme 24:**
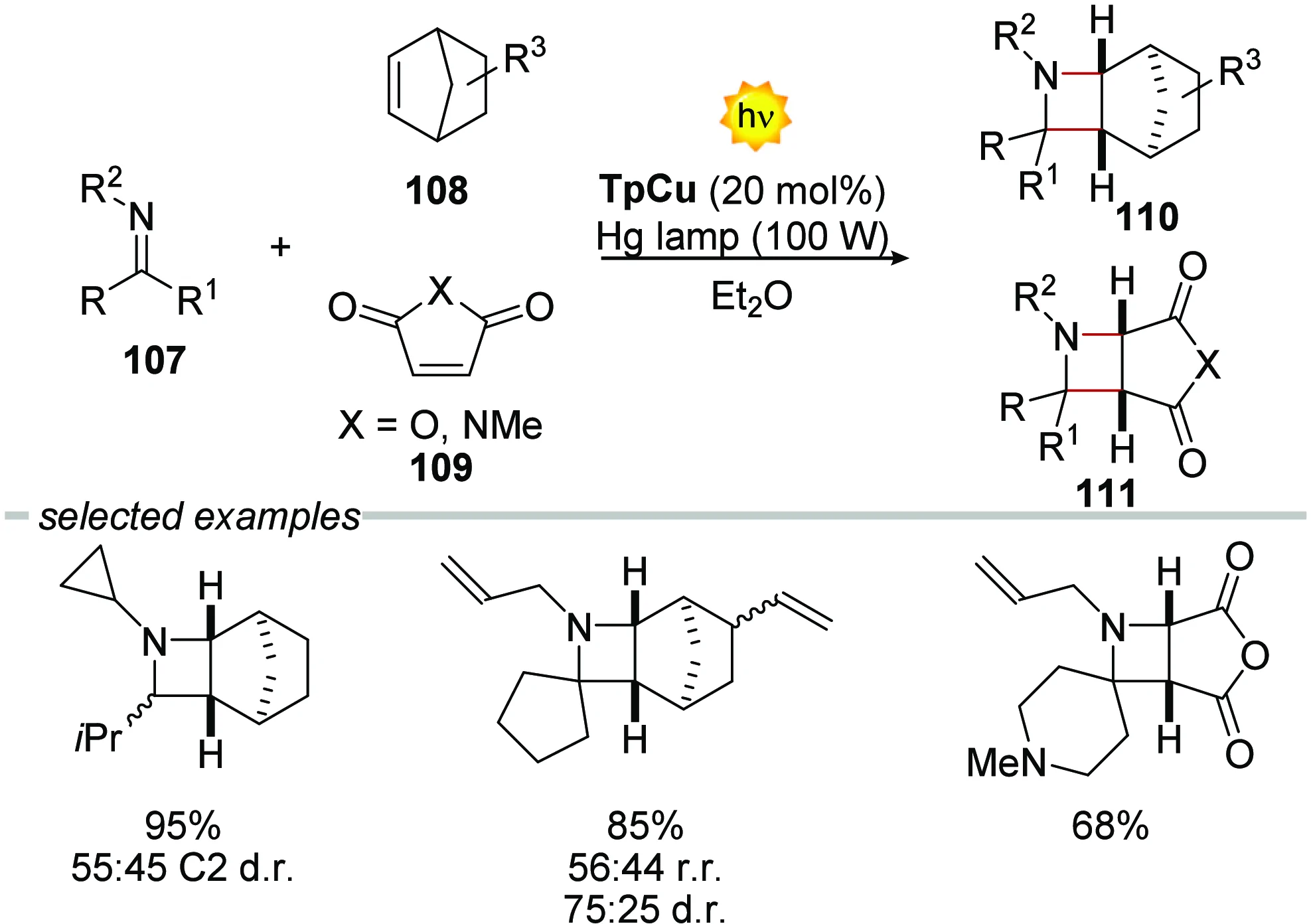
Intermolecular Aza-PB Reaction Enabled by Cu-Alkene MLCT

#### 2.4.2 Photocatalytic Methods

Following a mechanistically distinct approach, the group of Park reported a photoredox protocol to the intramolecular cycloaddition of imines and alkynes. Cyclization of the acyclic imine to the tethered alkyne within **112** was achieved under blue light irradiation using a pyrylium photoredox catalyst **Pyr-1**, a process that ultimately led to the formation of pyrrolizidinone **113** (Scheme 25).^173^ Upon light absorption, the excited state **Pyr-1** behaves a strong oxidant and engages with the alkyne moiety of **112** in a SET oxidation to give the radical cation **114**, as supported by Stern–Volmer quenching studies and cyclic voltammetry analyses. Then, intramolecular nucleophilic addition of the imine gives the dystonic radical cation **115**, that further rearranges to **116**. A SET reduction from the reduced **Pyr-1** gives the unstable 2-azetine **117** that undergoes ring opening to **118**. This intermediate reacts via a further SET oxidation to a second molecule of excited **Pyr-1** giving radical cation **119**. Finally, after two consecutive rearrangements, radical cation **120** reacts in a SET reduction from the reduced form of **Pyr-1** to give pyrrolizidinone **114**. This work represents a rare example on the use of photoredox catalysis within the [2 + 2] photocycloaddition landscape, which commonly relies either on direct irradiation strategies or on EnT photocatalysis.

**Scheme 25:**
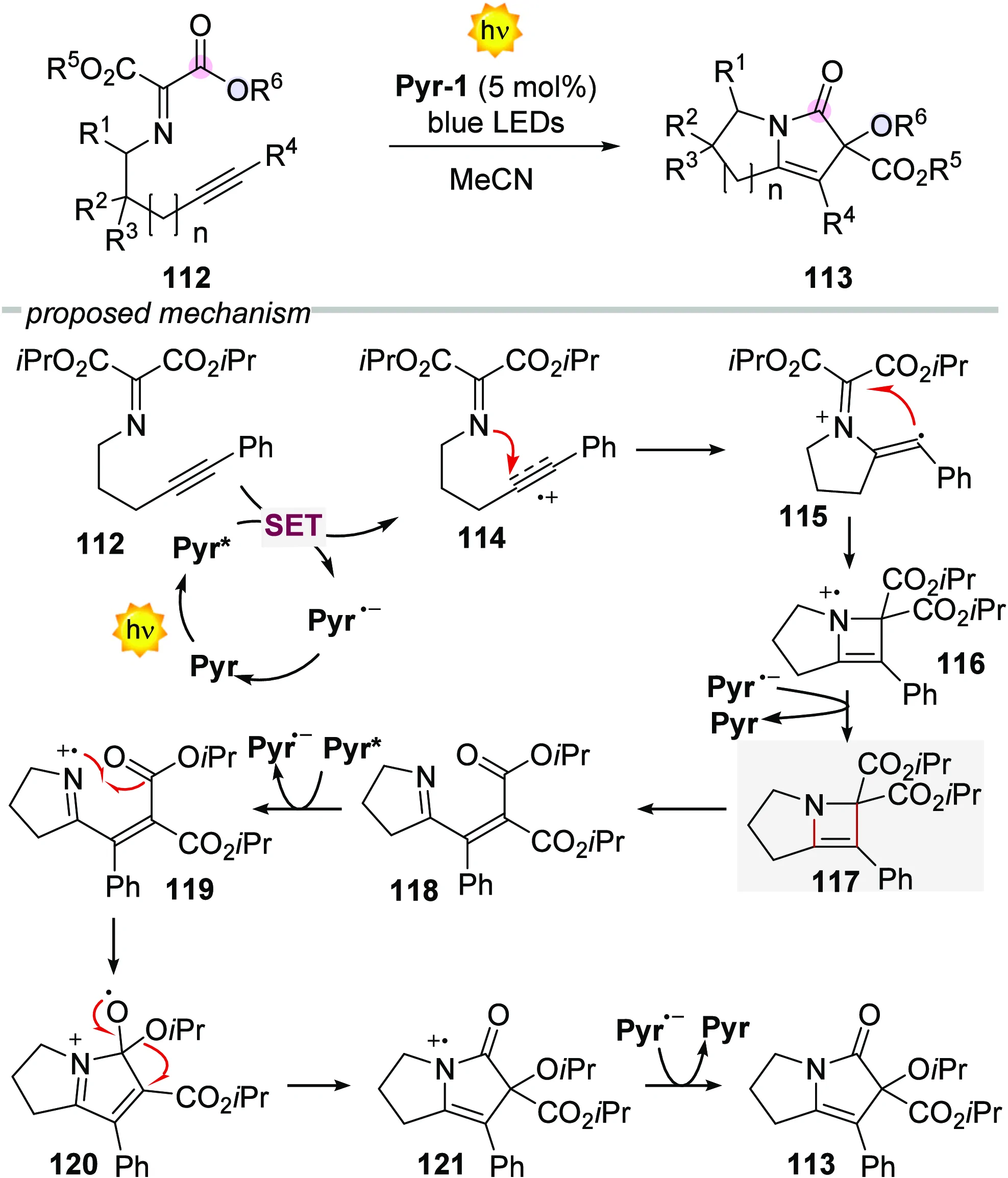
Intramolecular Photoredox Catalyzed Aza-PB Reaction of Imines and Alkynes

In analogy with their previous work,^102^ in 2017 the Sivaguru group reported the transposed aza-Paternò–Büchi reaction. Here atropisomeric enamides **122** bearing tethered acyclic imines in the presence of xanthone **XO** as photosensitizer at 350 nm irradiation underwent intramolecular [2 + 2] photocycloaddition to afford polycyclic azetidines **123** (Scheme 26).^174^ Detailed mechanistic investigations revealed that the process involves the selective excitation of the alkene chromophore rather than the imine, thereby circumventing the undesired relaxation pathways typically associated with excitation of acyclic imines. Beyond demonstrating highly efficient axial-to-point chirality transfer, this report established EnT photocatalysis as an enabling tool to go beyond substrate direct excitation strategies, paving the way to more general and operationally simple aza-PB processes.

**Scheme 26:**
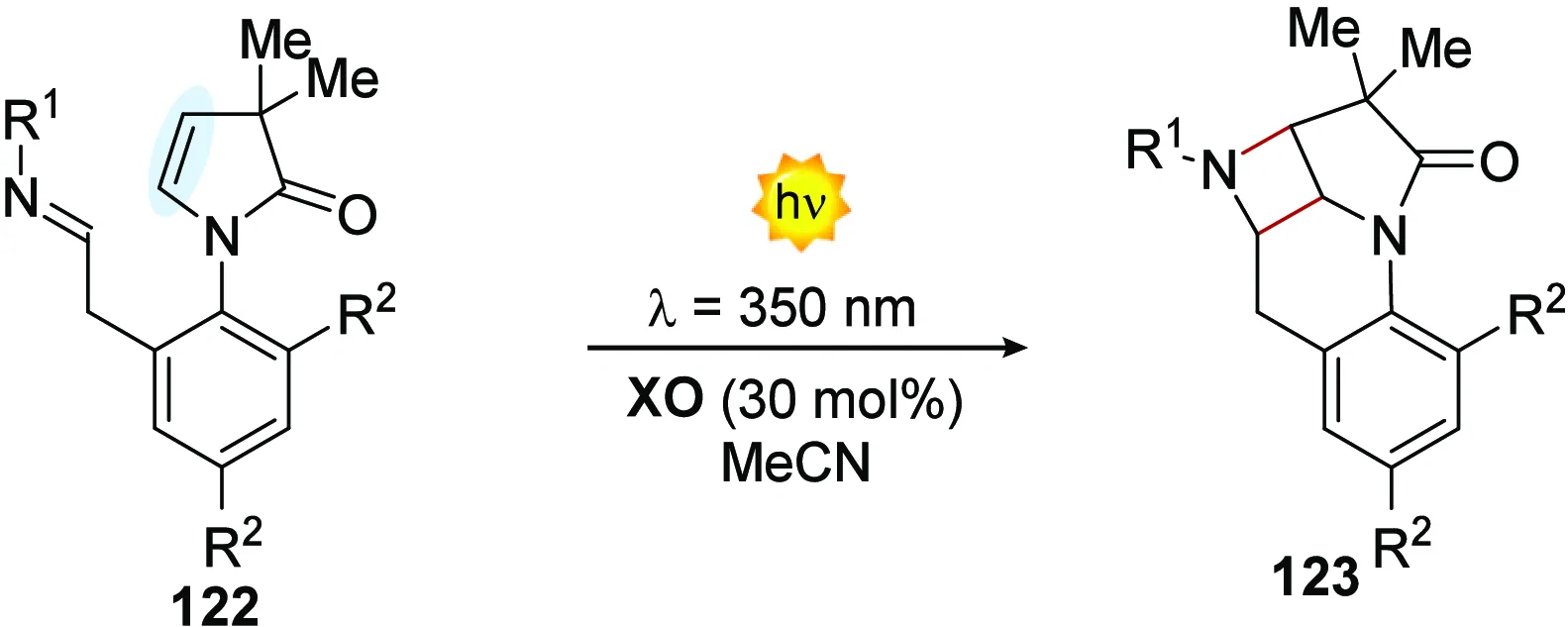
Intramolecular Transposed Aza-Paternò–Büchi Reaction of Atropoisomeric Enamides

A similar intramolecular approach was reported in 2019 by Schindler et al., who accomplished one of the first examples of aza-PB reactions promoted by visible-light irradiation (Scheme 27).^175^ Here, photocyclization of the oxime substrate **124** was achieved using an Ir photocatalyst as sensitizer under blue light irradiation (427 nm). The reaction design was based on the reason that the low-lying triplet state of the styrene makes it easier to be populated over the one of the oxime moiety. Therefore, the judicious selection of **Ir-2** photocatalyst with proper E_T_ value enables the selective sensitization of substrate **124** to give triplet diradical **124-T**_**1**_. This can undergo a reversible C–C bond formation, leading to intermediate **126-T**_**1**_. Finally, intersystem crossing (ISC) followed by a C–N bond-forming step delivers the desired azetidine product **125**. This mechanistic proposal was supported by Stern–Volmer quenching studies, while a photoredox process was considered thermodynamically unfavored based on the redox properties of the substrate.

**Scheme 27:**
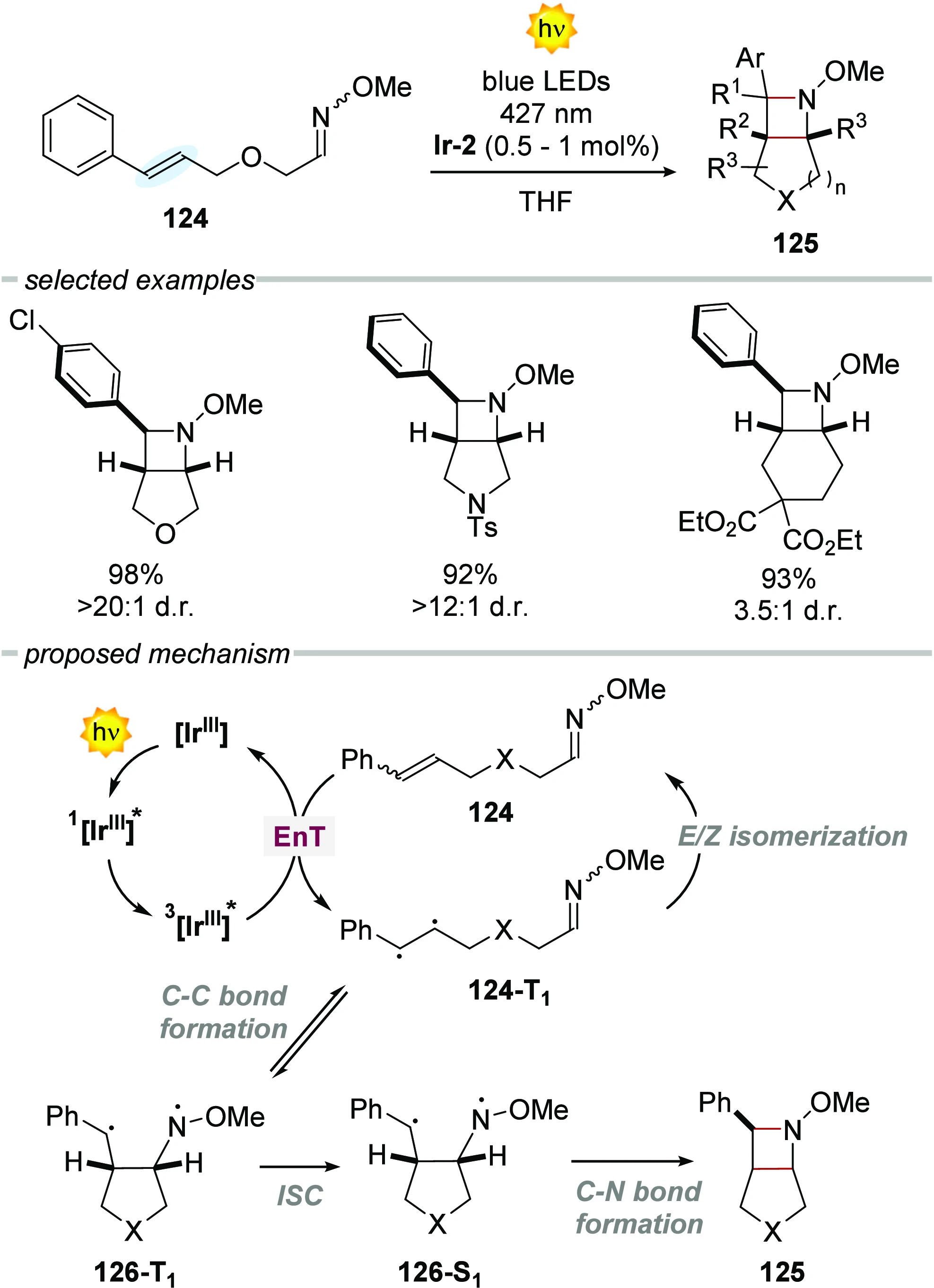
Intramolecular Transposed Aza-PB Reaction of Oximes

Following the same approach, Wei, Fu, Zhang, and co-workers reported the intramolecular photocycloaddition of isoquinolinones tethered to an oxime moiety. Here, a benzonitrile-derived organic photocatlyst is used as sensitizer as a more sustainable alternative to Ir.^176^

In 2020, You et al. reported a dearomative [2 + 2] cycloaddition of indoles **127** bearing a pendant oxime moiety (Scheme 28).^177^ Under the optimal conditions, the authors could access to an array of complex polycyclic indoline-fused azetidines **128** in moderate to excellent yields (33–98%) and diastereoselectivity (>20:1).

**Scheme 28:**
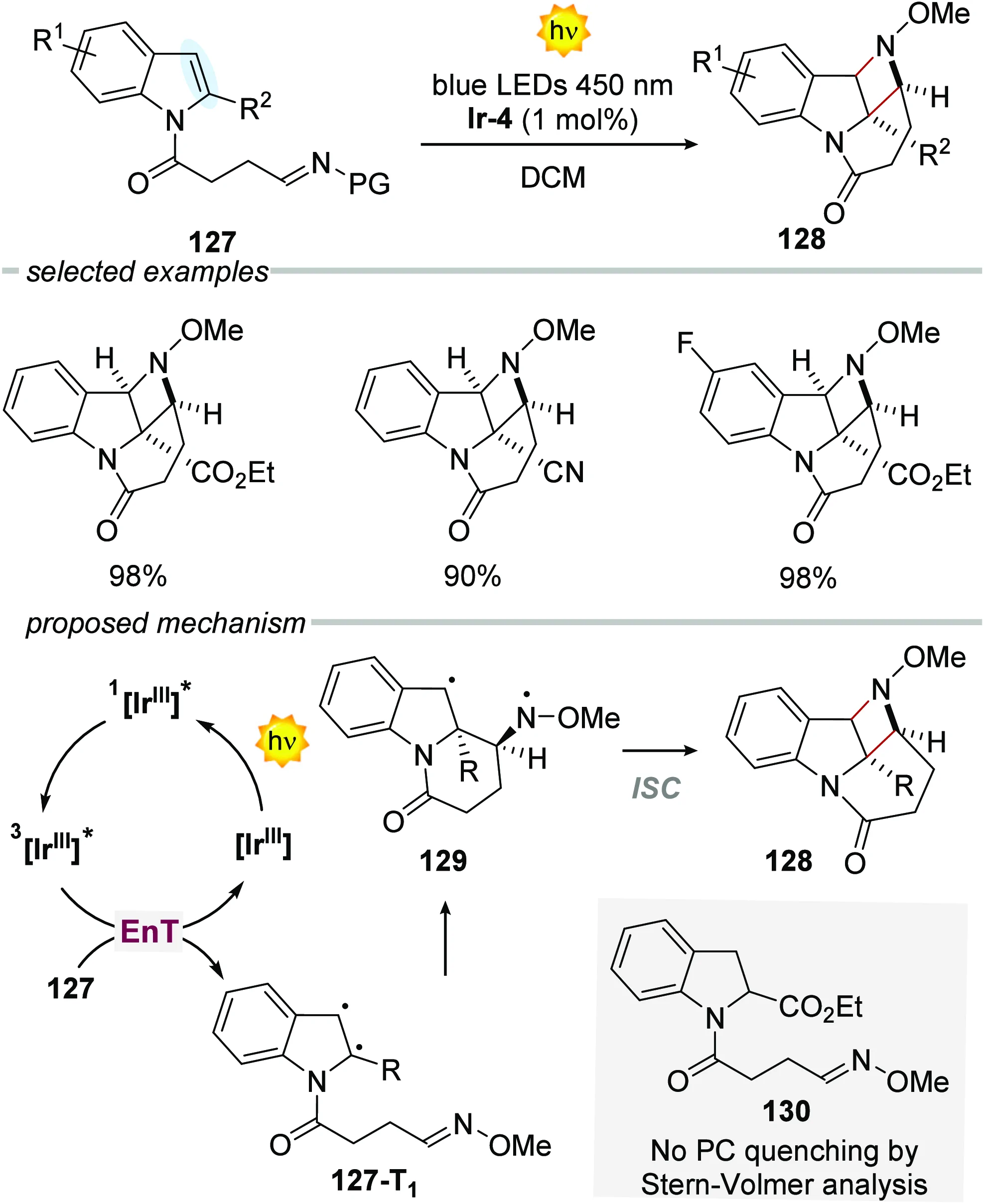
Intramolecular [2 + 2] Photocycloaddition of Indoles and Acyclic Imines

Cyclic voltammetry measurements as well as triplet quenching experiments of **127** and a related saturated indoline **130**, allowed to determine that the reaction is triggered by the excitation of the indole core, as no quenching of the excited photosensitizer was observed for **130**. Therefore, the proposed mechanism begins with an EnT event from the triplet **Ir-4** catalyst to the indole core of **127**, leading to the reactive intermediate **127-T**_**1**_. Then, radical addition to the C=N double bond of the oxime moiety leads to the triplet 1,4-biradical **129**. Finally, an ISC event followed by radical recombination delivers the indoline-fused azetidine product **128**.

A related intramolecular strategy was reported by Xu and co-workers for the preparation of polycyclic benzothiazoline derivatives.^178^

Depending on the position and substitution of the alkene within the tether, as well as the tether length, a crossed photocycloaddition can be obtained. For example, during their study on the cycloaddition of 1,5-dienes, Rigotti and Bach showed that photosensitization of oximes **131** led to an intramolecular crossed aza-Paternò–Büchi reaction to give the 5-azabicyclo[2.1.1]­hexanes **132** (Scheme 29a).^179^ Notably, these products represent pyrrolidine analogues with potential interest in medicinal chemistry.

**Scheme 29:**
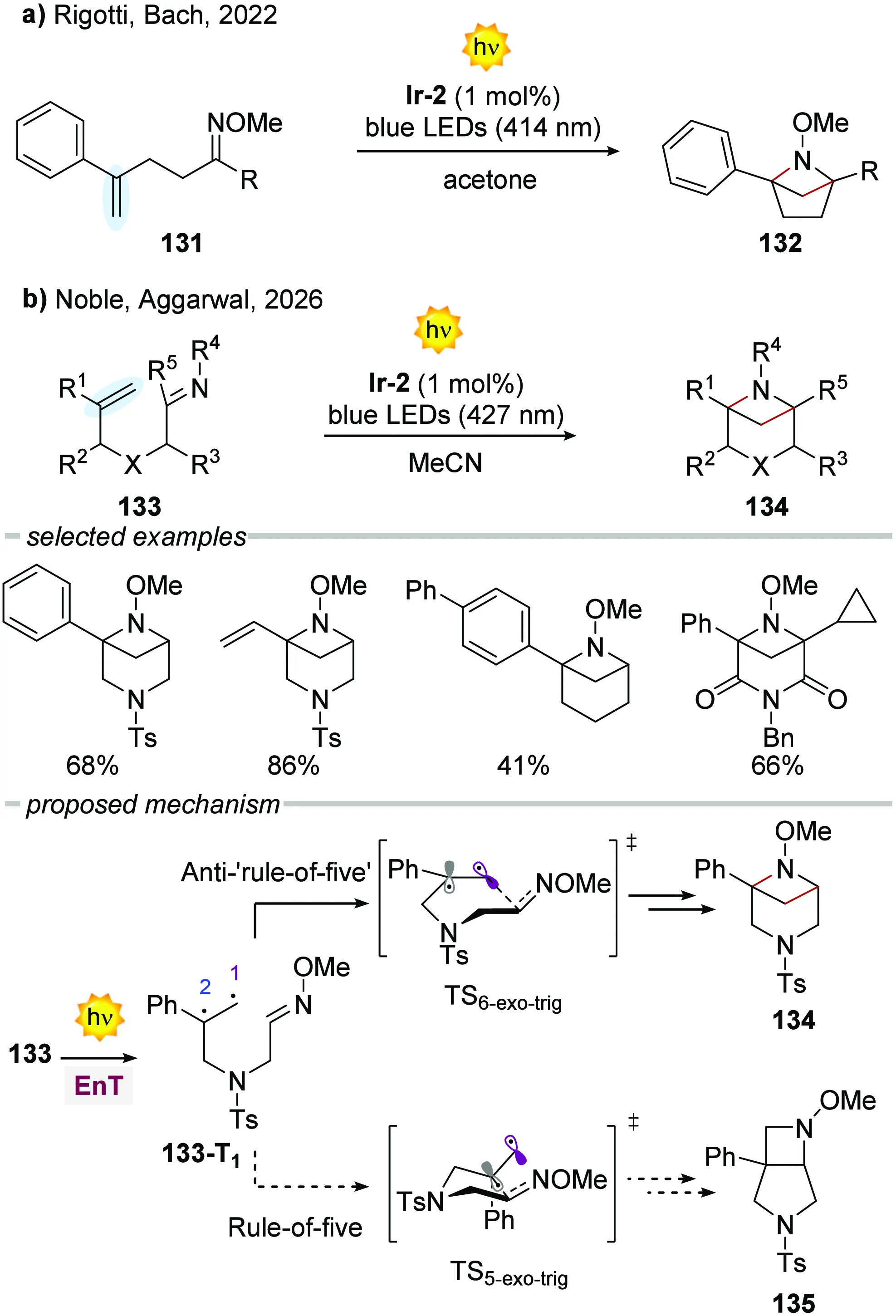
Intramolecular Crossed Aza-PB Reaction of Oximes

The related crossed photocycloaddition of aza-1,6-dienes is more challenging, as it goes against the empirical rule known as “rule-of-five”, which states that the 1,2-diradical formed upon sensitization of the substrate reacts to preferentially form a five-membered ring intermediate.^180–182^ Nevertheless, Noble, Aggarwal and co-workers demonstrated that oximes bearing a suitably substituted alkene tether **133**, upon photosensitization, can indeed react via a crossed aza-PB reaction to give 6-aza-bicyclo[3.1.1]­heptanes **134** (Scheme 29b).^183^ DFT calculations revealed a 6-*exo*-trig cyclization to be kinetically more favored than the alternative 5-*exo*-trig mode., which was tentatively explained by the presence of a stabilizing substituent at C2 that lowers the reactivity at that site. Remarkably, this study provided a general strategy to substituted aza-bicyclo[3.1.1]­heptanes, saturated compounds with well-defined exit vectors that could act as bioisosteres or a range of nitrogenated heterocycles.

In 2020, Schindler et al. reported a visible light photocatalytic intermolecular approach to the aza-PB reaction with unactivated alkenes **136** (Scheme 30).^184^ In contrast to their previous work,^175^ this [2 + 2] cycloaddition process relied on the sensitization of oxime **137** rather than the alkene. Importantly, the use of 2-isoxazoline-3-carboxylates as cyclic oximes was found to be crucial to prevent isomerization of the C–N bond as competing relaxation pathway. Blue light irradiation in the presence of **Ir-4** complex enabled to engage a variety of functionalized alkenes under mild conditions, delivering the desired azetidines **138** from moderate to high yields (46–99%), and up to >20:1 regioisomeric ratios. In contrast to previously reported aza-PB reactions involving singlet-state aryl isoxazolines, this transformation displays a different regioselectivity, which indicates the distinctive reactivity of isoxazoline carboxylates **137**. Indeed, no azetidine formation was observed upon excitation of **137** with UV light, suggesting that direct irradiation does not allow a sufficient population of the triplet state of **137**, and that the singlet **137-S**_**1**_ is probably not reactive toward [2 + 2] cycloaddition processes. Thus, based on the mechanistic investigation, the process is proposed to involve the triplet isoxazoline **137-T**_**1**_ that reacts with alkene **136** to form the biradical intermediate **139** or **139′**. Then, ISC, followed by C–N bond formation delivers azetidine **138**. The authors explained the kinetically preferred formation of the *exo*-isomer as a result of the minimized steric interactions of the alkene substituent with the isoxazolidine ring in intermediate **139**.

**Scheme 30:**
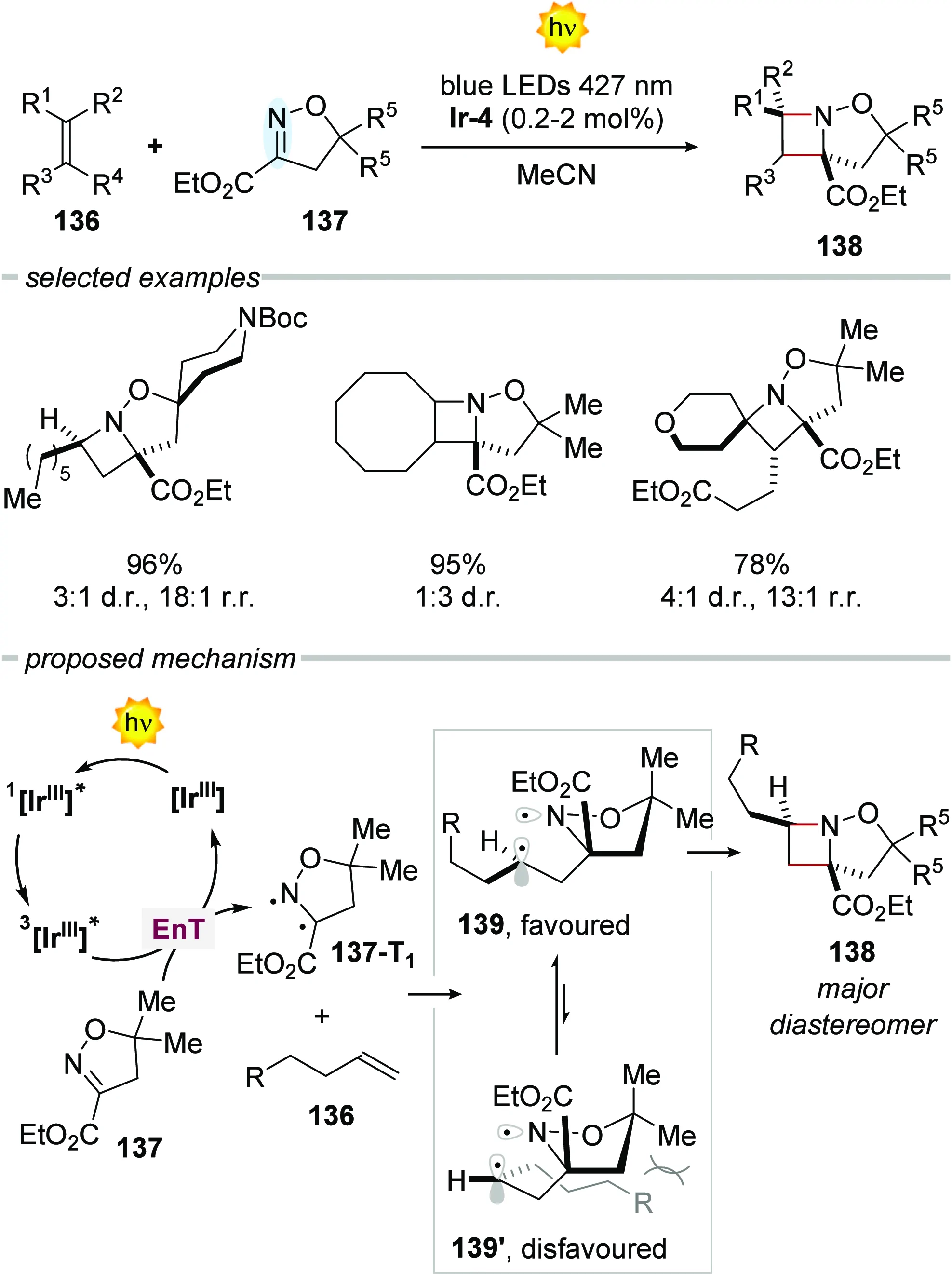
Intermolecular Aza-PB Reaction of Alkenes and Isooxazoline Carboxylates

Taking advantage of this design principle relying on oxime sensitization, Schindler et al. expanded the scope of intramolecular aza-PB reactions with isooxazoline carboxylates.^185^ Interestingly, both these inter- and intramolecular versions were applied to the preparation of azetidine-based energetic materials.^186^

Recently, the intermolecular PB reaction of alkenes and 2-isoxazoline-3-carboxylates was accomplished using a pyrrolo­[2,1-*a*]­isoquinoline derivative as an organic photoredox catalyst,^187^ while the group of Molander exploited this reactivity to prepare a DNA-encoded library of a variety of 2-oxa-1-azabicyclo[3.2.0]­heptanes.^188^

In 2021, the Schindler group further expanded the triplet state reactivity of 2-isoxazolines to the intermolecular aza-PB reactions with alkynes (Scheme 31).^189^

**Scheme 31:**
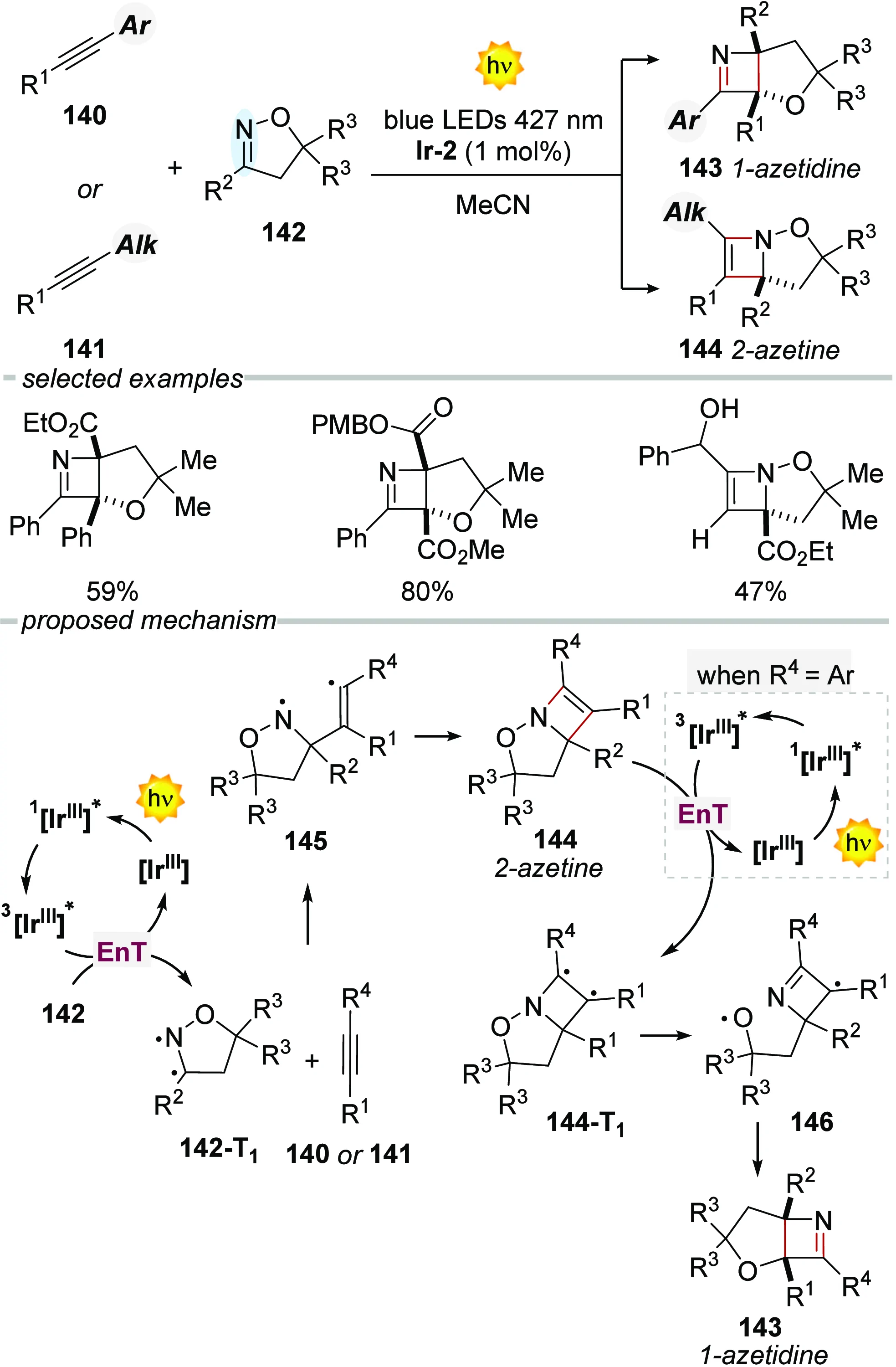
Intermolecular Aza-PB Reaction of 2-Isoxazolines with Alkynes

Through this process, they reported the expeditious preparation of an array of medicinally relevant 1-azetines **143** and 2-azetines **144** under mild reaction conditions. Specifically, 1-azetidines (**143**) were obtained from aryl alkynes (**140**), whereas the use of aliphatic alkynes (**141**) resulted in the selective formation of 2-azetines (**144**).

The proposed mechanism of this transformation initiates by a triplet EnT event from the triplet **Ir-2** catalyst to isooxazoline **142**, leading to the biradical **142-T**_**1**_. Then addition to the alkyne (whether **140** or **141**) occurs with a regioselectivity that favors the formation of the most stable triplet biradical **145**. This undergoes ISC to the singlet state, followed by radical recombination to give the 2-azetine product **144**. At this stage, 2-azetines formed from activated alkynes **140** undergo a second sensitization from the triplet **Ir-2** catalyst to generate the triplet biradical **144-T**_**1**_. Then cleavage of the N–O bond leads to **146**, that after ISC and radical recombination affords the 1-azetine product **143**. On the other hand, 2-azetines originated from aliphatic alkynes **141** cannot undergo further EnT from the Ir photocatalyst, thus **144** being the final product of the reaction.

Intriguingly, when the same group tried to extend this reactivity to the intramolecular [2 + 2] photocycloaddition of 2-isoxazolines tethered to an alkyne, they observed the exclusive formation of dimeric, macrocyclic azetines.^190^ In analogy with the previous work,^189^ 1-azetine dimeric products were obtained for activated aryl alkynes, while 2-azetine-based macrocycles were formed using aliphatic alkynes. Computational studies indicate that the reaction begins with an unexpected intermolecular [2 + 2] cycloaddition. Although an intramolecular pathway is also possible, the intermolecular process is preferred under nonstandard Curtin–Hammett conditions, enabling efficient macrocyclic dimerization.

In 2021 Bach et al. reported an elegant example of catalytic enantioselective aza-PB reaction, which constitute a landmark development in the field.^191^ Specifically, quinoxalinones **147**—traditional cyclic imine substrates in aza-PB reactions—were reacted with styrene derivatives **148** using visible light and a chiral thioxanthone photosensitizer **C-THX** at low temperature to access complex chiral azetidines **149** in high yields (50–99%) and enantiomeric excess (86–98%) (Scheme 32). The design principle relied on the 2-fold ability of the chiral thioxanthone catalyst to coordinate the lactam moiety within **147** through hydrogen-bond interactions, shielding the Si prochiral face of the quinoxalinone system, and to act as triplet sensitizer populating the imine triplet state. Thereby, addition to styrene **148** occurs from the Re face, leading to the biradical intermediate **150** that, after ISC, undergoes diastereoselective radical coupling to give azetidine **149**.

**Scheme 32:**
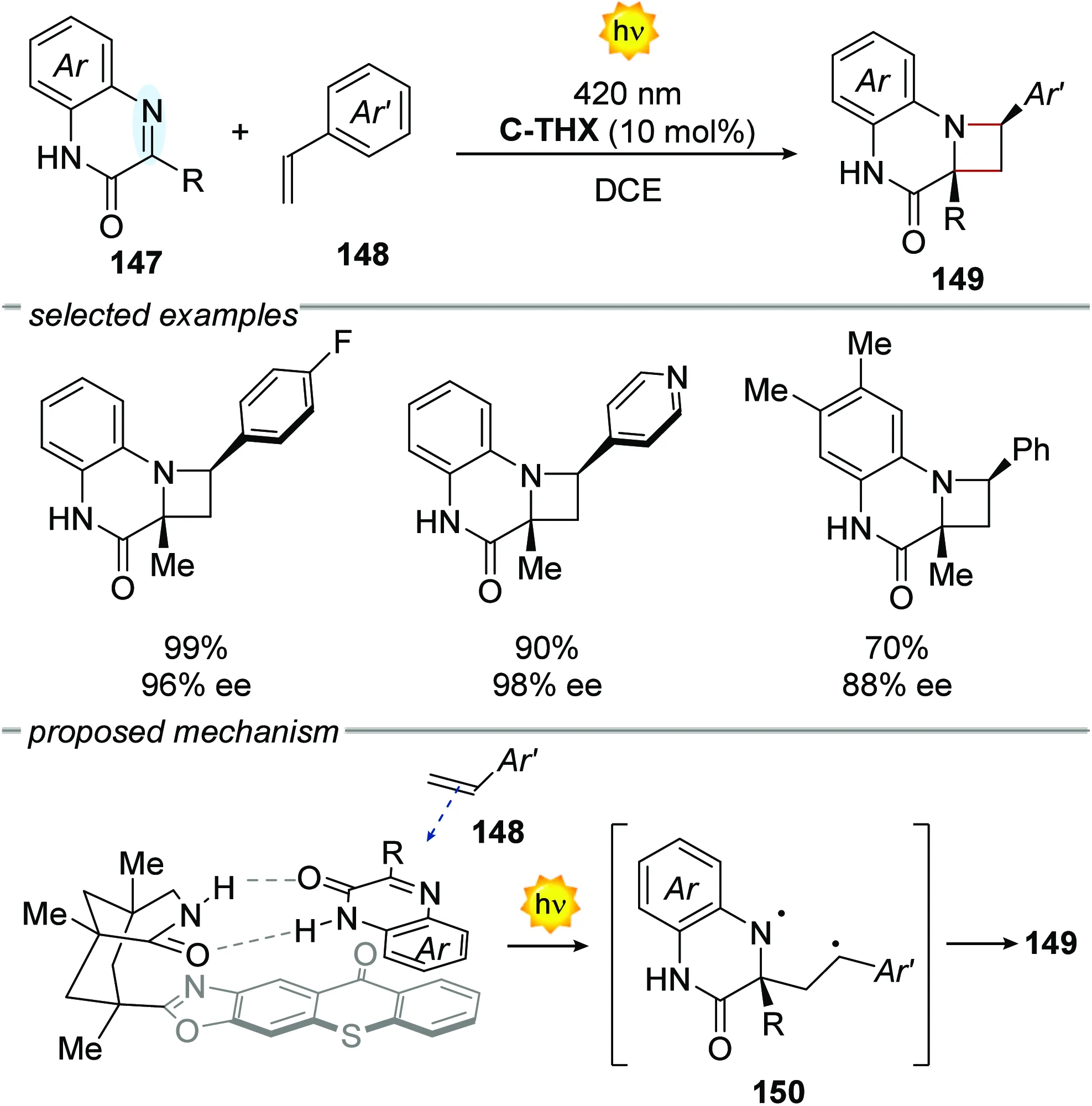
Enantioselective Intermolecular [2 + 2] Photocycloaddition of Quinoxalinones with Styrenes

Following this strategy, a few years later, the Hong group reported the same transformation using a thioxanthone catalyst based on a chiral [2.2]­paracyclophane core endowed with a carboxylic acid as the hydrogen-bonding donor/acceptor unit.^192^

Photocatalytic protocols enabling the [2 + 2] photocycloaddtion of quinoxalinones with styrene derivatives under visible light irradiation were also developed. Namely, Brascholz et al. reported an oxidative photocatalytic method for the *in situ* dehydrogenation of dihydroquinoxalinone under O_2_ atmosphere and ensuing aza-PB reaction with styrenes.^193^ Particularly, the process was found to be mediated by an iridium complex that acted both as photoredox catalyst in a radical-polar crossover sequence and as EnT photocatalyst. Besides, a photocatalytic aza-PB reaction of pyrazole-functionalized quinoxalinones with 4CzIPN as organophotoredox catalyst was reported by Liu and co-workers.^194^

Advances in the aza-PB reaction of azauracils, another cyclic imine typically employed this type of process, have been reported. Aitken and co-workers exploited the [2 + 2] cycloaddition of these heterocycles **151** with ethylene **152** under irradiation of a Hg lamp to access azetidine **153**, which can be conveniently elaborated to access *N*-aminoazetidinecarboxylic acids (Scheme 33a).^195^ A related photocatalytic variant was reported by Sureshkumar et al., with 6-azauracils undergoing [2 + 2] photocycloaddition with styrenes **148** via the triplet excitation of the alkene (Scheme 33a).^196^ In addition, the aza-PB reaction of 6-azauracils with alkenes was recently exploited for the positional determination of C–C double bonds in fatty acyl chains by HRMS spectroscopy.^197^

**Scheme 33:**
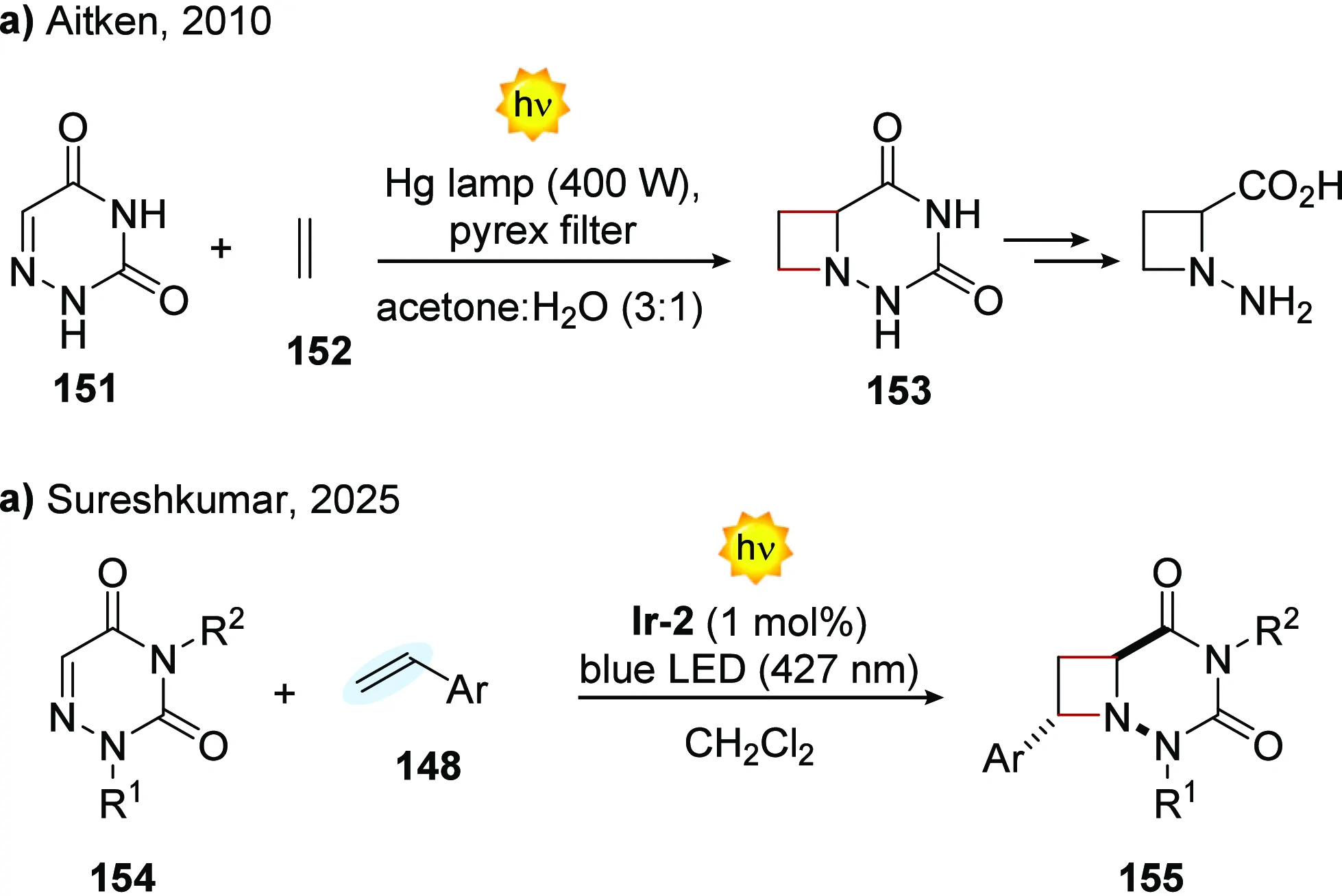
Advancements in aza-PB reactions of azauracils with alkenes

An intermolecular aza-PB reaction displaying unusual regioselectivity was reported in 2023 by Brown and co-workers.^198^ During their investigation of the [4 + 2] cycloaddition of activated naphthalenes with alkenes, the authors serendipitously discovered that benzoisothiazole could serve as cyclic imine partner in a photocatalyzed aza-PB reaction. Upon sensitization from an excited Ir photocatalyst, benzoisothiazole **156** underwent cycloaddition with alkenes **157** and alkynes **67** giving the corresponding azetidine **158** and azetine **159**, respectively (Scheme 34a). The products were obtained in high yield and diastereoselectivity. Interestingly, the observed regioselectivity is consistent with a mechanistic scenario where C–N bond formation precedes C–C bond formation in the photocycloaddition process—a bonding sequence that fundamentally deviates from previously reported aza-PB reactions. This proposal was supported by spin density calculations of **156-T**_**1**_, which revealed the highest spin density to be at the nitrogen atom.

**Scheme 34:**
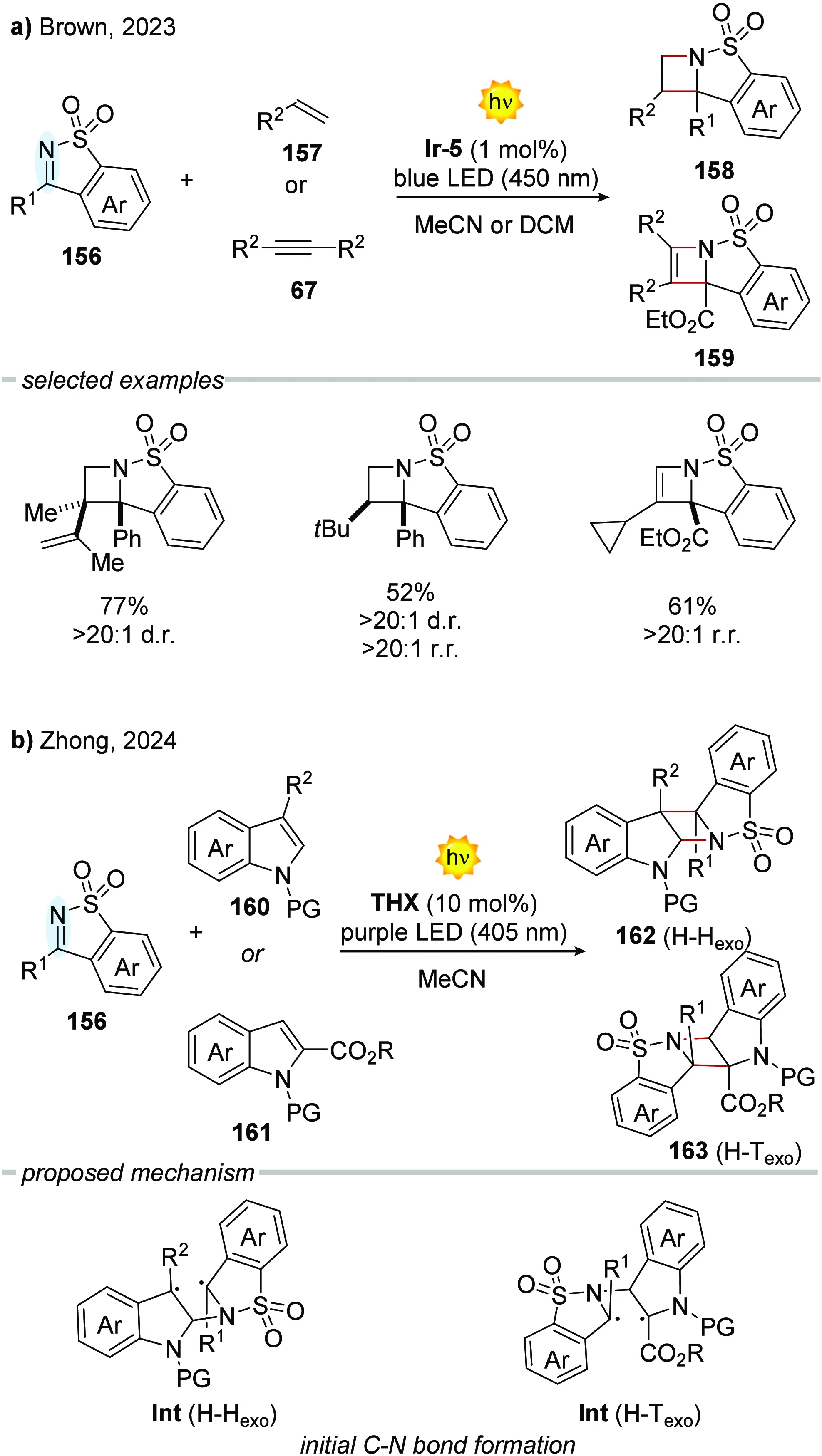
Intermolecular Photocatalyzed [2 + 2] Cycloaddition of Benzoisothiazoles with a) Alkenes and b) Indoles

In a related study by Liao, Zhong et al., benzoisothiazole **156** was also reported to undergo [2 + 2] cycloaddition with indole derivatives upon sensitization from a thioxanthone **THX** photocatalyst (Scheme 34a).^199^ Particularly, 3-substituted indoles **160** gave the head-to-head (H–H) product **162**, while indoles substituted at the C2 with an electron withdrawing group led to the head-to-tail (H-T) azetidine **163**. Again, such regioselectivity is consistent with the triplet benzoisothiazole **156** adding to the alkene via initial formation of a C–N bond, a pathway that was found to be kinetically more favored by DFT calculations. Moreover, in analogy with the previous study by the Brown group,^198^ both azetidine products **162** and **163** were exclusively obtained with *exo*-selectivity. Computational analyses revealed that this stereochemical preference is dictated by steric factors, being mainly distortion-controlled. Notably, the process was also extended to the cycloaddition of other aromatics, including benzofurans, benzothiophenes, and indenes.

More recently, the photocatalyzed [2 + 2] cycloaddition of 3-substituted benzoisothiazoles with alkenes has been combined with a subsequent Lewis acid-catalyzed ring expansion in a one-pot process, providing efficient access to 1,2-benzothiazepine 1,1-dioxides.^200^

In 2024 Schindler and Kulic groups developed an intermolecular [2 + 2] photocycloaddition of imines and styrenes that represented a further advancement toward more general aza-PB reactions.^201^ The authors observed that *acyclic* glyoxylate oximes **164**, which are activated by the conjugation of the oxime and ester group, could react with activated (conjugated) alkenes **165** in the presence of **Ir-2** photocatalyst under blue light irradiation (427 nm) to give azetidines **166** (Scheme 35). The methodology proved applicable to a broad range of activated alkenes, including styrenes, dienes, and cyclic olefins, enabling the preparation of up to 2,3,4-trisubstituted azetidines; late-stage functionalization of biologically relevant molecules was also demonstrated. Mechanistic investigation supported an energy-transfer pathway in which the photocatalyst preferentially sensitizes the alkene rather than the oxime to its triplet state, with the ensuing C–C bond formation being the rate-determining step. Most importantly, the authors established frontier orbital energy matching between the activated alkene and the oxime as the key factor governing productive cycloaddition. A strong correlation was found between the frontier orbital energy difference (ΔE_FO_) and the activation barrier for C–C bond formation, demonstrating that combining activated substrates possessing closely matched orbital energies is crucial to efficiently promote the cycloaddition and outcompete alkene dimerization. Moreover, a multivariate analysis revealed that reaction yields are influenced both by substrates ΔE_FO_ and oxime carbon solvent-accessible surface area (SASA), with better-matched orbital energies and less sterically hindered oximes leading to higher yields overall.

**Scheme 35:**
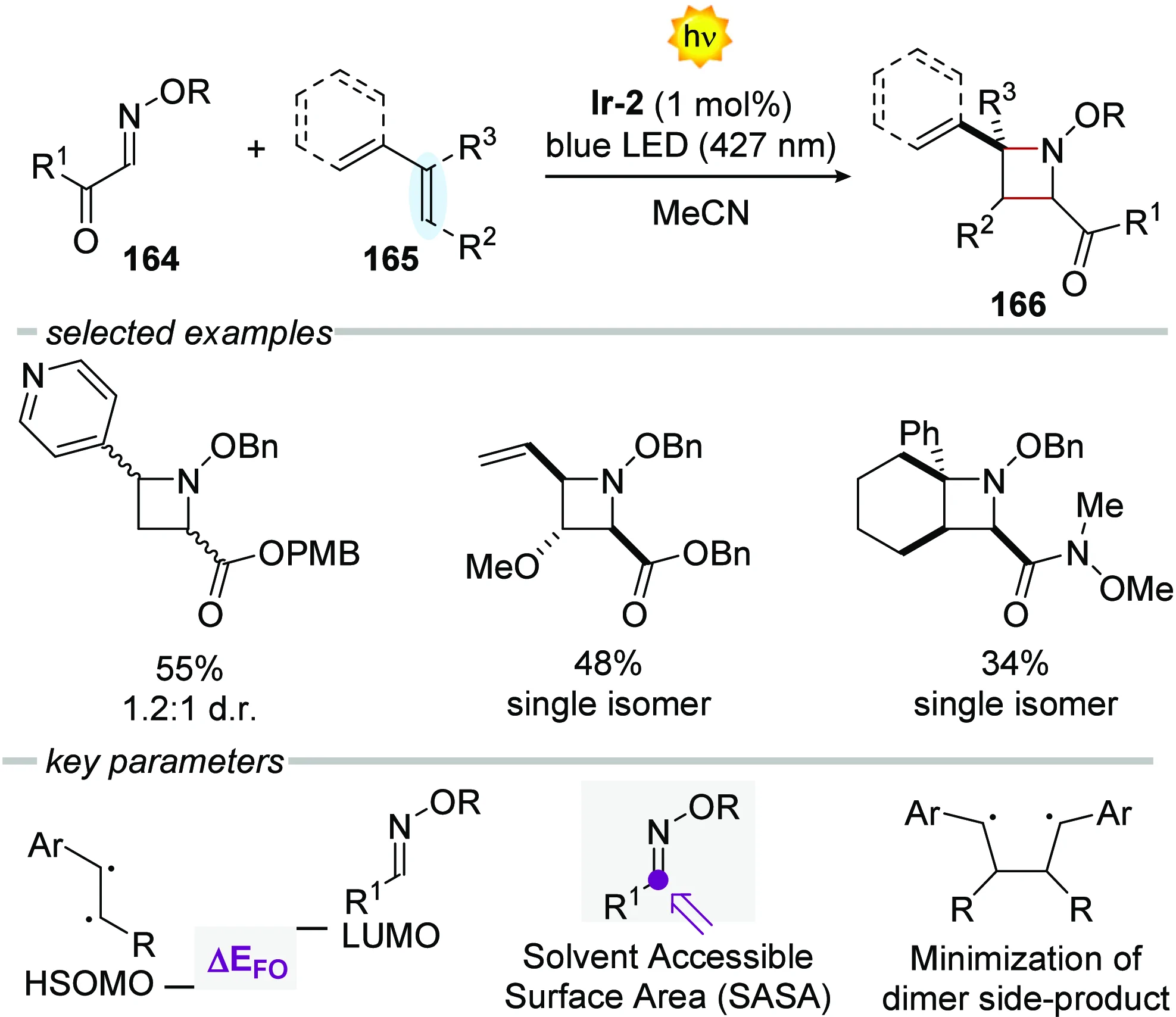
Intermolecular [2 + 2] Photocycloaddition of Acyclic Glyoxylate Oximes and Activated Styrenes Enabled by Styrene Sensitization

The high dependence on the oxime carbon SASA explains why ketone-derived oximes, which experience increased steric hindrance at the oxime carbon, are unreactive substrates for the protocol just mentioned. Wolf, Kulic, Schindler et al. envisaged that this limitation could be overcome through a change in the mechanism by switching the order of the bond forming events.^202^ This was achieved by using ketone-derived sulfonylimines **167** that could react in a related photosensitized protocol with activated alkenes **165** providing access to 2,2-disubstituted monocyclic azetidine products **168** (Scheme 36). DFT calculations confirmed that the reaction proceeds with the kinetically more accessible initial formation of the C–N bond, which leads to the more stabilized 1,4-diradical intermediate. While SASA calculation revealed that sterics still play a role, with the oxime N atom being relatively more accessible than the C atom, the reaction and its regioselectivity were found to be mainly driven by electronic factors. Notably, the reversed regioselectivity compared to the authors’ previous method provides a complementary approach to the preparation of 3-substituted azetidines. The *N*-tosylated products could be deprotected via a two-step reductive sequence.

**Scheme 36:**
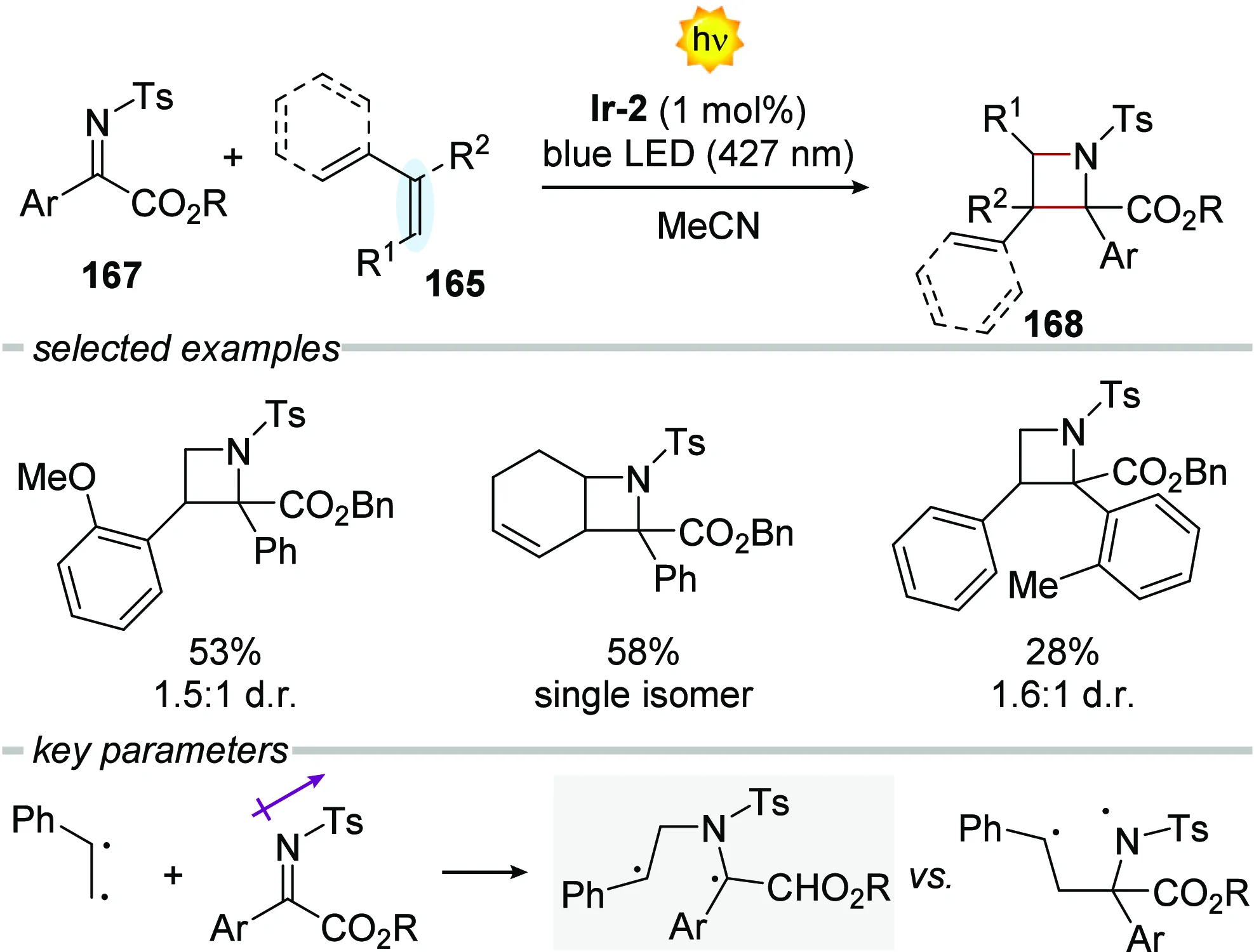
Intermolecular [2 + 2] Photocycloaddition between Acyclic Ketone-Derived Sulfonylimines and Activated Styrenes

Recently, inspired by works from Schindler and Zhong,^199,201^ the Cui group extended the photocatalytic sensitization of activated acyclic glyoxylate oximes to the cycloaddition with indole derivatives to access azetidine-fused indolines.^203^

During the same years, the intermolecular aza-PB reaction of *acyclic* imines was also targeted by the Willis group,^204^ although following a distinct mechanistic approach. Specifically, they disclosed that sensitization of *N*-fluorosulfonyl (−SO_2_F) substituted aryl aldimines **169** using a fluoro-substituted thioxanthone photocatalyst **3-F-THX** under 390 nm irradiation unlocked [2 + 2] cycloaddition with alkenes **170** (Scheme 37). Remarkably, the reaction design based on imine-sensitization enabled the functionalization of a variety of both activated and *unactivated* alkenes, providing access to variously substituted azetidines **171**. From a mechanistic perspective, computational studies revealed that the sulfonyl fluoride group plays multiple roles. First, it raises the energy of the N–S σ* orbital in the triplet excited state of the imine, thereby preventing unproductive homolytic cleavage. Second, it promotes addition to the alkene through a stepwise mechanism initiated by a barrierless C–N bond-forming event. Notably, the versatility of the products was demonstrated by several downstream manipulations, including functionalization of the sulfamoyl fluorides moiety by SuFEx click-chemistry.^205,206^

**Scheme 37:**
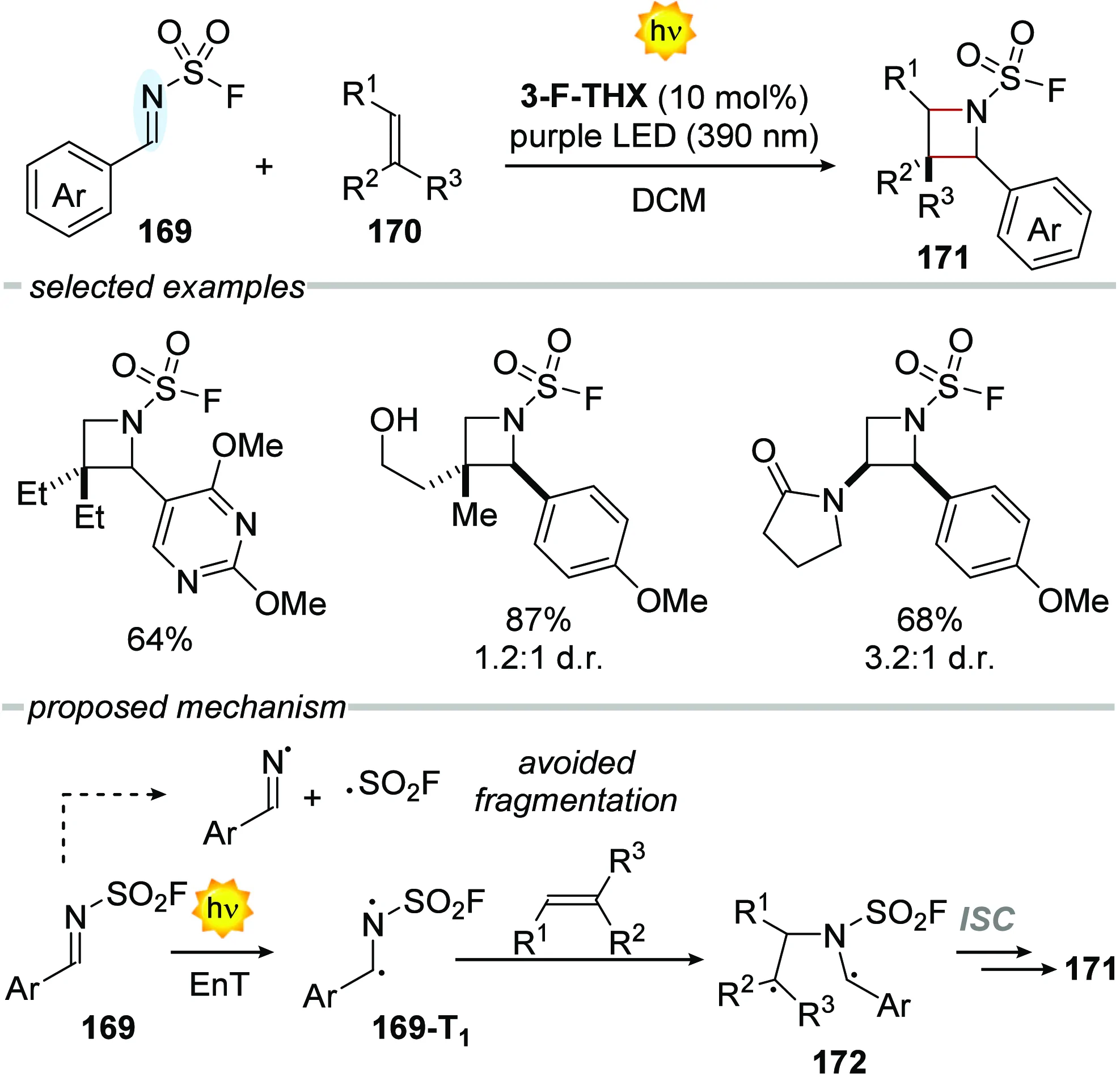
Intermolecular [2 + 2] Photocycloaddition of Acyclic *N*-Fluorosulfonyl Substituted Aryl Aldimines and Alkenes Enabled by Imine Sensitization

The same transformation was reported shortly after by Yamahuci and Hashimoto using an **Ir-2** as EnT catalyst under 427 nm irradiation.^207^

### 2.5 (Aza)­β-Lactams

The venerable Staudinger cycloaddition, namely the thermal [2 + 2] cycloaddition of ketenes **173** and imines **174**, represent one of the most straightforward approaches for the synthesis of β-lactams **175** (azetidin-2-ones) (Scheme 38).^208–210^ The generally accepted mechanism proceeds through a stepwise pathway initiated by the nucleophilic attack of the imine on the ketene, generating a zwitterionic intermediate that subsequently undergoes conrotatory ring closure to furnish the β-lactam ring.^211,212^ For unsymmetrical ketenes, the nucleophilic attack of the imine preferentially occurs with *exo*-selectivity from the less hindered face. In principle the reaction is stereospecific, with *E*-imines favoring the formation of *cis* β-lactams and *Z*-imines giving the corresponding *trans* isomers. However, rotation about the C–N bond of the zwitterionic intermediate can occur prior to ring closure, resulting in partial or complete erosion of stereospecificity (Scheme 38, bottom).

**Scheme 38:**
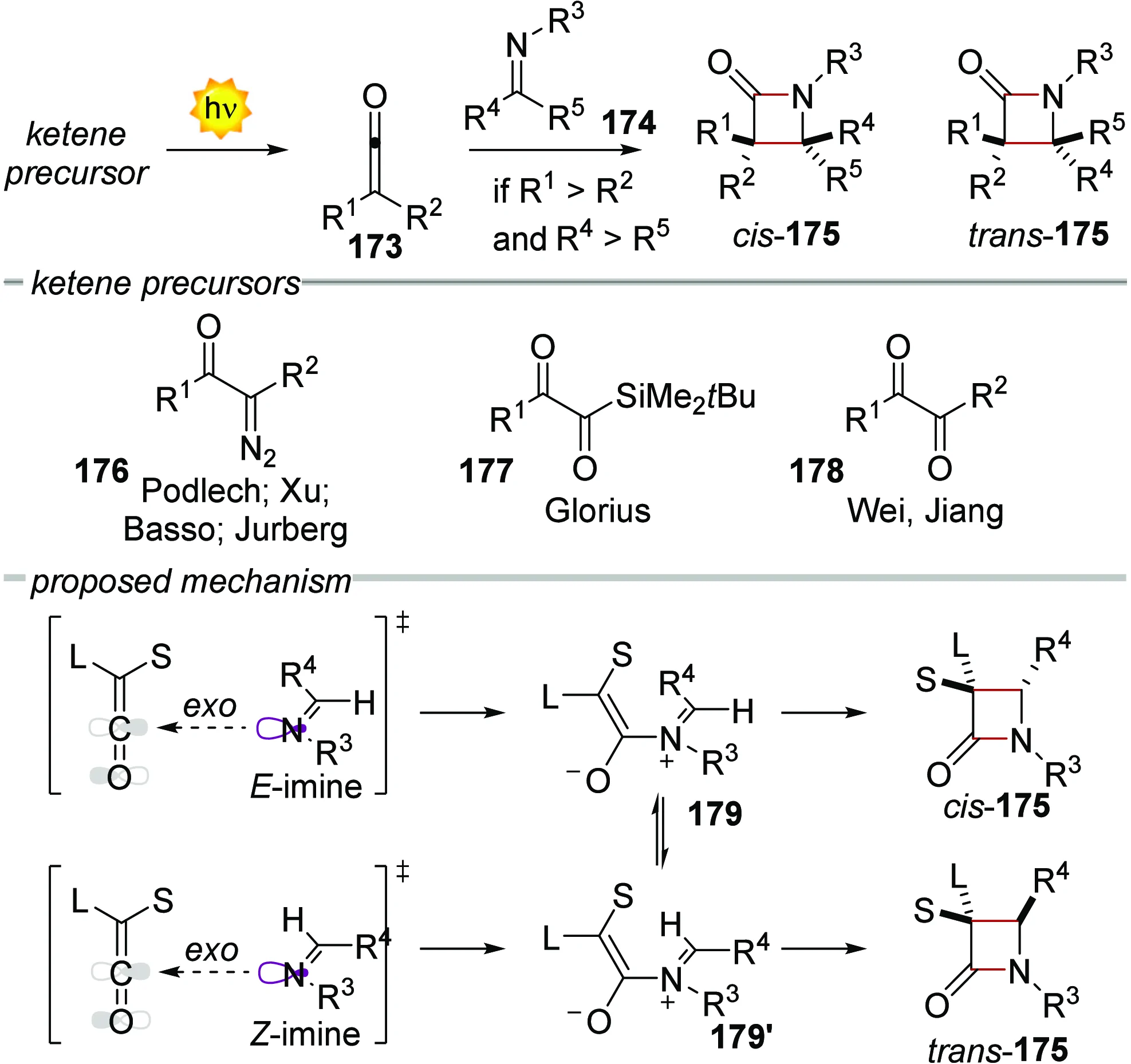
Ketene Precursors and Mechanism of Visible-Light Driven Staudinger Cycloaddition

#### 2.5.1 Direct Irradiation Methods

Among the various methods developed to generate ketene intermediates for the Staudinger cycloaddition, light-driven strategies have emerged as an effective approach (Scheme 38).^50^ First studies were conducted by Podlech et al., who exploited the Wolff rearrangement of diazoketones **176** derived from amino acids, which were activated by direct excitation using UV light.^213–217^ β-Lactams derived from *E*-imines were obtained with *trans* stereoselectivity, which was initially attributed either to photoexcitation and subsequent isomerization of the zwitterionic intermediate **179** or to *E*/*Z* photoisomerization of the imine substrate. This hypothesis was later revisited by Xu and co-workers, who demonstrated that the stereochemical outcome of the Staudinger cycloaddition of diazoketones **176** is primarily governed by electronic effects.^211,218–220^ In particular, electron-withdrawing substituents on the ketene decrease the rate of ring closure, allowing isomerization of the zwitterionic intermediate to compete effectively. Consequently, photoexcitation of the zwitterionic intermediate was ruled out as the origin of the observed stereoselectivity.

More recently, in 2021, the Basso group reported a visible-light-mediated Staudinger cycloaddition enabled by the blue-light (450 nm) activation of diazoketones **176**.^221–223^ Notably, the methodology was further extended to a three-component protocol in which the imine partner was generated *in situ* from the corresponding carbonyl compound and an excess of amine. The same year, Judberg and colleagues reported a related two-steps protocol to the Staudinger cycloaddition.^224^ Here, visible light was first used to activate aryl diazoacetates toward the formation of carbenes, which were reacted with azides to afford the corresponding imines. These were then reacted with aryldiazoketones under visible light irradiation to access 2,3,3-trisubstituted β-lactams. On a similar note, the visible light-driven Wolff rearrangement of diazoketones was also exploited by Zhang et al. for the cycloaddition of ketenes with cyclic imines to synthesize tetracyclic dibenzo­[*b*,*f*]­[1,4]­oxazepine-fused β-lactams.^225^

Interestingly, in 2021 the Glorius group demonstrated that α-ketoacylsilanes **177** can react with a variety of aldimines, ketimines, and hydrazones under blue light irradiation to access an array of β-lactams with generally good to excellent diastereoselectivities.^226^ The process relied on the direct phoexcitation of α-ketoacylsilanes that undergo [1,3]-silyl migration to form ketene intermediates, as supported by DFT calculations. Finally, the cycloaddition of dibenzyl derivatives **178** with imines was reported by Jiang et al. via a deoxygenative process enabled by triethyl phosphite and a dicyanopyrazine organic photoredox catalyst under blue LED irradiation.^227^

An interesting application of this reactivity was shown by Zhan, Li, Huang and co-workers who used cyclic imines to synthesize spiro compounds.^228^ Namely, α-diazoketone **180** and cyclic ketimines derived from pyrazolone **181** and other heterocyclic compounds **182** reacted under blue light irradiation via Staudinger cycloaddition to give the complex spirocyclic **183** and **184**, respectively (Scheme 39a). Intriguingly, besides driving the photochemical activation of diazoketone to ketene, visible light irradiation was also required to promote the cycloaddition step.

**Scheme 39:**
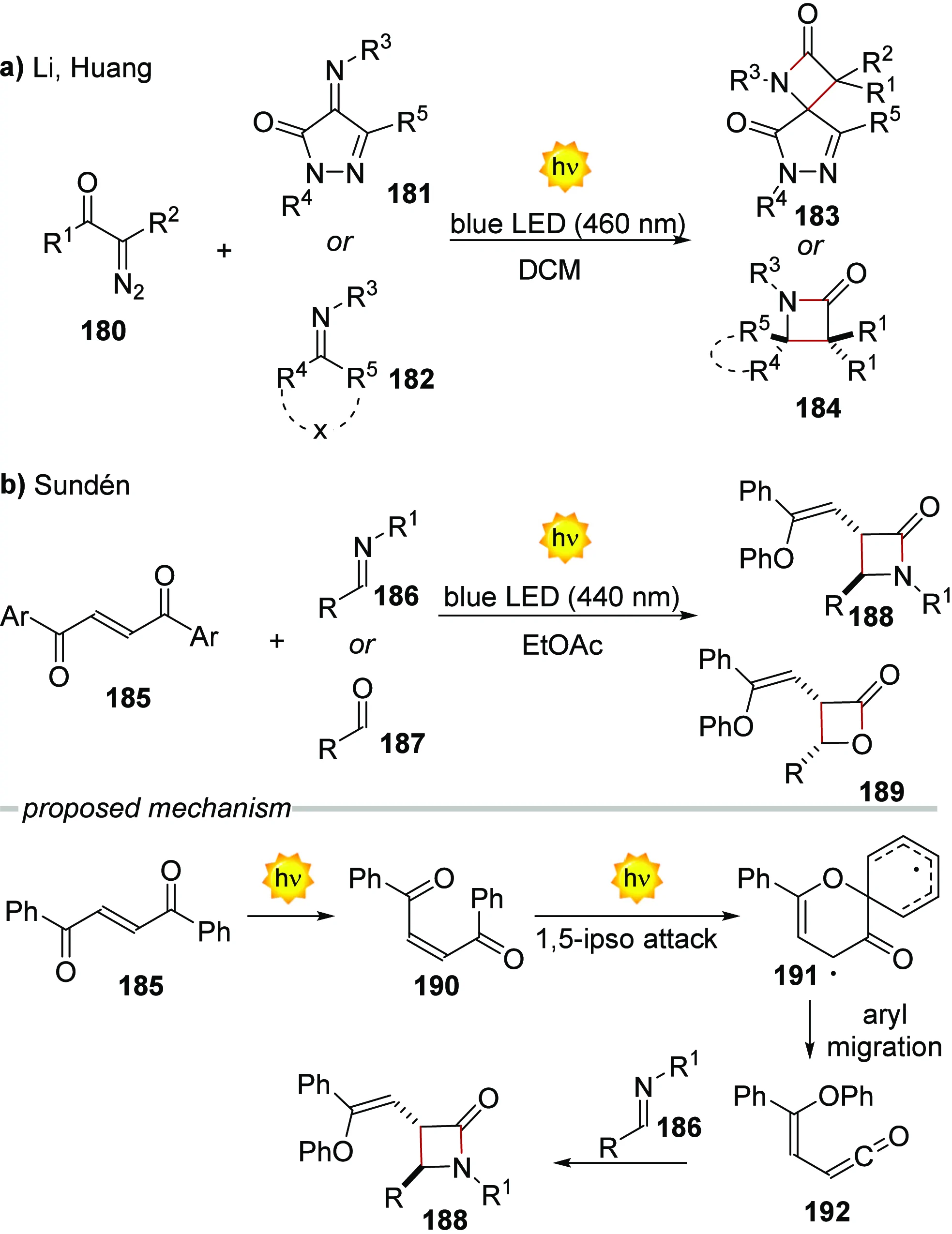
a) Staudinger Cycloaddition with Cyclic Ketimines to Spirocycles; b) Photoactivation of Dibenzoyl Ethylene to Ketene for Staudinger Cycloaddition

An alternative method for ketene generation in a Staudinger cycloaddition process was reported by the Sundén group.^229^ This method relied on the photochemical Zimmerman–O’Connell–Griffin rearrangement of dibenzoyl ethylene derivatives **185**,^230–232^ that could be reacted with imines **186** to give β-lactams **188** with exclusive *trans*-selectivity (Scheme 39b). Notably, this chemistry could be applied also to the cycloaddition of carbonyl compounds **187** to give lactones **189** with *cis*-stereoselectivity. The reaction mechanism involves the *E*-*Z* photoisomerization of dibenzoyl ethylene **185** to give intermediate **190** that, after a second photoexcitation, undergoes 1,5-ipso attack to the phenyl ring forming intermediate **191**. Then, aryl migration gives the ketene intermediate **192**, which is intercepted by imine **186** to form the final β-lactams **188**.

In 2024, Li, Yang, Han et al. showed that α-diazoketones **180** can react with arylazo esters **193** under visible-light activation to access the corresponding 1,2-diazetidin-3-ones **194** (Scheme 40a).^233^ It is worth noting that, electron donating groups on the aryl ring of azo esters are not tolerated as they induced an alternative (3 + 2) cycloaddition leading to γ-fused lactams.

**Scheme 40:**
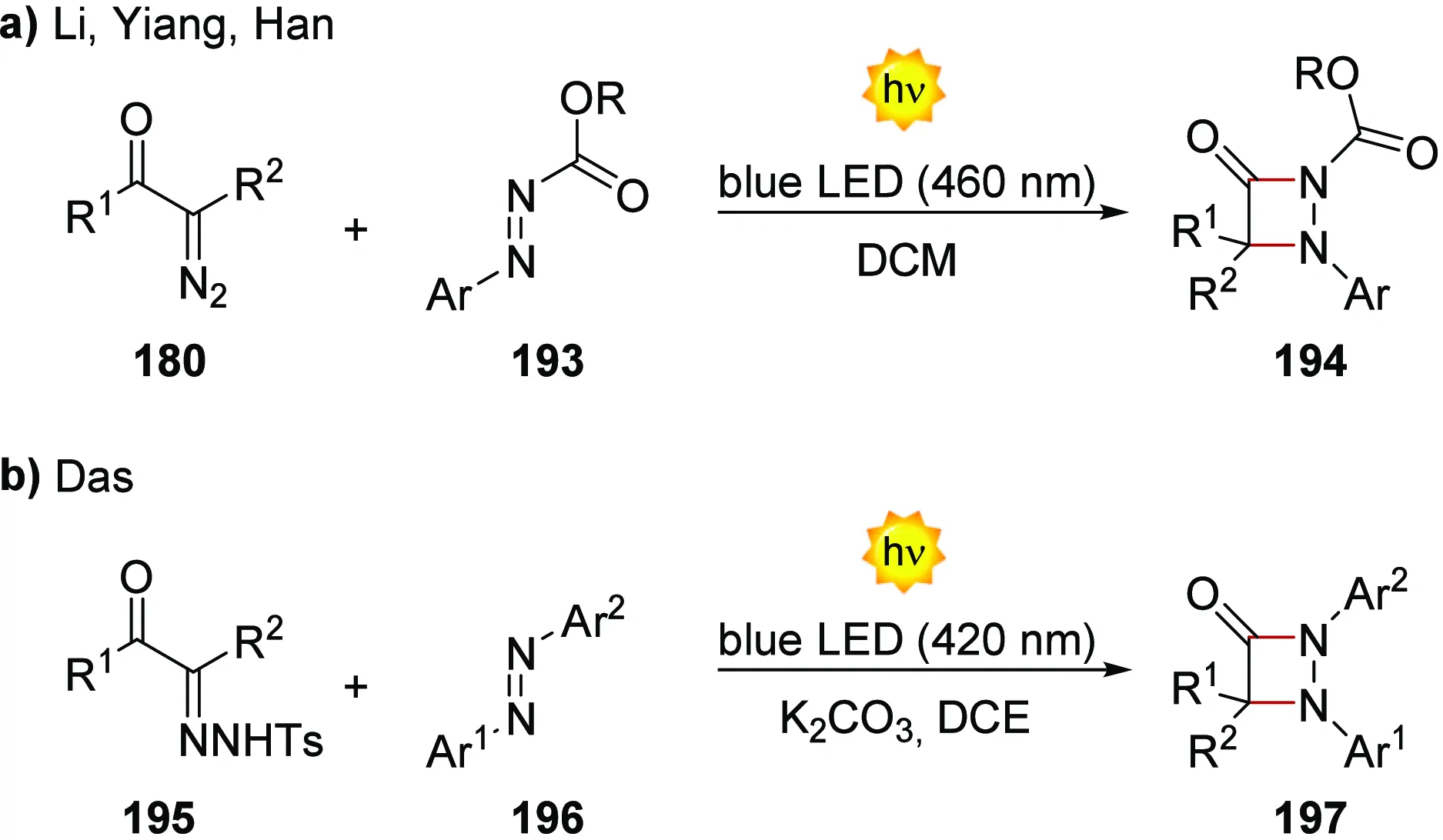
Intramolecular Staudinger Cycloaddition with Diazo Compounds to Aza-β-lactams

Recently, Das et al. disclosed *N*-tosylhydrazones **195** as precursors of α-diazoketones in Staudinger cycloaddition reaction (Scheme 40b).^234^ Through DFT calculations, the authors proposed that these species undergo deprotonation in the presence of an inorganic base to generate an anionic intermediate, which upon visible-light irradiation fragments to afford the corresponding α-diazoketone. Subsequent light-induced Wolff rearrangement followed by cycloaddition with azobenzene derivatives **196** furnished the desired aza-β-lactams **197**. Importantly, this strategy avoids the direct handling of hazardous diazo precursors. Besides promoting the fragmentation of the anionic intermediate and the subsequent Wolff rearrangement, light was also proposed to play a second role, inducing the photoisomerization of *trans*-azobenzene to the more reactive *cis* isomer.

### 2.6 Thietanes

The [2 + 2] photocycloaddition between thiocarbonyl compounds and alkenes, commonly referred to as the thia Paternò–Büchi (thia-PB) reaction, has traditionally relied on the direct photoexcitation of the thiocarbonyl substrate, typically requiring relatively harsh irradiation conditions and specialized photochemical setups.^235–238^ Moreover, the intrinsically high reactivity of thiocarbonyl derivatives often results in competing side reactions, which has limited the development of thia-PB chemistry compared to its carbonyl and aza-PB counterparts. Consequently, synthetic applications have largely remained confined to specific classes of thiocarbonyl compounds, most notably thioamides, thioesters, xanthates, and thioureas.^239^ Depending on the nature of the excited state, distinct reactivity patterns are observed.^240^ Reactions proceeding from the T_1_(n,π*) state generally afford thietanes with high regioselectivity but poor stereocontrol through a stepwise biradical pathway and preferentially involve electron-rich alkenes. In contrast, excitation to the singlet S_2_(π,π*) state typically leads to highly stereoselective, albeit less regioselective, cycloadditions, which have been proposed to proceed through a more polar or excimer-like intermediate. Consequently, the nature of the excited state plays a decisive role in controlling both the reaction mechanism and the selectivity of the thia-PB reaction.

#### 2.6.1 Direct Irradiation Methods

Building on these early mechanistic foundations, subsequent studies explored how specific thiocarbonyl scaffolds could be exploited synthetically despite the intrinsic instability of many thietane products. In several cases, the initially formed thia-PB cycloadducts could be isolated as strained spiro- or fused thietanes, whereas in others they underwent rapid ring opening, fragmentation, or intramolecular trapping to give more stable downstream products.^241^ Representative examples of these direct-irradiation processes are summarized in Scheme 41, highlighting both isolable thietanes **200**, **201**, **203**, and products **202** and **205** arising from subsequent transformations of transient thietane cycloadducts.

**Scheme 41:**
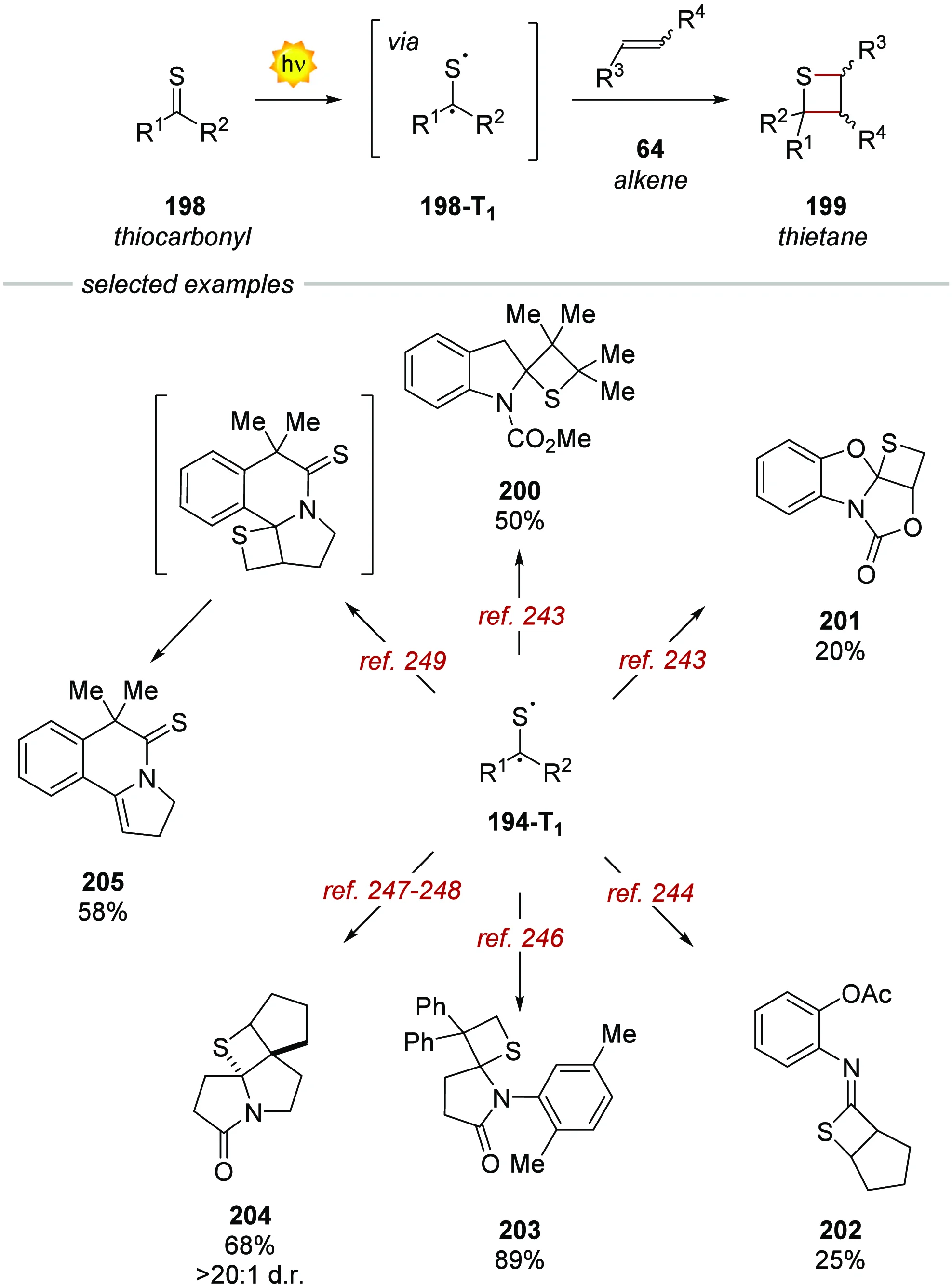
Representative Direct Thia-Paternò–Büchi Reactions of Thiocarbonyl Compounds with Alkenes and Selected Downstream Transformations of Transient Thietane Cycloadducts

Nishio and co-workers investigated benzoxazole-2-thione derivatives as thiocarbonyl partners in direct photochemical additions to alkenes.^242^ In their initial study on *N*-acylbenzoxazole-2-thiones, irradiation in the presence of alkenes was proposed to generate spirocyclic aminothietanes by regioselective C=S/C=C [2 + 2] photocycloaddition, although these primary adducts often underwent subsequent ring opening to furnish 2-substituted benzoxazoles and iminothietanes. Nishio and co-workers later showed that installation of alkoxycarbonyl or aryloxycarbonyl substituents on nitrogen stabilizes the thietane products, enabling the isolation of spiro­[benzoxazole-thietanes] such as **200**.^243^ The same study also disclosed an intramolecular variant, in which an ethenyl 2-thioxobenzoxazole-3-carboxylate furnished tetracyclic thietane **201**. Subsequent expansion to cycloalkenes and heteroaromatic partners further supported the intermediacy of spirocyclic aminothietanes, with cyclopentene providing the isolable iminothietane derivative **202** after intramolecular trapping of the initially formed zwitterionic intermediate.^244^

Stereochemical control in thia-PB chemistry was explored by Sakamoto and co-workers in two complementary settings. In the solid state, suitably organized monothioimide substrates underwent topochemically controlled intramolecular [2 + 2] photocycloaddition to give optically active thietane-fused β-lactams, illustrating how crystal packing can direct both reactivity and stereochemical outcome.^245^ In a related intermolecular variant, axially chiral monothiosuccinimides reacted with 1,1-diphenylethylene to afford spirothietanes such as **203** with high diastereoselectivity. These studies show that thia-PB reactions can provide not only strained sulfur-containing heterocycles, but also stereochemically enriched thietane frameworks.^246^ In a contemporaneous sulfur-based extension of hetero-[2 + 2] photochemistry, Padwa and co-workers investigated the intramolecular photocycloaddition of cyclic thioimides with tethered alkenes. In their initial report, irradiation of *N*-alkenyl thioxopyrrolidinones promoted thia-PB-type cycloaddition to afford highly strained spirocyclic amidothietanes such as **204**.^247^ In a subsequent full study, the same group expanded this strategy to additional *N*-alkenyl thioimides and dithione derivatives, while also revealing competing pathways such as γ-hydrogen abstraction from the *n−π* triplet state in selected thioxaphthalimide systems.^248^ Together, these studies established thioimide–alkene photocycloaddition as a useful entry to strained amidothietanes and fused azacyclic scaffolds. Related intramolecular thia-PB reactivity was later observed by Takechi and co-workers with thiohomophthalimides bearing an alkenyl group in the *N*-side chain.^249^ In these systems, photocycloaddition occurs regioselectively at the aromatic thiocarbonyl group rather than at the aliphatic thiocarbonyl unit. The initially formed fused thietane is not isolated, but undergoes fragmentation to ene-thiolactam products such as **205**. This example is therefore analogous to other transient hetero-[2 + 2] cycloadducts discussed in this Review: the isolated product is generated downstream, but the key photochemical bond-forming step remains a C=S/C=C cycloaddition involving direct incorporation of sulfur into a four-membered ring.

Thia-PB chemistry has also been used in more specialized contexts: Carell and co-workers employed thietane formation as a preparative step toward stable sulfur analogues of oxetane intermediates proposed in DNA photolyase repair, and then compared the flavin-induced cleavage behavior of oxetane and thietane model compounds. Although the main focus of that study was cycloreversion rather than thietane synthesis, it further illustrates the utility of photochemically generated thietanes as isolable mimics of otherwise unstable oxygen-containing intermediates.^250^

In 2020, Boddaert, Aitken, and co-workers reported an intriguing divergence in the thia-Paternò–Büchi reaction between thiobenzophenones **206** and electron-deficient alkenes **207**, showing that product selectivity could be controlled simply by varying the reaction concentration (Scheme 42).^251^ At high thiocarbonyl concentrations, the expected thietanes **208** were obtained selectively, whereas dilution favored the formation of tetrahydrothiophenes **209**. Experimental studies established that **209** arises from the subsequent reaction of thietane **208** with a second equivalent of thiobenzophenone **206**. Mechanistically, excitation of the thiocarbonyl to the S_2_ (π,π*) state was proposed to promote the initial [2 + 2] photocycloaddition with alkene **207**, while population of the T_1_ (n,π*) state leads to carbene **210** plausibly via a hydrogen atom transfer step. The latter reacts with thietane **208** to generate a sulfonium ylide intermediate **211**, which undergoes a regioselective thia-Stevens rearrangement to furnish tetrahydrothiophene **209**. The concentration dependence was rationalized by self-quenching of excited thiobenzophenone at higher substrate loadings, which suppresses the triplet pathway responsible for carbene formation and consequently favors thietane **208**. Notably, this reactivity was exploited in a two-step protocol, whereby isolated thietanes could be intercepted with a different thiobenzophenone, providing access to variously substituted tetrahydrothiophenes. Despite these promising results, the synthetic applicability of the methodology toward thietanes was demonstrated for only three examples. Moreover, these thietane derivatives were found to undergo decomposition upon prolonged irradiation, further underscoring the intrinsic challenges associated with the development of efficient thia-Paternò–Büchi reactions.

**Scheme 42:**
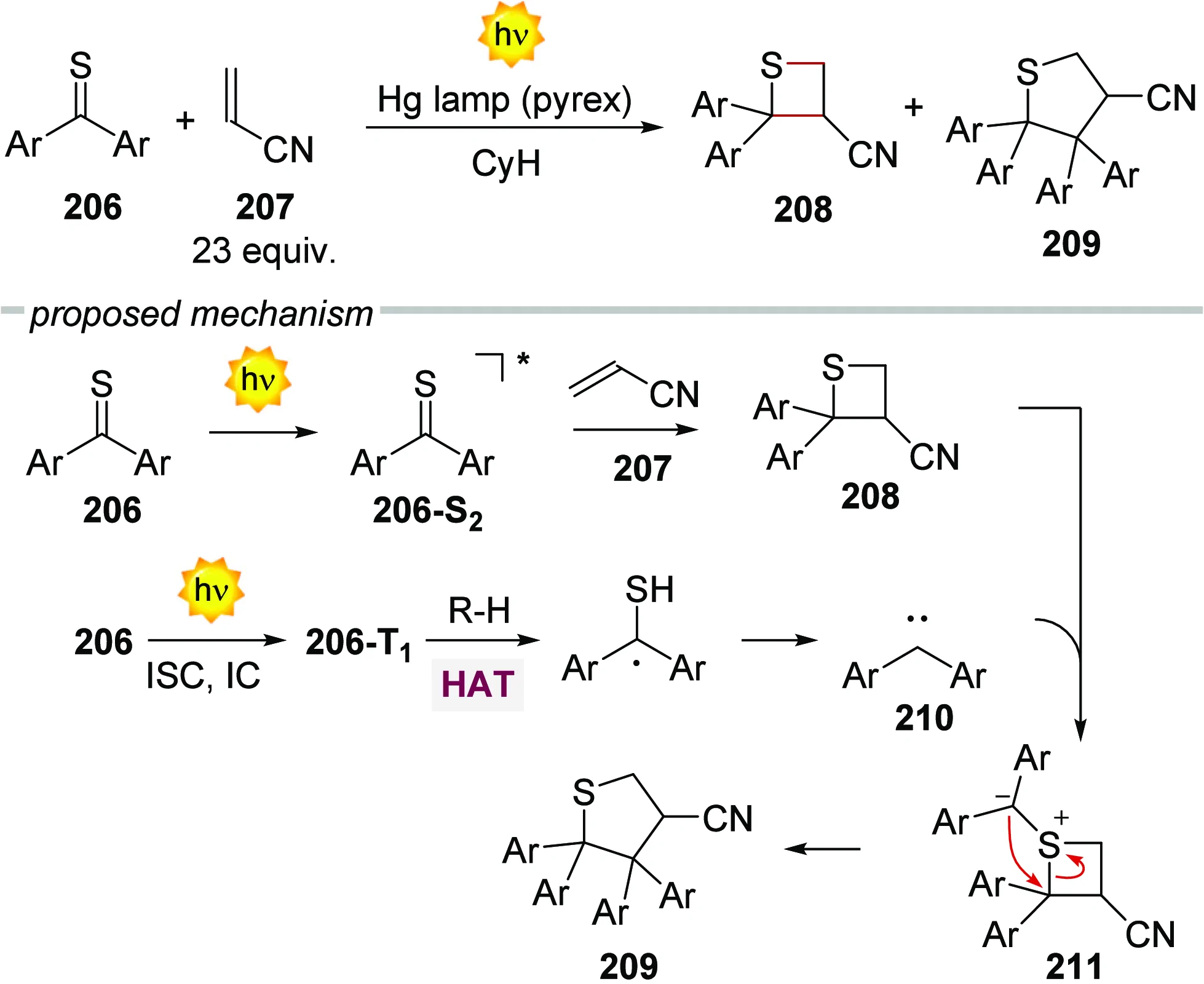
Divergent Intermolecular Thia-PB Reaction of Thiocarbonyls with Electron-Poor Alkenes

To circumvent the isolation and handling of unstable thiocarbonyl compounds while expanding the scope of their previous methodology, Boddaert and co-workers developed a light-driven domino strategy based on the *in situ* generation of thiocarbonyl intermediates.^252^ The protocol exploits the UV-light excitation of phenacyl sulfides **212**, which undergo a Norrish type II fragmentation to generate the corresponding thiocarbonyl species in the presence of an excess of electron-deficient alkenes **213** (Scheme 43). Under these conditions, a broad range of thiobenzophenones and electron poor alkenes afforded the corresponding thietanes in moderate to excellent yields. Most notably, the methodology enabled the efficient generation and ensuing thia-PB cycloaddition of highly reactive alkyl aryl thiocarbonyls, which are otherwise difficult to isolate and handle. Electronic effects strongly influenced the regioselectivity, with the highest selectivities observed for the most polarized substrate combinations. This observation is consistent with a mechanism involving nucleophilic attack of the sulfur atom of the **S**_**2**_ (π,π*)-excited thiocarbonyl onto the electron-deficient β-carbon of the ground-state alkene. Both steric and electronic factors were found to influence the diastereoselectivity of the cycloaddition when alkyl aryl thiocarbonyls were employed.

**Scheme 43:**
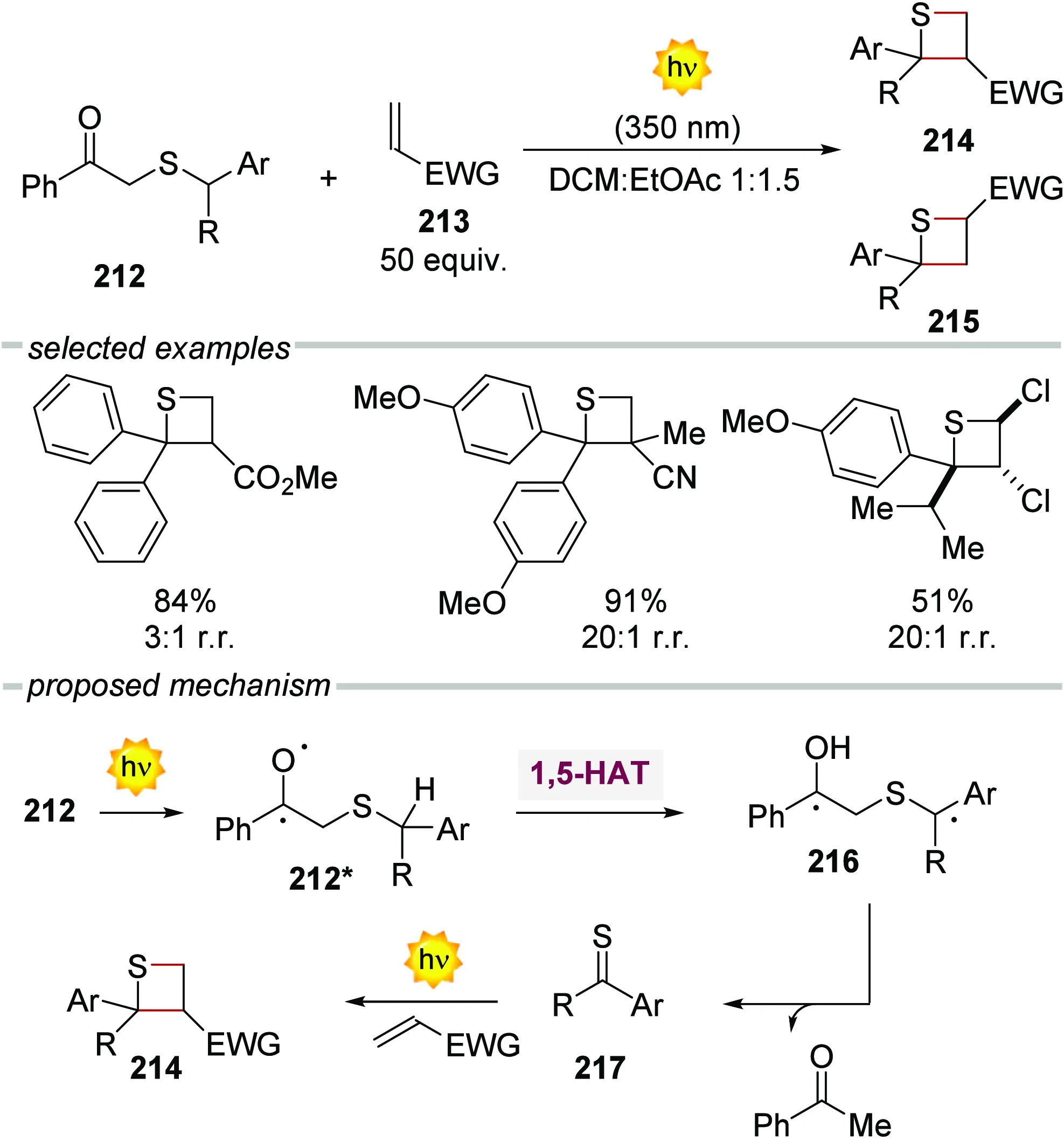
Light-Driven Domino Norrish Type II/Thia-PB Reaction of Phenacysulfides

Capitalizing on their domino photochemical sequence, the Boddaert group developed a visible light-driven protocol enabling a general thia-PB reaction of a variety of thiocarbonyls with electron rich alkenes.^253^ The method development was informed by seminal studies that showing that thiocarbonyls react with electron rich alkenes from the T1 (n, π*) state, which can be reached by excitation of a transition in the visible range. *In situ* generation of thiocarbonyls was achieved using pyrenacyl sulfide **218** that was reacted with an excess of alkene **170** under 405 nm irradiation to give thietane **219** (Scheme 44). Remarkably, the method enabled to engage several alkenes, including vinyl ethers and enamines. Most importantly, dropwise addition of the thiocarbonyl precursor enabled to expand the protocol to the functionalization of dialkylthioketones as well as alkyl and aryl thioaldehydes, which are challenging substrates in thia-PB reaction. Mechanistic studies revealed that, although direct excitation of the thiocarbonyl is possible, the reaction is accelerated by EnT from the excited 1-acetylpyrene **220** that is stoichiometrically formed under the reaction conditions upon Norrish type II cleavage of the pyrenacyl sulfide **218**. The increased efficiency provided by such a triplet sensitization mechanism is posited to be responsible for the efficiency of the reaction even with relatively low excess of the alkene partner (compared to previously reported protocols). High alkene excess are commonly required to disfavor further incorporation of thiocarbonyl that leads to the formation of 1,4-dithiopyranes, a side-product that was not observed in this study.

**Scheme 44:**
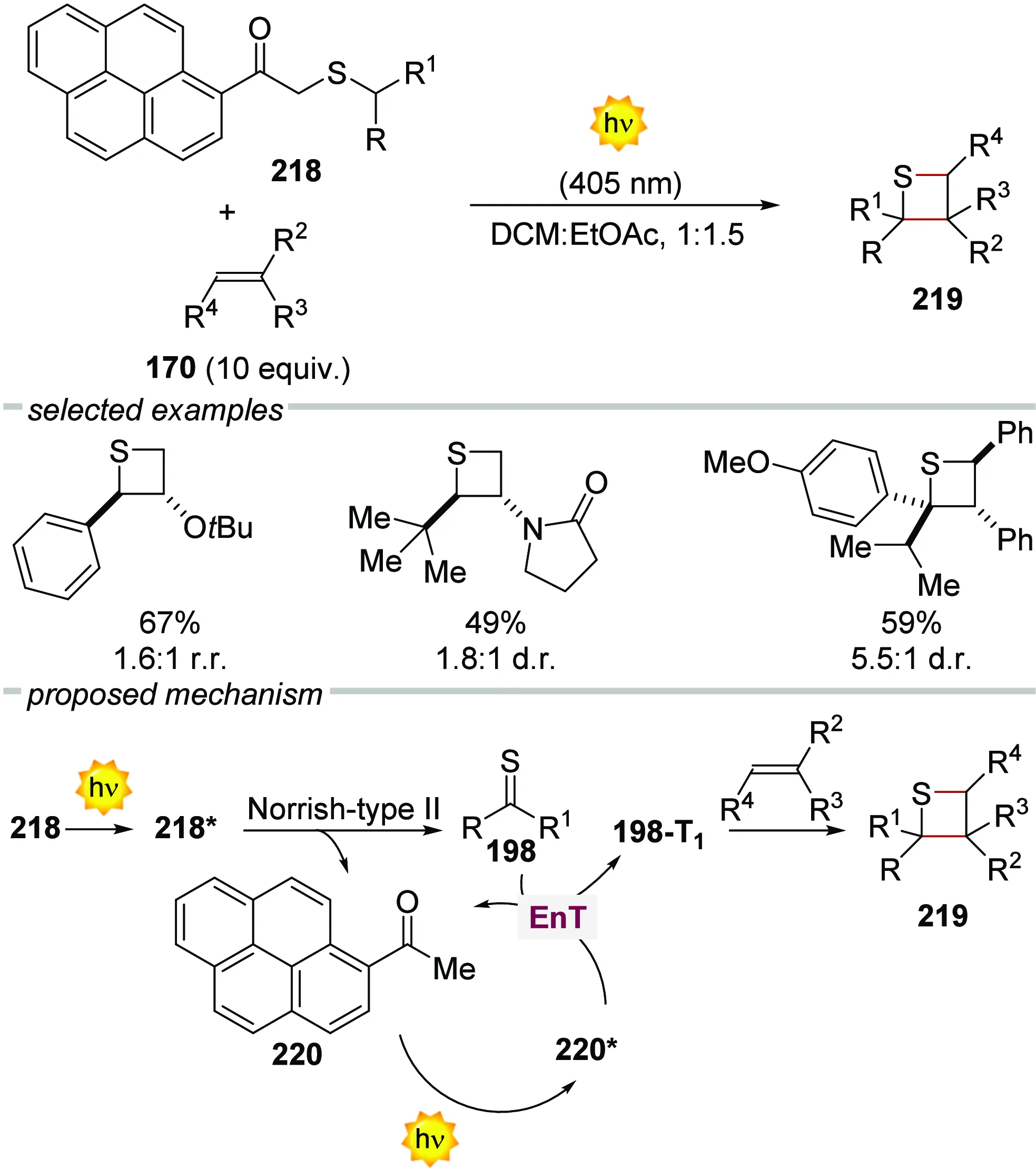
Intermolecular Thia-PB Cycloaddition of *in Situ* Generated Thiocarbonyls and Electron-Rich Alkenes

An interesting application of the rich photochemistry of 2-azaarenthiones in thia-PB cycloaddition was reported by Jiang and co-workers.^254^ The authors showed that blue light irradiation of 2-azaarenethiones **221** in the presence of alkenylboronates **222** provides access to vinyl azaarenes **223** (Scheme 45). A broad range of azaarene scaffolds was tolerated, and both aryl- and alkyl-α-Bpin-substituted olefins proved suitable substrates. Both terminal and internal alkenes were readily accommodated. Mechanistically, the reaction is proposed to proceed through a [2 + 2] cycloaddition of the excited 2-azaarenthiones **221** and alkene **222** to give the thietane intermediate **225**. Then, a rearomatization drives the thietane ring opening to give thiol **226**. Finally, the presence of a leaving-group on the olefine drives the 1,2-elimination to give product **223**.

**Scheme 45:**
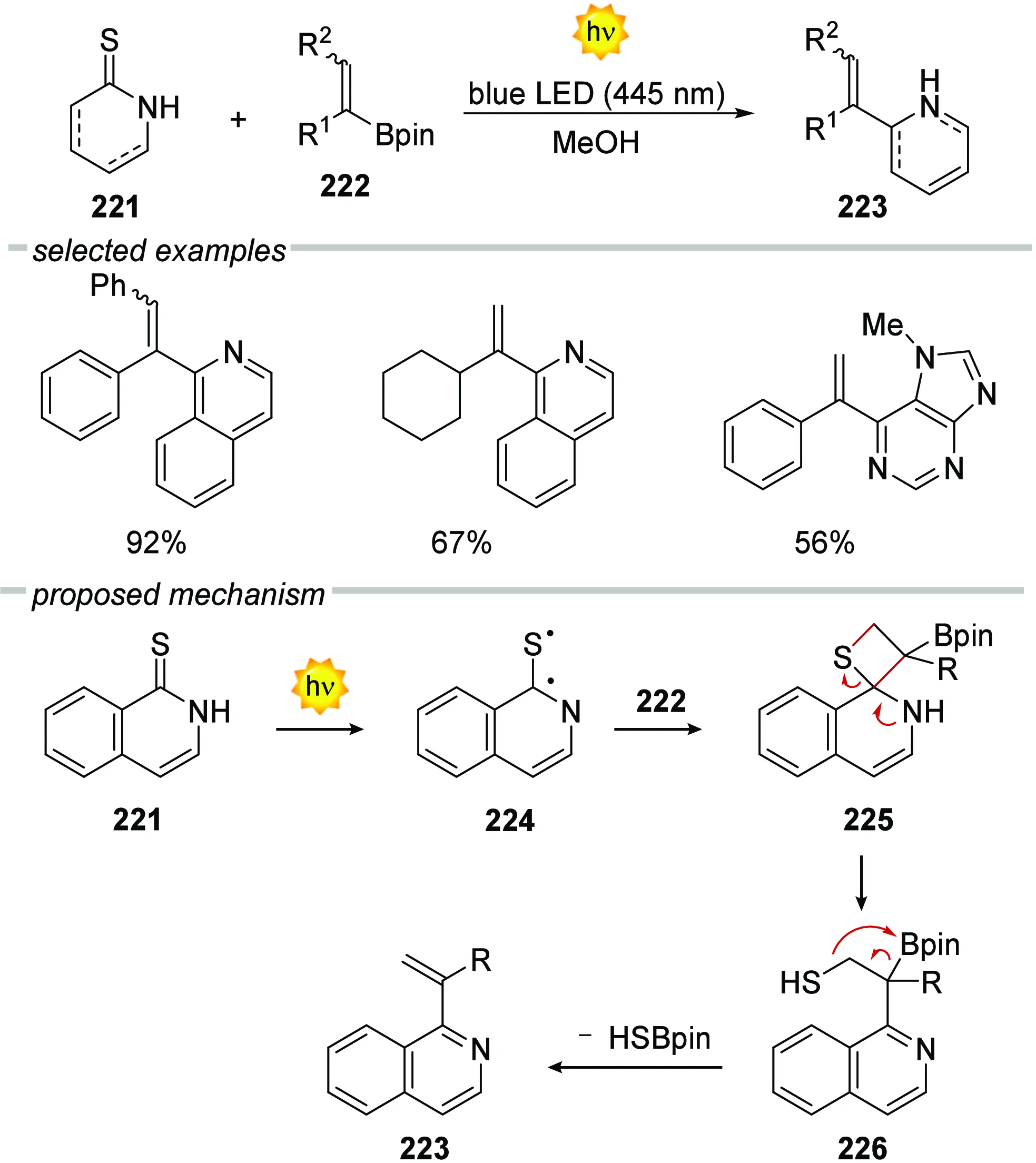
Vinyl Azaarene Synthesis via Thia-PB Reaction of 2-Azaarenthiones and Alkenylboronates

#### 2.6.2 Photocatalytic Methods

Capitalizing upon the concept of triplet sensitization, in 2021 Liu et al. reported a general thia-PB reaction between maleimides **227** and xanthates **228** to access functionalized thietanes **229** (Scheme 46).^255^ In contrast to previous visible-light thia-PB protocols, which relied on direct excitation of the thiocarbonyl partner and consequently displayed limited substrate scope, this methodology employs the unconventional, long-lived photocatalyst **Ir-6** (τ = 2.22 μs) to selectively sensitize the maleimide through triplet energy transfer. Photophysical studies, including phosphorescence and Stern–Volmer quenching experiments, supported a Dexter-type triplet energy transfer from **Ir-6** to the maleimide **227**, while excluding singlet–singlet energy transfer. Therefore, the proposed mechanism involves triplet sensitization of maleimide **227** to generate **227-T**_**1**_, which undergoes stepwise addition to the thiocarbonyl compound **228** to form a 1,4-biradical intermediate **230**. Subsequent intersystem crossing and radical recombination furnish the thietane product **229**.

**Scheme 46:**
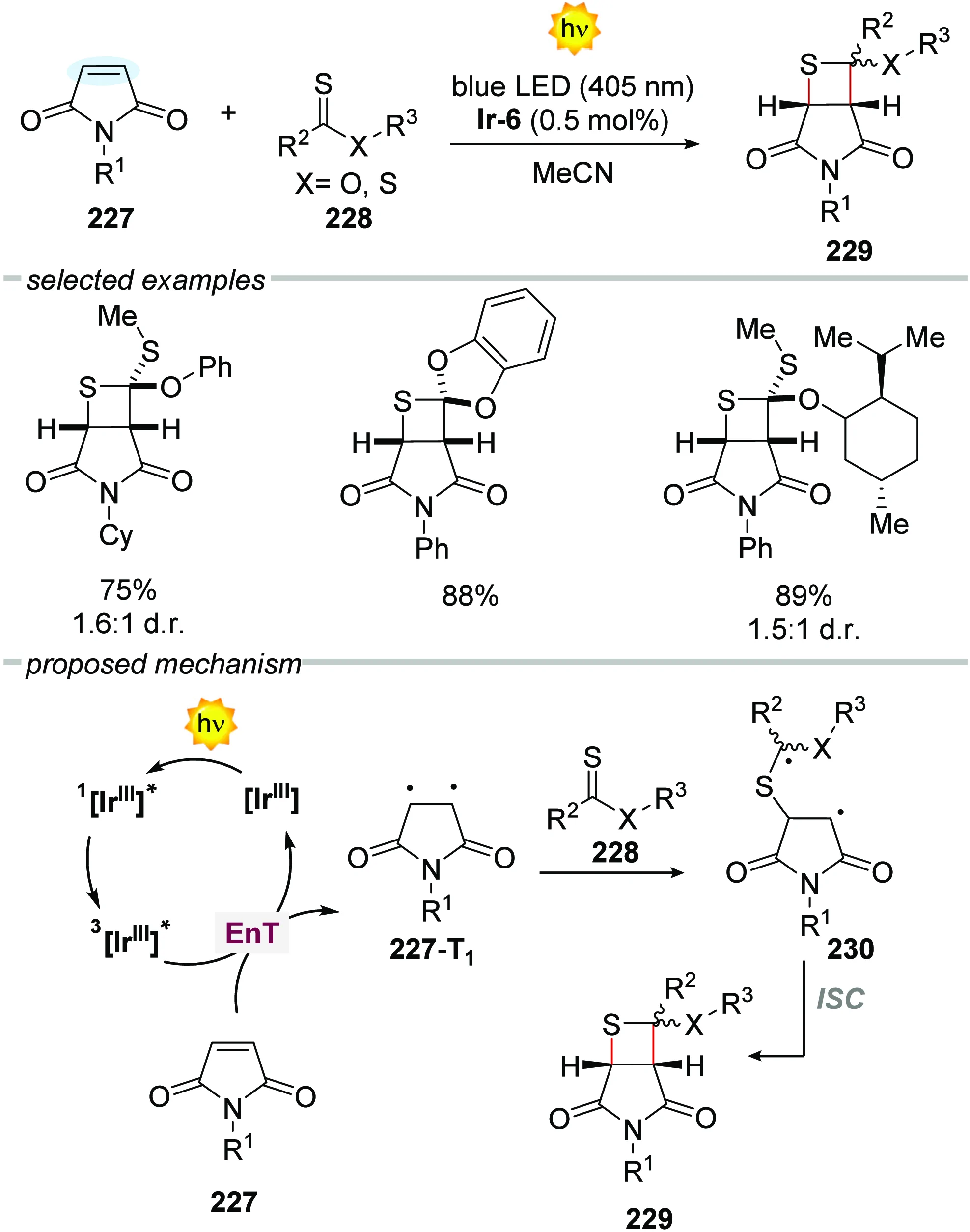
Intermolecular Photocatalytic Transposed Thia-PB Reaction of Maleimide with Xanthates

In line with their interest in the visible light photocatalytic dearomatization of indoles, the Zhong group reported a protocol for the synthesis of thietane-fused tetracyclic indolines.^256^ Particularly, indole-derived xanthates **231** were reacted in the presence of thioxanthone **THX** as triplet sensitizer under 405 nm irradiation providing access to various tetracyclic thietanes **232** (Scheme 47). Stern–Volmer quenching studies together with DFT calculations suggested that the reaction is driven by the sensitization of the xanthate moiety within **231** from the excited photocatalyst THX*. Then, C–S bond formation leads to the diradical **233** that undergoes ISC and radical coupling to give the thiane product **232**. For some substrates, undesired [2 + 2] photocycloaddition between thioxanthone and the indole led to the formation of side products. In these specific cases, the desired reactivity was restored by replacing thioxanthone with the **Ir-2** photocatalyst. Mechanistic studies suggested that, under these conditions, the reaction might proceed through triplet sensitization of the indole moiety.

**Scheme 47:**
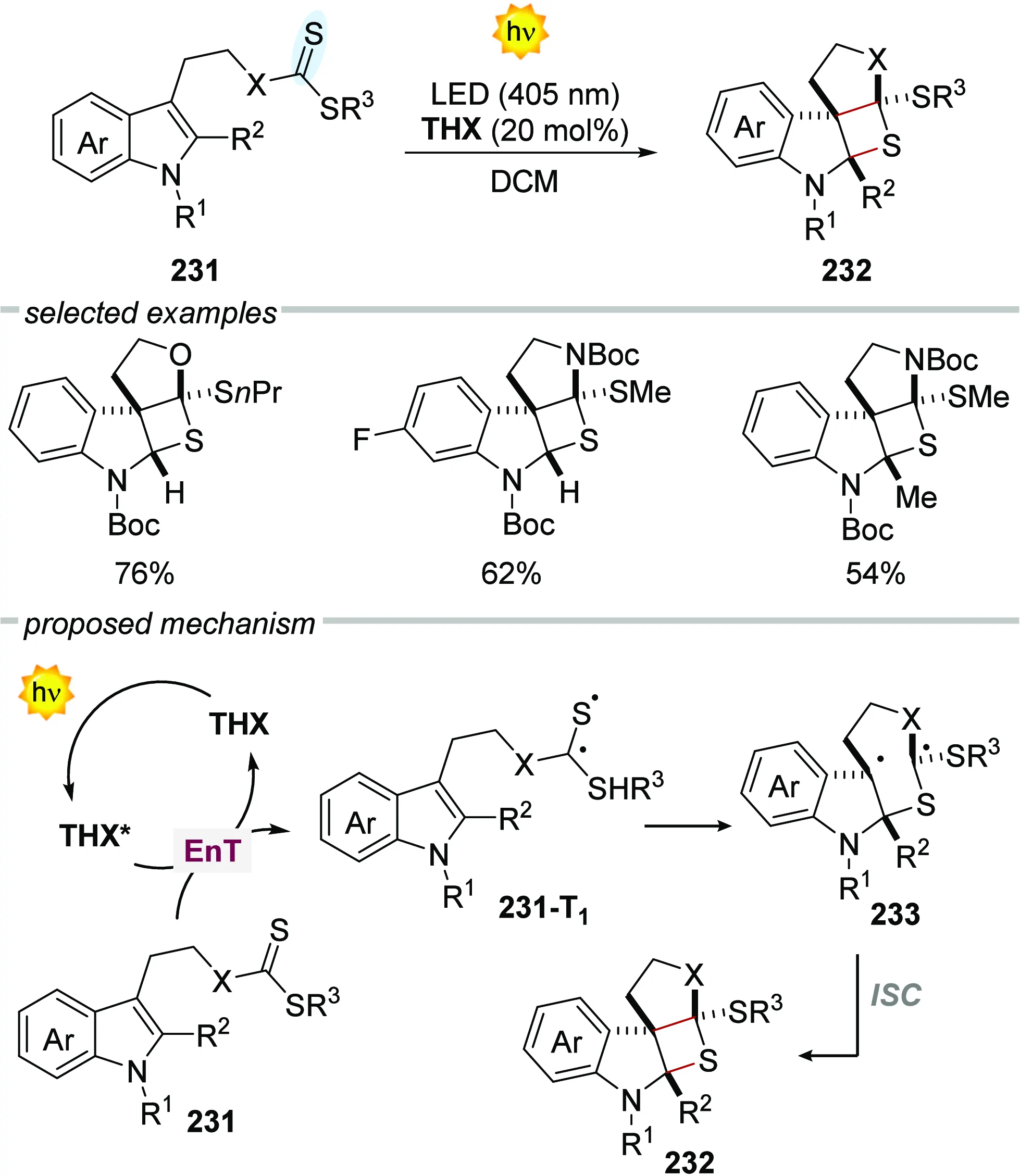
Intramolecular Photocatalytic Thia-PB Cycloaddition of Indole-Derived Xanthates

### 2.7 Sulfinamides

#### 2.7.1 Photocatalytic Methods

The application of triplet energy transfer (EnT) photocatalysis has recently been extended to *N*-silyl sulfinylamines, further broadening the scope of [2 + 2] photocycloaddition chemistry. In 2026, the groups of Willis and of Zhang and Yang independently reported that *N*-silyl sulfinylamines **234** undergo visible-light-mediated [2 + 2] cycloaddition with alkenes **186** upon sensitization by 4-CzIPN, providing a direct and modular route to β-cyclic sulfinamides **235** (Scheme 48).^257,258^ The methodology displays broad substrate scope, tolerating aryl, 1,1-disubstituted, and cyclic alkenes while consistently affording the products with *cis* diastereoselectivity.

**Scheme 48:**
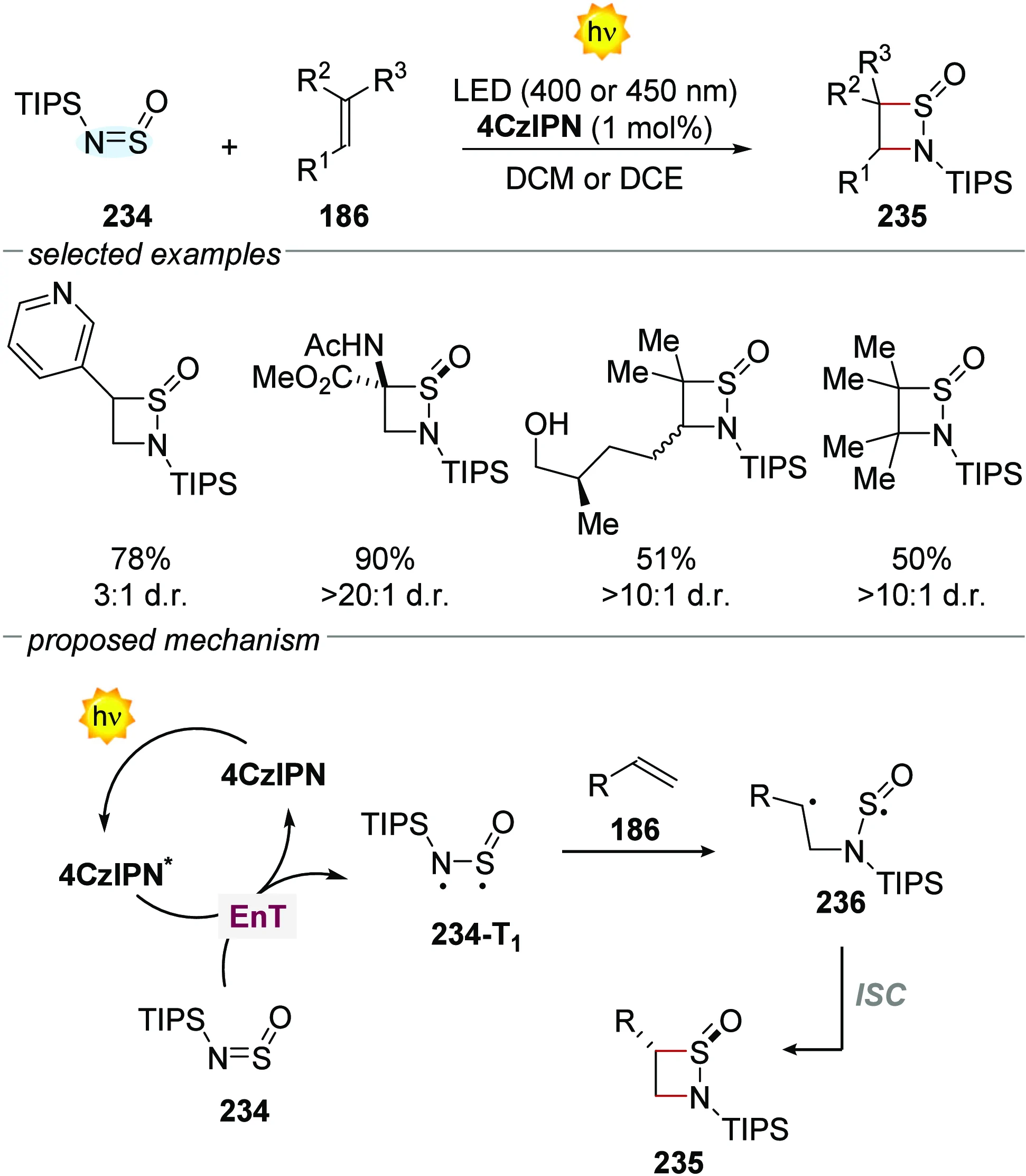
Intramolecular Transposed Paternò–Büchi Reaction of Atropoisomeric Enamides

Most importantly, β-cyclic sulfinamides proved to be versatile synthetic intermediates, provided unconventional routes to relevant heterocyclic compounds. Particularly, oxidation furnished the corresponding β-sultams, cyclic sulfonamides of synthetic and pharmacological relevance. In addition, imidation of β-sulfinamides gave the unprecedented cyclic monoaza variants sulfonimidamides. These transformations further highlight the potential of this methodology to access unexplored sulfur-containing heterocyclic scaffolds of relevance to medicinal chemistry.

A combination of experimental and computational studies supported a reaction mechanism initiated by the sensitization of *N*-silyl sulfinylamines **234**. Subsequent stepwise addition to the alkene **186** proceeds through initial C–N bond formation to generate a 1,4-diradical intermediate **236**, which undergoes intersystem crossing followed by radical recombination to afford sulfinamide **235**. Consistent with the observed regioselectivity, NBO analysis of the triplet-excited sulfinylamine revealed the highest spin density at the nitrogen atom. Computational studies further identified radical addition to the ground-state alkene as the rate-determining step, while the main orbital interaction involves the overlap between the SOMO localized on the N of **234-T**_**1**_ with the π orbital of the alkene. The observed *cis* selectivity was proposed to arise from hydrogen-bonding interactions during ring closure.

## 3 [2π+2σ]-Cycloadditions

Photochemical hetero-[2π+2σ] cycloadditions provide a strain-release approach to bridged heterocyclic frameworks through the combination of an unsaturated two-atom π-component with a two-atom σ-component. The latter is most commonly represented by the highly strained central C–C bond of bicyclo[1.1.0]­butanes (BCBs) or related small bicyclic systems.^259,260^ Cleavage of this σ-bond and formation of two new bonds with the π-component enable the rapid construction of oxygen-, nitrogen-, or sulfur-containing bridged scaffolds, including heterobicyclo[2.1.1]­hexanes and related polycyclic structures (Scheme 49).^48^ These conformationally rigid, *sp* ^3^-rich frameworks have recently attracted considerable interest as three-dimensional building blocks and potential bioisosteres of aromatic or heteroaromatic rings. Accordingly, photochemical hetero-[2π+2σ] cycloadditions have rapidly developed into a valuable strategy for accessing structural motifs that are often challenging to prepare through conventional ground-state methods.

**Scheme 49:**
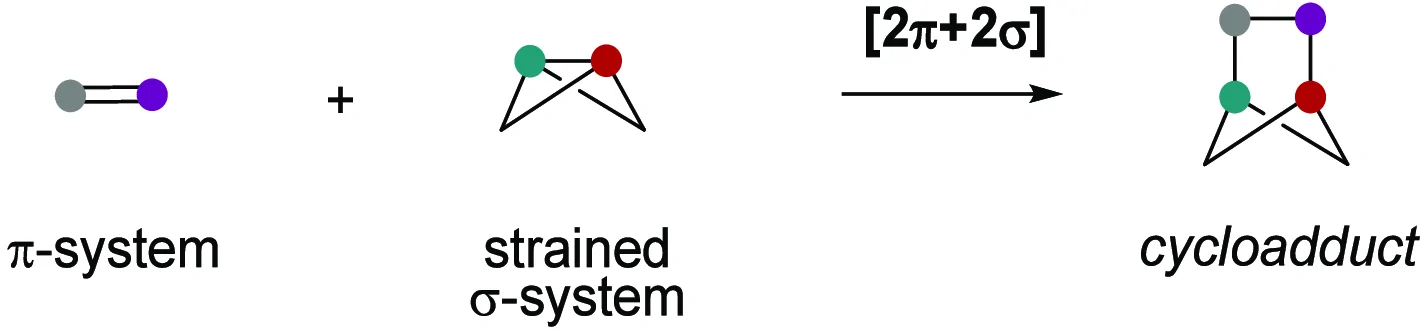
General [2π+2σ] Cycloaddition between a π-System and a Strained σ-System

The mechanistic scenario of these transformations is diverse, and the designation [2π+2σ] generally describes the net bond reorganization rather than implying a concerted process.^261^ In a common activation mode, direct excitation or triplet sensitization of the heteroatom-containing π-component generates an excited-state species that adds stepwise across the strained σ-bond. The resulting triplet diradical commonly undergoes intersystem crossing and radical recombination to furnish the bridged cycloadduct.^262^ Alternatively, photoredox catalysis enables the activation of the strained component through single-electron oxidation or reduction, thus generating radical-ion or ring-opened cyclobutyl intermediates. Those species are subsequently trapped by the heteroatom-containing reaction partner and undergo ring closure. Cooperative metal catalysis, hydrogen-atom transfer, group migration, and secondary ring-opening or ring-closing events may further modify these elementary pathways, accounting for the considerable structural and mechanistic diversity observed within this reaction class.^261^

Within this framework, the present section focuses on photochemical hetero-[2π+2σ] processes involving BCBs or related strained σ-components, in which the heteroatom-containing π-partner participates directly in construction of the bridged cycloadduct. Both intra- and intermolecular examples are considered. Reactions in which the primary hetero-[2π+2σ] cycloadduct undergoes subsequent rearrangement, fragmentation, ring opening, or skeletal reconstruction are also included when the connectivity of the initial cycloaddition event remains identifiable. The material is organized according to the heteroatom-containing bridged scaffold generated or implicated in the primary strain-release event, with each class further subdivided by activation mode, including direct excitation, photoredox single-electron transfer, and photosensitized energy transfer.

### 3.1 Oxa-bicyclic Scaffolds

#### 3.1.1 2-Oxabicyclo­[2.1.1]­hexanes

##### 3.1.1.1 Direct Irradiation Methods

In 2024, Glorius, Houk and co-workers introduced a catalyst-free double strain-release [2π+2σ]-photocycloaddition between cyclobutenones **237** and bicyclo[1.1.0]­butanes **238** (BCBs) under direct visible-light irradiation (Scheme 50).^263^ In this strategy, cyclobutenones **238** act as photochemical ketene **240** precursors, while BCBs provide the strained σ-bond component, enabling access to 2-oxabicyclo[2.1.1]­hexanes (oxaBCH) **239** bioisosteres under blue LEDs irradiation (λ = 400 nm). The reaction exploits the divergent reactivity of the two orthogonal π-systems of the transient ketene, with selected BCB substitution patterns favoring cycloaddition through the ketene C=O unit to give oxaBCH-type **239** products. The method tolerated a range of aromatic and heteroaromatic cyclobutenones, as well as functionalized BCBs bearing amide, ester, ketone, alkynyl, and chiral alcohol-derived substituents. Mechanistic studies supported a two-photon sequence: initial excitation of the cyclobutenone **237** promotes electrocyclic ring opening to the ketene **240** to the corresponding 1,4-biradical, which was experimentally trapped with allylamine, while a second excitation event enables the [2π+2σ] cycloaddition. The reduced yield observed in the presence of β-ionone, together with the low quantum yield and DFT calculations, supported a triplet pathway involving BCB σ-bond cleavage, ketene–BCB diradical formation **241**, and radical recombination as the selectivity-determining stage.

**Scheme 50:**
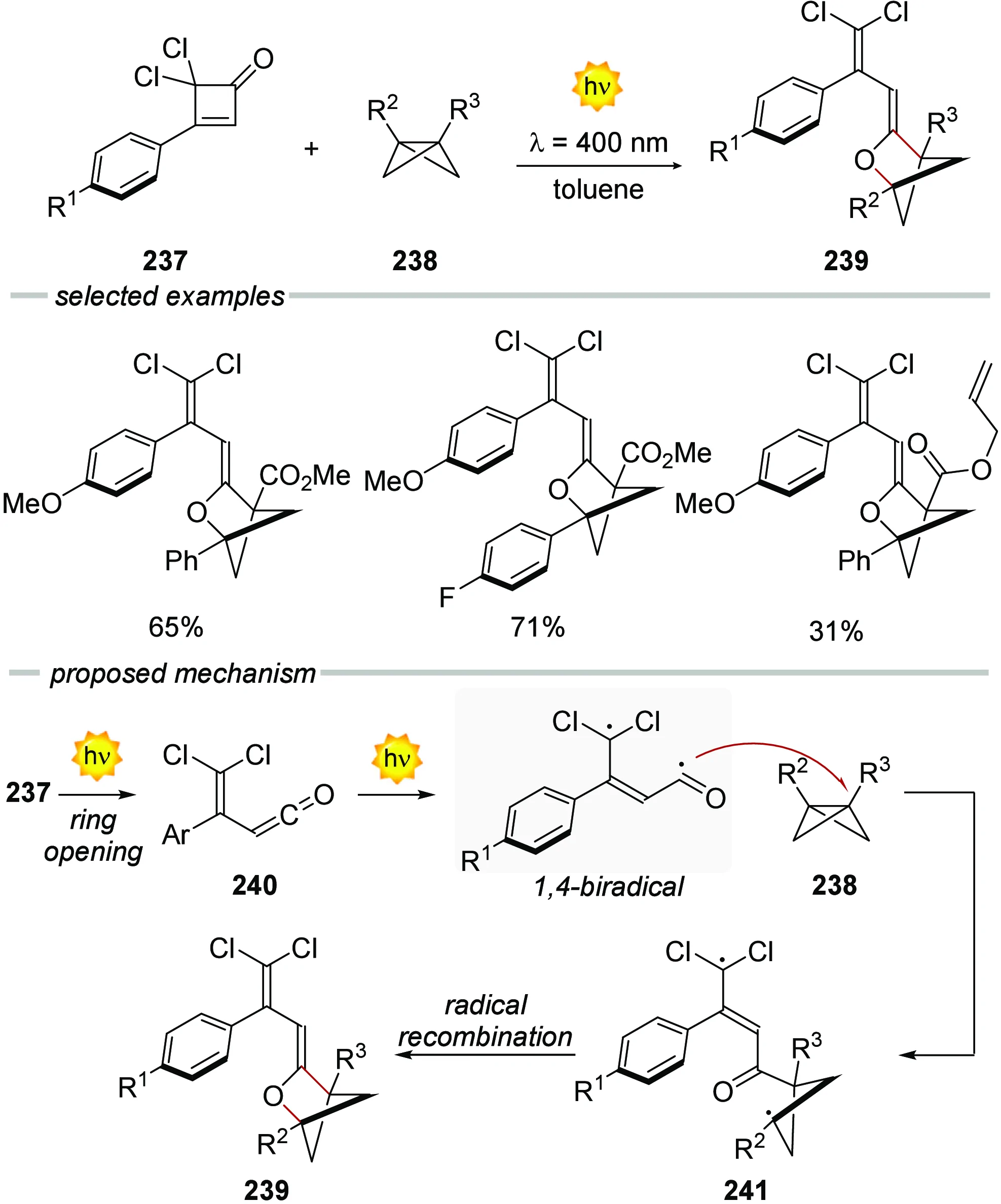
Catalyst-Free Double Strain-Release Photocycloaddition of Cyclobutenone-Derived Ketenes with BCBs To Access oxaBCH Scaffolds

##### 3.1.1.2 Photocatalytic Methods

Walker, Crespi and co-workers later reported a complementary photoredox strategy based on the oxidative activation of BCBs.^264^ Using a strongly oxidizing acridinium photocatalyst **Acr-1** under blue LED irradiation (λ = 456 nm), ketone- and ester-substituted BCBs **243** could be engaged in [2π + 2σ] cycloadditions with aldehydes **242** to afford oxaBCH products **244** (Scheme 51). In the hetero-cycloaddition manifold, benzaldehyde and substituted benzaldehydes were competent partners, while ethyl glyoxylate and differently substituted BCBs also provided access to oxygen-containing bicyclo[2.1.1]­hexane scaffolds. Mechanistic studies supported a photoredox oxidative pathway in which the excited acridinium catalyst oxidizes the BCB, rather than the aldehyde, to generate a BCB radical cation. Subsequent trapping by the aldehyde gives a cationic radical intermediate that is reduced by the acridinium radical and closes to the oxaBCH product. Cyclic voltammetry, Stern–Volmer studies, UV/vis spectroscopy, radical trapping experiments, and DFT calculations supported this oxidative activation mode and highlighted how subtle changes in BCB and aldehyde electronics influence the observed regioselectivity.

**Scheme 51:**
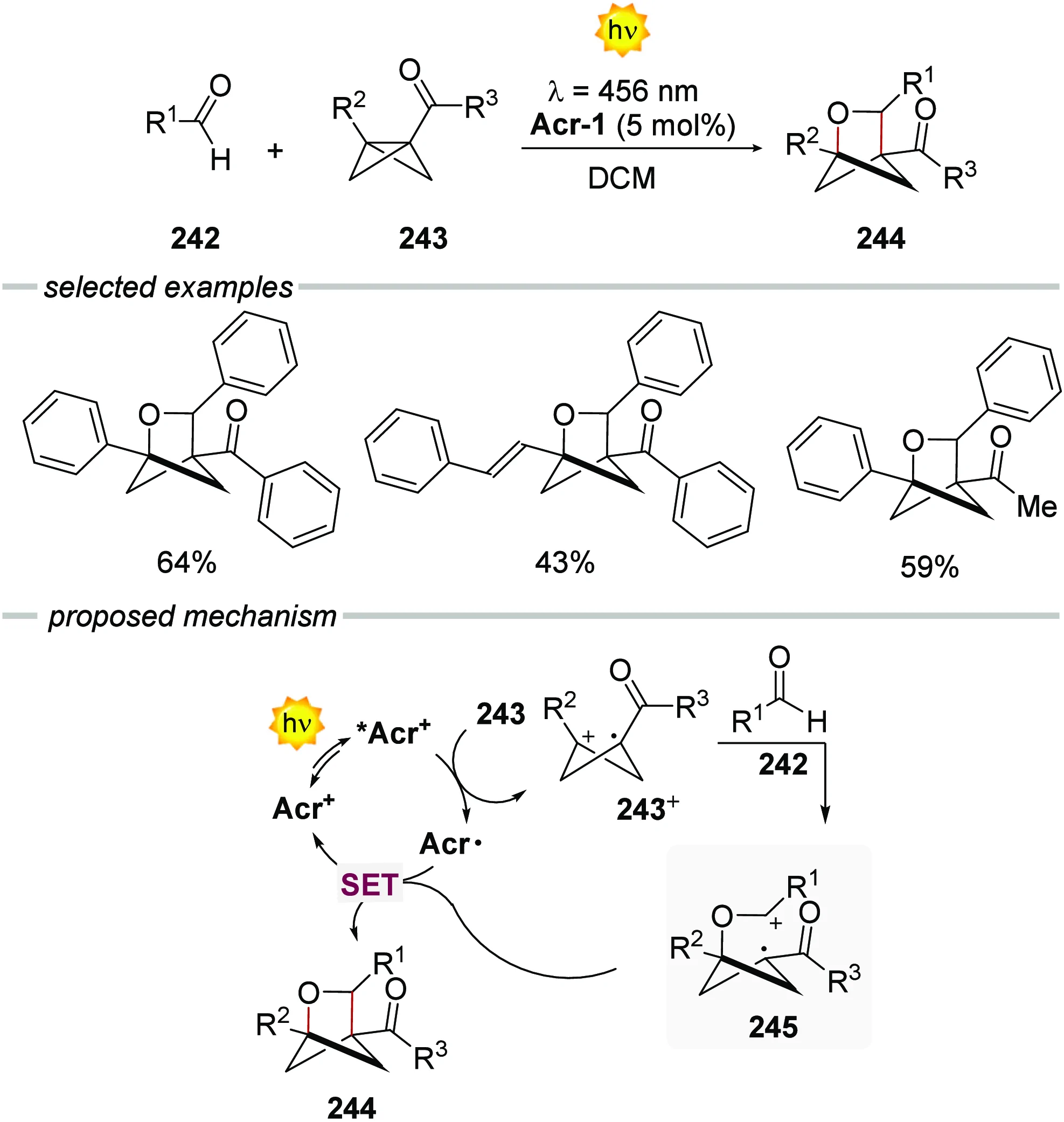
Photoredox Oxidative Activation of BCBs for [2π+2σ] Cycloadditions with Aldehydes

Building on oxidative BCB activation, Huang and co-workers reported a cobalt-enhanced photocatalytic [2π+2σ] cycloaddition of BCBs **246** with aldehydes **242** for the synthesis of oxaBCHs **247** (Scheme 52).^265^ Under blue LED irradiation at 450 nm, a strongly oxidizing **Acr-1** in combination with CoCl_2_ enabled the coupling of ester-substituted BCBs **246** with both aliphatic and aromatic aldehydes **242**, generally improving the efficiency relative to the photocatalyst-only variant. The scope included linear, branched, cyclic, and functionalized aliphatic aldehydes, as well as aromatic aldehydes bearing electron-donating and electron-withdrawing substituents. BCBs **246** bearing substituted aryl groups and complex ester fragments, including menthol- and pregnenolone-derived units, were also tolerated. Mechanistic experiments supported an oxidative radical-polar pathway: the excited **Acr-1** oxidizes the BCB **246** to the corresponding radical cation **246** ^**+•**^, while cobalt promotes the formation of a nucleophilic ring-opened intermediate **248** that undergoes Reformatsky-type addition to the aldehyde **242**, followed by ring closure to give the oxaBCH product **246**. Radical trapping, oxygen and methanol capture experiments, Stern–Volmer and UV–vis analyses, and a low quantum yield supported the involvement of BCB radical cations and disfavored a radical-chain mechanism.

**Scheme 52:**
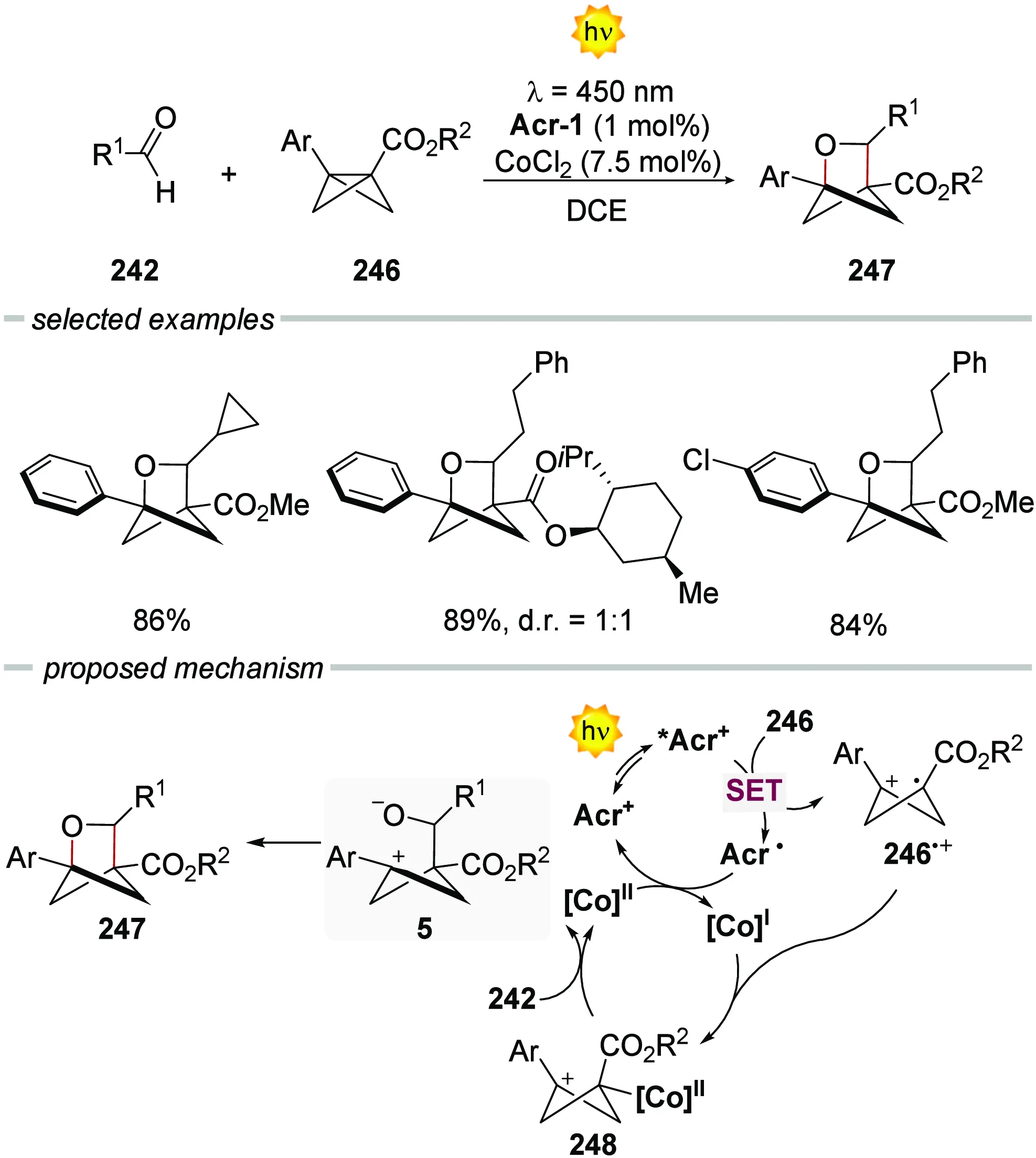
Dual Cobalt-Photoredox Synthesis of oxaBCHs from BCBs and Aldehydes

In 2022, Glorius and co-workers reported an energy-transfer-enabled synthesis of polysubstituted oxaBCHs **250** from benzoylformate esters **249** and BCBs **246** (Scheme 53).^266^ Under blue LED irradiation (λ = 450 nm), the high-triplet-energy iridium photocatalyst **Ir-2** promoted the reaction of benzoylformate esters **249** with substituted BCBs **246**, providing access to C1-, C2-, and C3-substituted oxaBCH scaffolds **250**. The method tolerated different ester groups on the α-ketoester component, electronically diverse aryl and heteroaryl benzoylformates, and several aryl- and ester-substituted BCBs, although monosubstituted BCBs were not competent substrates. Mechanistic studies supported a triplet energy-transfer pathway rather than a photoredox or EDA-complex process: Stern–Volmer experiments showed substrate quenching of the excited photocatalyst, while cyclic voltammetry and UV–vis studies argued against SET and donor–acceptor complex formation. Direct UV excitation of the benzoylformate ester also afforded the product, and an ortho-methyl substrate gave a Norrish type II product, supporting the involvement of excited benzoylformate species. The proposed mechanism involves triplet benzoylformate **249-T**_**1**_ addition to the BCB **246** σ-bond, intersystem crossing and radical recombination, followed by backbone C–H abstraction, aryl migration, and final HAT from **254** to deliver the polysubstituted oxaBCH product **250**.

**Scheme 53:**
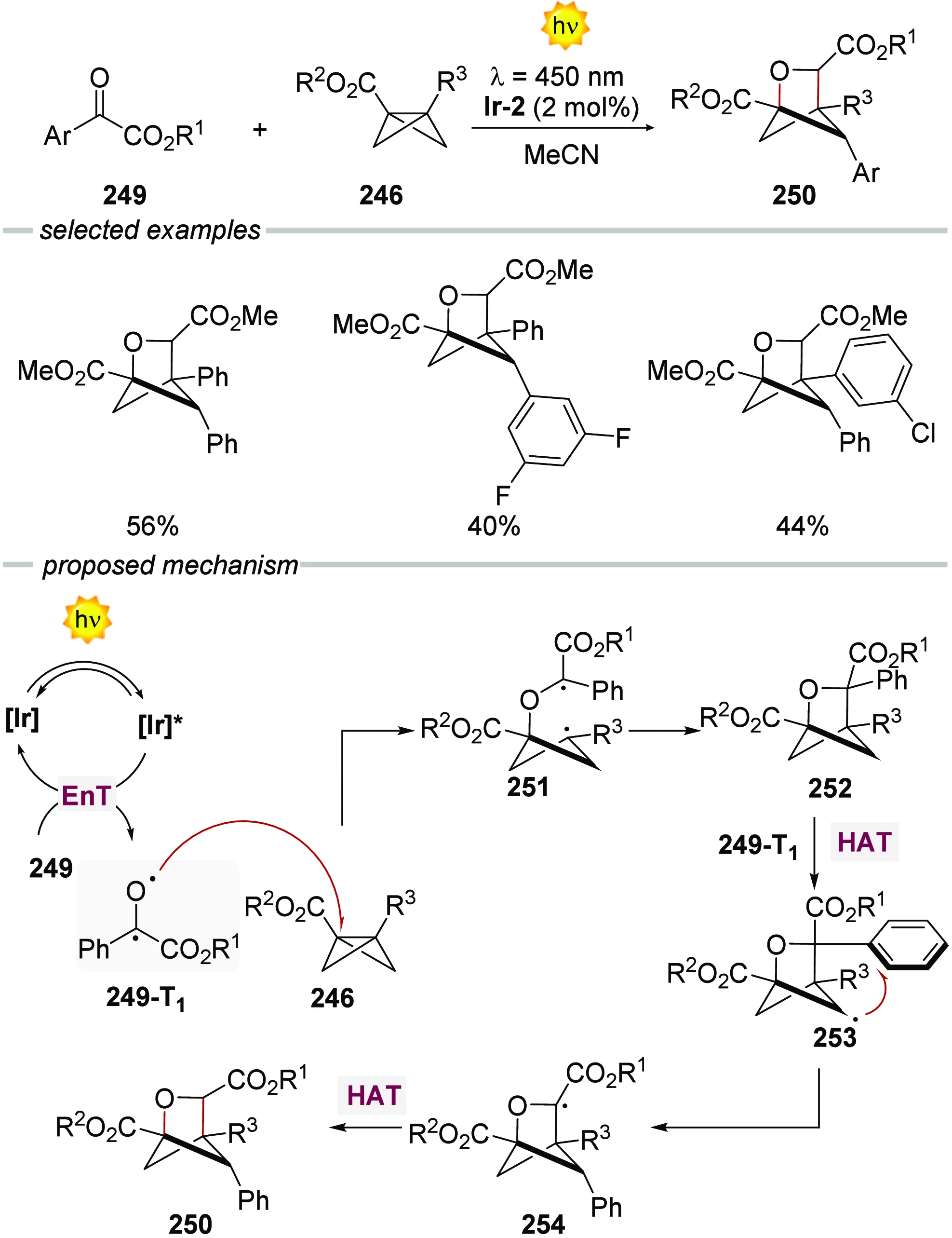
Energy-Transfer-Enabled Synthesis of Polysubstituted oxaBCHs from Benzoylformates and BCBs

#### 3.1.2 Other *O*-Containing Bridged Bicyclic Scaffolds

##### 3.1.2.1 Photocatalytic Methods

A more recent and mechanistically distinct EnT-enabled strategy further expanded this area beyond direct intermolecular BCB cycloadditions. Hong and co-workers recently described a visible-light-driven reconstruction of BCBs into oxygenated bicyclic scaffolds through a programmed ring-open/ring-close sequence.^267^ In this process, pyridine *N*-oxides serve as oxene surrogates under photocatalytic conditions, promoting selective oxygenative opening of ketone-substituted BCBs to β,γ-unsaturated 1,2-diketones. These intermediates are then intercepted in situ through an intramolecular PB [2 + 2] cycloaddition to afford 5-oxa-2-oxobicyclo[2.1.1]­hexanes. Although either carbonyl group can initially engage in the photocycloaddition, reversible retro-[2 + 2] equilibration funnels the reaction toward the productive 5-oxa-BCHex products. Mechanistic experiments supported a triplet energy-transfer pathway for the oxygenative ring-opening step and showed that photocatalysis also accelerates the subsequent PB process.

### 3.2 Aza-bicyclic Scaffolds

#### 3.2.1 1-Azabicyclo­[n.1.1]­alkanes

##### 3.2.1.1 Direct Irradiation Methods

Lu and co-workers reported a catalyst-free, visible-light-induced [2π+2σ] photocycloaddition of 4-azacoumarins with BCBs for the synthesis of azabicyclo[2.1.1]­hexanes (aza-BCHs).^268^ In contrast to photosensitized EnT variants, the reaction relies on direct excitation of the imine chromophore, enabled by the ester-substituted 4-azacoumarin scaffold, and proceeds under blue-light irradiation without an external photocatalyst. Both mono- and disubstituted BCBs were competent partners, with the latter displaying reversed regioselectivity depending on radical-intermediate stability. Mechanistic experiments, including inhibition by triplet quenchers and O_2_, exclusion of donor–acceptor complex formation, and stereochemical probes with β-methylstyrene, supported a pathway occurring primarily on the triplet hypersurface.

##### 3.2.1.2 Photocatalytic Methods

Huang and co-workers further expanded strain-release heterophotocycloaddition chemistry to azabicyclo[1.1.0]­butanes (ABBs) **257**, developing a visible-light-driven palladium-catalyzed coupling with 1,3-dienes **255–256** (Scheme 54).^269^ Under irradiation at 450 nm, Pd­(PPh_3_)_4_ in combination with Xantphos, LiBr, and K_2_CO_3_ enabled the synthesis of 1-azabicyclo[2.1.1]­hexanes **258**, formally engaging the strained σ-bond of the ABB **257** and one π-bond of the diene **255–256**. The method tolerated aryl-, heteroaryl-, linear alkyl-, and cyclic alkyl-substituted ABBs, as well as electronically diverse 1-aryl-1,3-dienes, heteroaryl dienes, fused aryl dienes, and alkyl-substituted dienes. Notably, changing the diene substitution pattern together with the ligand/halide system redirected the reaction toward larger 1-azabicyclo[4.1.1]­octene products **259**, highlighting the divergent nature of the platform. Mechanistic studies supported a photoinduced palladium radical-polar crossover pathway: halide opening of the ABB **257** generates an alkyl bromide or iodide intermediate **260**, which is reduced by photoexcited Pd(0) to an α-carbonyl radical **261**; addition to the diene **255–256**, capture by Pd­(I), and intramolecular nitrogen-centered cyclization then furnish the aza-bicyclic products **258–259**. Radical trapping, cyclopropyl radical-clock experiments, EPR analysis, Stern–Volmer studies, and DFT calculations supported the proposed open-shell mechanism and rationalized the ligand- and diene-controlled divergence.

**Scheme 54:**
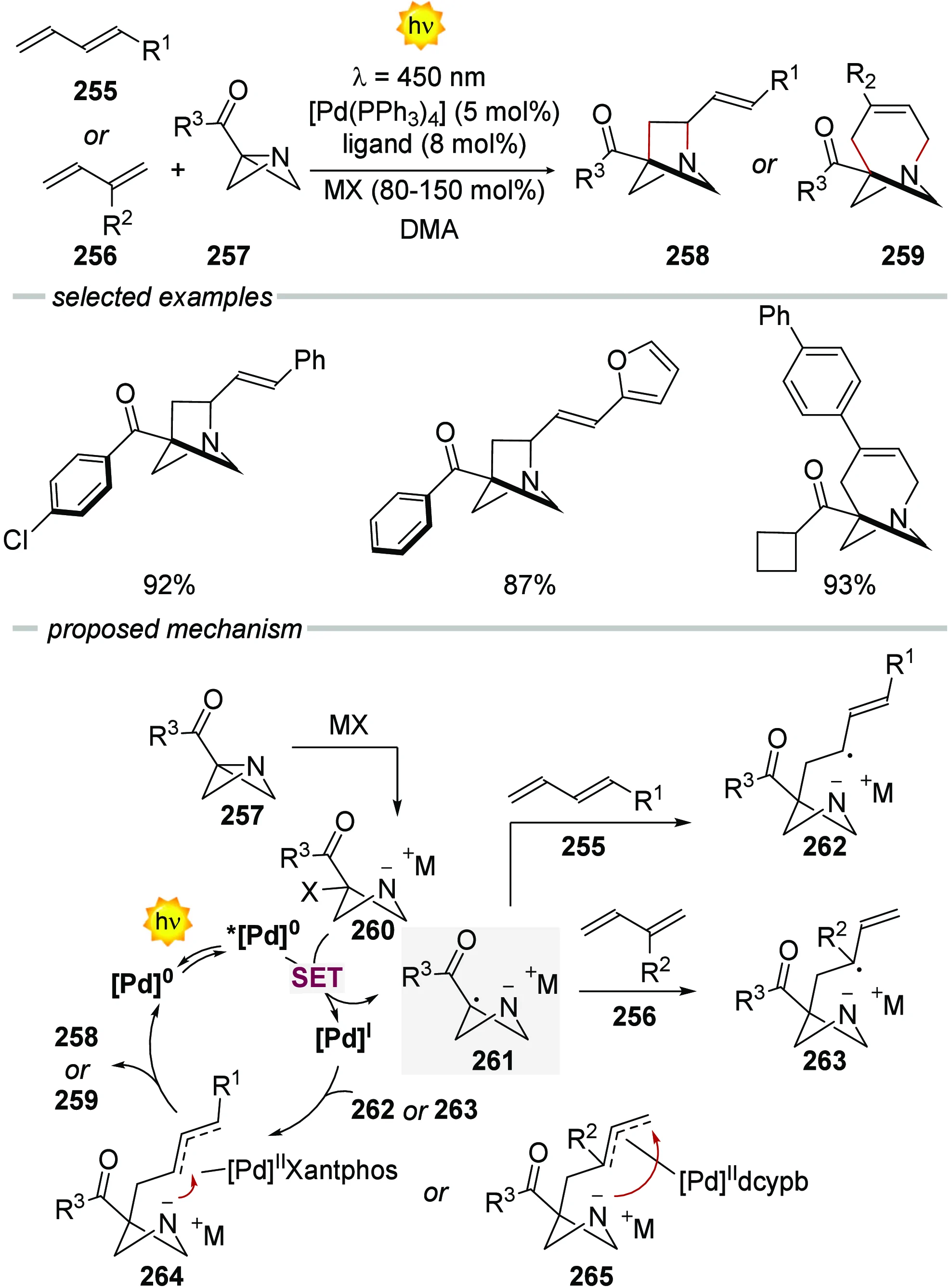
Photoinduced Palladium-Catalyzed Divergent Synthesis of Aza-Bicyclic Scaffolds from ABBs and Dienes

Guo, Duan and co-workers reported a substrate-regulated EnT strategy for the addition of *N*-sulfonyl ketimines **156** to BCBs **238** (Scheme 55).^270^ In the hetero-[2π+2σ] manifold, saccharin-derived cyclic ketimines **156** reacted with substituted BCBs **238** under purple light irradiation (λ = 390 nm) using thioxanthone (**THX**) as triplet photosensitizer, providing benzosultam-fused aza-BCHs **266** with good yields and high regioselectivity. The reaction tolerated 1,3-disubstituted BCBs bearing electronically diverse aryl groups, different ester substituents, amides, naphthoyl groups, and more complex alcohol-derived fragments, while variation of the cyclic ketimine component allowed aryl, heteroaryl, alkyl, and hydrogen substituents at the imine carbon. Mechanistic studies supported an energy-transfer pathway: the reaction required continuous light irradiation, the yield correlated with the triplet energy of the photosensitizer, and a triplet quencher strongly inhibited product formation. The proposed mechanism involves sensitization of the cyclic ketimine **156** to its triplet state **156-T**_**1**_, stepwise addition to the BCB **238** σ-bond to form a triplet diradical **267**, and intersystem crossing/radical recombination to afford the aza-BCH product **266**, with regioselectivity governed by the stability of the competing radical intermediates.

**Scheme 55:**
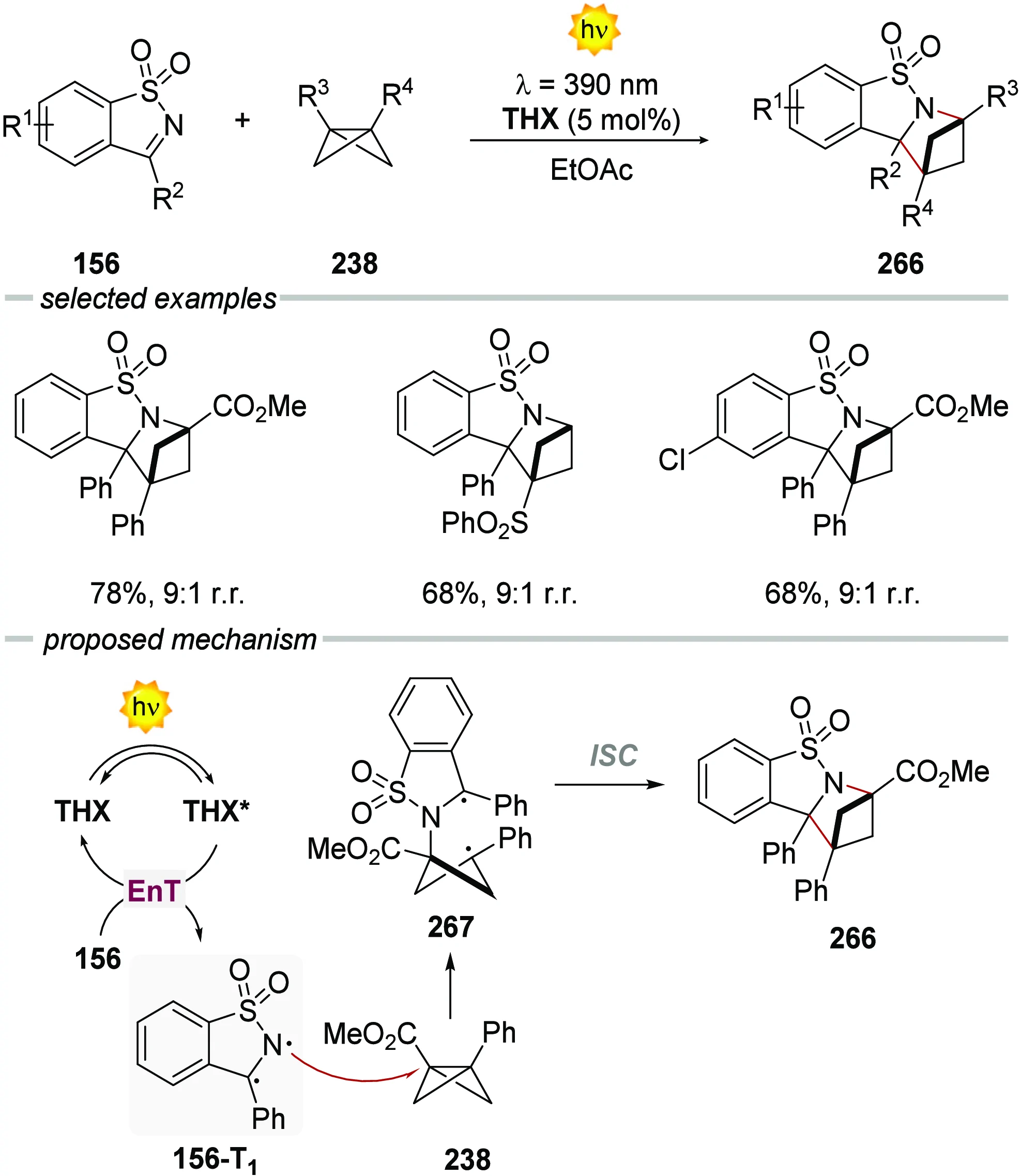
Energy-Transfer-Enabled Addition of Cyclic Ketimines to BCBs for the Synthesis of Benzosultam-Fused aza-BCHs

#### 3.2.2 2-Azanorbornanes and Related N-Containing Scaffolds

##### 3.2.2.1 Direct Irradiation Methods

In a related enantioselective study, Bach and co-workers developed chiral-template-controlled [2π+2σ] photocycloadditions of 2­(1*H*)-quinolones with BCBs.^271^ Although the main quinolone scope falls outside the photochemical hetero-cycloaddition definition adopted here, the authors also disclosed two aza variants with quinoxalinones, providing highly enantioenriched azetidine-containing products through direct irradiation in the presence of a recoverable hydrogen-bonding template.

##### 3.2.2.2 Photocatalytic Methods

Brown, Fessard and co-workers reported an energy-transfer-initiated strain-release strategy for the synthesis of 2-azanorbornanes **270** (Scheme 56).^272^ Although the study also included [2π+2σ] cycloadditions of azahousanes with alkenes, the heterophotocycloaddition-relevant manifold involves the reaction of naphthyl ketone-substituted housanes **269** with electrophilic imines **268** under blue light irradiation (λ = 450 nm). In this variant, α-imino glyoxylates **268** served as competent C=N π-components, providing polysubstituted 2-azanorbornanes **270** through [2π+2σ] cycloaddition. The scope was relatively focused but tolerated variation of the imine ester group and amide derivatives, while less electrophilic imines were not suitable partners. Mechanistically, the naphthyl ketone unit acts as an energy-transfer antenna: sensitization by an external triplet photosensitizer, more efficiently than direct UV excitation, promotes intersystem crossing and homolysis of the strained C–C bond to generate a triplet diradical **271**, which adds to the imine and undergoes cyclization to form the nitrogen-containing bicyclic scaffold.

**Scheme 56:**
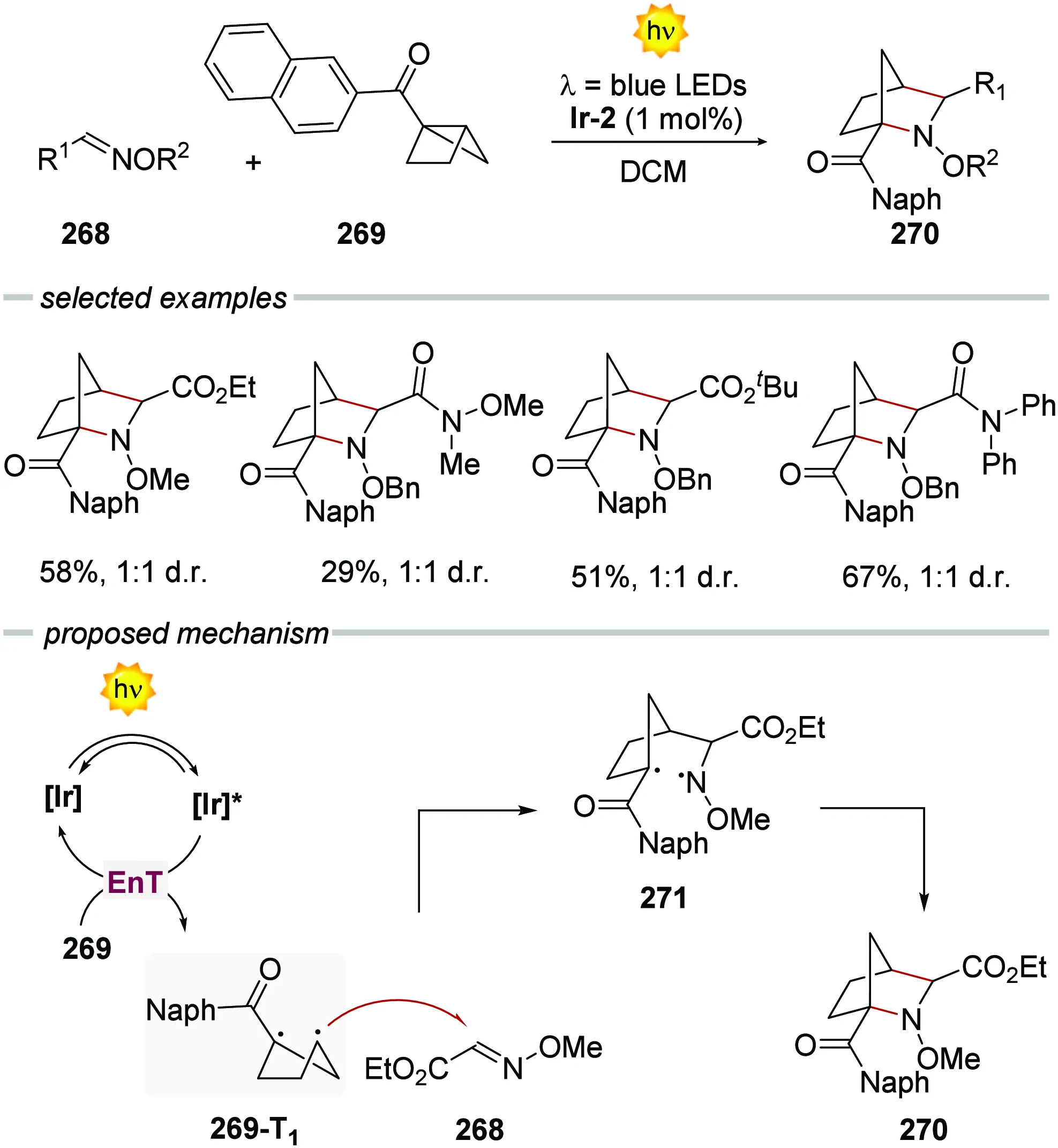
Energy-Transfer-Mediated [2π+2σ] Cycloaddition of Imines with Strained Housanes To Access 2-Azanorbornanes

### 3.3 S-Containing Ring-Expanded Scaffolds

#### 3.3.1 Thiophene-Derived Ring-Expanded Scaffolds

##### 3.3.1.1 Photocatalytic Methods

In 2023, Glorius, Houk and co-workers reported a photoredox dearomative ring expansion of thiophenes **272** through BCB **273** insertion.^273^ Under blue LED irradiation (λ = 450 nm), an acridinium photocatalyst **Acr-2** enabled the conversion of thiophenes and benzothiophenes **272** into eight-membered sulfur-containing bicyclic scaffolds **274** under mild conditions (Scheme 57). The reaction afforded the ring-expanded products **274** in high yield and regioselectivity, and the method tolerated diverse BCBs bearing amide, ester, ketone, and ether groups, as well as substituted thiophenes and benzothiophenes containing halides, carbonyl groups, heteroaryl substituents, alcohols, alkenes, alkynes, and chiral fragments. Mechanistically, Stern–Volmer and cyclic voltammetry studies supported selective oxidation of the thiophene partner **272** by the excited acridinium catalyst, rather than direct oxidation of the BCB. The resulting thiophene radical cation **272** ^**+•**^ undergoes regioselective attack by the strained BCB, followed by C–S bond formation, reduction, and C–S bond cleavage to deliver the dearomatized ring-expanded product **274**. The inhibition observed with TEMPO, a quantum yield below unity, and DFT calculations further supported a photoredox radical pathway, with spin-density distribution in the heteroarene radical cation accounting for the different regioselectivity observed with thiophenes and benzothiophenes.

**Scheme 57:**
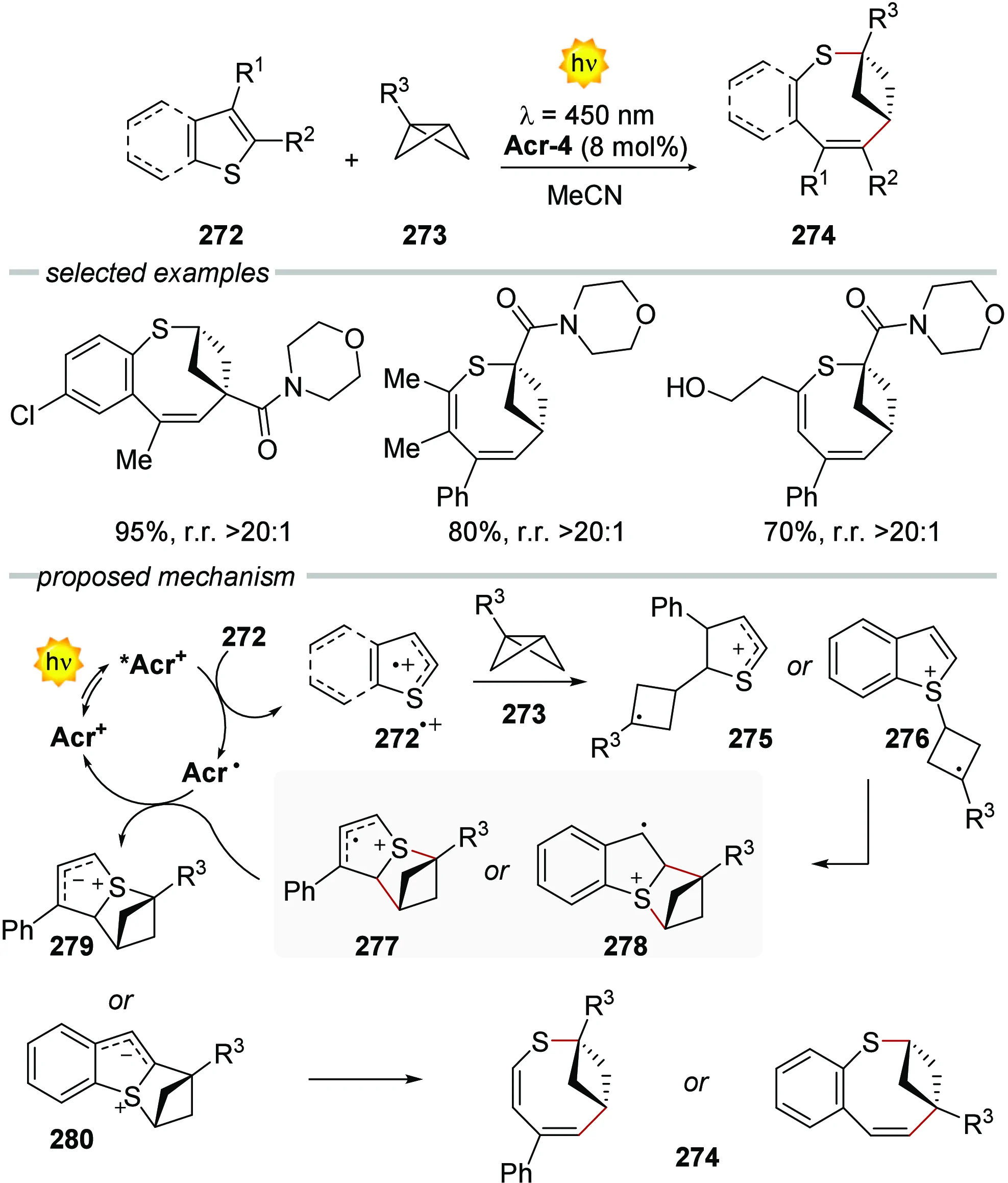
Photoredox Dearomative Ring Expansion of Thiophenes and Benzothiophenes through BCB Insertion

Glorius and co-workers in 2025 used a data-driven strategy to identify energy-transfer-catalyzed dearomative cycloadditions of challenging monocyclic heteroarenes (Scheme 58).^274^ In the [2π+2σ] manifold, substituted thiophenes **281** were engaged with BCBs **238** under blue light irradiation (λ = 450 nm) using **Ir-4** as PC, providing 6-thiatricyclo­[5.1.1.0^2,5^]­non-3-ene **282** scaffolds through a dearomative cycloaddition/rearrangement cascade. The method was particularly effective for benzoyl- and aryl-substituted thiophenes, tolerating methyl, methoxy, silyl, halogen, amide, cyano, trifluoromethyl, ester, and pyridyl substituents, as well as more complex thiophene derivatives. Mechanistic studies supported an EnT rather than SET pathway: the Ir PC is the absorbing species at 450 nm, the thiophene **281** quenches the excited catalyst, SET is thermodynamically disfavored, and the low quantum yield excludes a radical-chain process. The proposed pathway involves triplet sensitization of the thiophene **281**, regioselective addition to the BCB **238** to form a triplet diradical **283-T**_**1**_, C–S bond cleavage and following radical recombination to a diene intermediate **285**. Lastly, photochemical disrotatory 4π-electrocyclization delivers to the final sulfur-containing tricyclic product **282**.

**Scheme 58:**
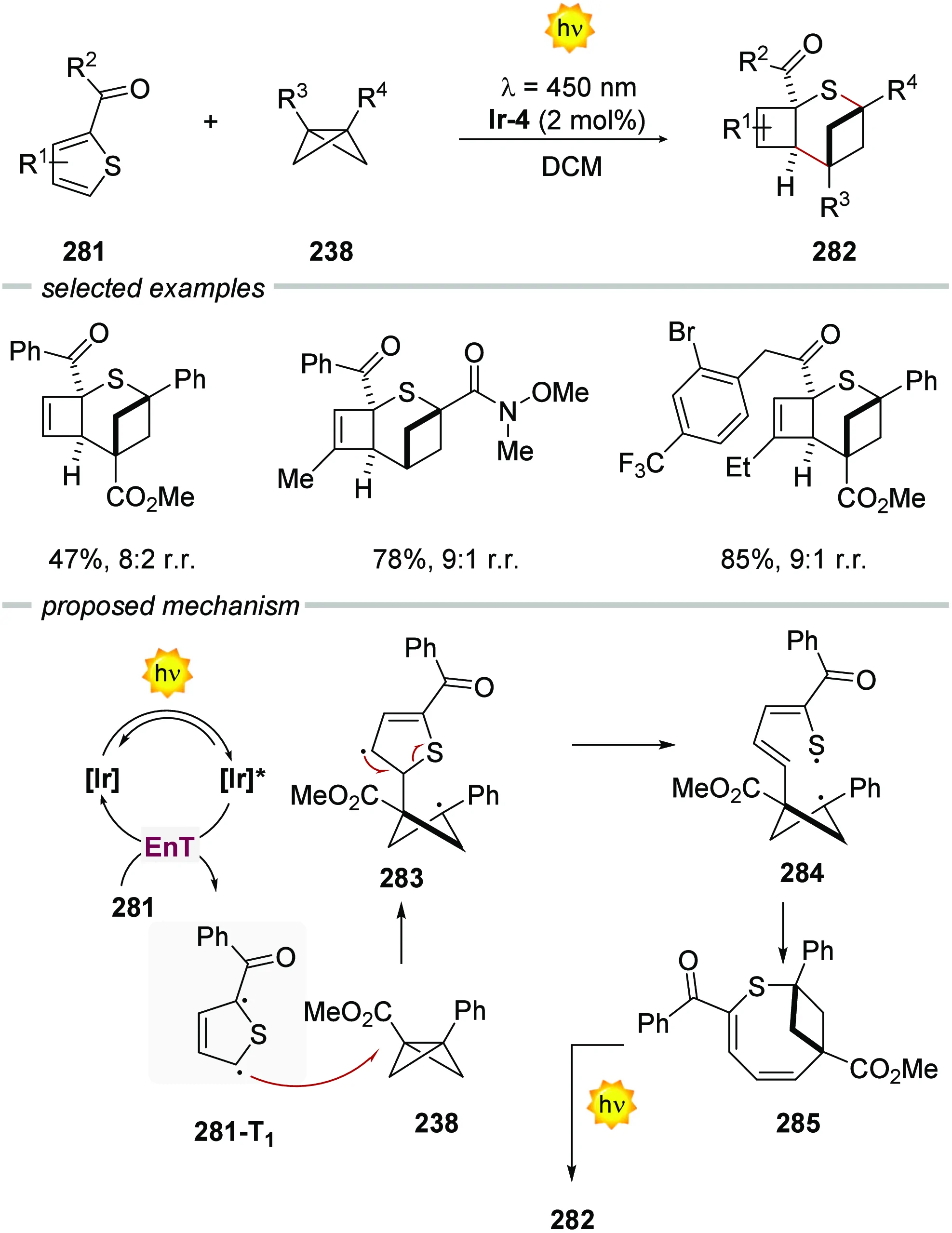
Energy-Transfer-Catalyzed Dearomative Cycloaddition of Thiophenes with BCBs To Form Sulfur-Containing Tricyclic Scaffolds

#### 3.3.2 Dithianes and Related Sulfur-Centered Ring Expansions

##### 3.3.2.1 Photocatalytic Methods

A conceptually related, although non-BCB-based, thia-[2π+2σ] manifold was recently reported by Boddaert, Ciofini and co-workers in the visible-light-induced ring enlargement of thietanes with thiocarbonyl compounds.^275^ In this transformation, thiocarbonyls generated in situ by Norrish-type II fragmentation of pyrenacyl sulfides engage thietanes to afford regioselectively either 1,2- or 1,3-dithianes, depending on whether thioketones or thioaldehydes are involved. From a formal electronic perspective, the process can be viewed as the insertion of a C = S π-component into a strained C–S σ-bond of the thietane, thereby constituting an unusual sulfur-centered hetero-[2π+2σ] ring expansion. Mechanistic experiments and TD-DFT calculations supported the involvement of triplet-excited thiocarbonyl species, generated predominantly through energy transfer from 1-acetylpyrene, followed by S–S bond formation, thietane ring opening, intersystem crossing, and radical recombination to deliver the dithiane products.

## 4 (3 + 2)-Hetero-cycloadditions

The (3 + 2)-cycloaddition reaction constitutes a versatile and efficient approach for assembling five-membered heterocycles. Within the polar regime, this transformation proceeds through the interaction of a 1,3-dipole with an unsaturated partner, commonly referred to as a dipolarophile. The process involves a cyclic redistribution of electron density, leading to the formation of a five-membered ring accompanied by neutralization of the formal charges (Scheme 59). Although the notion of 1,3-dipolar cycloadditions was first proposed by Smith in 1938,^276^ it was the seminal contributions of Rolf Huisgen and co-workers in the 1960s that established the conceptual framework and demonstrated the broad synthetic utility of this reaction.^277^

**Scheme 59:**
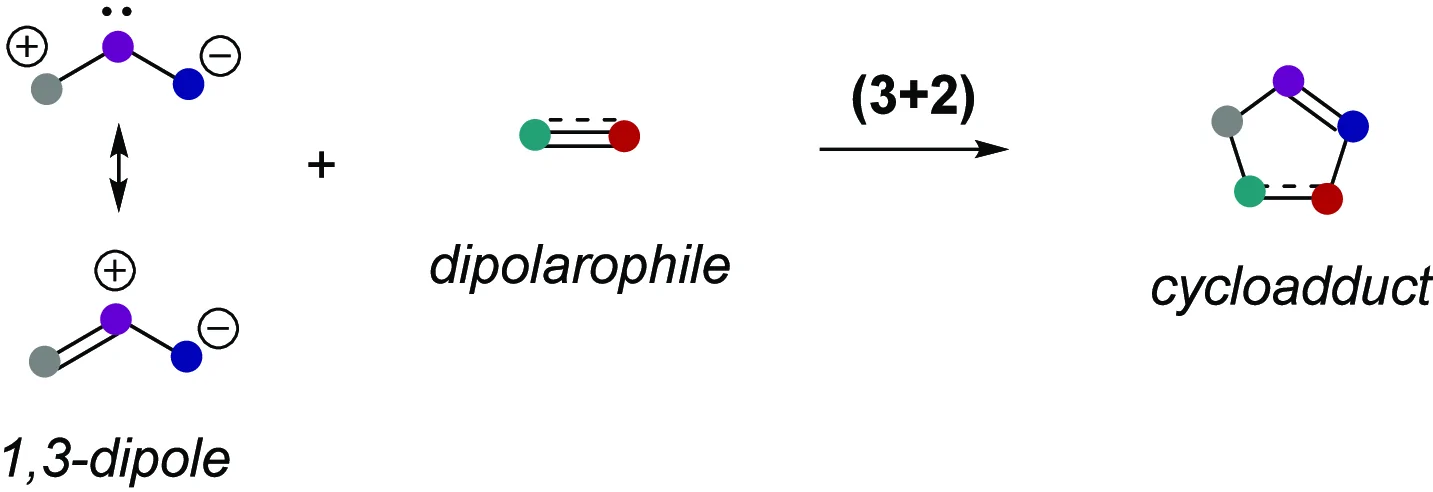
(3 + 2)-Cycloadditions of 1,3-Dipoles to Dipolarophiles

A wide range of species have been identified as competent 1,3-dipoles, including azomethine ylides, azides, nitrile oxides or nitrones, among others; which have been extensively employed for the synthesis of densely functionalized heterocycles, often under Lewis or Brønsted acid catalysis.^278^ More recently, the emergence of photocatalytic methodologies has enabled alternative strategies for generating 1,3-dipoles from diverse precursor classes, significantly expanding the synthetic toolbox available for heterocycle construction. Furthermore, these advances have facilitated access to other highly reactive intermediates, including distonic radical ions^279^ or ketocarbenes, thereby broadening the range of substrates and reaction pathways that can be harnessed for the preparation of valuable five-membered heterocyclic frameworks.

According to the IUPAC,^1^ the term “cycloaddition” denotes a a reaction in which two or more unsaturated molecules (or parts of the same molecule) combine with the formation of a cyclic adduct in which there is a net reduction of the bond multiplicity. Cycloadditions may proceed via concerted pericyclic pathways or through nonconcerted, stepwise mechanisms. In this section, we focus on photochemical transformations that convert reactants to products through a single cycloaddition event, irrespective of whether the underlying mechanism is concerted or stepwise. By contrast, processes involving multiple discrete bond-forming events and intermediates have been treated under the broader category of annulations and are therefore excluded. For instance, cascade sequences based on radical addition followed by cyclization fall outside the scope of this discussion; comprehensive reviews addressing such photochemical cascade annulation strategies, are available elsewhere.^44,45,53,59,279–282^ Likewise, light-driven (3 + 2)-cycloadditions in which the initially formed cycloadduct undergoes subsequent transformation into a more thermodynamically stable product are not considered herein.

The examples compiled in this section encompass cases in which photoexcitation enables the generation of either the 1,3-dipole or the dipolarophile. The material is organized according to both the mechanistic regime governing the cycloaddition and the nature of the precursors employed for constructing the five-membered heterocyclic core. At a general level, methodologies are classified based on the mode of activation: (i) *direct photoexcitation*, where the reacting substrates are promoted to electronically excited states, evolving to reactive 1,3-dipoles; and (ii) *photocatalytic approaches*, in which a photocatalyst mediates the formation of reactive intermediates, such as ylides or radical cation species. Within the latter category, further subdivision is provided according to the type of precursor used to generate the three-atom component involved in the (3 + 2)-cycloaddition.

### 4.1 Direct irradiation methods

One strategy for enabling photochemical (3 + 2)-cycloadditions involves the direct photoexcitation of at least one reactant. This activation mode leverages the intrinsic absorption properties of the substrate. Here, upon irradiation at an appropriate wavelength, chromophoric species are promoted to electronically excited states, which can subsequently evolve into highly reactive 1,3-dipolar intermediates.

Early studies conducted in the late 1950s–60s on the photochemistry of 2*H*-azirines, tetrazoles and sydnones established a foundational platform for the generation of reactive 1,3-dipoles.^283–288^ Upon UV-light irradiation (λ < 310 nm), these heterocycles undergo efficient ring opening to furnish nitrile ylides **286** or nitrile imines **287** (Scheme 60a-b). These transient intermediates can be readily intercepted in (3 + 2)-cycloaddition reactions, enabling the rapid assembly of *N*-containing 5-membered heterocycles. This photochemical strategy has proven particularly valuable in the context of “click chemistry”, finding widespread application in rapid biorthogonal ligation processes as well as in polymer synthesis.^289^ An important consideration for biological applications is the irradiation energy, as prolonged exposure to high-energy wavelengths (i.e., UV-light) can induce photodamage in living cells. Consequently, substantial efforts have been devoted to shifting photoactivation toward more biocompatible visible and near-infrared regions. One strategy to address this limitation involves the incorporation of auxochromic groups into the photoactivatable substrate, as demonstrated by the groups of Lin^290^ and Barner-Kowollik,^291^ who employed purposely designed tetrazoles and azirines, respectively (Scheme 60c). A wide variety of *photoclick* (3 + 2)-cycloadditions have been developed in the last decades, and these transformations have been comprehensively reviewed in recent literature.^289^ On the other hand, continuous-flow methodologies have been applied for the rapid and controlled generation of some 1,3-dipoles, upon direct irradiation of 2*H*-azirines. For instance, Kirsching and co-workers have designed a flow protocol for the *in situ* generation of nitrile ylides **1** from vinyl azides,^292^ followed by (3 + 2)-cycloadditions with electron-deficient alkenes and alkynes. More recently, the group of Baumann has applied this technology to generate nitrile ylides **1** from diarylazirines under irradiation at 365 nm, enabling their subsequent trapping with CO_2_ to afford oxazolones.^293^

**Scheme 60:**
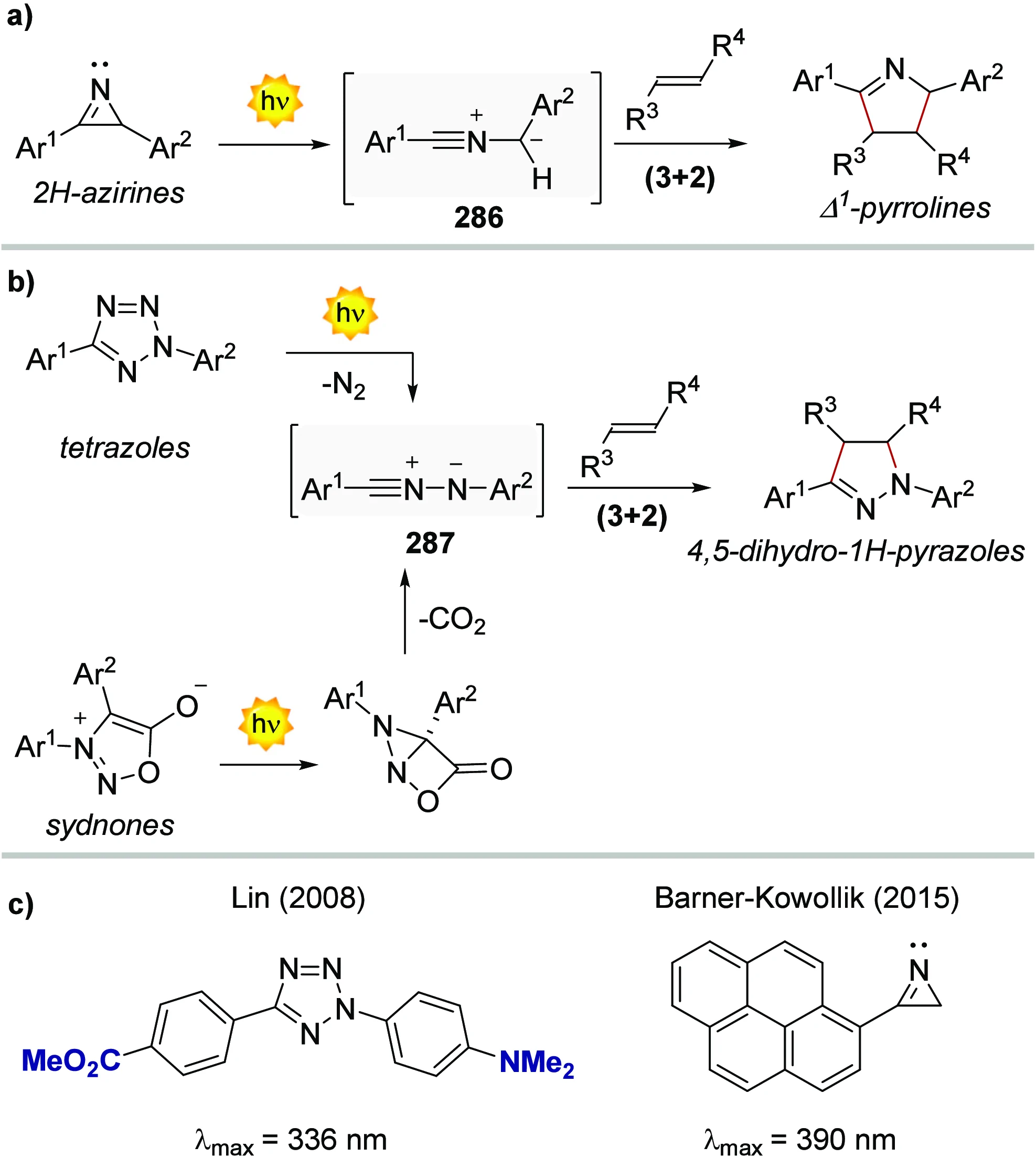
Photoinduced Generation of (a) Nitrile Ylides 286 from Azirines and (b) Nitrile Imines 287 from Tetrazoles or Sydnones; (c) Examples of Photoactivatable Tetrazoles and Azirines at λ > 365 nm

Photolysis of 2*H*-azirines is well established to induce C–C bond cleavage, generating reactive nitrile ylides **286**. However, alternative reactivity patterns have also been identified. In particular, studies on azirines bearing electron-withdrawing substituents revealed a distinct photochemical pathway involving selective C–N bond cleavage. Under these conditions, trapping of the resulting diradical intermediate with molecular oxygen leads to the formation of nitrile oxides, representing an unusual divergence from classical azirine photochemistry and providing access to complementary 1,3-dipolar intermediates.^294^

Other studies have demonstrated the generation of carbonyl and azomethine ylides through the photochemical activation of other strained three-membered rings, such as epoxides and aziridines. For instance, Kamboj and co-workers reported intramolecular (3 + 2)-cycloadditions initiated by photolysis of benzoyl-substituted oxiranes (with λ_max ≈ 320 nm), leading to carbonyl ylide intermediates.^295,296^ In a related approach, Sugiyama and co-workers employed trisubstituted aziridines under UV irradiation (λ = 254 nm) to generate azomethine ylides, which subsequently underwent (3 + 2)-cycloaddition with electron-deficient alkenes.^297^ Mechanistic investigations in the latter study support an initial homolytic C–C bond cleavage of aziridines, giving rise to 1,3-biradical species.

Ketocarbenes are another class of conjugated 1,3-systems capable of engaging in (3 + 2)-cycloaddition reactions with dipolarophiles. These transient intermediates are readily generated from well-established precursors, including iodonium ylides, diazoacetates and diazoketones (Scheme 61). For instance, in 2000, Hadjiarapoglou and co-workers reported the use of 2-phenyliodonio-5,5-dimethyl-1,3-dioxacyclohexanemethylide **288**, which, upon UV-light irradiation in the presence of various alkenes, afforded the corresponding dihydrofuran derivatives **290**.^298^ In a complementary approach, Maiti’s group employed α-carbonyl diazo compounds under visible-light irradiation (λ = 440 nm) to generate singlet electrophilic carbenes that participate in (3 + 2) cycloadditions with nitrile, thereby providing a diverse array of oxazoles **293**.^299^ Spectroscopic studies on differently substituted diazoacetates (alkyl, aryl and heteroaryl) revealed absorption in the visible region (λ_max_ 390 nm, 440 and 456 nm). Notably, this methodology was also amenable to flow conditions. The method could also be implemented under flow conditions. In the same year, Sen and co-workers, developed an electrophotocatalytic protocol using α-carbonyl diazo compounds, enabling the generation of carbene radical anions.^300^ These intermediates were efficiently trapped by nitriles to form oxazoles, as well as by maleimides to furnish imide-fused pyrroles. The reaction proceeded rapidly (1–1.5 h), and control experiments established that the application of electrical current was essential for productive reactivity.

**Scheme 61:**
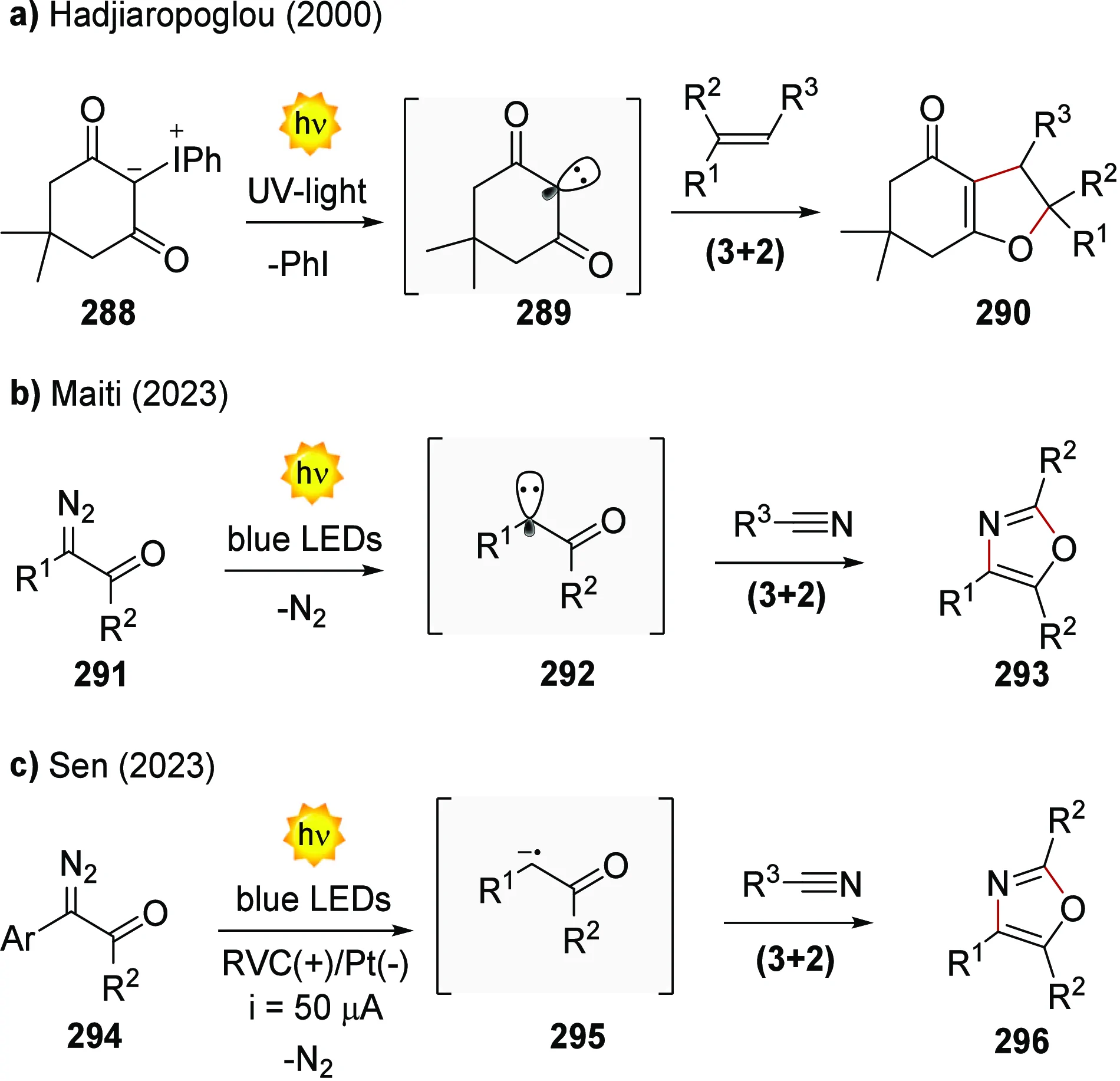
Photoinduced (3 + 2)-Cycloadditions between Ketocarbenes or Conjugated α-Keto Radical Anions and Alkenes or Nitriles (RVC: Reticulated Vitreous Carbon)

An alternative strategy for the activation of α-carbonyl diazo compounds and the generation of reactive 1,3-dipoles was reported by the group of Koenigs (Scheme 62).^301^ This approach relies on the photochemical excitation of azodicarboxylates **298** under blue-light irradiation to access the reactive triplet state **298-T**_**1**_ via a π–π* transition. DFT-calculations suggested that this species undergoes a radical-type addition with **297**, forming the triplet intermediate **301-T**_**1**_. Subsequent denitrogenation proceeds in a nearly barrierless fashion to generate **302-T**_**1**_, which, upon intersystem crossing, furnished the more stable azomethine ylide **303**. Finally, **303** undergoes a (3 + 2)-cycloaddition with the azomethine ylides **299**, delivering highly functionalized triazoles **300**.

**Scheme 62:**
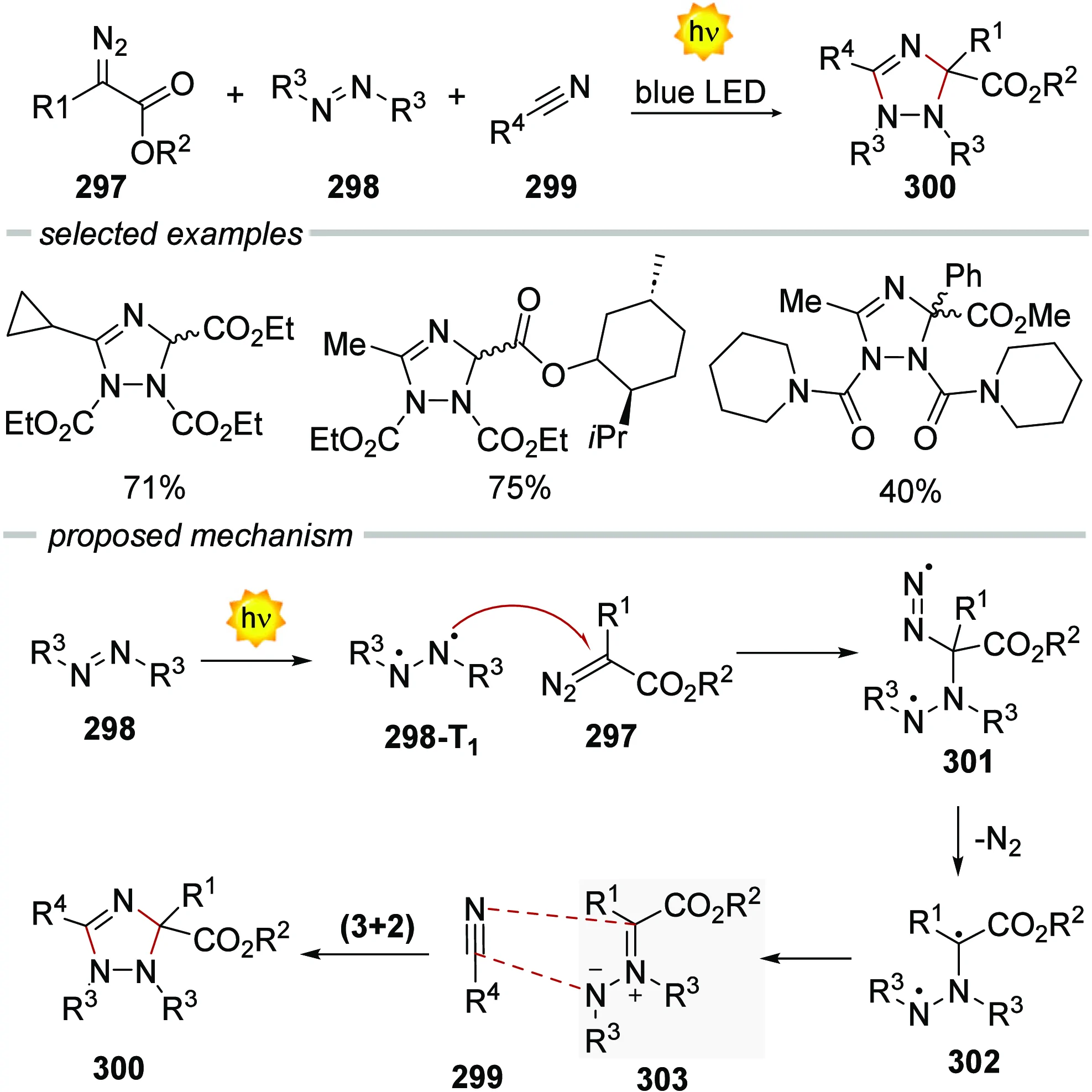
Photochemical Synthesis of Tetrazoles from Diazoacetates, Alkyl Azodicarboxylates and Nitriles

Other subclasses of diazo compounds **2** have likewise proven competent partners in two-component (3 + 2)-cycloaddition manifolds. For instance, Li and co-workers reported that azobenzenes (i.e., **298**, where R^3^ = Ar) undergo sunlight-driven (3 + 2) cycloadditions with arynes generated *in situ* from 2-(trimethylsilyl)­aryl triflates, thereby providing a direct and efficient entry to structurally diverse carbazole frameworks.^302^

In a seminal contribution, Suero and co-workers demonstrated that hypervalent iodine­(III) reagents **304** can be effectively harnessed under photoredox catalysis to generate diazomethyl radicals, thereby enabling site-selective C–H diazomethylation of (hetero)­arenes.^303^ This study has sparked considerable interest in the use of such reagents **304** across diverse synthetic scenarios, including (3 + 2)-cycloaddition chemistry (Scheme 63).^304–306^ Later, alternative diazo-based precursors, such as α-diazosulfonium salts **305** have proven to be efficient sources of these valuable radicals under photoredox conditions.^307^ Complementary to these photocatalytic approaches, direct excitation of hypervalent iodine­(III) reagents **304** and α-diazosulfonium salts **305** has been also shown to provide access to these reactive intermediates under catalyst-free conditions.^306,308,309^

**Scheme 63:**
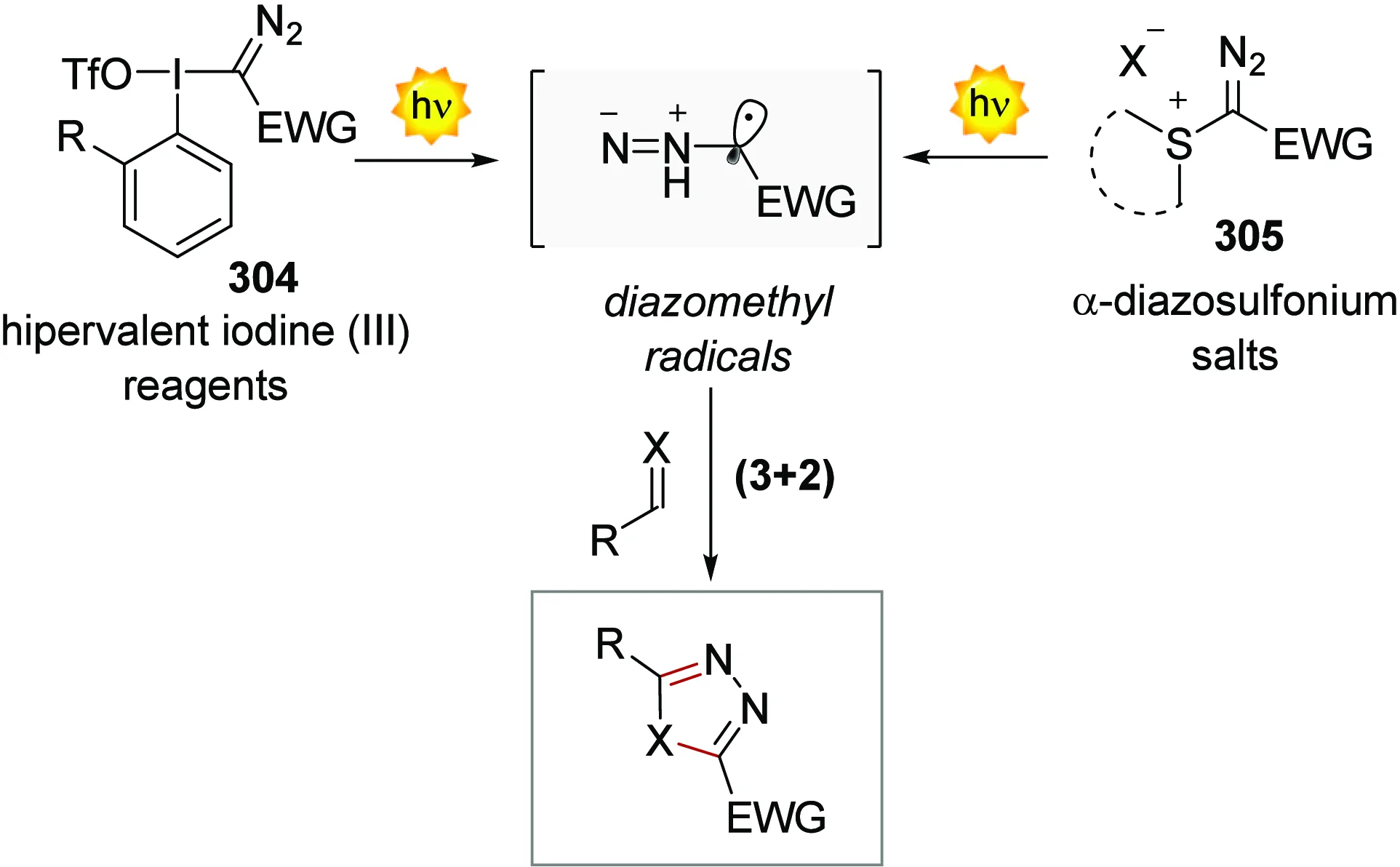
Photochemical Generation of Diazomethyl Radicals from Hypervalent Iodine Reagents and α-Diazonium Salts

For instance, Han and co-workers. used the trifluoromethylated hypervalent iodine­(III) reagent **306** in combination with 2-oxo-2-aryl acetic acids **307** to synthesize a variety of 5-CF_3_-1,3,4-oxadiazoles **308** in up to 96% yields (Scheme 64).^308^ Mechanistically, the process is initiated by photoinduced homolytic cleavage of **306**, generating the diazotrifluoromethyl radical **310** along with the iodanyl radical **309**. A subsequent HAT process between **309** and the α-ketoacid **307** affords the carboxyl radical **312** and *ortho*-iodobenzoic acid **311**. Rapid decarboxylation of **312** furnishes the acyl radical **313**, which undergoes radical coupling with the conjugated diazo-derived species **314** to deliver intermediate **315**. Finally, the authors proposed an intramolecular cyclization of **315**, affording the desired product **308**.

**Scheme 64:**
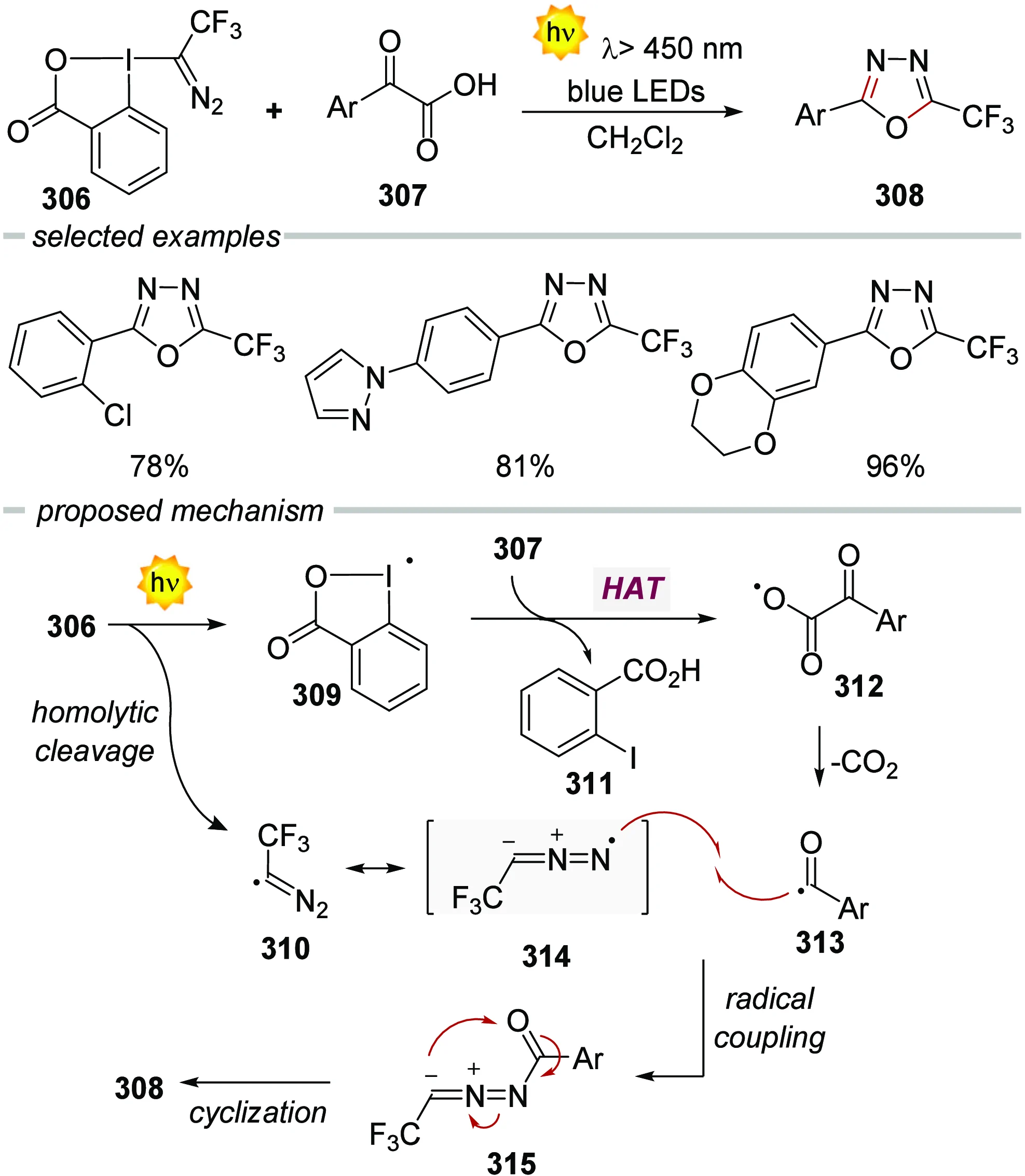
Photochemical Synthesis of 5-CF_3_-1,3,4-Oxadiazoles from Hypervalent Iodine­(III) Reagents and α-Ketoacids

Recently, Gryko, Doyle and co-workers demonstrated the utility of vinyldiazo compounds in photocycloaddition reactions with diverse classes of 1,3-dipoles (Scheme 65).^310^ Their methodology relies on the direct photoexcitation of vinyldiazo precursors **316**, promoting dinitrogen extrusion to generate highly reactive cyclopropene intermediates **317**. Interestingly, this photochemical pathway stands in sharp contrast to the corresponding thermal reactivity, which preferentially leads to pyrazole formation (pathway not shown). The strained cyclopropane intermediates **2** were effectively engaged in (3 + 2)-cycloadditions with diverse dipolar species, including nitrones **318**, azomethine imines **319**, and nitrile oxides **320**, affording highly complex 5-membered heterocycles **321**–**323** with high levels of chemoselectivity, good stereoselectivities and broad functional group tolerance.

**Scheme 65:**
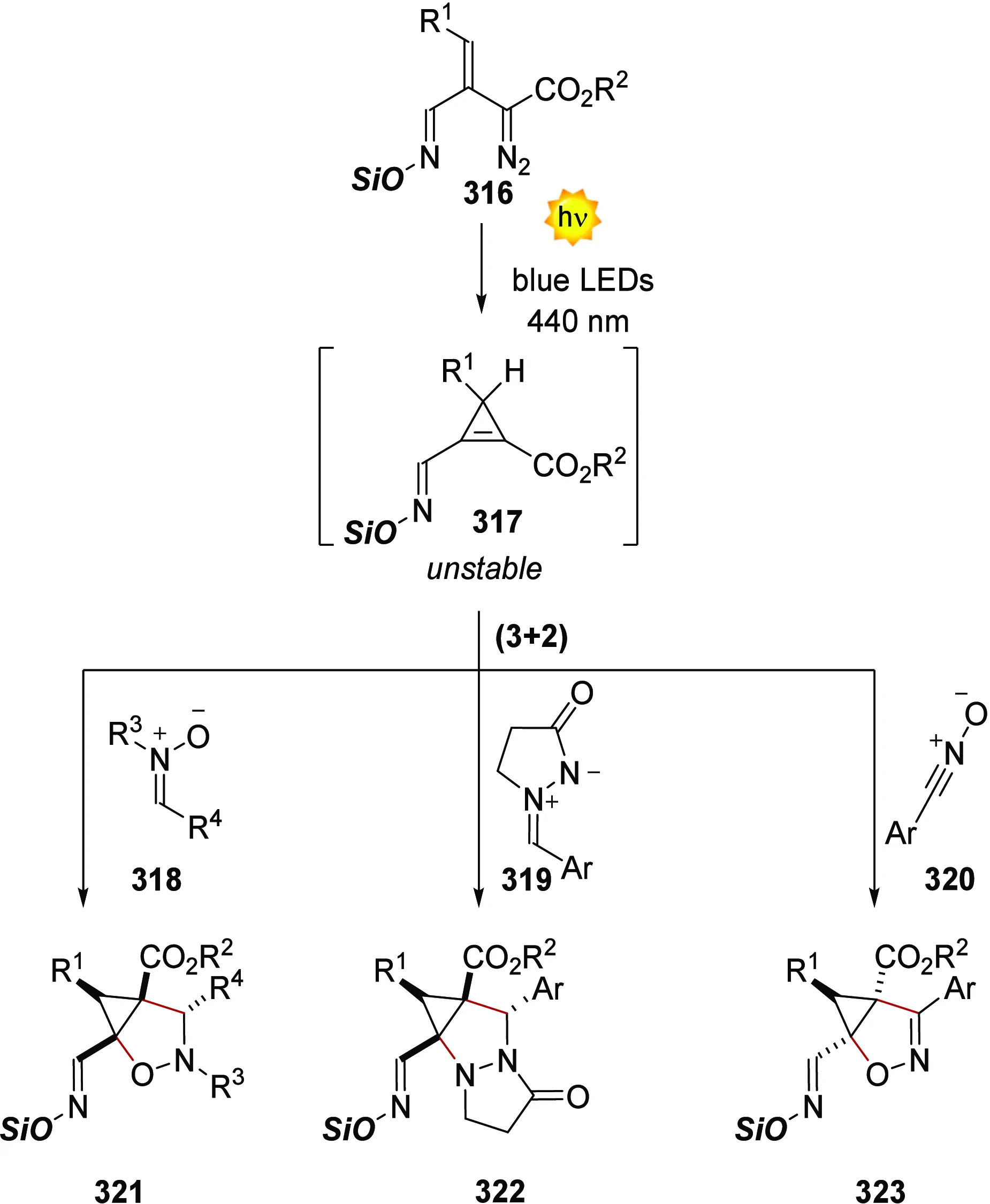
Photochemical Synthesis of Isooxazolidines 321, Pyrazolidin-3-ones and Isoxazolines, from Diazoacetates

A recent example reported by Yu and co-workers describes the photochemical *in situ* generation of sililoxyketenes as reactive 1,2-π systems in (3 + 2) cycloadditions with C,N-cyclic azomethine imines **325** (Scheme 66).^311^ The proposed mechanism, supported by DFT calculations, involves initial formation of the sililoxyketene through [1,3]-silyl migration from the photoexcited acyl α-ketoacylsilanes **327**. Subsequently, nucleophilic addition of the C,N-azomethine imine occurs preferentially at the more accessible face of the sililoxyketene, generating an *E*-enolate intermediate **328**. This intermediate then undergoes intramolecular cyclization onto the prochiral C=N unit of the dihydroisoquinolinium substrate. Cyclization can occur from either face, affording the corresponding pyrazolidines **326** as a mixture of diastereoisomers, with a preference for formation of the *cis*-diastereomer (d.r. up to 7.3:1).

**Scheme 66:**
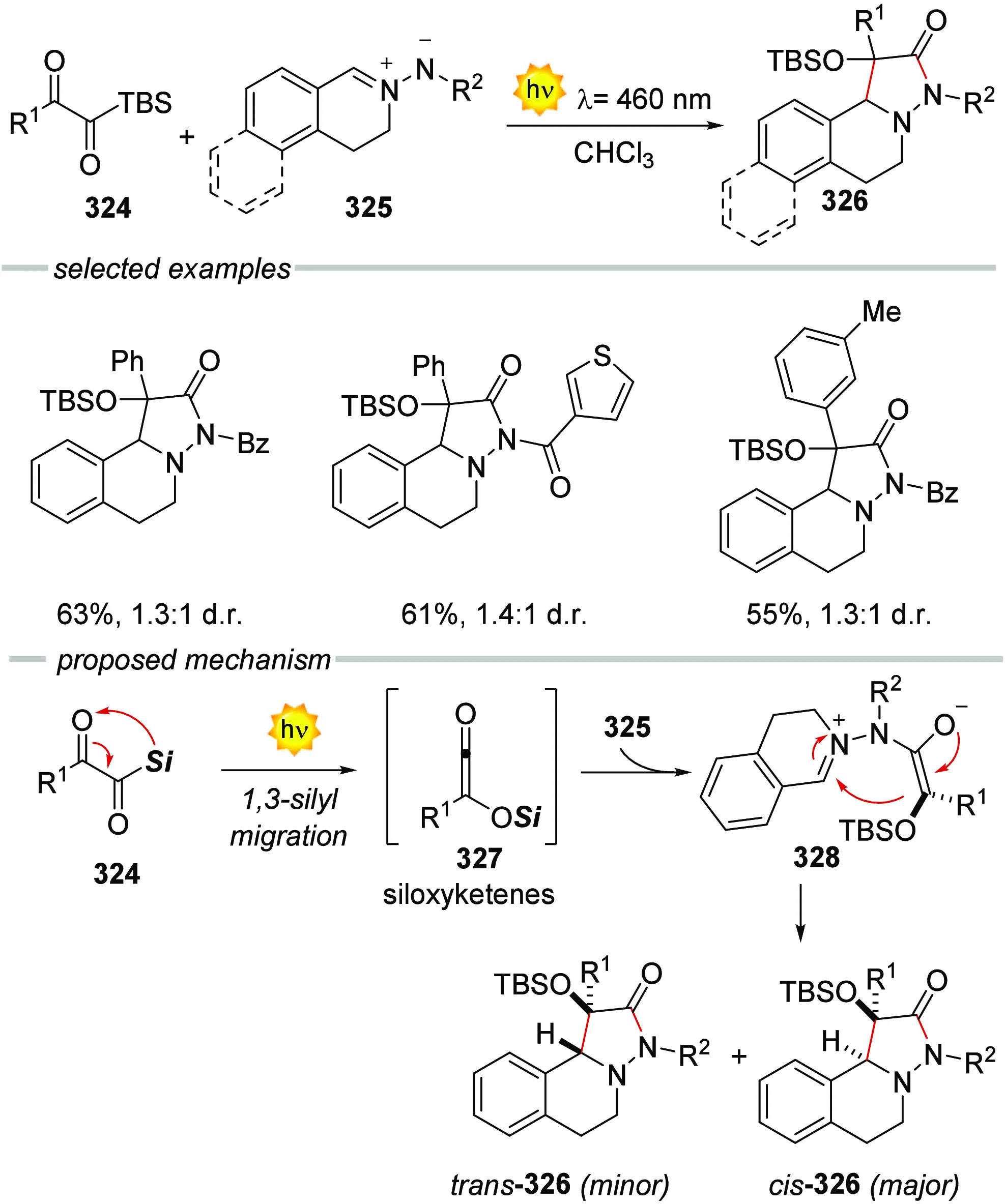
Photochemical Synthesis of Pyrazolidinone Derivatives 3 from Acyl α-Ketoacylsilanes 324 and Azomethine Imines 325

A conceptually distinct approach to the generation of reactive 1,3-dipoles was recently disclosed by Bošković and co-workers, based on the direct photochemical decarbonylation of strained four-membered heterocyclic ketones (Scheme 67).^312^ Upon UV irradiation (λ > 280 nm), readily available substrates such as 3-oxetanone **329** and *N*-Boc-3-azetidinone **330** undergo excitation to their singlet excited states, followed by a stepwise Norrish type I C–C bond cleavage that results in the extrusion of carbon monoxide and formation of carbonyl and azomethine ylides, respectively. Spectroscopic and computational studies support a mechanism involving initial population of an n→π* excited state, subsequent bond scission via a low-energy transition state, and evolution through a conical intersection to yield the corresponding dipolar intermediates. These transient species, generated without the need for metal catalysts or additives, can be efficiently intercepted by a broad range of dipolarophiles, including electron-deficient alkenes, to furnish substituted tetrahydrofurans **331** and pyrrolidines **332**.

**Scheme 67:**
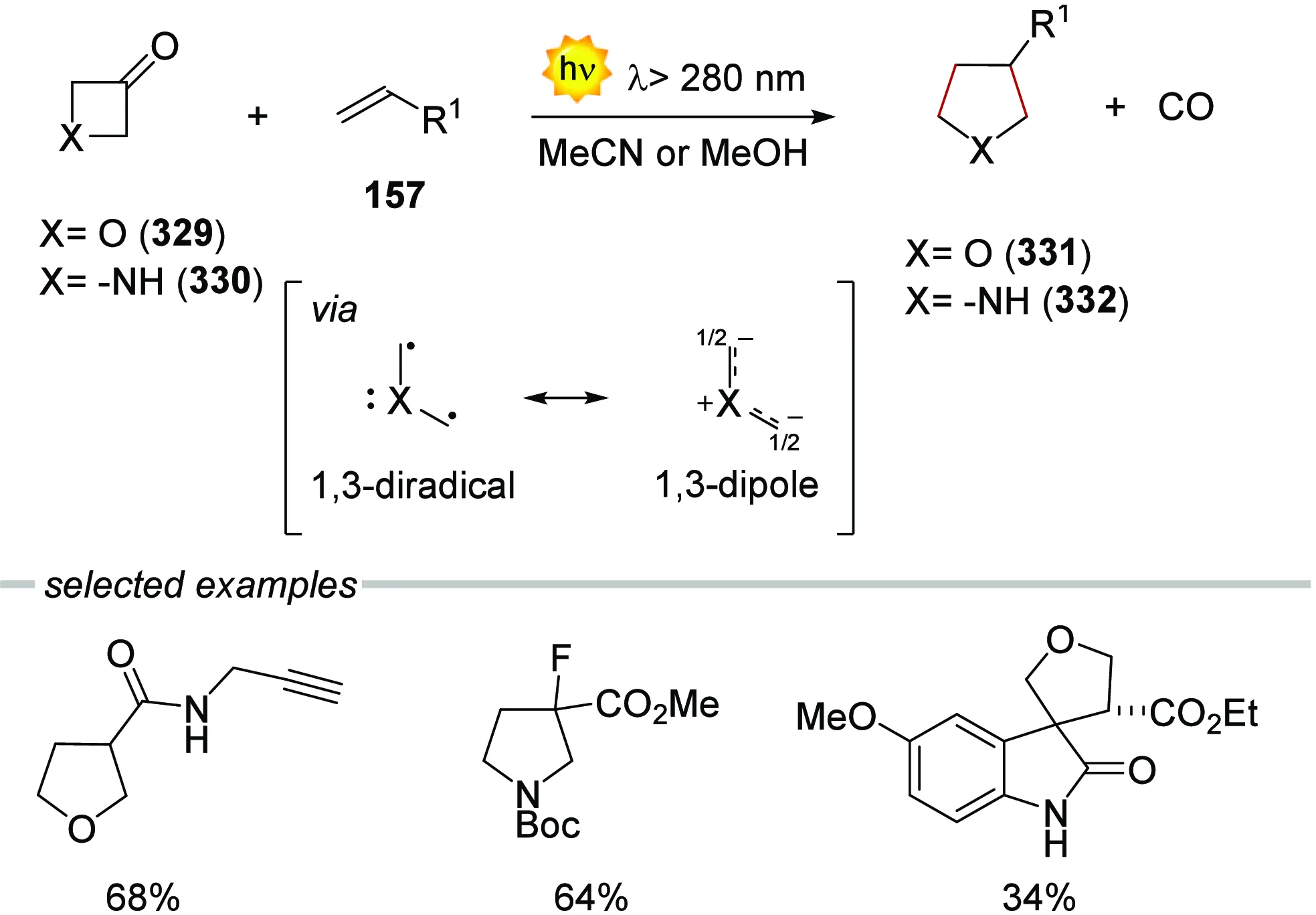
Photochemical Synthesis of Tetrahydrofurans 331 and Pyrrolidines 332 from 3-Oxetanone 329 and *N*-Boc-3-azetidinone 330 and Alkenes

Other additional photochemical (3 + 2)-cycloaddition reactions have been recently disclosed that defy straightforward categorization, reflecting the mechanistic diversity of this transformation. For example, Duan and co-workers developed a method that leverages saturated amines as unconventional coupling partners in place of unsaturated hydrocarbons, enabling their engagement with *N*-imino­(iso)­quinolinium ylides to access pyrazolo-fused (iso)­quinoline frameworks.^313^ On the other hand, Liu and collaborators developed a visible-light promoted (3 + 2)-cycloaddition between 3-alkenylindole-2-ones with 2-mercaptoimidazoles acting as the 1,3-dipolar component. This latter process was initiated by direct photoexcitation of the 3-alkenylindole-2-one substrate.^314^ Alternatively, Zhang and co-workers recently reported a three component strategy, based on the direct photoactivation of *N*-tosylhydrazones, enabling the in situ generation of diazo intermediates. In the presence of *tert*-butyl nitrite the diazo compounds are further transformed into nitronate intermediates, which act as reactive 1,3-dipoles in subsequent (3 + 2)-cycloaddition processes.^315^

### 4.2 EDA-Complex Photochemistry

An alternative activation mode relies on the formation of EDA complexes through the association of an electron-rich donor with an electron-deficient acceptor.^55^ Upon irradiation, these complexes undergo intermolecular SET, generating open-shell intermediates. This radical initiation strategy has also been successfully extended to the domain of (3 + 2)-hetero-cycloaddition processes. For instance, the groups of Zhang^316,317^ and Wang^318^ have independently demonstrated that fluorinated α-diazosulfonium salts **333** can serve as efficient precursors for the synthesis of structurally diverse fluorine-containing heterocycles (Scheme 68). These transformations are initiated *via* the formation of EDA complexes between **333** and various electron donors, including triarylamines **334** in catalytic fashion, hydrazones **336**, and dihydropyridines **338**. Photoexcitation of the resulting complexes **340** triggers the formation of the key diazomethyl radical **341**, which subsequently engages in the corresponding (3 + 2)-cycloaddition pathways.

**Scheme 68:**
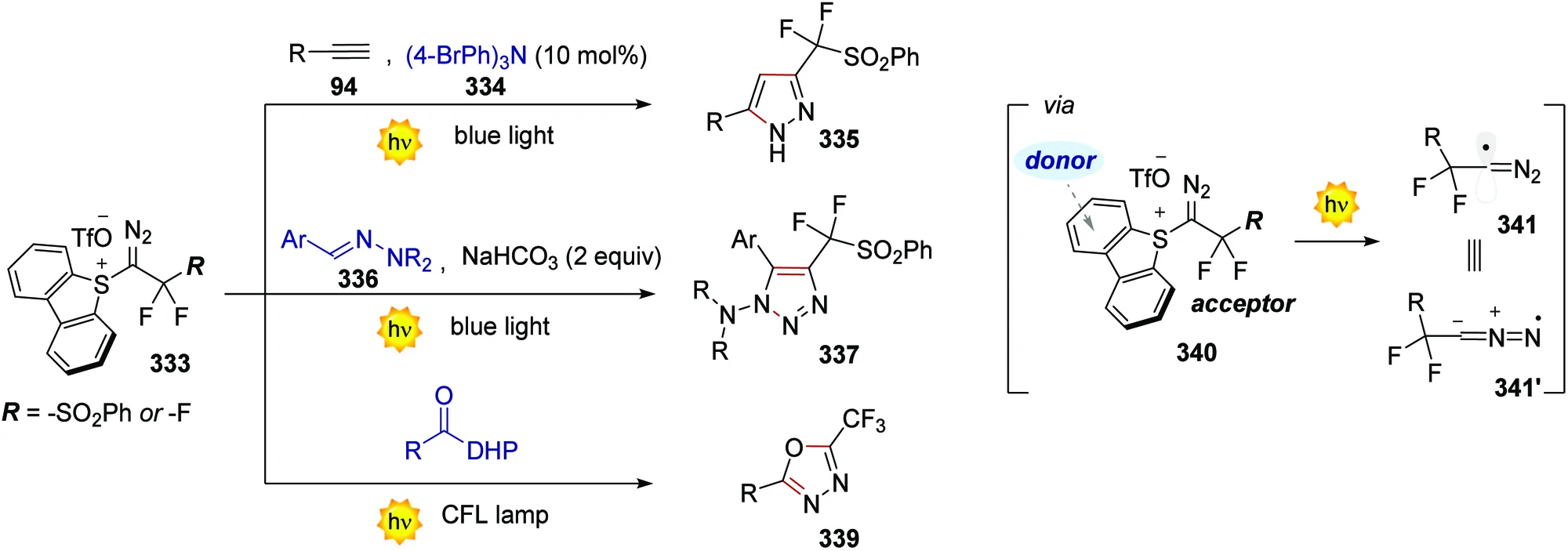
Photochemical Synthesis of 1H-Pyrazoles, Triazoles and Oxadiazoles from Fluorinated α-Diazosulfonium Salts

In 2023, Wang, Das, Zhang and co-workers reported a strategy for the *in situ* generation of diazoalkanes as 1,3-dipoles *via* an EDA activation manifold, (Scheme 69).^319^ Mechanistic studies indicate that the transformation proceeds through the formation of a photoactive EDA complex between the deprotonated *N*-tosylhydrazone **342′** and DBU. Upon visible-light irradiation, this complex undergoes excitation followed by homolytic N–S bond cleavage, generating the diazo intermediate **344** along with a sulfonyl anion. The resulting diazo species **345** subsequently undergoes a (3 + 2) cycloaddition with the alkene partner **157**, delivering the final pyrazoline product **343**.

**Scheme 69:**
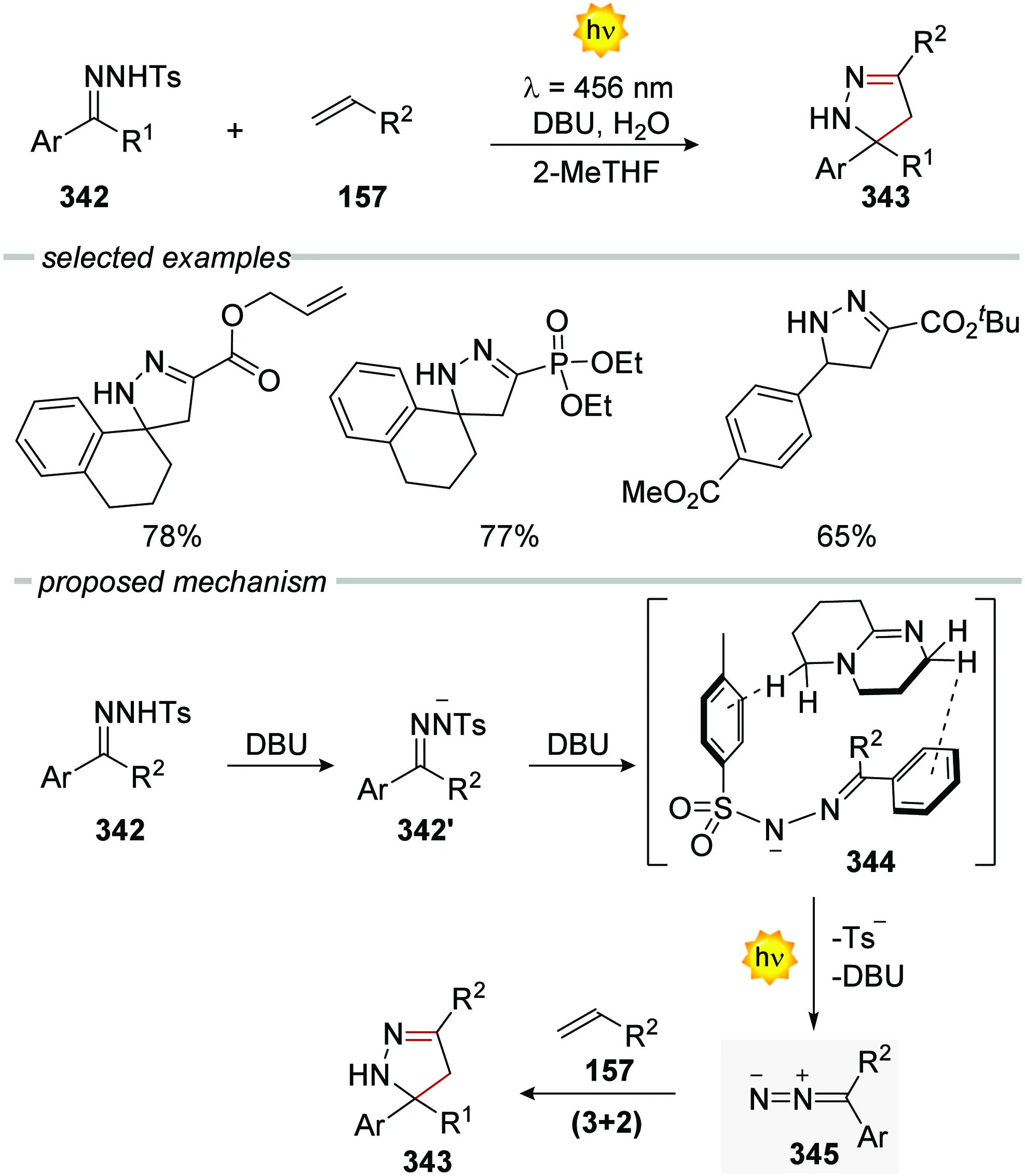
Photochemical Synthesis of (Spiro)­Pyrazolines from Hydrazones and Alkenes

A recent contribution by Li, Shen and co-workers illustrates the potential of EDA complex activation in enabling cascade (3 + 2) cycloaddition processes from simple nitroarenes (Scheme 70).^320^ Mechanistically, the process was proposed to be initiated by the association of electron-deficient nitrobenzene **347** with electron-rich DMAP to form the ground-state EDA complex **349**. Upon visible-light irradiation (λ = 450–460 nm), this complex undergoes SET, generating **351** and the radical cation **350**. Intermediate **351** engages in sequential hydrogen atom transfer (HAT) and protonation events, followed by dehydration, to afford the nitrous intermediate **352**. Further association of **352** with DMAP gives rise to a second EDA complex (**353**), which undergoes analogous SET and HAT processes to furnish the hydroxylamine intermediate **354**. Condensation of **354** with the iminium species **355**, generated within the first HAT event, then delivers the key nitrone intermediate **357**, which ultimately undergoes a (3 + 2) cycloaddition with methacryloyl benzamide **346** to provide the isoxazolidine product **343**. Notably, DMAP plays a dual role as both electron donor and C_1_ synthon in nitrone **357** formation.

**Scheme 70:**
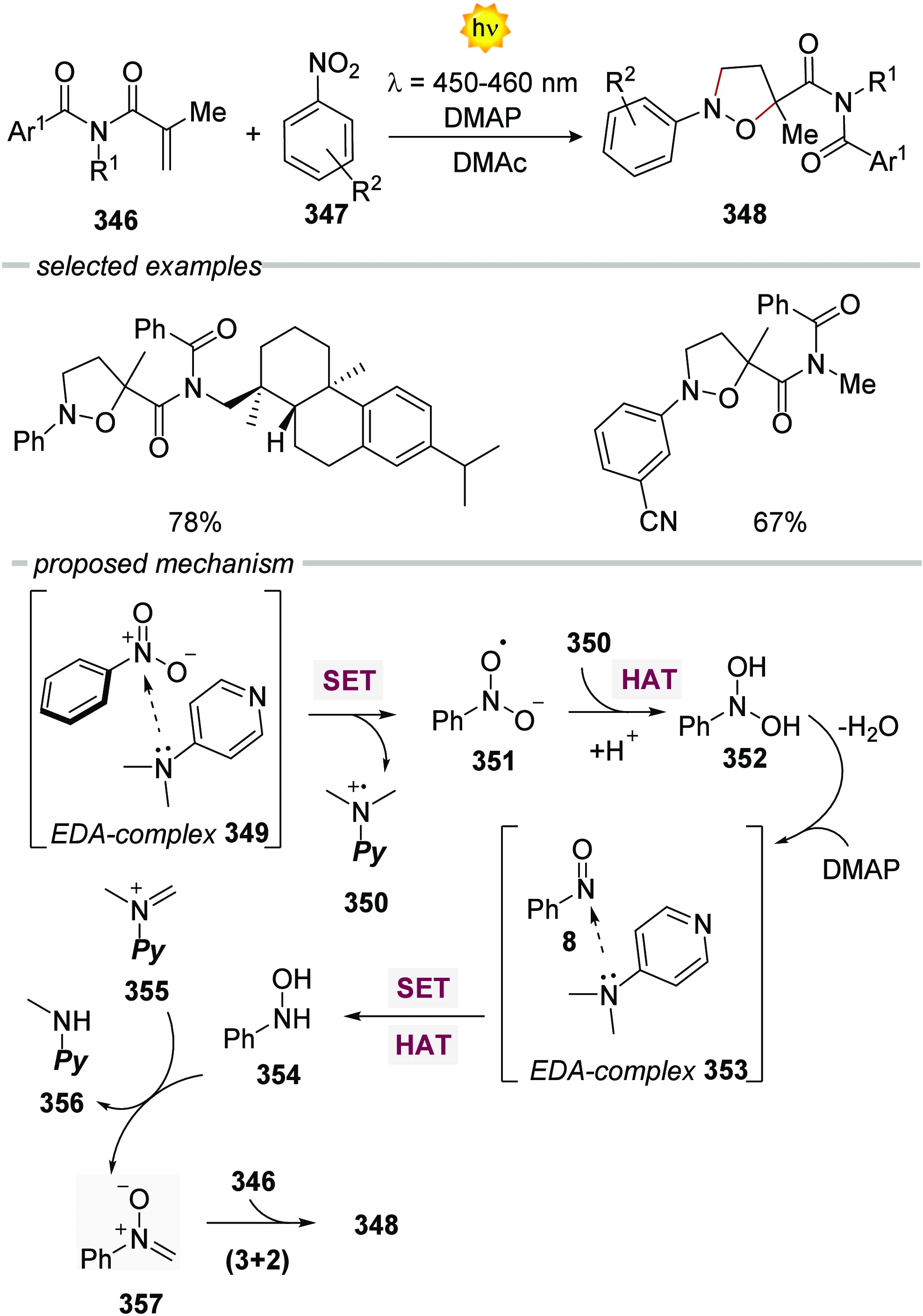
Photochemical Synthesis of Isoxazolidines from Methacryloyl Benzamides and Nitrobenzenes

### 4.3 Photocatalytic Methods

#### 4.3.1 Use of Epoxides

Early studies on the photocatalytic generation of carbonyl ylides from stilbene oxides, and their subsequent engagement in (3 + 2)-cycloadditions were independently reported in the late 1970s by Albini^321^ and Griffin.^322^ Mechanistically, this transformation proceeds via an initial SET process from the epoxide **358** to a photoexcited **PC**, generating a radical cation that undergoes rapid rearrangement to an oxonium radical intermediate **359** (Scheme 71). A subsequent SET from the reduced **PC** to **359** furnishes the corresponding carbonyl ylide **360**. Notably, the reaction typically displays a preference for *cis*-configured products, a selectivity rationalized by minimization of allylic 1,3-strain in intermediate **359**. Accordingly, *cis*-epoxides generally afford improved diastereoselectivity. Despite its operational simplicity, this photocatalytic approach has remained underdeveloped, with only limited substrate scope reported.^323^

**Scheme 71:**
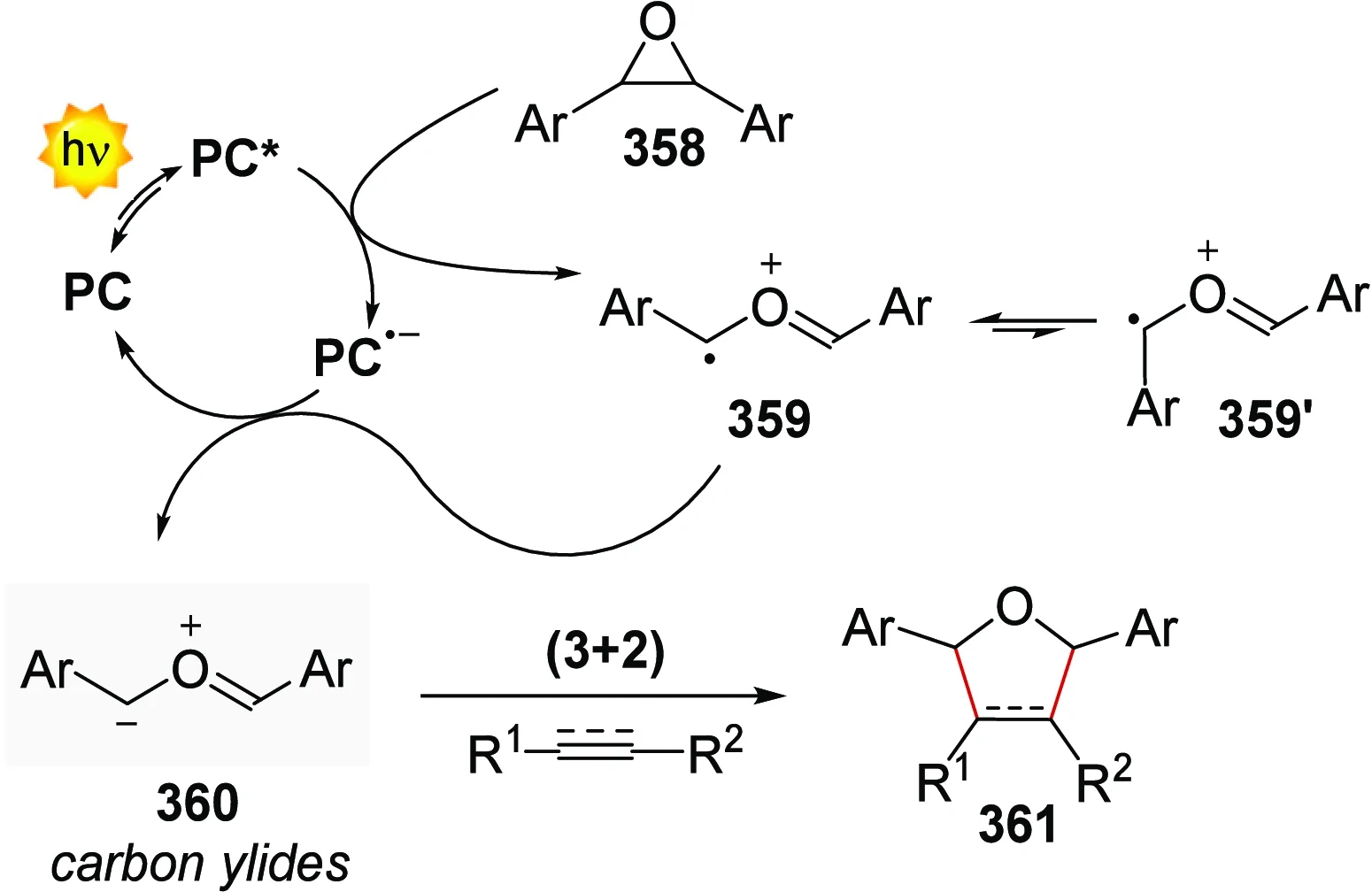
Generation of Carbon Ylides from Epoxides by Means of Photoredox Catalysis

In 2017, the group of Beeler expanded the scope of this strategy using 2,4,6-triphenylpyrylium tetrafluoroborate (**Pyr-2**) as photocatalyst, enabling the efficient formation of carbon ylides from a variety of symmetrical and unsymmetrical diaryloxiranes **362**, which were subsequently trapped by alkynes **363** (Scheme 72).^324^ Building on this reactivity, the same group further demonstrated its synthetic utility through concise routes to diverse subclasses of lignan natural products, wherein a key step involved a (3 + 2)-dipolar hetero-cycloaddition mediated by 2,6-di-*tert*-butylanthracene-9,10-dicarbonitrile (**DCA-1**) as photocatalyst.^325^

**Scheme 72:**
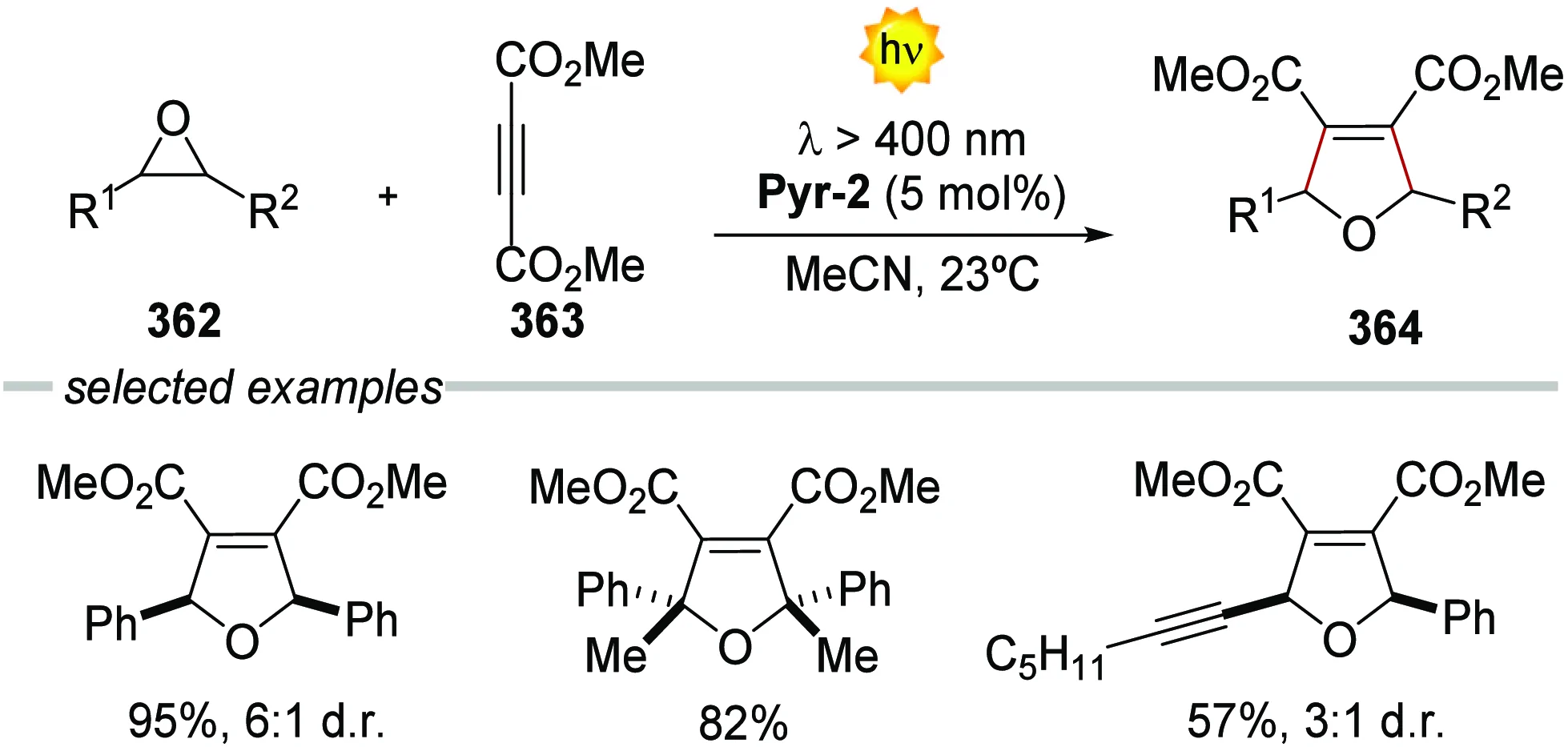
(3 + 2)-Cycloaddition between Photochemically Generated Carbonyl Ylides and Alkynes

#### 4.3.2 Use of 2*H*-Azirine, Aziridines and Derivatives

2*H*-Azirines represent versatile synthetic intermediates for the construction of diverse nitrogen-containing heterocycles, traditionally accessed *via* direct UV irradiation (see Section 1) or transition-metal-catalyzed methodologies. With the emergence of photoredox catalysis, new activation modes have enabled their controlled ring opening through photoinduced SET, thereby providing access to reactive 1,3-dipolar species under mild conditions. In a pioneering study, Xuan and co-workers reported an efficient pyrrole synthesis from 2*H*-azirines **365** and electron-deficient alkynes **366**, employing an acridinium photocatalyst (**Acr-3**) (Scheme 73).^326^ Mechanistically, photoexcitation of the acridinium catalyst promotes SET oxidation of the azirine substrate to generate the radical cation intermediate **368**, which undergoes rapid ring opening to furnish an aza-allenyl radical cation **369** in equilibrium with its electronic isomer **370**. Subsequent radical addition of **370** to the alkyne **366** delivers intermediate **371**, which, upon SET reduction by the reduced form of the photocatalyst, undergoes intramolecular cyclization and rearomatization to afford the pyrrole product **367**. This seminal work has stimulated the development of related cycloaddition manifolds employing a broader range of acceptors with **Acr-3** emerging as a particularly effective photocatalyst (Scheme 74).^327–334^ Furthermore, the methodology has recently translated to continuous-flow conditions using, α,β-unsaturated ketones as acceptors, highlighting its scalability and potential for practical applications.^335^

**Scheme 73:**
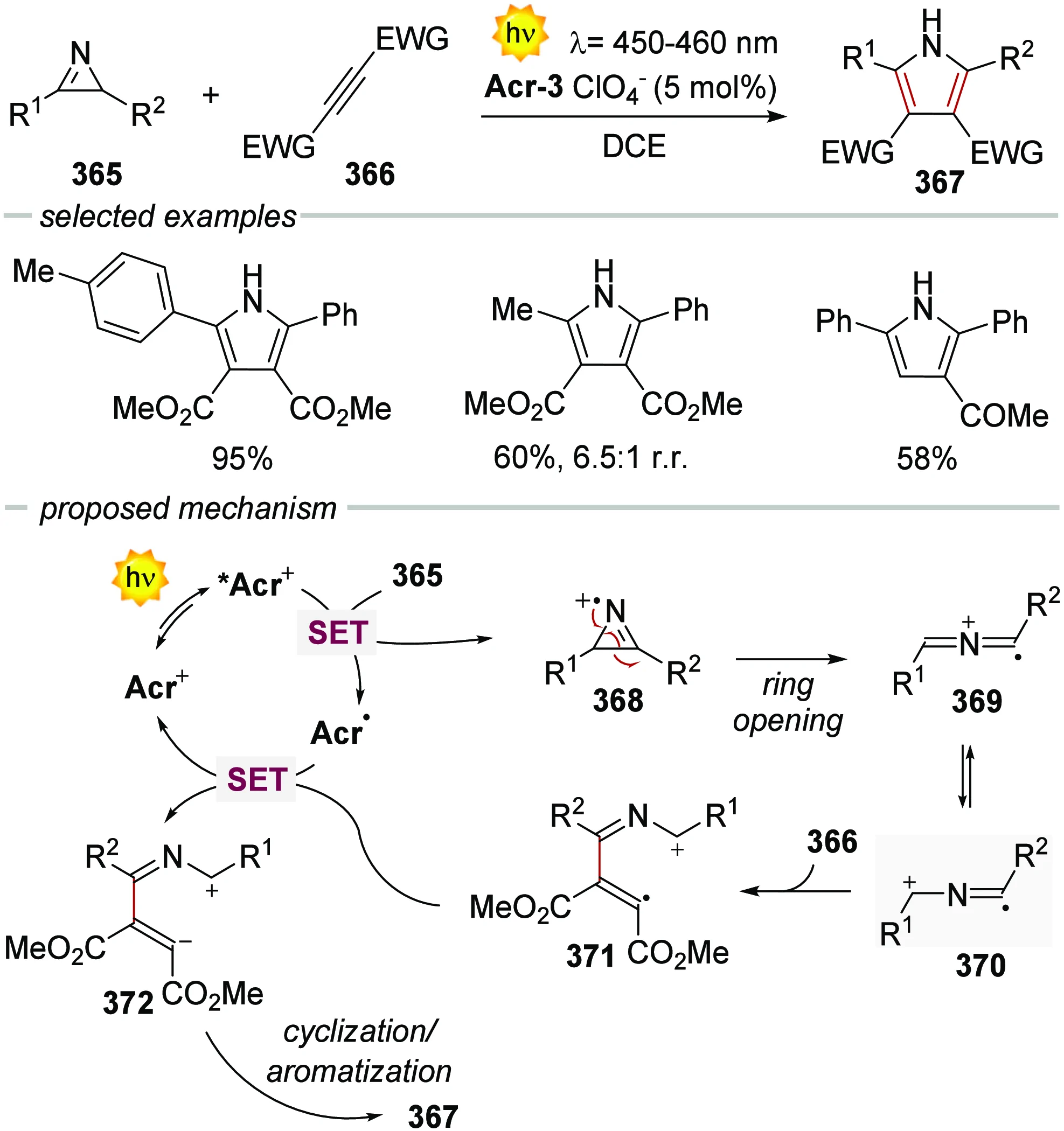
Photochemical Synthesis of Pyrroles from 2*H*-Azirines and Alkynes

**Scheme 74:**
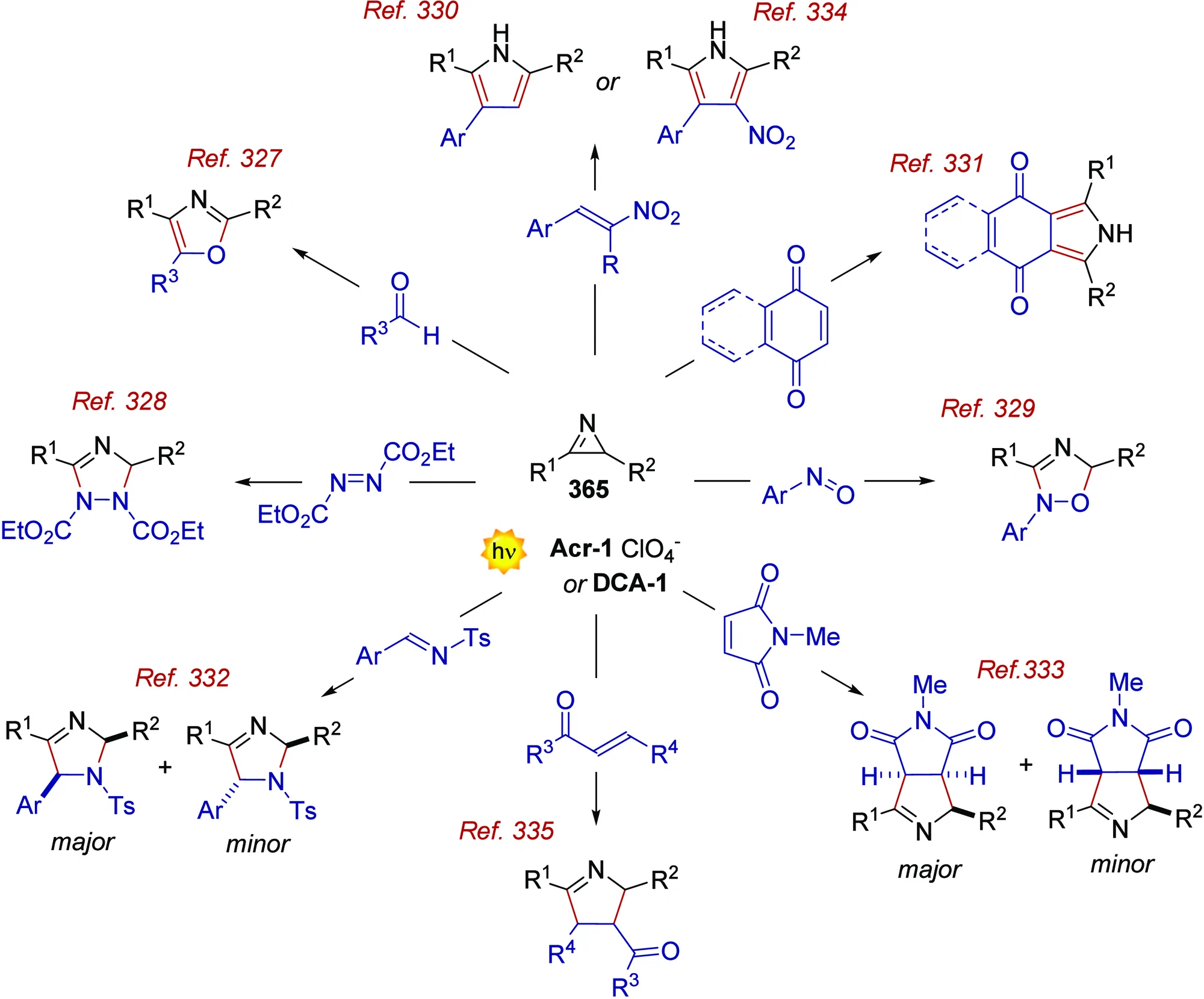
Photocatalytic (3 + 2)-Cycloadditions between 2*H*-Azirines and Different Acceptors

Other *N*-containing three-membered heterocycles have also been exploited as precursors for the generation of 1,3-dipoles under photoredox conditions. For example, Woo and co-workers reported the use of oxaziridines **373** as nitrone precursors, which were subsequently intercepted by alkynes **374** to afford 4-isoxazolines **375** (Scheme 75).^336^ In this transformation, the key nitrone **378** is proposed to arise via SET oxidation of oxaziridine **373** by the photoexited acridinium photocatalyst **Acr-3**, generating a radical cation **376** that undergoes ring-opening to furnish intermediate **377**. Subsequent SET reduction of **377** delivers the nitrone **378**, which then participates in a (3 + 2)-cycloaddition with the alkyne partner **374**. The intermediacy of nitrone **378** was confirmed through its isolation by flash column chromatography and characterization.

**Scheme 75:**
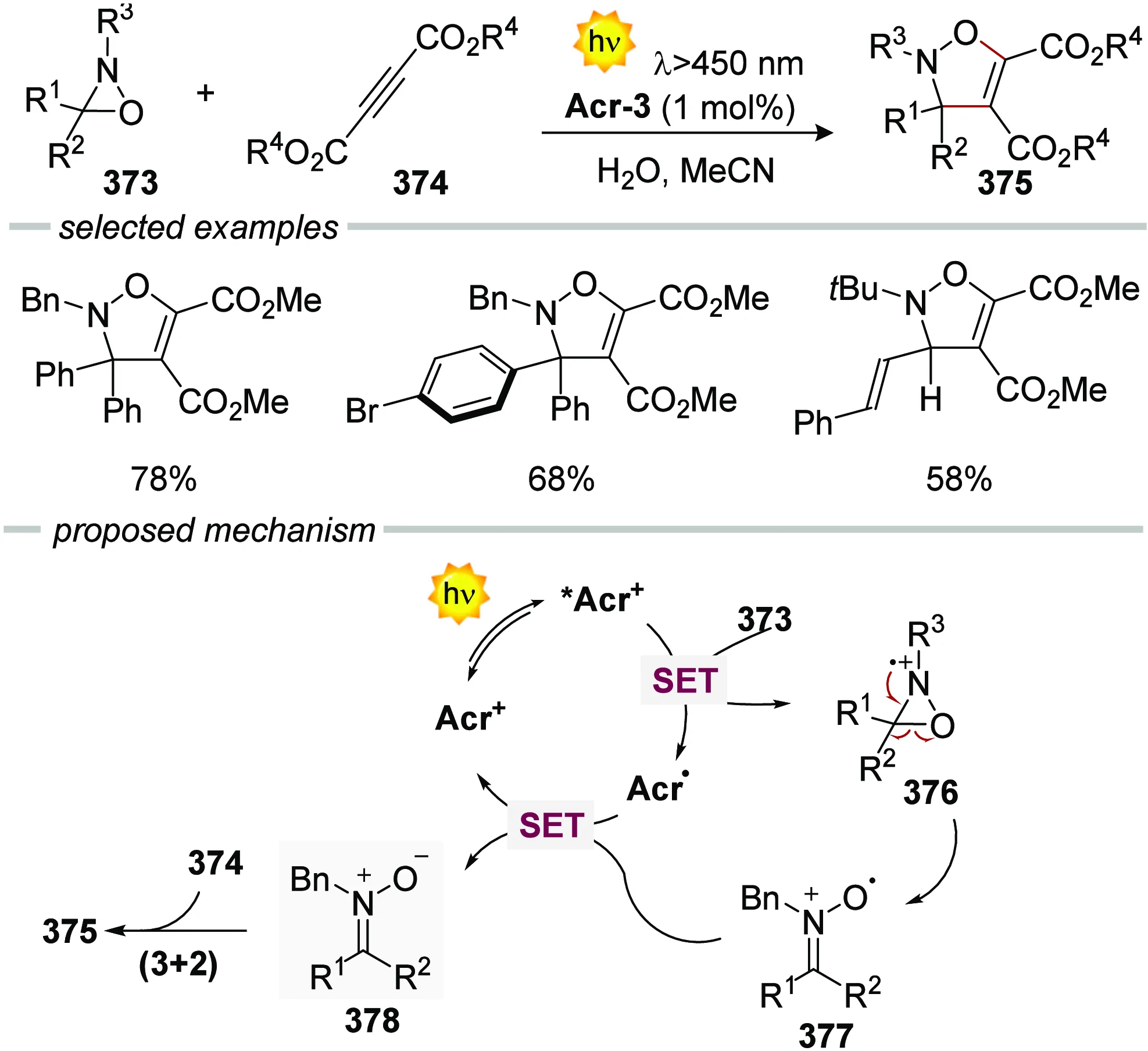
Photocatalytic Synthesis of 4-Isoxazolines from Oxaziridines and Alkynes

On the other hand, the group of Dell’Amico recently disclosed a general photocatalytic strategy for the generation of azomethine ylides from aziridines (Scheme 76).^337^ In this system, anthracene-9,10-dicarbonitrile (**DCA-2**) was identified as an optimal photocatalyst, promoting SET oxidation of a broad range of diarylazetidines **379** to afford radical cation intermediates **382**, which undergo rapid ring opening to furnish azomethine ylides **383** following reduction by **DCA** ^**•–**^. These reactive dipoles reacted with a variety of dipolarophiles **380**, including olefins, aldehydes, azodicarboxylates, and Schiff bases, delivering structural diverse products **381** with high diastereoselectivity.

**Scheme 76:**
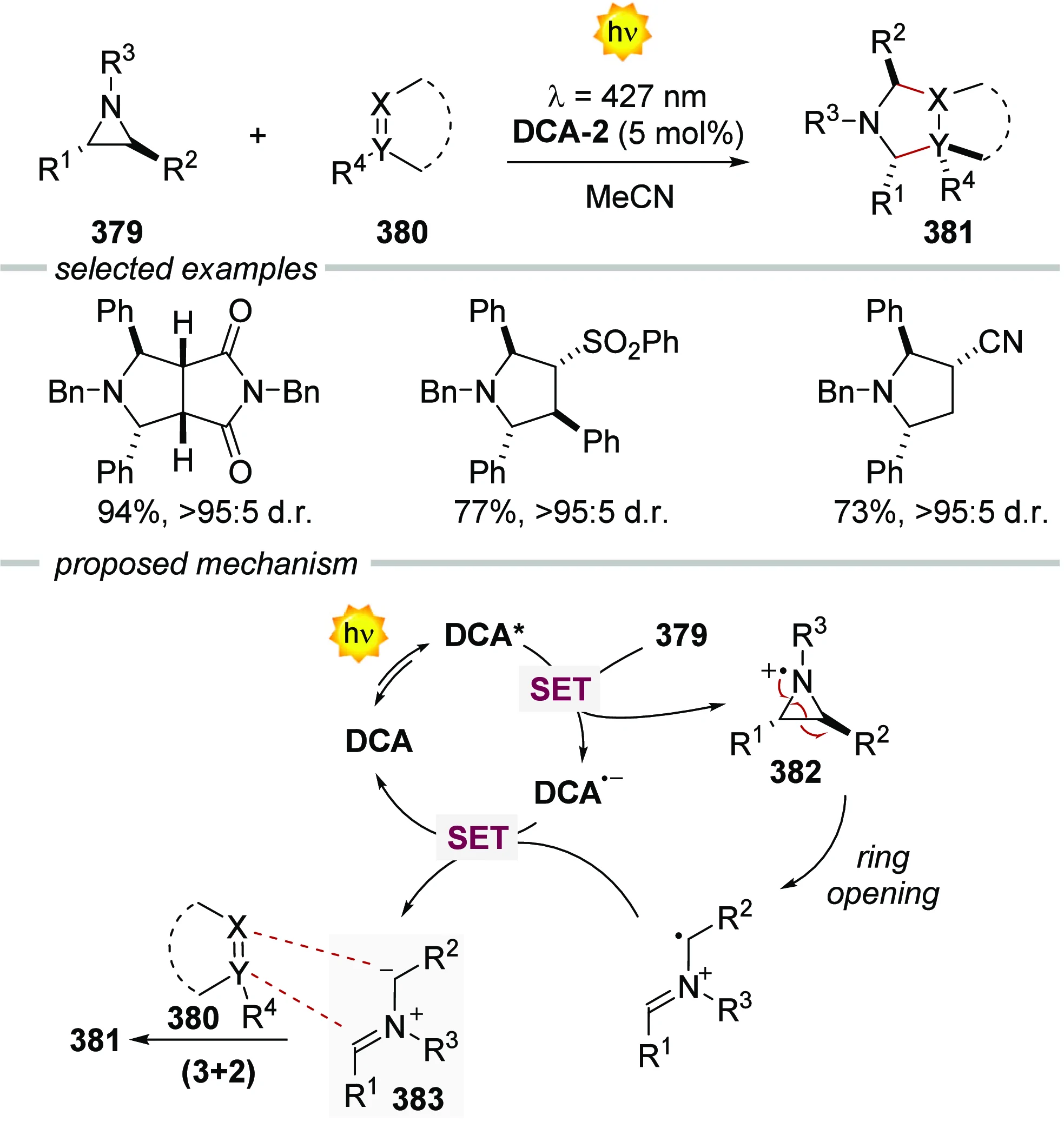
Photocatalytic Synthesis of Functionalized Pyrrolidines from Aziridines

When alkynes are employed as reaction partners under aerobic conditions, the transformation proceeds through a photocatalytic cascade to afford densely functionalized pyrroles **384** in high yields (Scheme 77). In this variant, the initially formed (3 + 2)-cycloaddition 2,5-dihydropyrole undergoes sequential SET oxidation by DCA*, followed by a HAT process involving the superoxide (O_2_^•–^). Final deprotonation by in situ generated HO_2_–leads to the formation of the pyrrole product **384**. Importantly, the method was shown to be also amenable to late-stage functionalization of biologically relevant scaffolds, including glucose or phenylglycine.

**Scheme 77:**
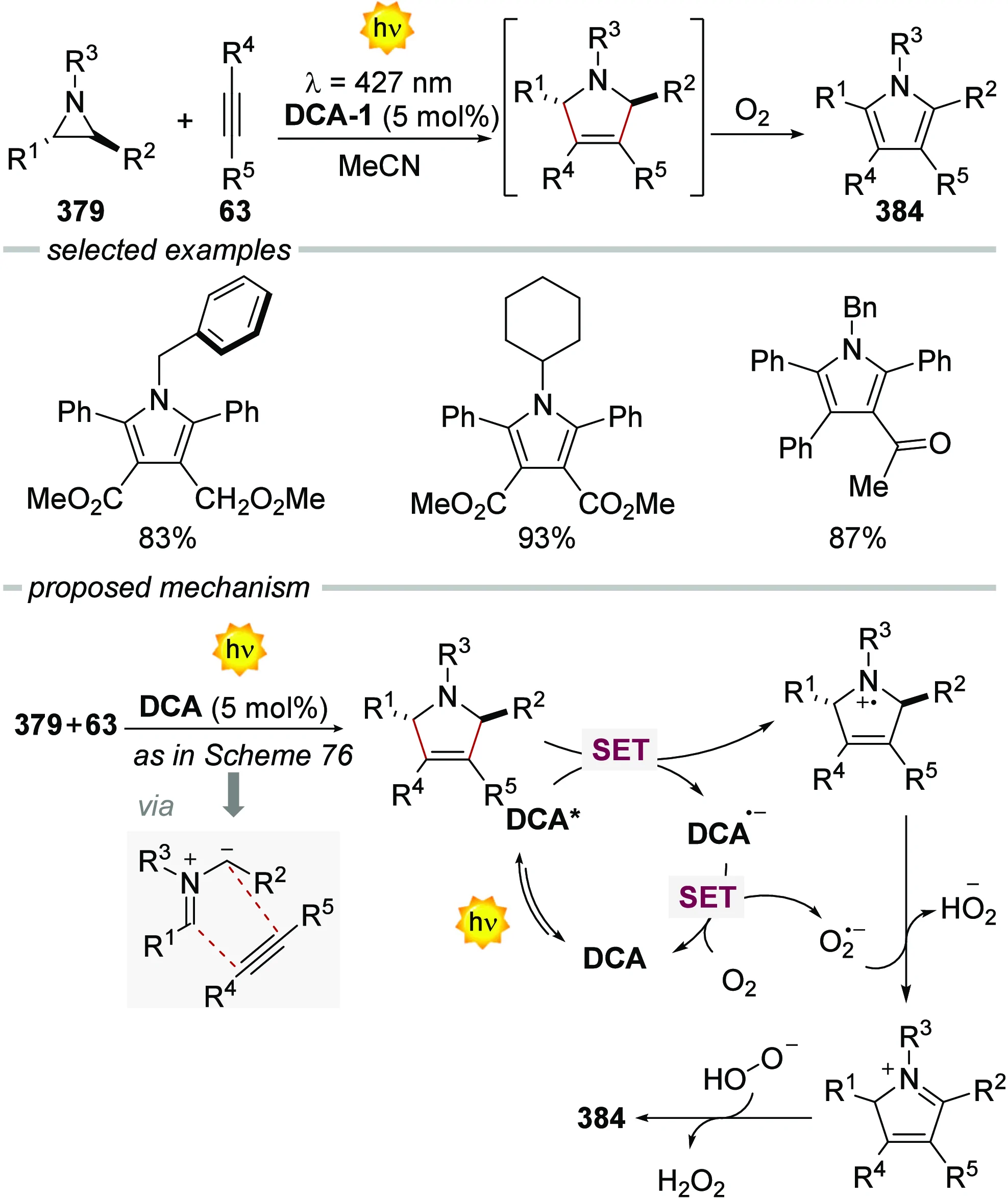
Photocatalytic Synthesis of Functionalized Pyrroles from Aziridines and Alkynes

#### 4.3.3 Use of α-Aminoesters

An alternative strategy for the generation of azomethine ylides exploits dihydroisoquinoline esters under photoredox catalysis. Pioneering contributions were independently reported in 2011 by Zou^338^ and Rueping^339^ (Scheme 78). Both approaches employ **Ru-1** as photocatalyst to promote single-electron transfer (SET) oxidation of the dihydroisoquinoline ester **385**, affording the corresponding radical cation **388**. This intermediate undergoes HAT with superoxide (O_2_^•–^), generating the iminium ion **389**, which upon deprotonation by in situ formed HO_2_^–^ furnishes the key azomethine ylide **390**. In the study by Zou, a broad range of dipolarophiles—including maleimides, styrene derivatives, alkynes, and maleic anhydride—were successfully employed with **387a**, with the optimized conditions favoring the formation of oxidized fused-pyrrole products **387b**. In contrast, Rueping and co-workers primarily utilized **385b** with maleimides, leading predominantly to the direct fused-pyrrolidine (3 + 2)-cycloadducts **387a**. Alternative photocatalysts such as Rose Bengal (**RB**),^340^ methylene blue (**MB**),^341^ porphyrin-based covalent organic frameworks (COF)­s,^342^ among others,^343–347^ have likewise demonstrated efficacy in promoting this transformation. On the other hand, structurally related α-amino esters derived from 1,2,3,4-tetrahydro-β-carbolines have been successfully used as precursors in other (3 + 2)-hetero-cycloadditions for the construction of complex fused β-carboline architectures.^348,349^

**Scheme 78:**
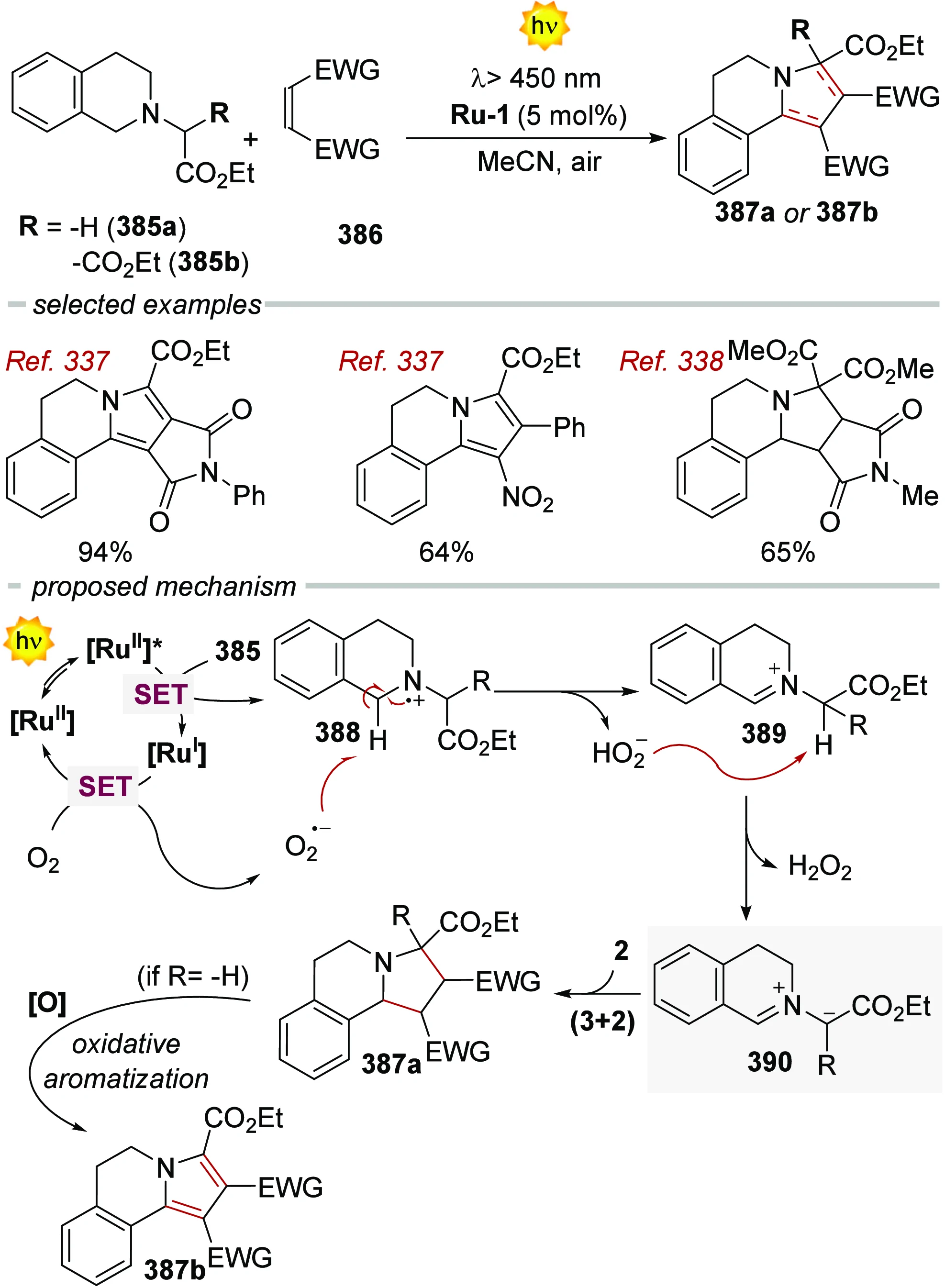
(3 + 2)-Cycloaddition between Photochemically Generated Azomethine Ylides and Alkenes

#### 4.3.4 Use of Nitrones, Oximes, and Nitrile Oxides

Nitrones constitute one of the most extensively studied classes of 1,3-dipoles, owing to their stability, ease of handling, and the synthetic versatility of the resulting isoxazolidine (3 + 2)-cycloaddition adducts, which can be readily transformed into valuable β-amino acids, β-lactams, and β-amino alcohols.^350^ Photoredox catalysis have provided a valuable platform for the *in situ* generation of these dipolar species, an approach that is particularly advantageous for accessing otherwise unstable or sensitive nitrones. In this context, Rueping and co-workers reported in 2014 a strategy that exploits the mild conditions of photoredox catalysis to generate reactive nitrone intermediates *in situ*, enabling their direct engagement in subsequent (3 + 2) cycloaddition processes with alkenes (Scheme 79).^351^ Specifically, the authors used *N*-alkylsubstituted arylhydroxylamines **391** as precursors, which are oxidized to nitrones with the mediation of an iridium photocatalyst **Ir-7**. Several mechanistic pathways for nitrone formation were proposed. A first SET oxidation from the excited Ir^III^ to **391**, affords the radical cation **393**, together with Ir^II^, which is oxidized by O_2_ to regenerate the catalyst Ir^III^ and produce O_2_^•–^. The open-shell species **393** can then undergo: *(i)* a HAT process mediated by O_2_^•–^ to form **394**, that after deprotonation by HOO^–^ furnished the nitrone **399**; *(ii)* deprotonation to form a carbon-centered radical **395**, that after oxidation and deprotonation delivers **399**; or *(iii)* deprotonation to form the nitroxide species **396**, which can evolve to nitrone **399** either through disproportionation (i.e., two molecules of **396** leading to **399** + **391**; pathway not shown in Scheme 79) or via sequential electron- and proton transfer processes.

**Scheme 79:**
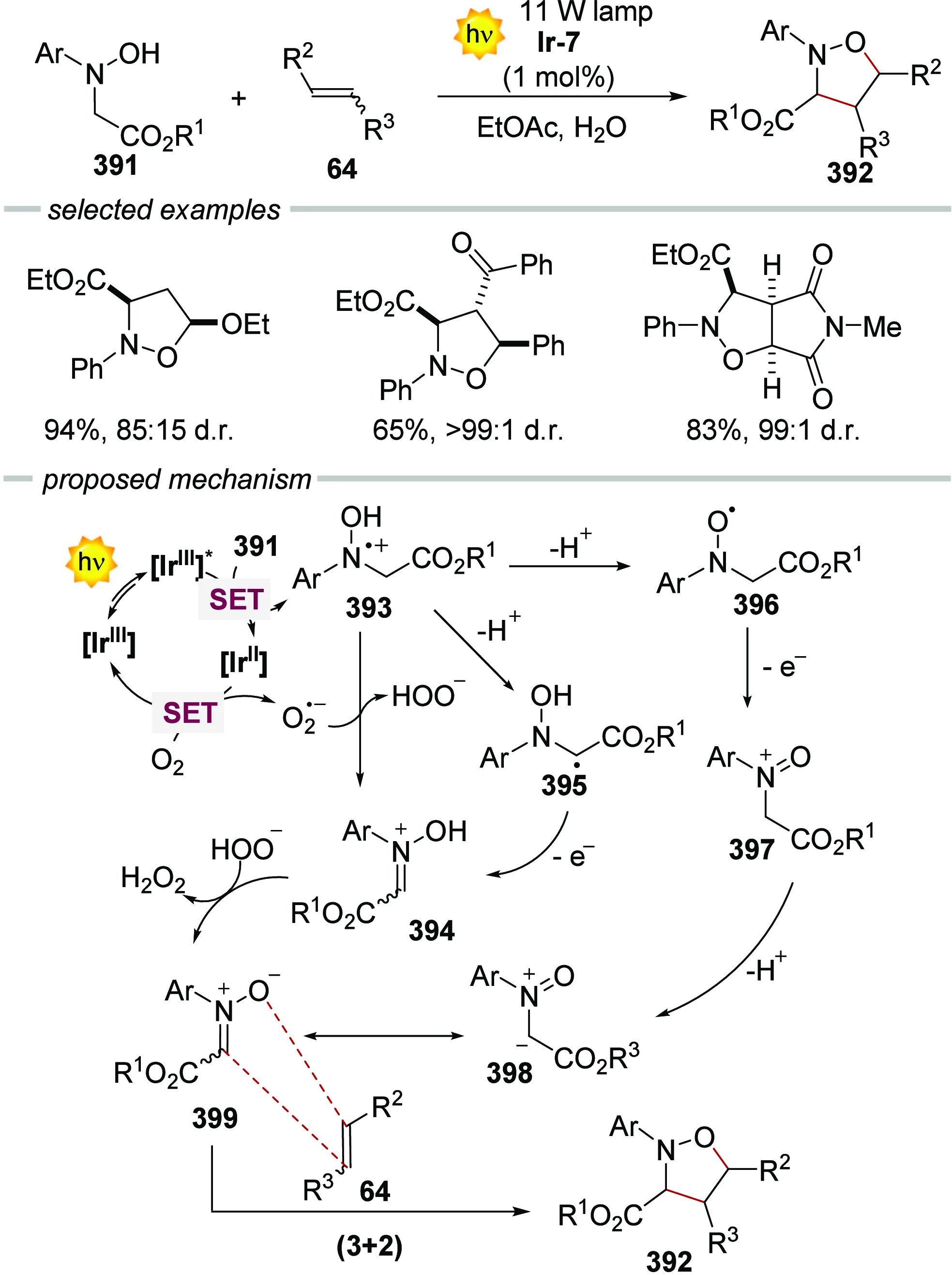
Photocatalytic Synthesis of Isoxazolidines from Arylhydroxylamines and Alkenes

The preceding example illustrates a strategy in which photoredox catalysis is primarily employed for the *in situ* generation of the nitrone dipole. In contrast, a distinct mechanistic paradigm was reported by Xia and co-workers in 2017 (Scheme 80), wherein the photoredox cycle targets activation of the dipolarophile.^352^ In this approach, oxidation of alkene **401** by the photoexcited catalyst Pyr-3* generates a radical cation **403**, which undergoes a stepwise (3 + 2)-cycloaddition with nitrone **400** to form isoxazolidines **402**. Notably, the observed regioselectivity is opposite to that typically obtained under classical thermal Huisgen conditions, thereby providing a complementary synthetic approach. Regarding the formation of product **402**, two mechanistic scenarios were proposed. In the first, the intermediate adduct **405** undergoes reduction by the reduced form of the photocatalyst (Pyr^•^), closing the catalytic cycle. Alternatively, the measured quantum yield value (Φ = 9) suggested the involvement of a chain propagation pathway, in which intermediate **405** oxidizes another molecule of alkene **401**, delivering product **402** while regenerating the radical cation **403**. Supporting this proposal, Stern–Volmer quenching studies revealed that the excited state Pyr-3* is more efficiently quenched by the alkene **401** than by the nitrone **400**, consistent with preferential initial oxidation of the alkene partner. Recently, König and co-workers, developed a similar transformation using alkynes as dipolarophiles to generate 4-isoxazolines.^353^ In this case, **Acr-3** was identified as optimal PC to oxidize the alkyne partner, while generating the corresponding radical cation.

**Scheme 80:**
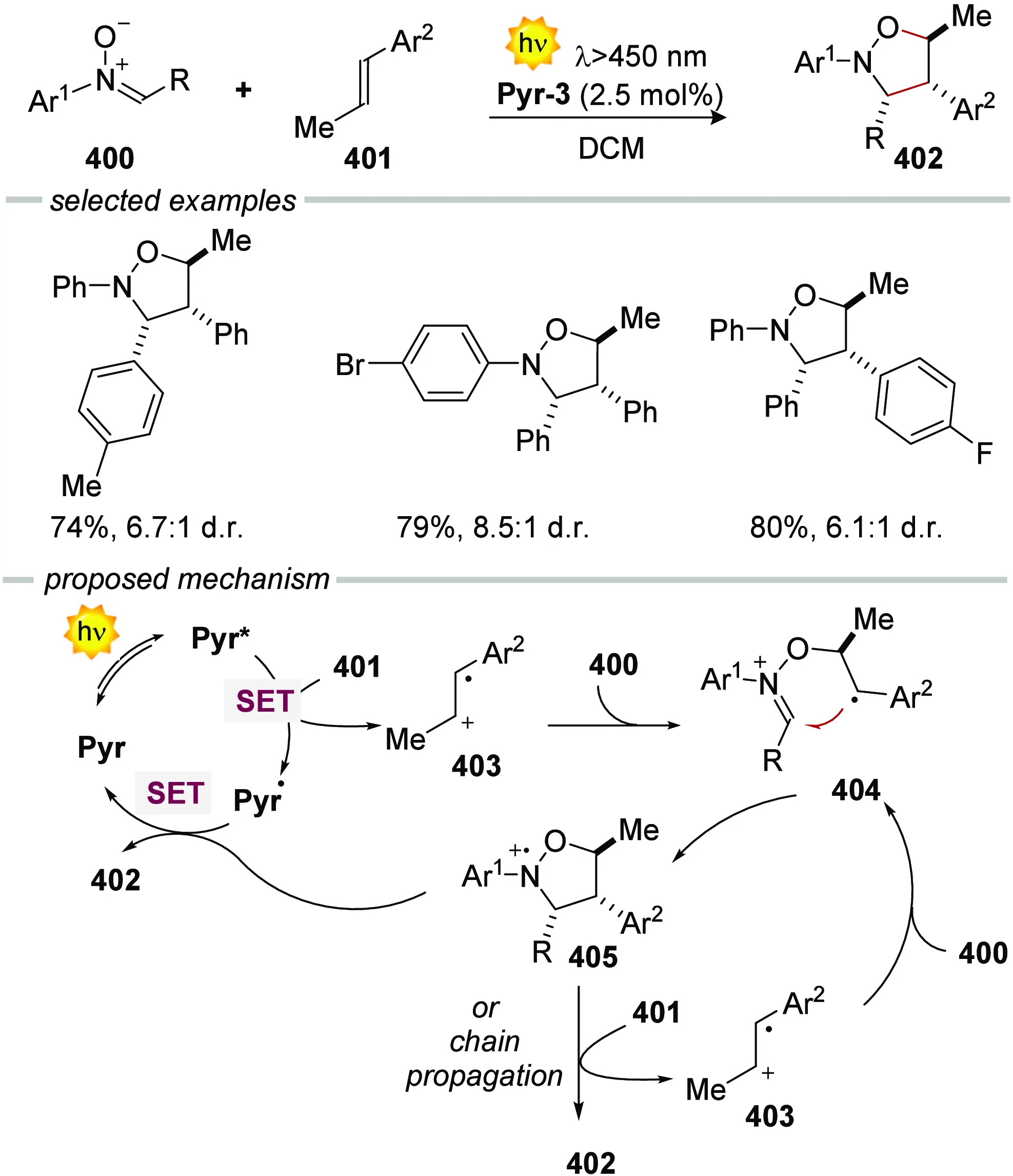
Photocatalytic Synthesis of Isoxazolidines from Nitrones and Alkenes

In 2016, Leonori and co-workers introduced hydroxyimino acids **406**—readily accessible via condensation of α-ketoacids with hydroxylamine—as bench-stable and modular precursors to nitrile oxides (Scheme 81).^354^ Hydroxyimino acids **406** are suited for photoredox activation, as they incorporate two redox-active functionalities—the carboxylic acid moiety and the N–OH group—that can be engaged sequentially en route to the required nitrile oxide. In this context, initial SET oxidation of the deprotonated hydroxyimino acid by the Ru^III^ photocatalyst (**Ru-1**) triggers decarboxylation to generate radical **409**. A subsequent photoredox oxidation furnishes the key nitrile oxide **411**, which undergoes (3 + 2)-cycloaddition with alkenes or alkynes **407** to afford isoxazolines or isoxazoles, respectively. The feasibility of this second oxidation step is supported by the oxidation potentials of related acyl radicals, which fall within the accessible range of Ru­(bpy)_3_^2+^. Mechanistic investigations further corroborate the involvement of nitrile oxide **411** as the reactive intermediate. In particular, Hammett analysis of reactions with *para*-substituted styrenes revealed a V-shaped correlation, consistent with a change in the dominant frontier molecular orbital (FMO) interactions along the substituent series.

**Scheme 81:**
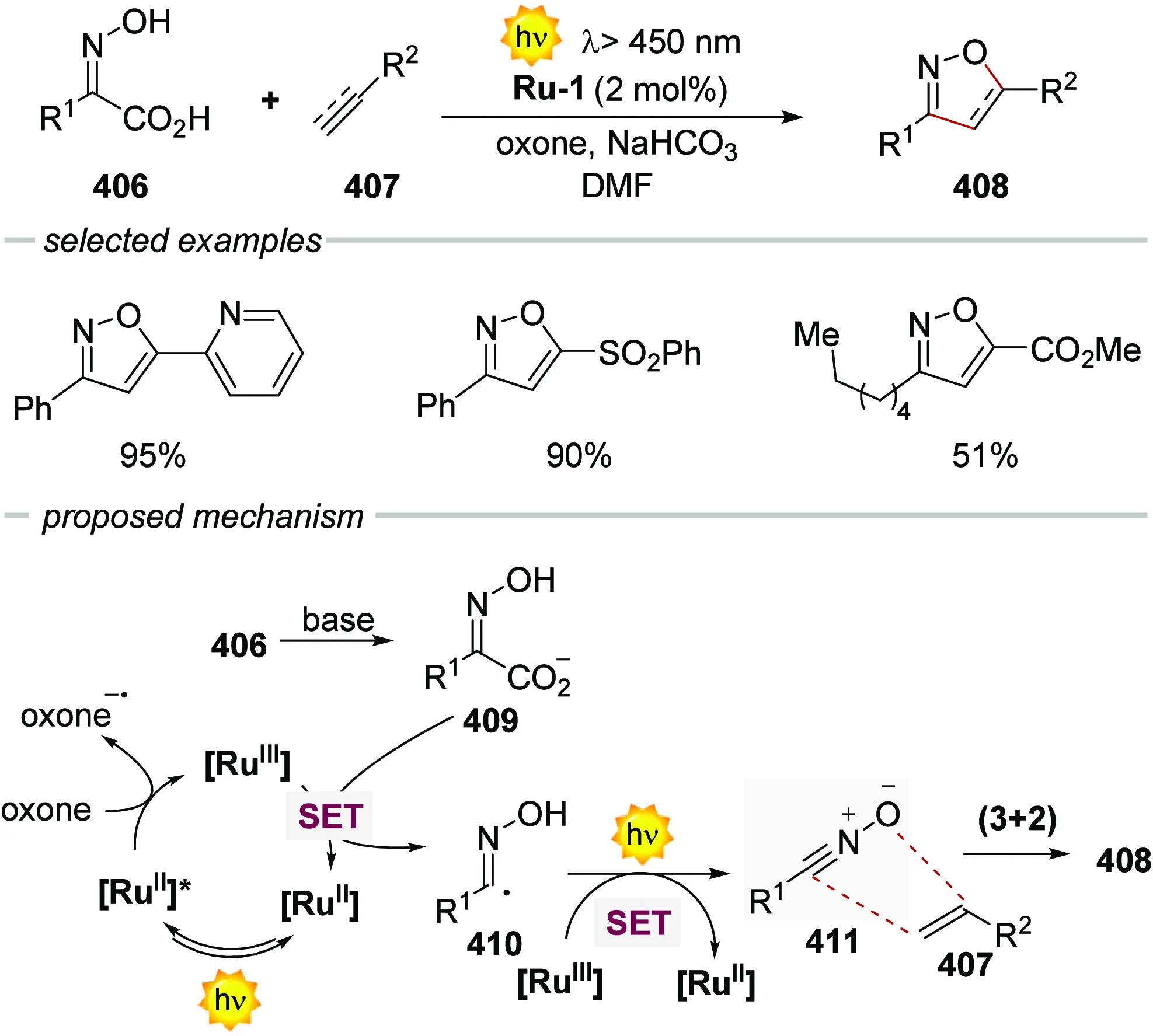
Photocatalytic Synthesis of Isoxazolines and Isoxazoles from Hydroxyimino Acids and Alkenes or Alkynes, Respectively

More recently, Landais and co-workers reported a general photocatalytic strategy for the synthesis of 1,2,4-dioxazoles **413–414** and 1,2-oxazoles **415** from oximes **411** and ketones **412**, nitriles **299**, or alkynes **94**, respectively (Scheme 82).^355^ This protocol employs **4DPAIPN** as PC and O_2_ as the sole oxidant, enabling the *in situ* generation of nitrile oxides as reactive 1,3-dipoles. Mechanistic studies support an initial ground-state association between the photocatalyst and oxime **411** via hydrogen bonding, as evidenced by ^1^H NMR analysis. Upon photoexcitation of **416**, the excited PC-complex **417** engages O_2_ to generate the oxidized species PC^•+^ along with superoxide (O_2_^•–^). Subsequent SET from the oxime to PC^•+^ within the preassociated complex **418** affords the iminoxyl radical **419**, concomitant with regeneration of the ground-state PC. Although PC^•+^ alone lacks sufficient oxidizing power to directly oxidize oximes, H-bonding interactions within **418** are proposed to facilitate this SET process by modulating the electronic density at the hydroxyl group. The resulting iminoxyl radical **419** undergoes HAT with O_2_^•–^ to generate the key nitrile oxide **411**, which then participates in (3 + 2)-cycloaddition with the corresponding dipolarophiles to furnish the heterocyclic products **413–415**.

**Scheme 82:**
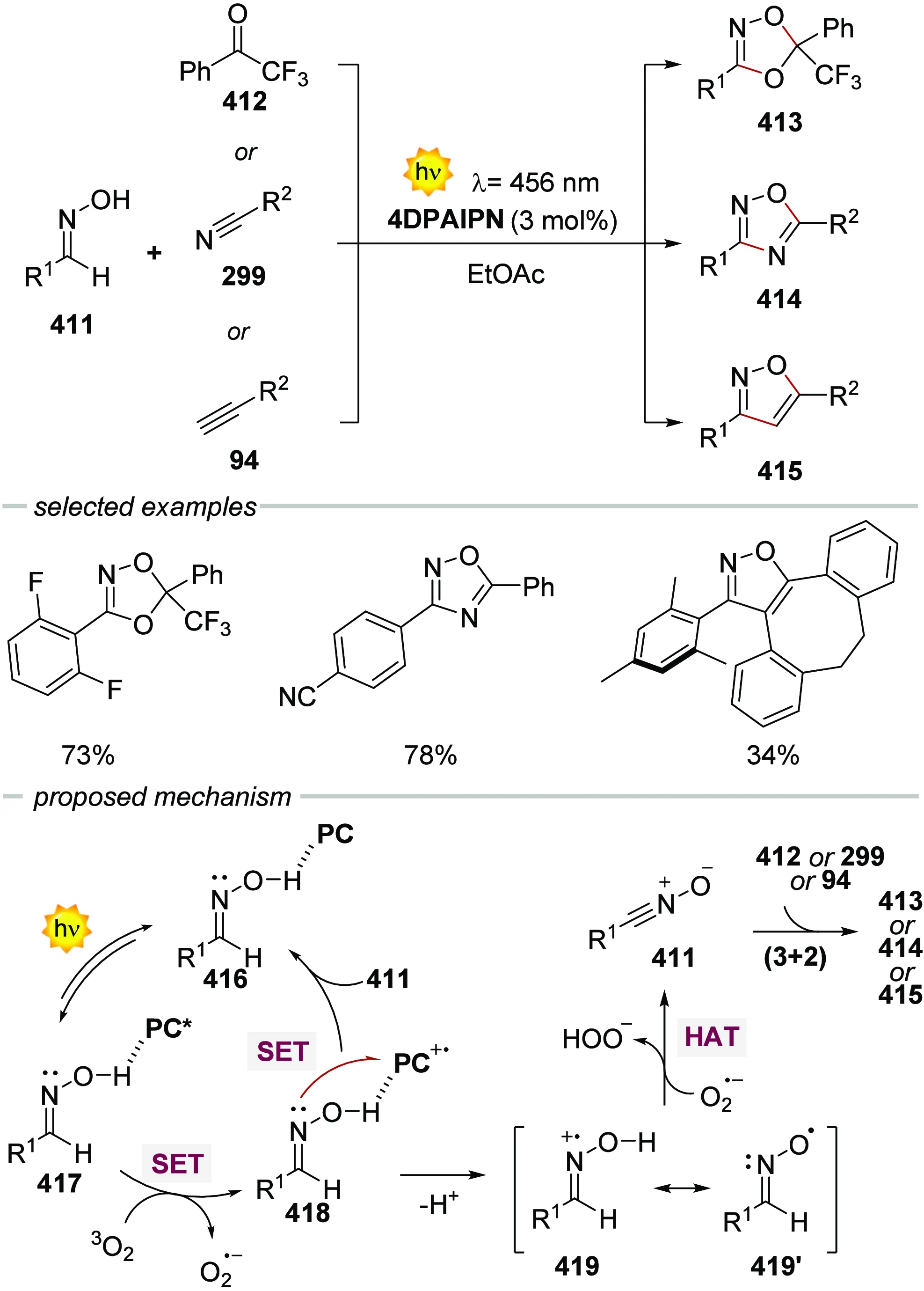
(3 + 2)-Cycloaddition between Oximes and Trifluoroketones; Nitriles or Alkynes

Complementary approaches for the oxidation of oximes to nitrile oxides have also been reported, including the use of carbon nitride-based materials as photocatalysts.^356,357^

#### 4.3.5 Use of Trimethylsilyl Derivatives

In 2019, Lequeux and co-workers demonstrated that readily available *N*-(methoxymethyl)-*N*-(trimethylsilylmethyl)­benzylamine **420** (Achiwa’s reagent) can serve as an efficient precursor for the generation of nonstabilized azomethine ylides under photoredox conditions (Scheme 83).^358^ This appoach enables access to these highly reactive 1,3-dipoles under mild redox neutral conditions, thereby avoiding the need for activation of **420** with strong acids. The formation of the azomethine ylide occurs via SET oxidation of **420** by the photoexcited EY*, affording the radical cation **423.** This intermediate undergoes fragmentation to generate the transient iminium radical **424**. Subsequent reduction furnishes the azomethine ylide **425**, which can be readily intercepted by diverse π-systems **421** to deliver pyrrolidine products **422**. This strategy has since been extended to other dipolarophiles, including azobenzenes^359^ and chiral sulfinamides,^360^ and has also been successfully implemented under continuous-flow conditions.^361^

**Scheme 83:**
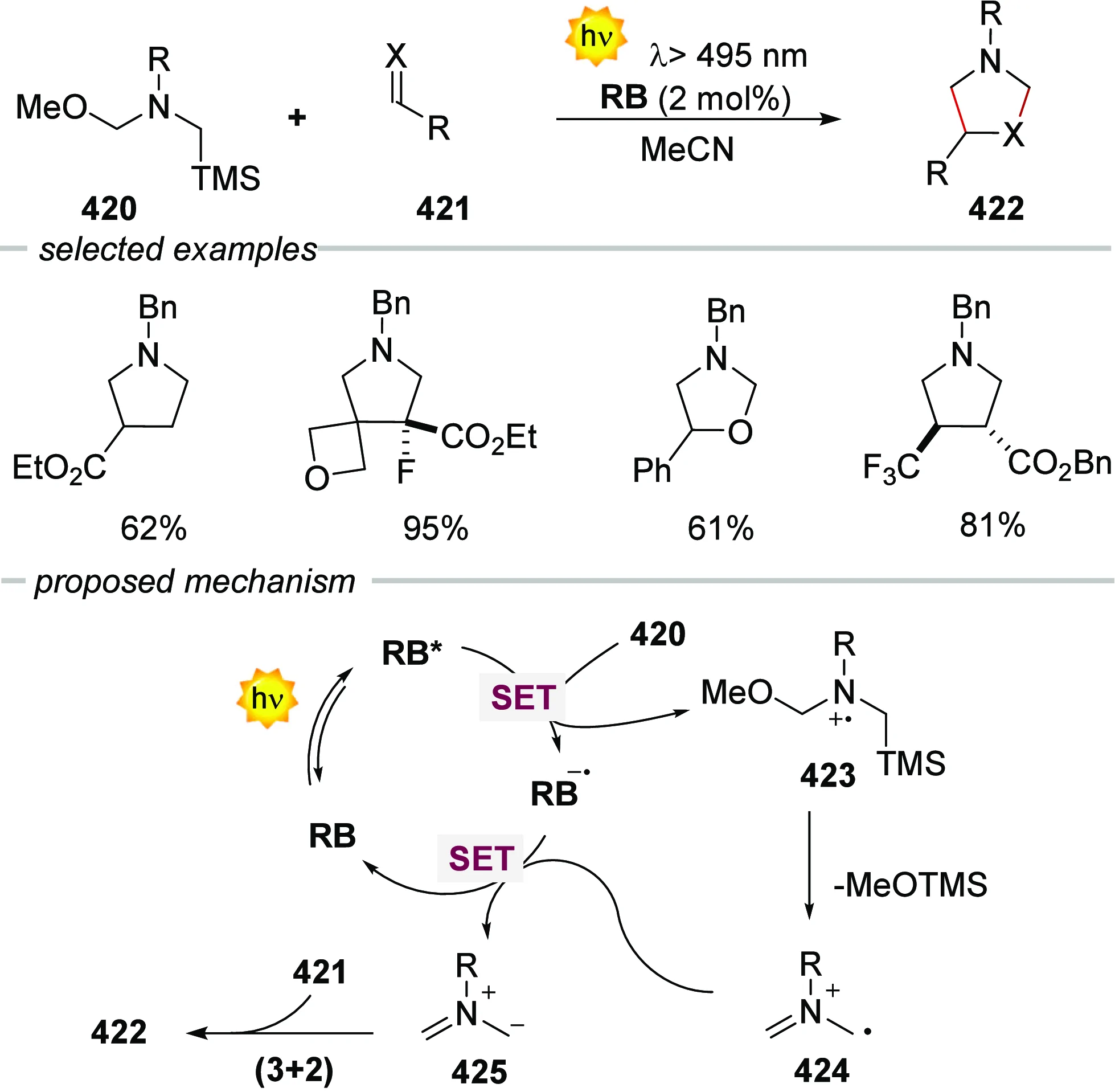
Photocatalytic Synthesis of Pyrrolidines from in Situ Generated Azomethine Ylides and Alkenes

#### 4.3.6 Use of (Hetero)­Arenes

Several strategies have been developed in which aromatic compounds act as 1,3-components in (3 + 2)-hetero-cycloaddition processes with diverse dipolarophiles. A representative example was reported by Yoon and co-workers (Scheme 84).^362^ In this study, phenols **426** were engaged in oxidative (3 + 2)-cycloadditions with alkenes **401** to furnish a range of dihydrobenzofuran derivatives **427**. The reaction employs Ru­(bpz)_3_^2+^ (**Ru-2**) as photocatalyst in combination with ammonium persulfate as terminal oxidant. Interestingly, the reaction displays an induction period accompanied by the formation of an orange precipitate, which was subsequently identified as the catalytically active species. Direct use of this material eliminates the induction period, leading the authors to propose the formation of an insoluble Ru­(bpz)_3_^2+^ persulfate complex *via* slow salt metathesis. Photoexcitation of this complex, followed by oxidative quenching, generates the active oxidant Ru­(bpz)_3_^3+^ species along with a sulfate radical anion. The oxidation of phenol **426** affords the corresponding radical cation **428**, which can undergo further oxidation to generate a resonance-stabilized phenoxonium intermediate **429**. This electrophilic species is then intercepted by electron-rich olefins, ultimately delivering the observed dihydrobenzofuran products **427**. Other metal–organic framework (MOF)-based photocatalysts have also demonstrated efficacy in promoting this transformation.^363^

**Scheme 84:**
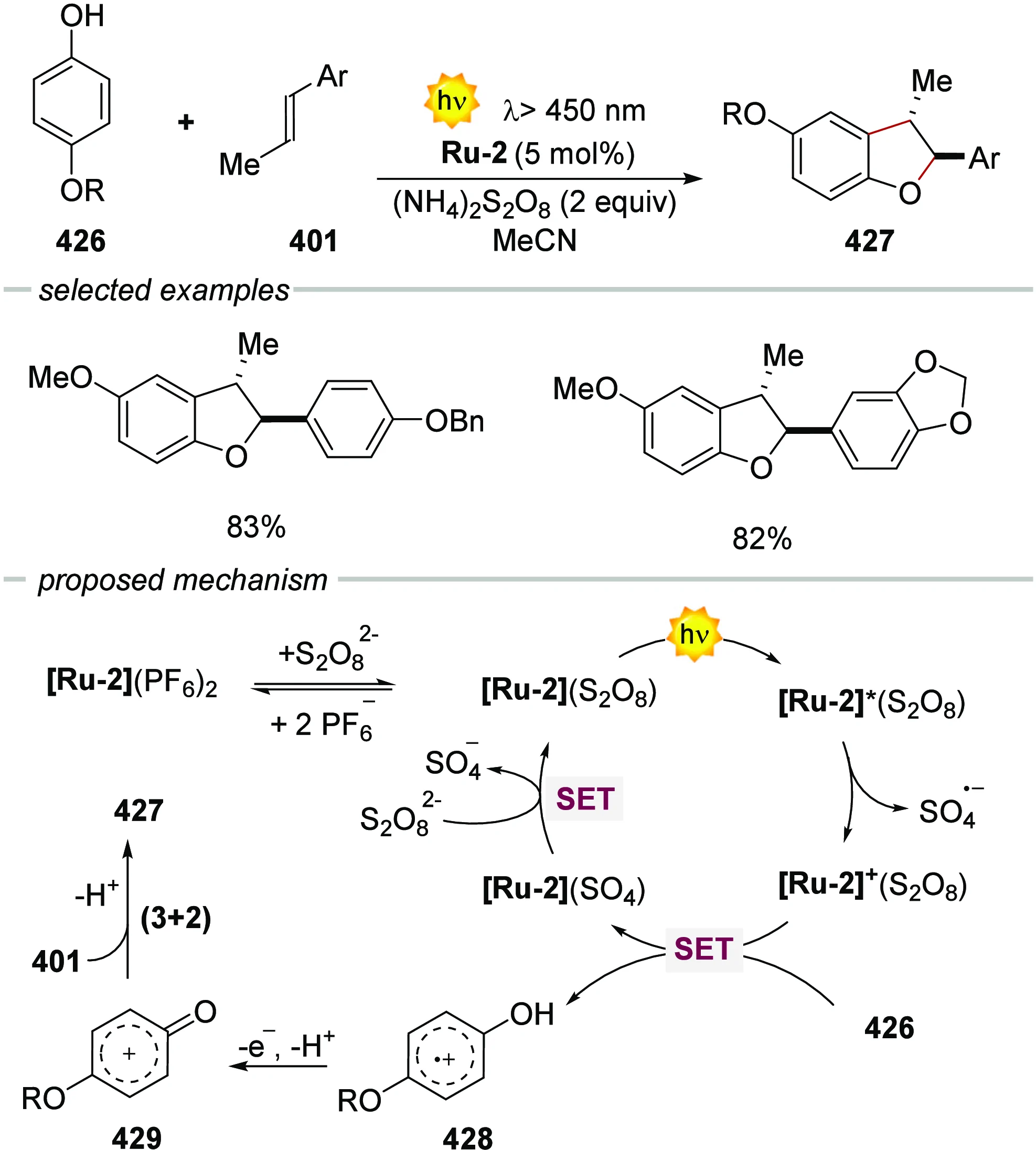
Photocatalytic Synthesis of Dihydrobenzofurans from Electron-Rich Phenols and Alkenes

A related strategy was reported by Zhu and co-workers, who employed thiophenols and alkynes in the presence of an acridinium photocatalyst (**Acr-3**) and molecular oxygen as the terminal oxidant to access benzothiophene derivatives.^364^

In 2020, Dixon, Duarte and co-workers developed a dearomative (3 + 2)-photocycloaddition between *N*-arylimines and quinolines to synthesize bridged 1,3-diazepane frameworks (Scheme 85).^365^ This strategy effectively diverts the conventional Minisci pathway using net reducing conditions, enabling dearomatization and cyclization events. The reaction is initiated by photoredox generation of an α-amino radical **433** from the *N*-arylimine partner **430** via proton coupled electron transfer (PCET) reduction or lewis acid assisted SET. Intermediate **433** undergoes selective addition to quinoline **431**, triggering dearomatization. Subsequent SET reduction, taumerization to form **436** and intramolecular cyclization furnish the bridged 1,3-diazepane products **432**. Computational studies suggested that the high C4-selectivity arises from a fragmentation/recombination manifold that diverts unproductive C2-functionalized intermediates back into the productive pathway, thereby amplifying regioselectivity and suppressing the classical reductive Minisci reaction.

**Scheme 85:**
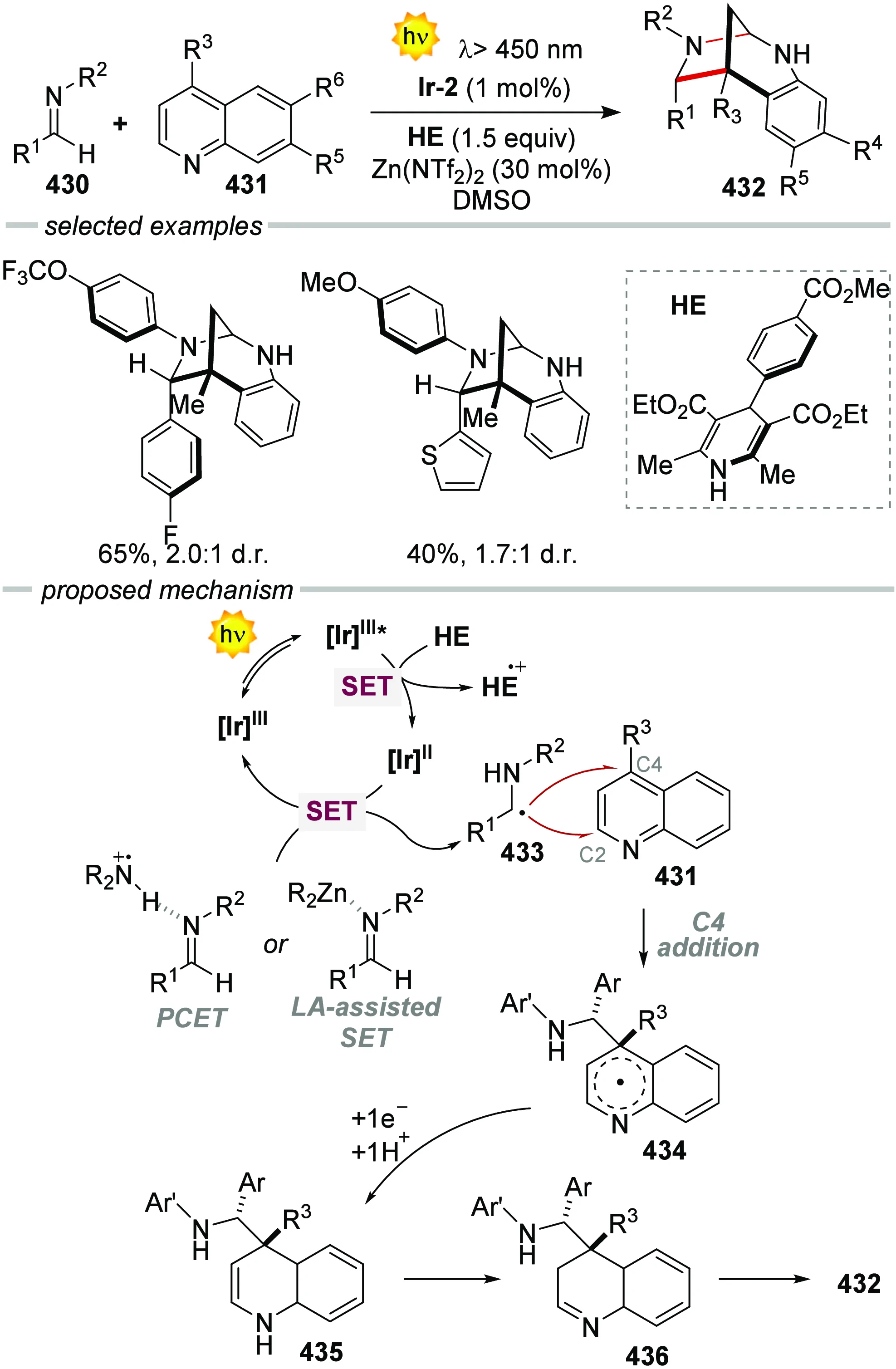
Photocatalytic Synthesis of 1,3-Diazepanes from Imines and Quinolines

More recently, Jiang and co-workers reported an asymmetric dearomative photocycloaddition of isoquinolines enabled by the synergistic combination of photoredox and chiral Brønsted acid catalysis (Scheme 86).^366^ The transformation is initiated by reductive quenching of the photoexcited catalyst **DPZ** by amines **440** or **443**, generating the reduced species DPZ^•–^. Through a PCET process mediated by **439** or **442**, the α,β-unsaturated carbonyl compounds **438** or **441** are reduced to the corresponding enolate radical intermediate **447**, a species whose formation was supported by TEMPO trapping experiments. Protonation of the isoquinoline substrate **437** facilitates regioselective radical addition of **447** *via* a putative ternary transition state **448**, affording the radical intermediate **449.** Subsequent reduction of **449** by **DPZ** ^**•–**^, delivers the corresponding enamine (not shown), which upon tautomerization, undergoes an intramolecular Mannich-type cyclization with the imnium counterpart to complete the annulation sequence.

**Scheme 86:**
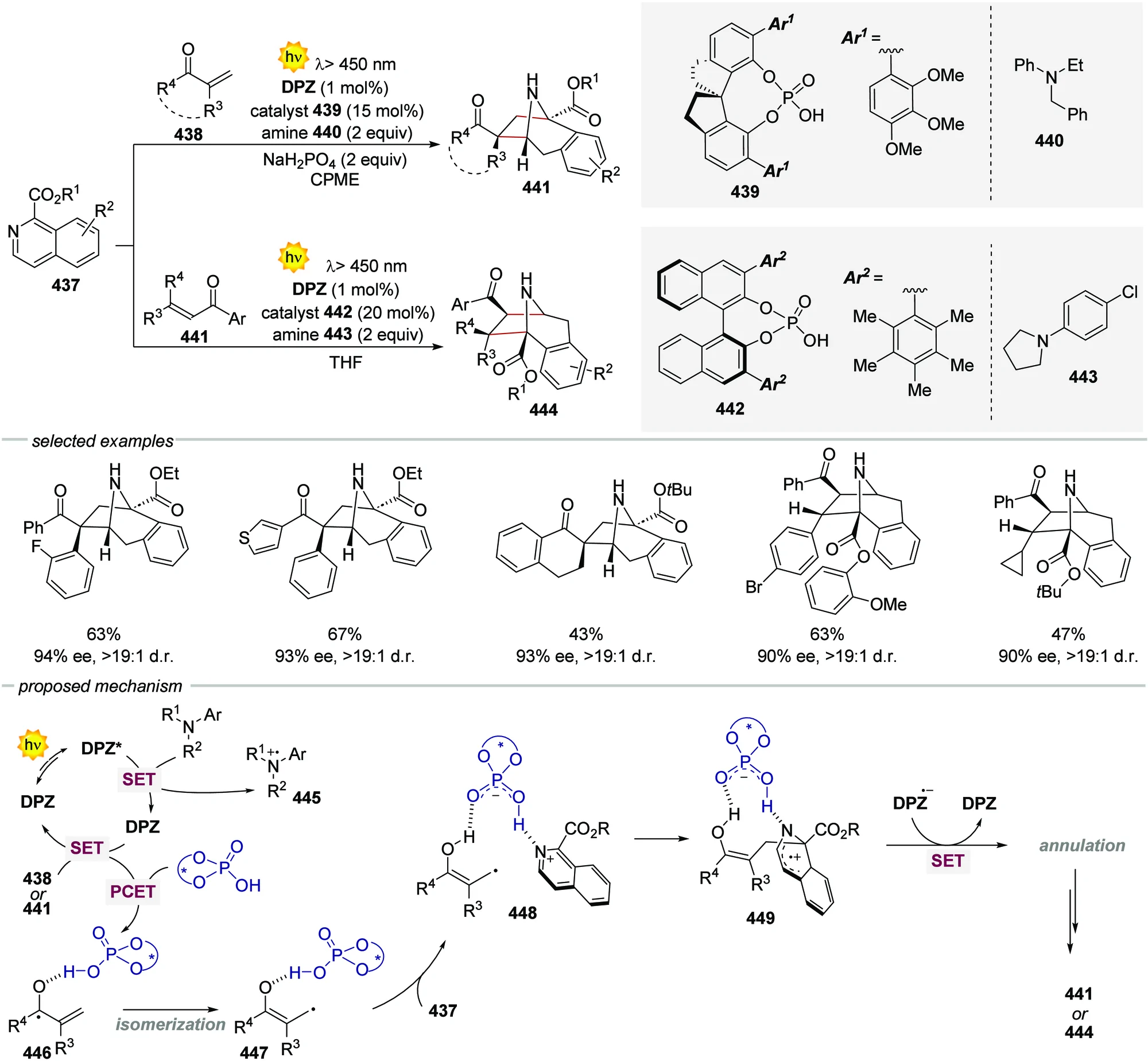
Photocatalytic Synthesis of Tropane Derivatives from Isoquinolines and α,β-Unsaturated Carbonyl Compounds

### 4.4 Azide–Alkyne Cycloadditions

The copper-catalyzed azide–alkyne cycloaddition (CuAAC) is widely regarded as a prototypical click reaction, enabling the synthesis of 1,2,3-triazoles from organic azides and terminal alkynes. Owing to its rapid kinetics, high yields, orthogonality, and tolerance of a broad variety of conditions, CuAAC has been extensively applied across diverse fields, including biomolecular ligation, combinatorial synthesis, medicinal chemistry and materials science.^289^ Typically, the reaction is performed using a catalytic system comprising Cu^II^ salts in combination with a reducing agent, most commonly sodium ascorbate or hydroquinone, to generate the active Cu^I^ species in situ. Alternative strategies based on the electrochemical^367,368^ or photochemical generation of Cu^I^ have been also developed. In particular, photochemical approaches offer the distinct advantage of spatiotemporal control over the initiation of the process, which is especially valuable in biological and materials science applications. In this regard, König and co-workers reported one of the first examples of photocatalytic CuAAc, using riboflavin tetraacetate as a redox active chromophore (Scheme 87a).^369^

**Scheme 87:**
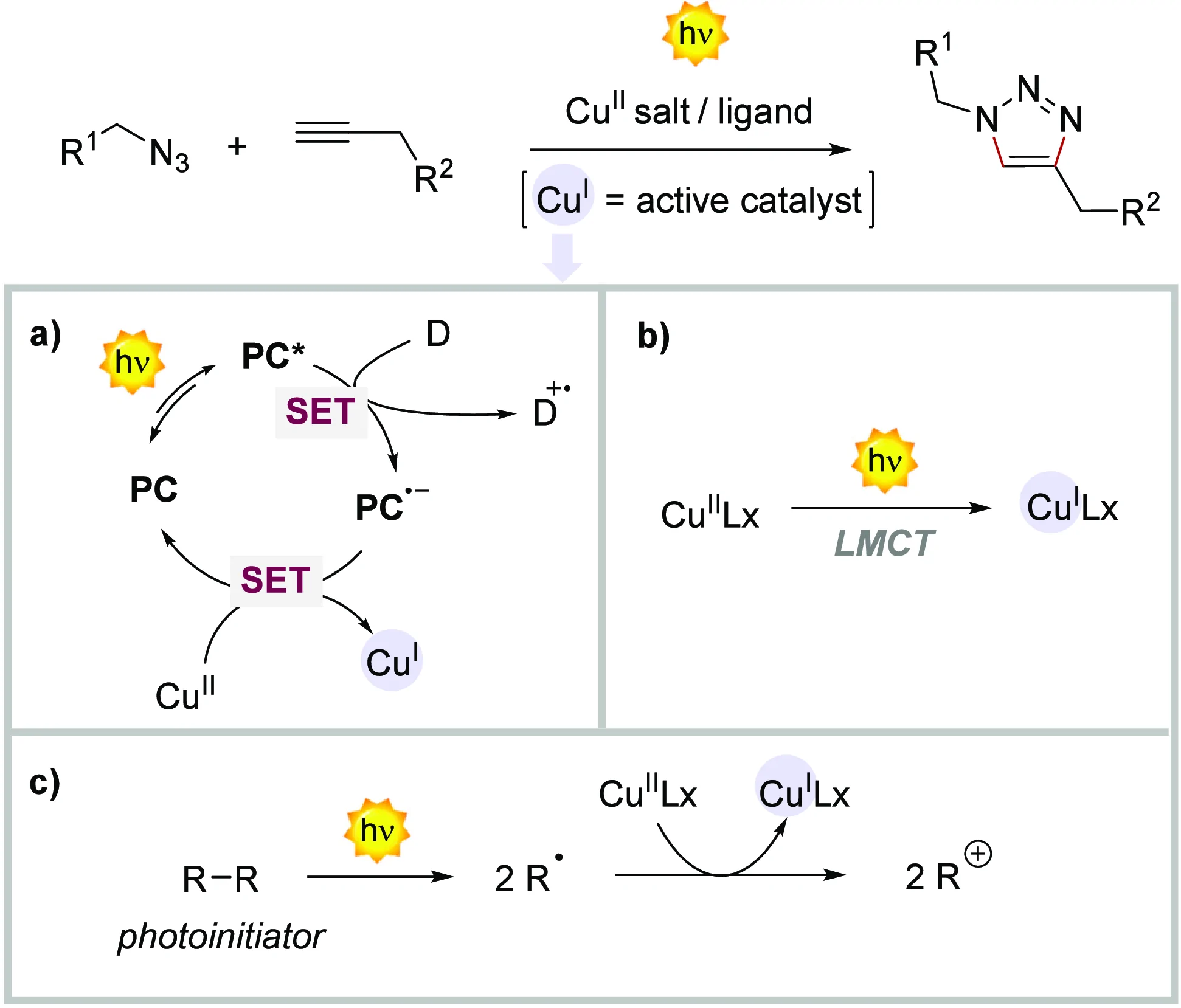
Photocatalytic Strategies for in Situ Cu­(I) Generation in CuAAC Processes

The generation of catalytically active Cu^I^ species can also be achieved through either direct or indirect photolysis of Cu^II^ complexes. In direct photolysis, UV-light is generally absorbed by Cu^II^ complexes bearing ligands that undergo ligand-to-metal charge transfer (LMCT), thereby reducing the metal center (Scheme 87b).^370,371^ In this strategy, tertiary amines are commonly used as ligands. In contrast, indirect photolysis relies on the irradiation of photoinitiators that absorb UV–visible light and generate radical intermediates capable of reducing Cu^II^ to Cu^I^ (Scheme 87c). Several photoinitiators with distinct UV–visible absorption profiles have been successfully used for this purpose, including Irgacure 2959, titanocene, 2-benzyl-2-(dimethylamino)-4′-morpholino­butyrophenone (DBMP), or phenothiazine, among others.^372–374^ In parallel, considerable effort has been devoted to the development of alternative catalytic systems operating under UV or visible-light absorption,^375–379^ which have been reviewed recently.^289^

### 4.5 Miscellaneous

Other classes of substrates have also been identified as precursors of reactive 1,3 or 1,2 π-systems in (3 + 2) cycloaddition processes. For instance, in 2019, Brachet and co-workers reported the use of benzoyl azides **450** for the selective formation of oxazolines **452** under photoredox conditions (Scheme 88).^380^ The proposed mechanism involves triplet energy transfer from the excited-state **Ru-1** complex to the benzoyl azide **450**, generating the corresponding triplet-excited species **450**-**T**_**1**_. This intermediate undergoes nitrogen extrusion to afford the benzoyl nitrene **453**. In the presence of an acid, this nitrene is likely protonated to give intermediate **454**. Subsequent regioselective reaction of the electron-rich alkene with protonated species **454** leads to the formation of the stabilized carbocation intermediate **455**, which upon sequential cyclization and deprotonation furnishes the final oxazoline product **452**.

**Scheme 88:**
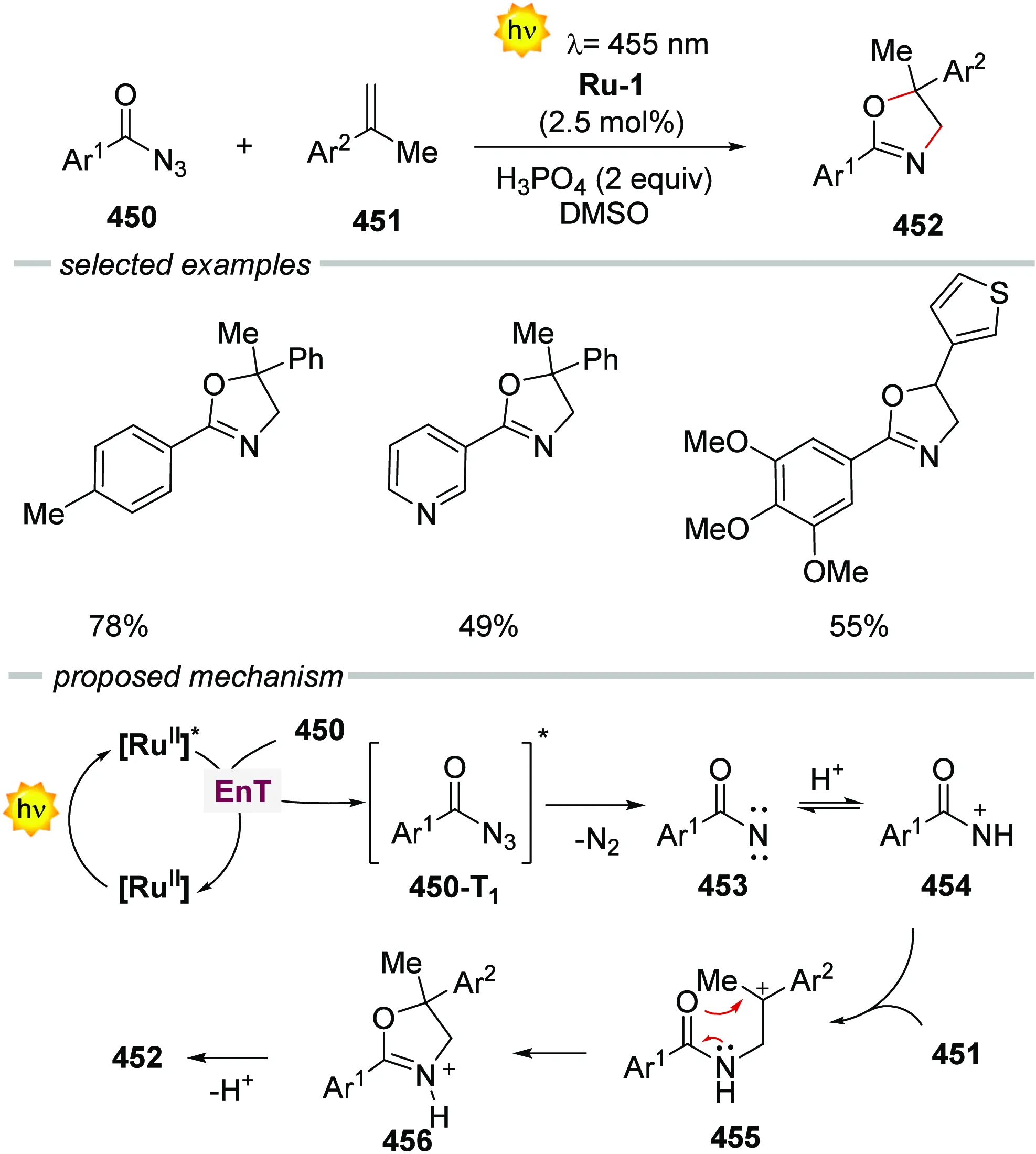
Photocatalytic Synthesis of Oxazolines from Benzoyl Azides and Styrenes

## 5 [4 + 2] Hetero-cycloadditions

[4 + 2] Cycloadditions, involving the reaction of a diene with a dienophile, constitute one of the most extensively studied classes of cycloaddition reactions. Their prominence stems largely from the seminal work of Otto Diels and Kurt Alder, whose discovery of the eponymous Diels–Alder reaction revolutionized synthetic organic chemistry and was recognized with the 1950 Nobel Prize in Chemistry.^381^ When a heteroatom is incorporated in either the diene or the dienophile the hetero-cycloaddition affords heterocyclic products, key structural frameworks in medicinal chemistry and natural products synthesis. Photochemical hetero-Diels–Alder reactions, analogously to other photochemical cycloadditions, relied on direct excitation of unsaturated substrates with high-energy UV light to generate reactive intermediates, such as biradicals or excited-state species, which could subsequently be intercepted by suitable dienes or dienophiles. While effective, these approaches often suffer from limited functional-group tolerance, poor selectivity, and the need for high-energy irradiation. In recent years, however, the rapid emergence of photocatalytic strategies, including photoredox and EnT catalysis, has fundamentally transformed also this area. These methodologies provide efficient access to analogous or entirely new reactive intermediates under mild visible-light irradiation, thereby greatly expanding the scope of photochemical hetero-cycloadditions. As a result, a wealth of innovative hetero-[4 + 2] cycloaddition protocols have been developed, enabling the efficient synthesis of structurally diverse oxygen- and nitrogen-containing heterocycles. The examples discussed in this section are classified according to the type of heterocycle formed, between oxygen-containing, nitrogen-containing, and mixed *N,O*-containing heterocycles. Within each category, the reactions are further organized based on the activation mode, namely direct photoexcitation or photocatalyst-mediated activation (Scheme 89).

**Scheme 89:**
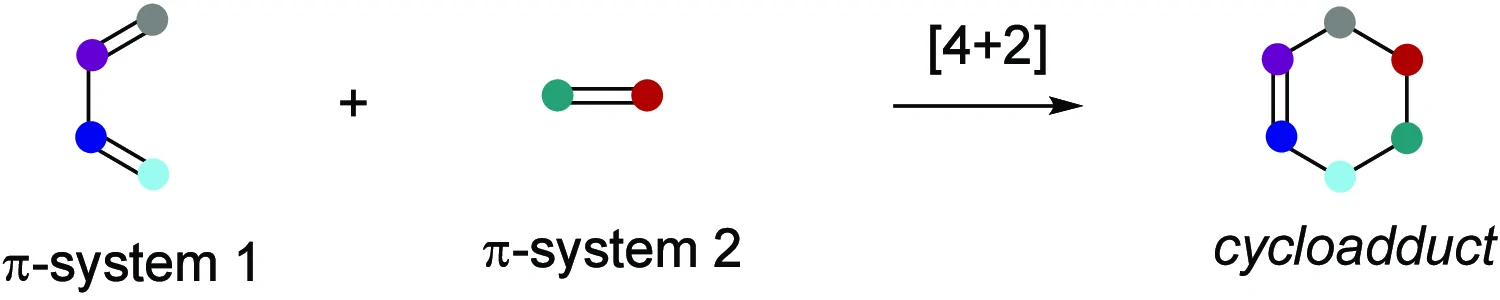
General [4 + 2] Cycloaddition of a Diene with a Dienophile for the Synthesis of Six-Membered Heterocycles

### 5.1 Oxygen-Containing Heterocycles

#### 5.1.1 Direct Irradiation Methods

In 2008, Bochet et al. report the intramolecular [4 + 2] photocycloaddition of *ortho*-allenyl benzaldehydes under UV irradiation, delivering mixtures of two distinct products (**458**, **459**) depending on the electronic/steric nature of the substituents on the arene ring (Scheme 90).^382^ Formation of benzoxepines **458** is rationalized as a sequence comprising an intramolecular hetero-Diels–Alder cycloaddition, in which the aldehyde acts as diene and the allene as dienophile, followed by a formal 1,3-oxygen-to-carbon sigmatropic rearrangement in order to restore the aromaticity of the phenyl ring.

**Scheme 90:**
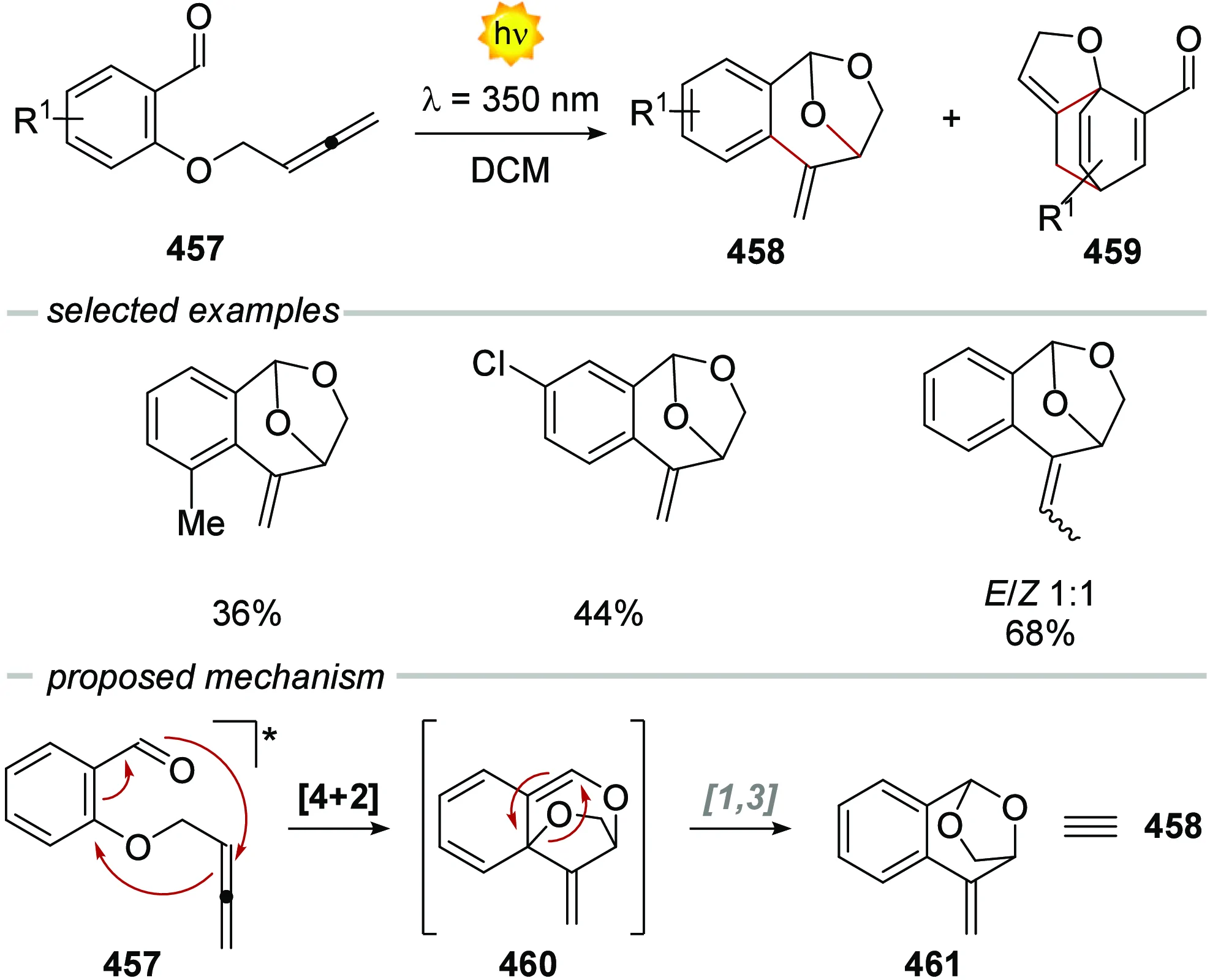
Photochemistry of Allenyl Salicylaldehydes

Popik et al. reported a light-induced hetero-Diels–Alder ″photoclick″ reaction based on the photochemical generation of *o*-naphthoquinone methides (*o*-NQMs) from 3-(hydroxymethyl)-2-naphthol precursors **462**, and their subsequent cycloaddition with electron-rich polarized alkenes, such as vinyl ethers or enamines to afford benzochromans in high yields under aqueous conditions (Scheme 91).^383^ The reactive *o*-NQM intermediate is generated upon light absorption (300–350 nm) of the diol precursor **462** and it is quickly intercepted by electron rich alkenes **463**. The very short lifetime (τ ≈ 7 ms) of the reactive intermediate is due to the hydration back-reaction leading to the starting material, thus leading to high chemical yields and avoiding undesired decomposition pathways. This study becomes particularly relevant in the context of bioconjugation as it establishes a “photoclick” platform based on a hetero-Diels–Alder cycloaddition.

**Scheme 91:**
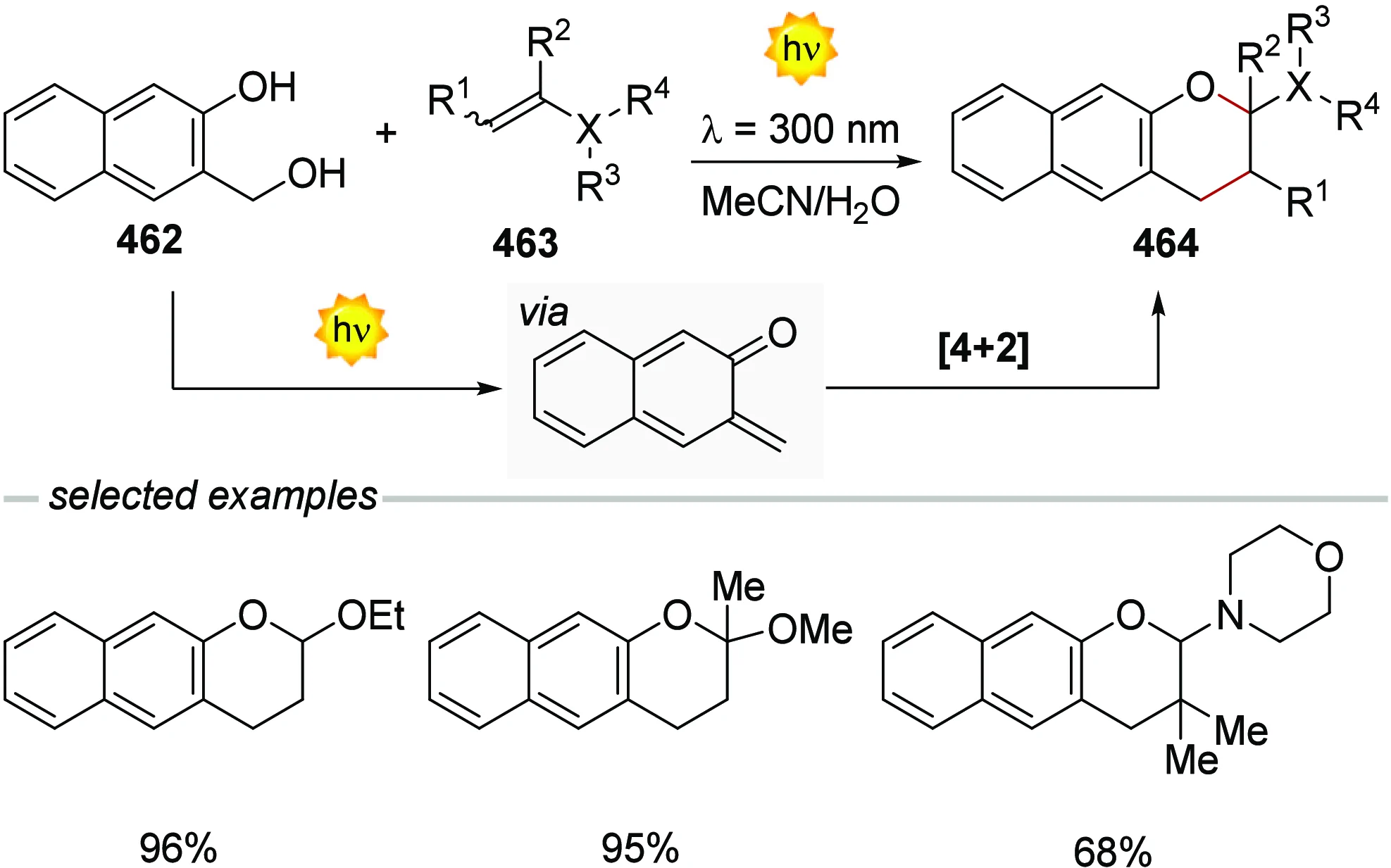
Photochemical Hetero-Diels–Alder Reaction via Ortho-Naphthoquinone Methides

#### 5.1.2 Photocatalytic Methods

In 2011, Yoon et al. reported a visible-light photocatalytic intramolecular hetero-Diels–Alder cycloaddition of tethered bis­(enones) **465**, employing **Ru-1** as the photoredox catalyst to obtain dihydropyran products with high diastereoselectivity (Scheme 92).^384^ Notably, this method overcome a limitation of classical hetero-Diels–Alder chemistry: the requirement for electronic matching between diene and dienophile. Here, two electron-deficient partners are coupled efficiently through single-electron reduction, a manifold inaccessible under thermal activation. The authors of this work propose a stepwise radical anion pathway where photoexcitation of **Ru-1** followed by reductive quenching by a sacrificial amine (*i*Pr_2_NEt) generates the strong reductant [Ru^I^], which transfers one electron to a Lewis acid-activated enone **467** to produce an enone radical anion **468**. This species undergoes β,β carbon–carbon bond formation to give a monocyclized radical anion intermediate **469**, which cyclize onto the carbonyl oxygen to give bicyclic intermediate **470**. The regioselectivity of the process in unsymmetrical substrates is dictated by preferential stabilization of the resulting ketyl radical after the final cyclization step (e.g., R^1^ = Ph and R^2^ = Me). The neutral cycloadduct is released upon single-electron oxidation, either via chain propagation to another enone equivalent **467** or via chain termination involving the amine radical cation. Interestingly, the length of the tether plays a remarkable role in determining the reaction outcome as compared to previous studies.^385^

**Scheme 92:**
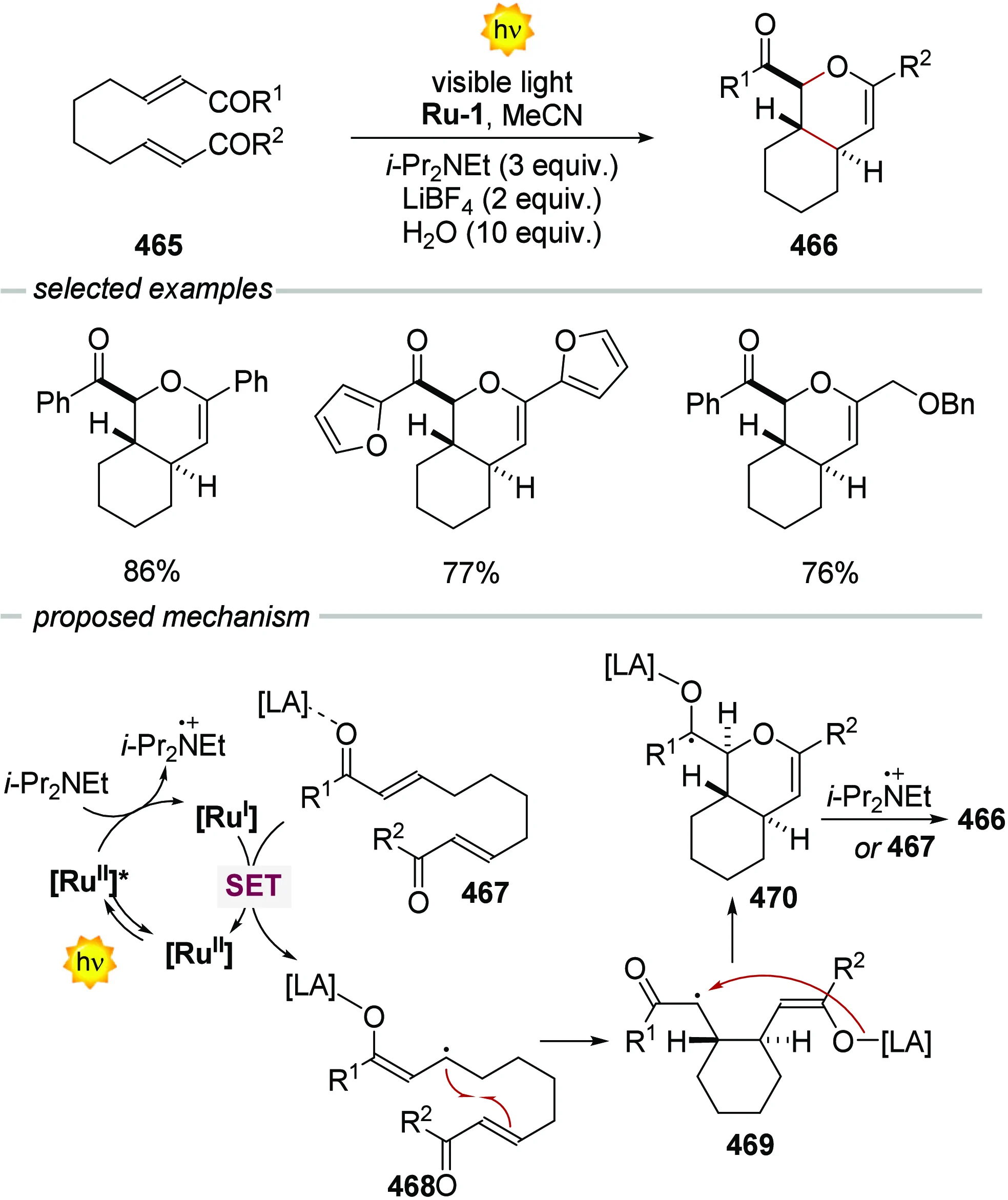
Photocatalytic Intramolecular Hetero-Diels–Alder Reaction

Shortly after, the research group of Bach reported an enantioselective photocatalytic [4 + 2] cycloaddition between 3-substituted 2-pyridones **471** and singlet oxygen through the use of chiral hydrogen-bonding template **472** (Scheme 93).^386^ Overall, this process involves a two-step sequence combining the hetero-Diels–Alder cycloaddition with an acid-catalyzed Kornblum–DeLaMare rearrangement of the transiently formed endoperoxide.^387^ From a mechanistic point of view, singlet oxygen is generated upon sensitization with *meso*-tetraphenylporphyrin (**TPP**) under visible light irradiation. Then, the reactive species undergoes [4 + 2] cycloadditions with the 2-pyridone substrate affording a bicyclic endoperoxide intermediate in an enantioselective manner because of a 1:1 complex between the pyridone derivative and the chiral bicyclic lactam template. Alternatively, Toste et al. developed a similar methodology that focuses on the enantioselective base promoted Kornblum–DeLaMare rearrangement of *meso*-endoperoxides, mediated by cinchona-alkaloid derivatives, to deliver optically active γ-hydroxyenones.^388^

**Scheme 93:**
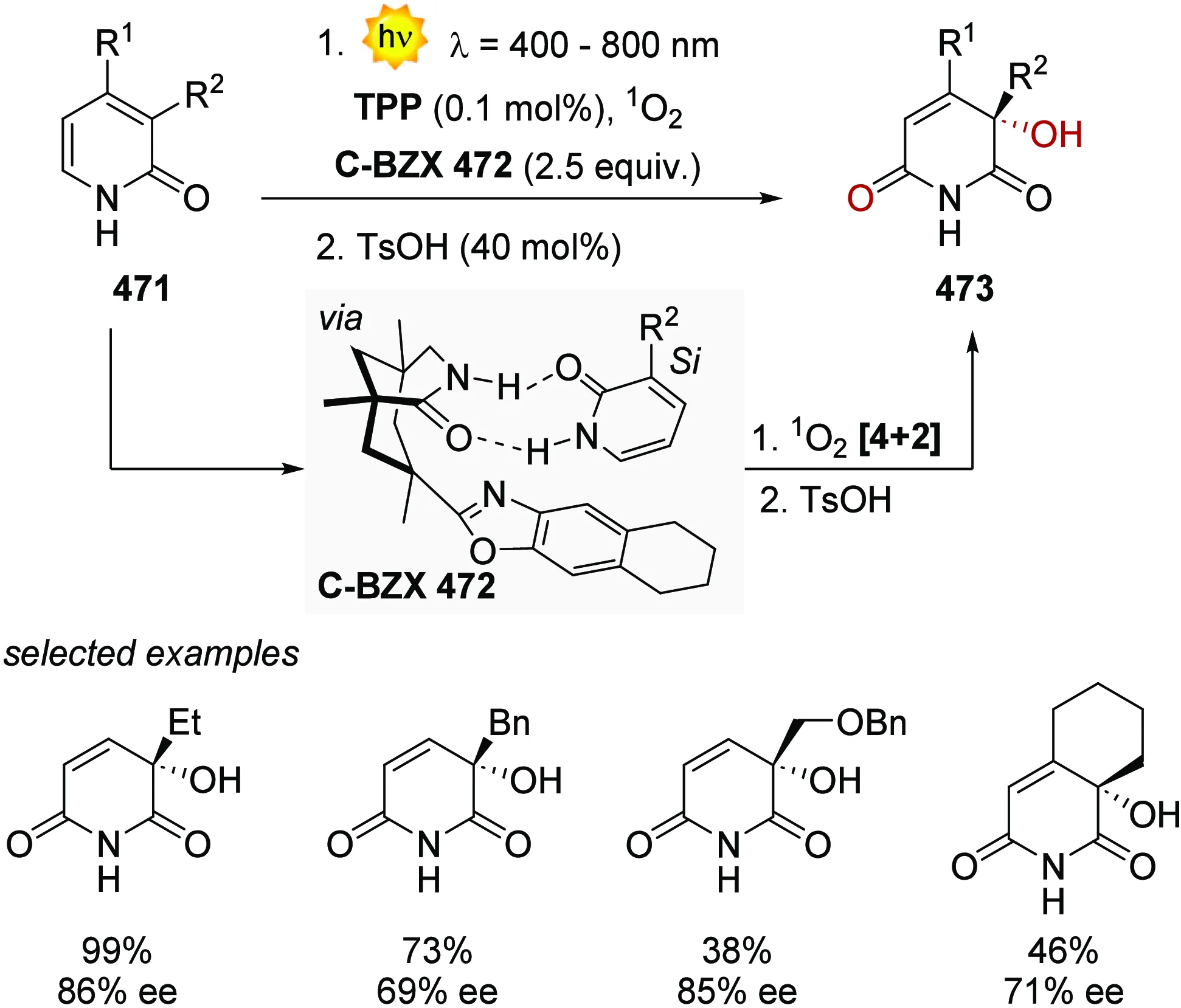
Enantioselective Visible-Light Induced Singlet Oxygen [4 + 2] Cycloadditions with Pyridines

More recently, de Oliveira and co-workers reported a continuous-flow methodology for the photocatalytic [4 + 2] cycloaddition of conjugated dienes **474** with singlet oxygen, generated *in situ* by **TPP** sensitization, followed by base-mediated Kornblum–DeLaMare rearrangement of the resulting endoperoxide intermediates (Scheme 94).^389^ The adoption of flow conditions was primarily motivated by practical and safety considerations, as it enables precise control over residence time, efficient gas–liquid mixing, and safe handling of potentially explosive endoperoxides and flammable solvent–oxygen mixtures, while simultaneously improving reproducibility and facilitating scale-up relative to conventional batch protocols. The use of this method facilitated the synthesis of a variety of valuable oxygenated building blocks including hydroxyenones, substituted furans, 1,4-dicarbonyl compounds, and tropone, among others.

**Scheme 94:**
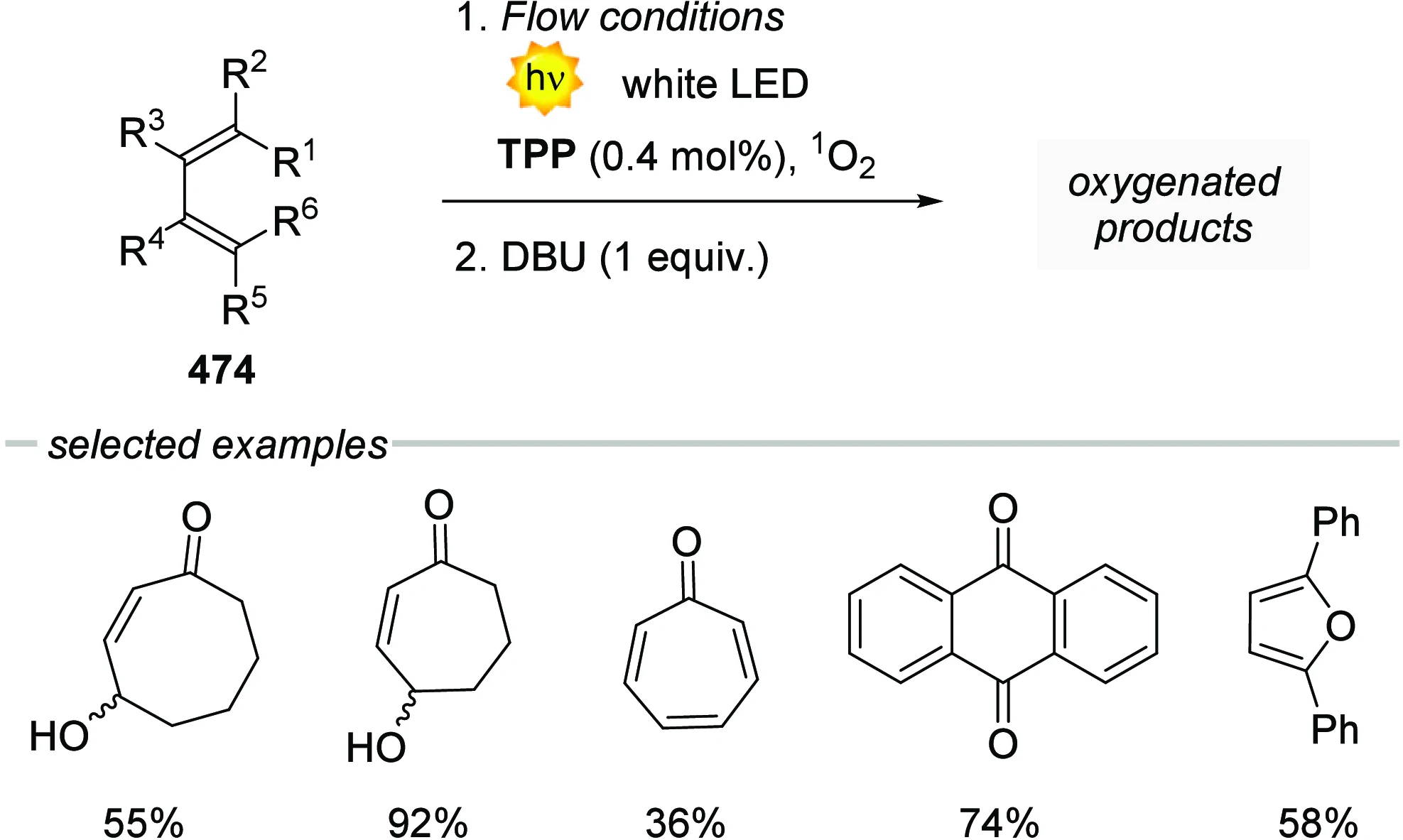
Photocatalytic Singlet Oxygen [4 + 2] Cycloadditions under Flow Conditions

The group of Hoshino, Honda, and Tanaka reported two methodologies based on the thioxanthylium (**TXT**) organophotoredox-catalyzed [4 + 2] cycloaddition of alkenes or pentafulvenes with *ortho*-quinone methides under green light irradiation at room temperature, affording tetrahydrocyclopenta­[*b*]­chromenes in good yields and moderate to good values of diastereoselectivity (Scheme 95).^390–392^

**Scheme 95:**
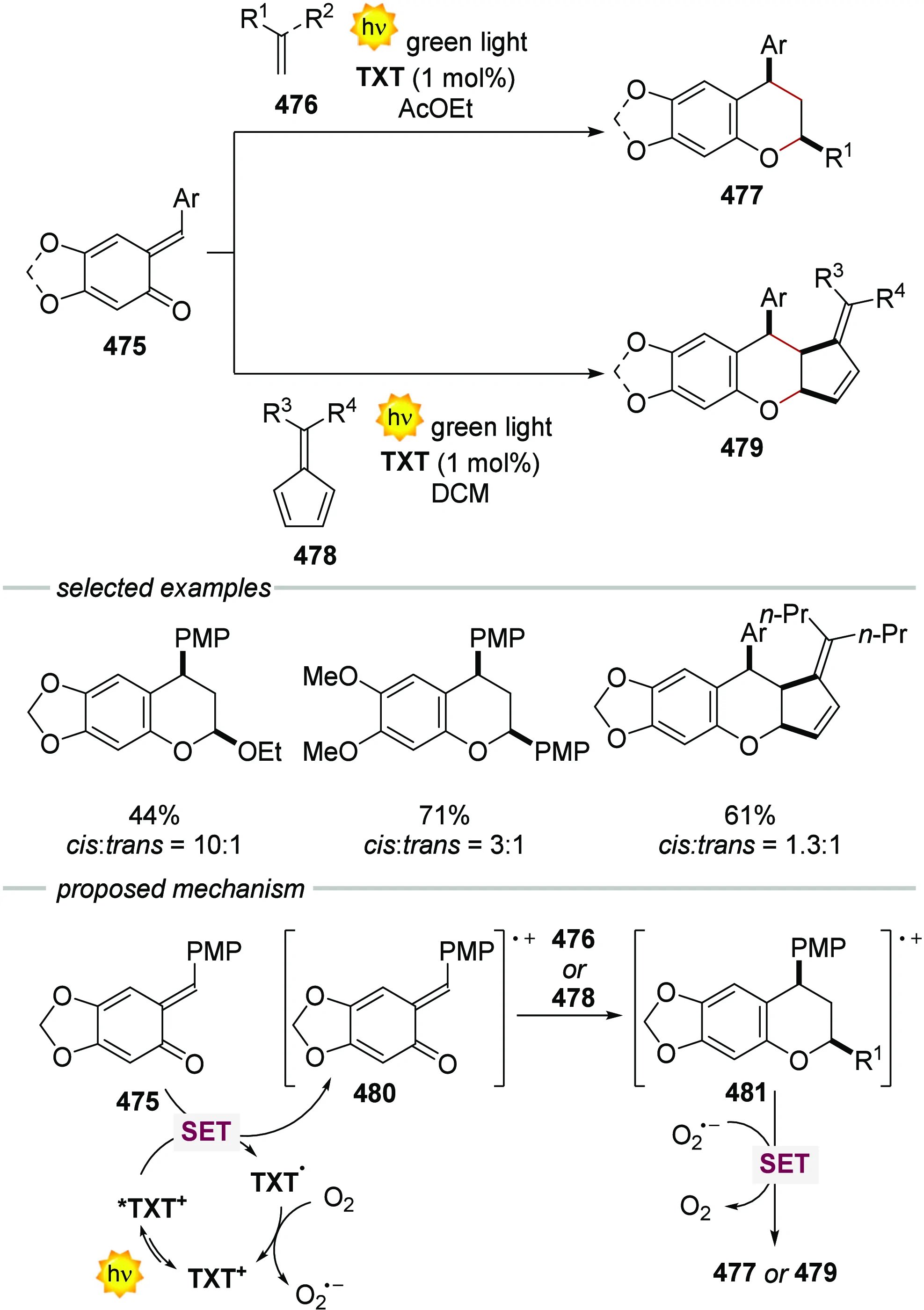
Photocatalytic [4 + 2] Cycloadditions for the Synthesis of Chromane Derivatives

In this method the excited **TXT** photocatalyst oxidizes the *ortho*-quinone methide **475** via single electron transfer, generating the corresponding radical cation **480**, as confirmed by Stern–Volmer quenching experiments. This radical cation undergoes stepwise [4 + 2] cycloaddition with the pentafulvene dienophile to yield distonic radical cation **481**. Catalyst turnover is achieved by single-electron reduction of **481** by superoxide (O_2_^•–^), which is generated *in situ* by oxidation of the reduced photocatalyst (**TXT** ^**•**^) by molecular oxygen. The measured quantum yield (Φ = 0.15) indicates that the reaction proceeds predominantly *via* a closed photocatalytic cycle rather than a radical chain propagation mechanism.

### 5.2 Nitrogen-Containing Heterocycles

#### 5.2.1 Direct Irradiation Methods

Benzene derivatives possess multiple unsaturated π-bonds that could, in principle, engage in cycloaddition reactions, making them highly attractive synthetic building blocks. Nevertheless, the exceptional thermodynamic stability associated with aromaticity poses a major challenge to their dearomatization. The loss of aromatic stabilization typically results in elevated kinetic barriers and products that are thermodynamically less favored than the parent aromatic compounds. As a consequence, dearomatization reactions often exhibit limited efficiency and are prone to thermal reversion to the aromatic starting materials. Nonetheless, reagents that are able to overcome these limitations preferentially react with the partially dearomatized products, often resulting in poor yield and selectivity.^393^

In this context, Sarlah et al. reported, inspired by the seminal work of Hamrock and Sheridan,^29,394^ a two-step dearomatization strategy based on the activation by visible light of “arenophile” 2π components. Notably, the photochemical activation of the arenophile effectively overcomes many of the challenges traditionally associated with the photochemistry of benzene derivatives. In particular, it avoids the need for high-energy UV irradiation and mitigates the selectivity issues arising from the complex excited-state reactivity of arenes, which often leads to multiple competing reaction pathways. The key aspect of this strategy was the selection of the proper arenophile 4-methyl-1,2,4-triazoline-3,5-dione (MTAD, **483**), whose narrow HOMO–LUMO gap allows selective visible-light excitation without exciting the arene **482**, therefore engaging in a *para*-cycloaddition at −78 °C. The thermal lability of intermediate **484** requires *in situ* trapping by complementary transition-metal catalytic manifolds, delivering stable dearomatized products **485** or **486** (Scheme 96).^395^ From the common bicyclic intermediate **484**, a range of divergent derivatization pathways can be accessed. For instance, two distinct catalytic protocols were developed for the *in situ* oxygenation of the cycloadducts. Dihydroxylation under catalytic OsO_4_ in the presence of 4-methylmorpholine *N*-oxide (NMO) afforded diol **487**, whereas application of a modified Narasaka–Sharpless protocol enabled the synthesis of the corresponding boronic ester **488**.^396^ Furthermore, urazole hydrolysis followed by CuCl_2_-mediated oxidative N_2_ extrusion affords acetonide-protected 3,4-dihydrodiols **489**; alternatively, urazole hydrolysis, benzoylation and SmI_2_-mediated reductive N–N cleavage delivers diaminodiols **490** bearing four heteroatom-containing stereocenters. The reported strategy was then extended to site-selective functionalization of polynuclear arenes using a two-step protocol that involves MTAD cycloaddition/dihydroxylation and urazole hydrolysis/catalytic hydrogenation to afford a series of diaminodiols such as **491**. Furthermore, enantioenriched *trans*-1,2-carboaminated product **492**.^397^ was obtained using a Grignard reagent in the presence of a palladium complex and resulted to be a key intermediate for the synthesis of (+)-7-deoxypancratistatin.^398^ Finally, the enantioselective synthesis of *syn*-1,4-oxyaminated product **493** ^399^ or *syn*-1,4-diaminated product **494**.^400^ was accomplished by using oxygen- or nitrogen-based nucleophiles under similar reaction conditions.

**Scheme 96:**
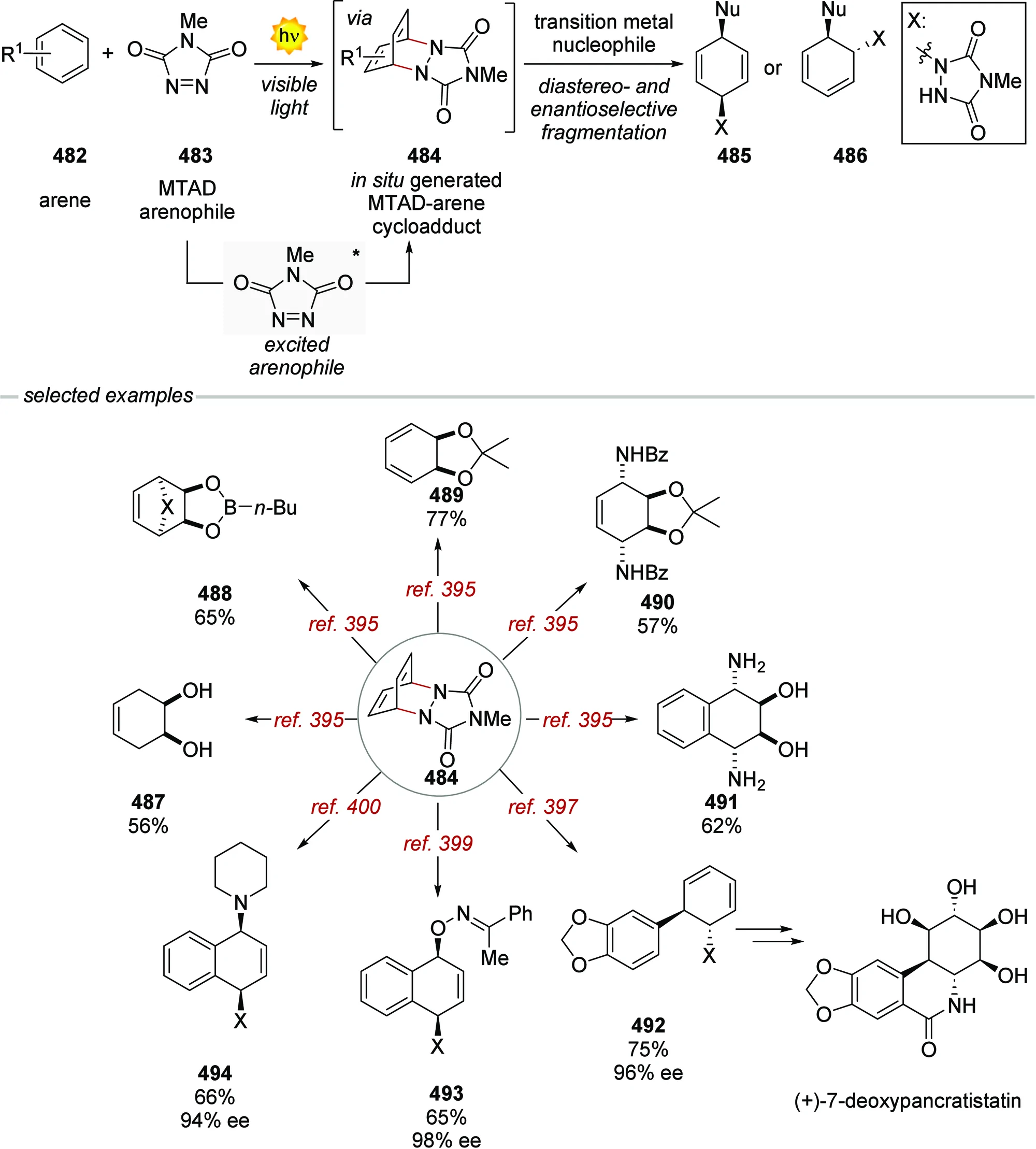
Visible-Light Activation of MTAD for the Dearomatization of Arene Derivatives via [4 + 2] Cycloadditions Followed by Transition Metal Catalyzed Functionalization of Labile Intermediate 484

More recently, the study of Yoshino, Matsunaga et al. introduces 1,2-dihydro-1,2,4,5-tetrazine-3,6-dione derivatives (TETRAD, **496**) as novel arenophiles for the visible-light-induced dearomative [4 + 2] cycloaddition of nonactivated aromatic compounds (Scheme 97).^401^ Unlike MTAD-derived cycloadducts, which are highly thermally labile, the resulting TETRAD adducts can be isolated, purified, and fully characterized. Compared to the previous examples, the improved stability of the TETRAD-cycloadducts allows for their further manipulations at room temperature or above. A broad substrate scope was demonstrated, including substituted benzenes, naphthalenes, quinolines, quinazolines, quinoxalines, acridines, and benzo­[*h*]­quinolines.

**Scheme 97:**
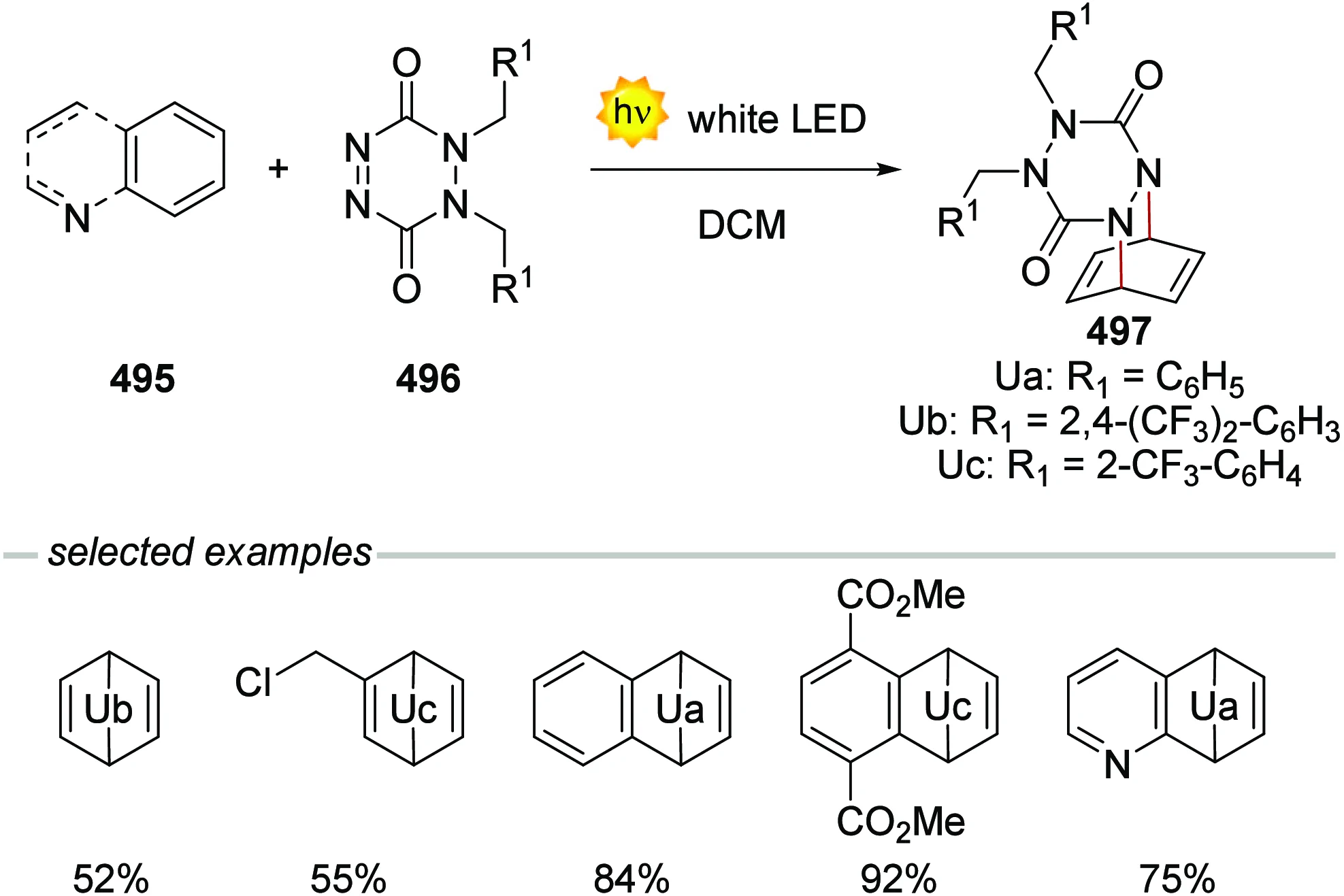
Visible-Light Dearomatization of Arenes Using TETRAD Derivatives To Afford Stable and Isolable Cycloadducts

In 2024, Doyle and co-workers developed a visible-light-induced [4 + 2] cycloaddition that converts readily available vinyldiazo compounds **498** into reactive cyclopropene intermediates **501** without the need for transition-metal catalysis (Scheme 98).^402^ Unlike thermally generated cyclopropenes, these transient species are produced under mild conditions and are intercepted before undergoing the competing Alder–ene dimerization.

**Scheme 98:**
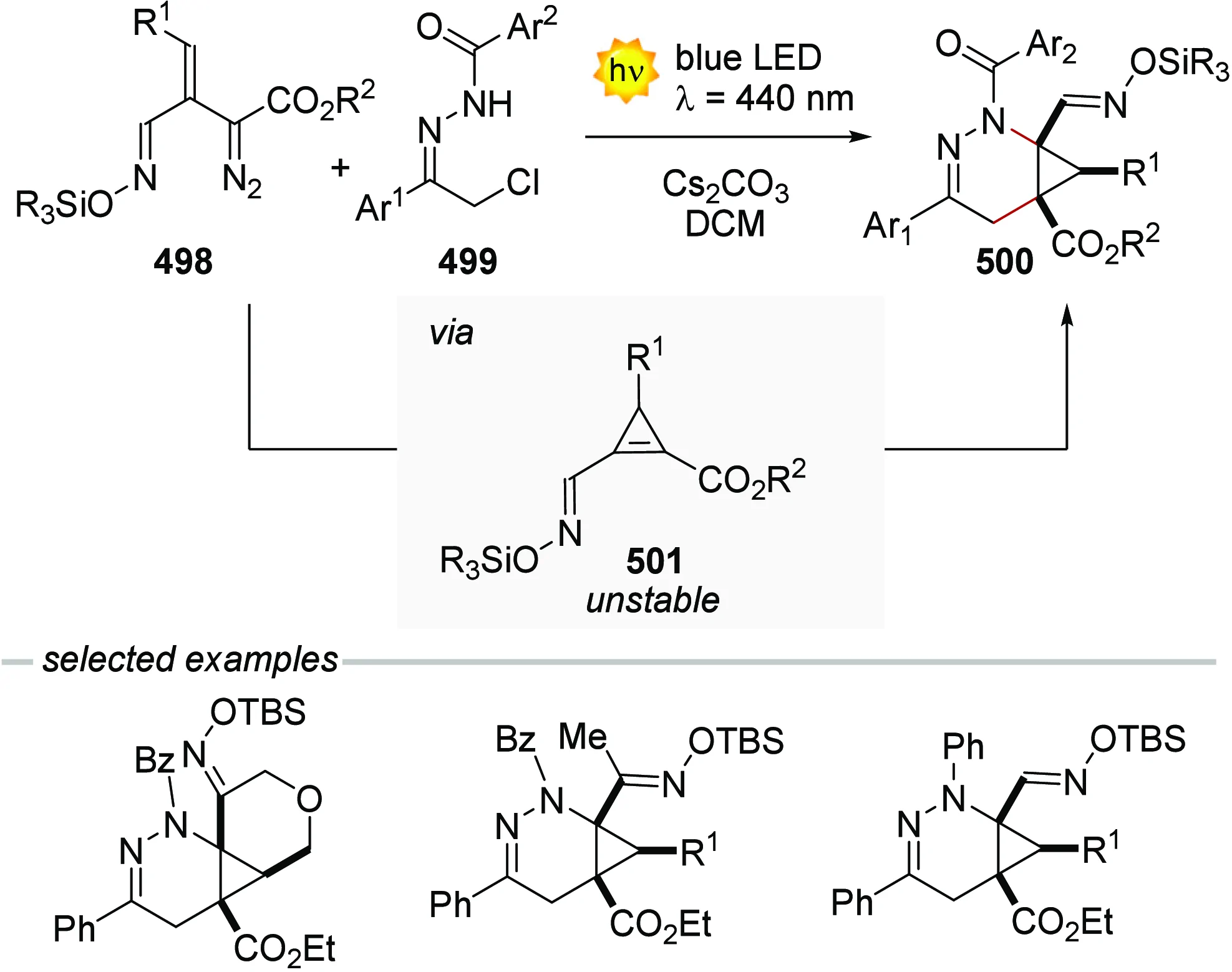
Photogeneration of Cyclopropene Derivatives and Their Reactivity in Hetero-[4 + 2] Cycloadditions

The methodology enables the efficient synthesis of bicyclo[4.1.0]­tetrahydropyridazines through [4 + 2] cycloaddition with in situ generated azoalkenes, obtaining high diastereoselectivity (d.r. > 20:1).

#### 5.2.2 Photocatalytic Methods

In recent years energy-transfer catalysis (EnT) has emerged as a privileged tool for achieving dearomative cycloaddition reactions. This approach provides direct access to complex, three-dimensional, *sp* ^3^-rich molecular frameworks from simple and readily available aromatic compounds, thereby enabling a rapid increase in molecular complexity from otherwise unreactive starting materials. In this context, Glorius et al. reported one of the first examples of catalytic dearomative [4 + 2] cycloaddition of pyridines under visible-light-mediated triplet–triplet sensitization, providing rapid and efficient access to highly functionalized isoquinuclidine (2-azabicyclo[2.2.2]­octane) analogues (Scheme 99).^403^

**Scheme 99:**
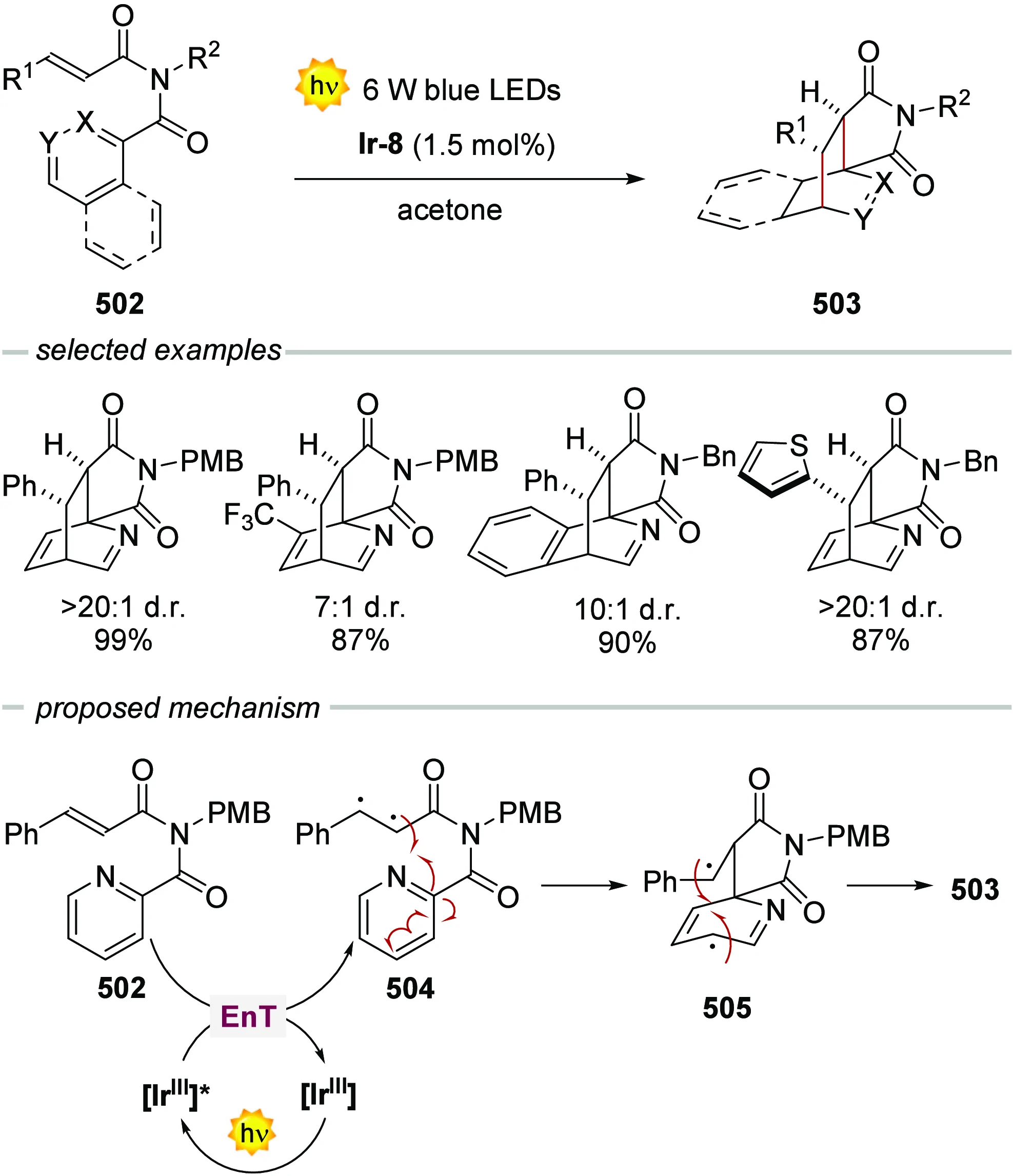
Photocatalytic Dearomatization of Pyridine via Intramolecular [4 + 2] Cycloadditions (PMB: *para*-Methoxybenzyl)

Pyridines are inaccessible to direct EnT activation due to their high triplet energy (E_T_ > 70 kcal/mol), however, in the presence of suitable and highly reactive 1,2-biradical species they can participate in [4 + 2] cycloadditions. Indeed, cinnamyl-tethered scaffolds **502** (E_T_ 48–62 kcal/mol) can be activated via EnT from an Ir-based photosensitizer to engage the pyridine ring as the 4π component in an intramolecular cycloaddition process. The scope of the method is very broad and the polymer-immobilized Ir photocatalyst (**Ir-8**) could be reused at least ten times without noticeable yield erosion. Finally, Stern–Volmer quenching experiments confirmed EnT process from the excited photocatalyst to the alkene moiety, therefore corroborating the original working hypothesis.

### 5.3 Azaxylylenes

#### 5.3.1 Direct Irradiation Methods

Aza-*ortho*-xylylene, also known as aza-*ortho*-quinone methide (aza-*o*-QM), consists of a cyclohexadiene core conjugated to an exocyclic imine and an *ortho* methylene unit, which can engage in diverse reactivity. These species can be generated using various techniques, including thermal extrusion strategy, photochemical generation, base-induced elimination, and acid-promoted elimination.^404,405^ Historically, the photochemical generation of azaxylylenes relied on the extrusion of small molecules, such as CO or N_2_, from suitable precursors under UV light irradiation. Indeed, the seminal studies of Fischer showed that oxindole derivatives could afford azaxylylene intermediate via decarboxylation reaction mediated by UV light (Scheme 100a). Similarly, Smith showed that the UV irradiation of sultams delivers the same type of intermediate (Scheme 100b,c).^406^

**Scheme 100:**
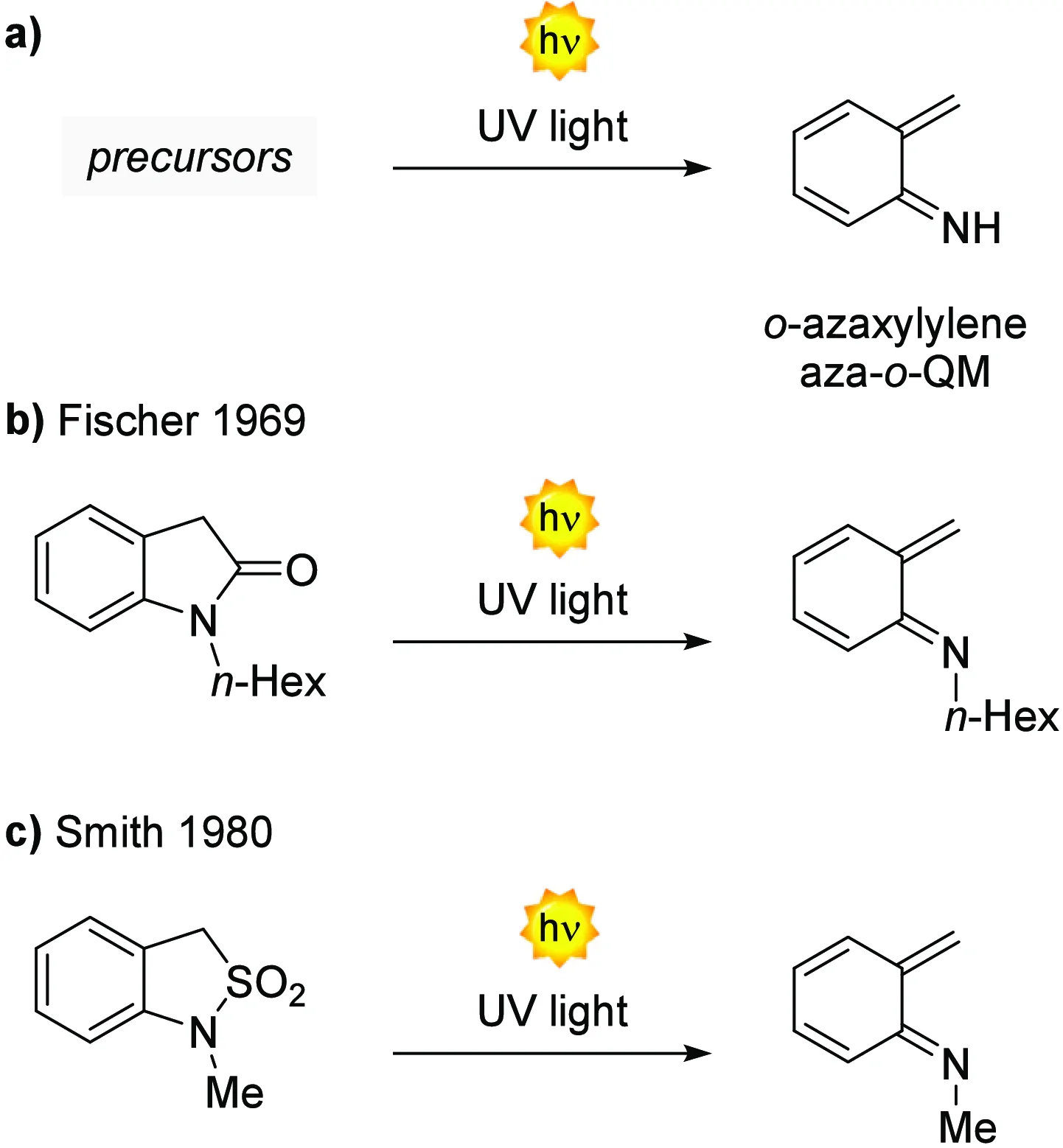
Photogeneration *o*-Azaxylylenes and Related Seminal Examples

More recently, Kutateladze and co-workers described a photochemical approach to the *in situ* generation of *o*-azaxylylenes from *o*-amidoketone and *o*-amidobenzaldehyde precursors through excited-state intramolecular proton transfer (ESIPT), demonstrating for the first time that these transient intermediates can be productively intercepted in intramolecular [4 + 2] and [4 + 4] cycloaddition reactions.^407^ One of the main challenges to efficiently exploit *o*-azaxylylenes is the fast back proton transfer that prevents their possibility to participate in bimolecular processes. Accordingly, the authors of this work carefully designed a series of substrates with pendant heterocyclic moieties to engage in intramolecular cycloadditions for the production of polyheterocycles. Depending on the nature of the pendant group and reaction conditions, the azaxylylene acts as the 4π component in inverse-electron-demand [4 + 2] or [4 + 4] cycloadditions, delivering conformationally constrained fused *N,O,S*-polyheterocycles in moderate to good yields with generally high diastereoselectivity relative to the −OH epimer of each product (not shown, Scheme 101a). The synthesis of the precursors is modular and peptoid-synthesis-inspired, relying on simple high-yielding amide bond formations, making the overall sequence amenable to diversity-oriented and combinatorial approaches (Scheme 101b). This possibility was further demonstrated in other studies by the same group in which the photochemical step was either derived from enantiomerically pure precursors,^408,409^ or combined with Suzuki-Miyaura couplings,^410,411^ epoxide formation via N_2_ extrusion,^412^ or other types of postmodifications.^413,414^ When l-proline or l-thiaproline was incorporated into the tether, complete diastereoselectivity was achieved in the [4 + 4] cycloaddition, delivering the corresponding product, bearing up to four stereocenters including a quaternary carbon, enantiomerically pure (Scheme 101c).

**Scheme 101:**
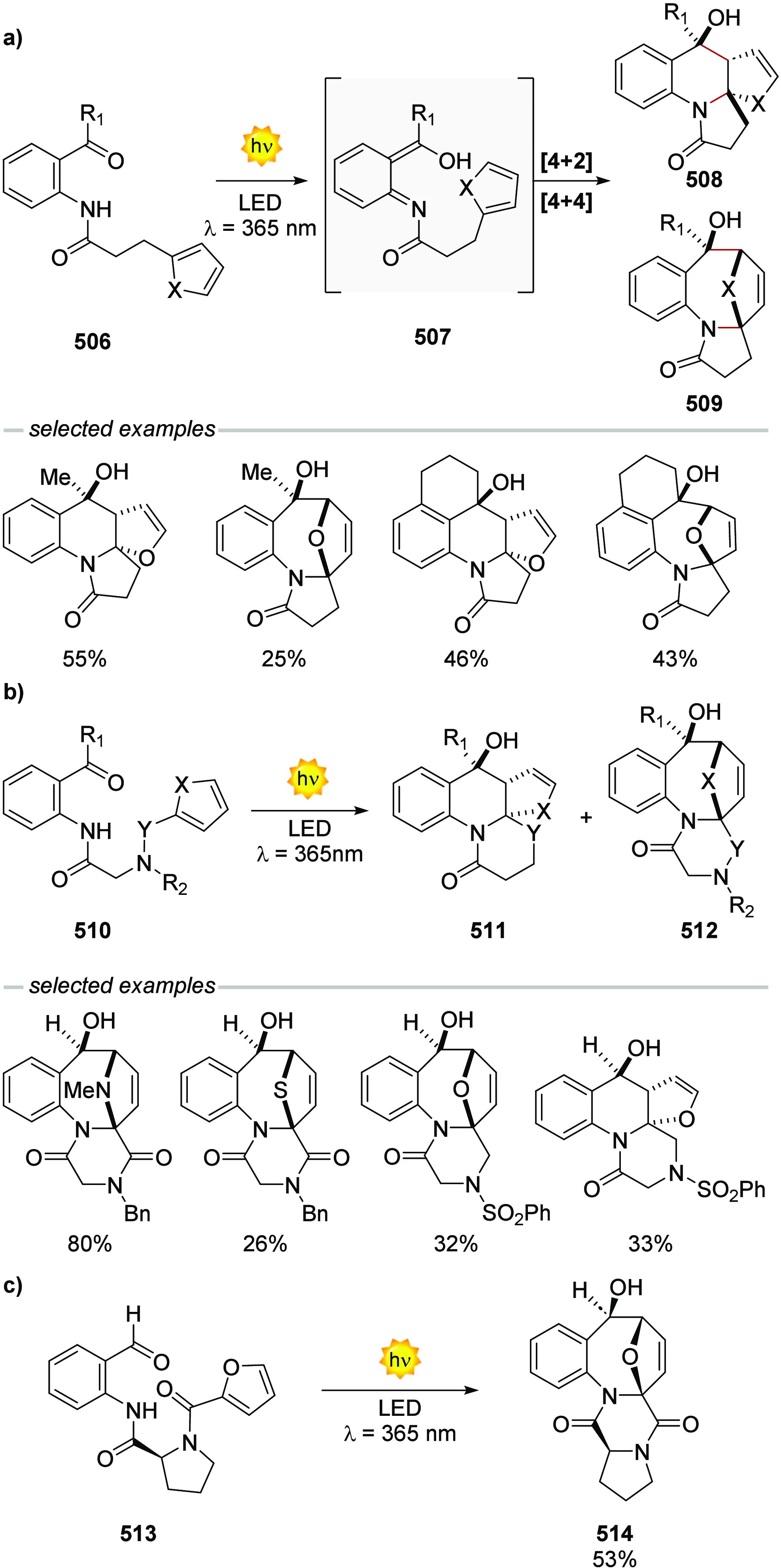
Photogeneration of Azaxylylenes and Their Intramolecular Photocycloadditions

Afterward, the same group showed that similar reactive intermediates could be obtained from imine precursors instead of carbonyl groups (Scheme 102).^415^ In this report, the authors addressed one of the main challenges related to the photochemistry of imines: the rapid dissipation of the excitation energy through nonproductive *E*/*Z* geometrical isomerization of the C=N bond. The authors reasoned that, since ESIPT is known to occur rapidly in *ortho*-substituted hydrogen-bond donor–acceptor systems including imines, it might occur on a time scale competitive with rotational relaxation.

**Scheme 102:**
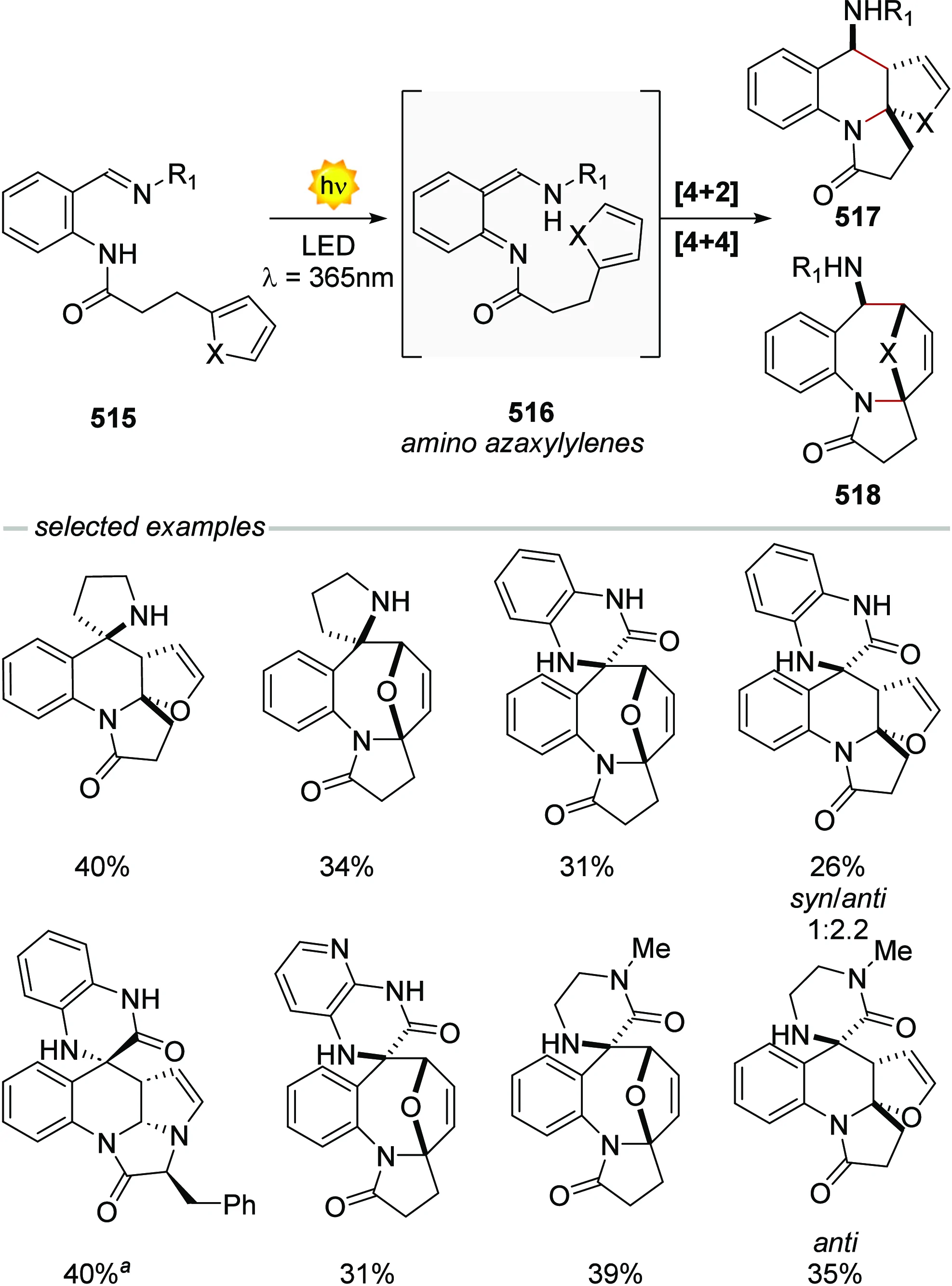
Photogeneration of Amino Azaxylylenes and Their Reactivity To Afford Spirocyclic Heterocycles^a^

a 1.5 equiv of Ti­(OiPr)_4_.

This hypothesis was validated using a broad spectrum of competent substrates that reacted smoothly under UV light irradiation to afford the corresponding [4 + 2] or [4 + 4] cycloaddition products via amino azaxylylene intermediates (Scheme 102). A key aspect of this methodology was the use of cyclic imines, which lack a rotatable C=N bond and thus cannot dissipate excitation energy through isomerization. This geometrical constraint not only improved the photoreactivity of the desired substrate but they also provided a straightforward entry to *sp* ^3^-rich spiro-heterocycles.

In 2016, Kutateladze and co-workers developed a visible-light-induced strategy for the dearomatization of benzenoid arenes based on the photochemical generation of aza-*o*-xylylene intermediates through ESIPT. These transient intermediates undergo intramolecular [4 + 2] cycloadditions with tethered benzene derivatives, providing rapid access to complex fused polycyclic architectures (Scheme 103).^416^ Interestingly, in this study is presented a peculiar reactivity of the arene moiety as a 2π dienophile in [4 + 2] cycloadditions, rather than the more common (3 + 2) (*meta*-cycloaddition) or [4 + 2] (*para*-cycloaddition). A broad range of readily accessible photoprecursors containing benzaldehyde-, acetophenone-, and tetralone-derived chromophores were investigated. The reaction proceeded with good regio- and diastereoselectivity, affording fused polycyclic heterocycles in moderate to good yields. From a synthetic standpoint, the photoproducts possess a highly versatile cyclohexadiene core amenable of extensive molecular diversification, such as, depending on substrate substitution and reaction conditions, spontaneous or acid-promoted hydrolysis to cyclohexenones or intramolecular cyclization to furnish fused cyclic ketals. Interestingly, the diene motif underwent hetero-Diels–Alder reactions with *in situ* generated benzoylnitroso species, enabling rapid construction of additional fused heterocyclic frameworks. Interestingly, the *syn*/*anti* diastereoselectivity (defined by the relative orientation of the hydroxyl group and the cyclohexadiene core) strongly depends on the nature of the carbonyl precursor. Aldehyde-derived substrates selectively afford the *syn*-isomer, methyl ketones preferentially yield the *anti*-diastereomer, whereas tetralone-derived substrates exclusively produce the anti-isomer. This trend is likely to be attributed to the progressively reduced conformational flexibility of the carbonyl group, which governs the facial selectivity of the final cyclization step.

**Scheme 103:**
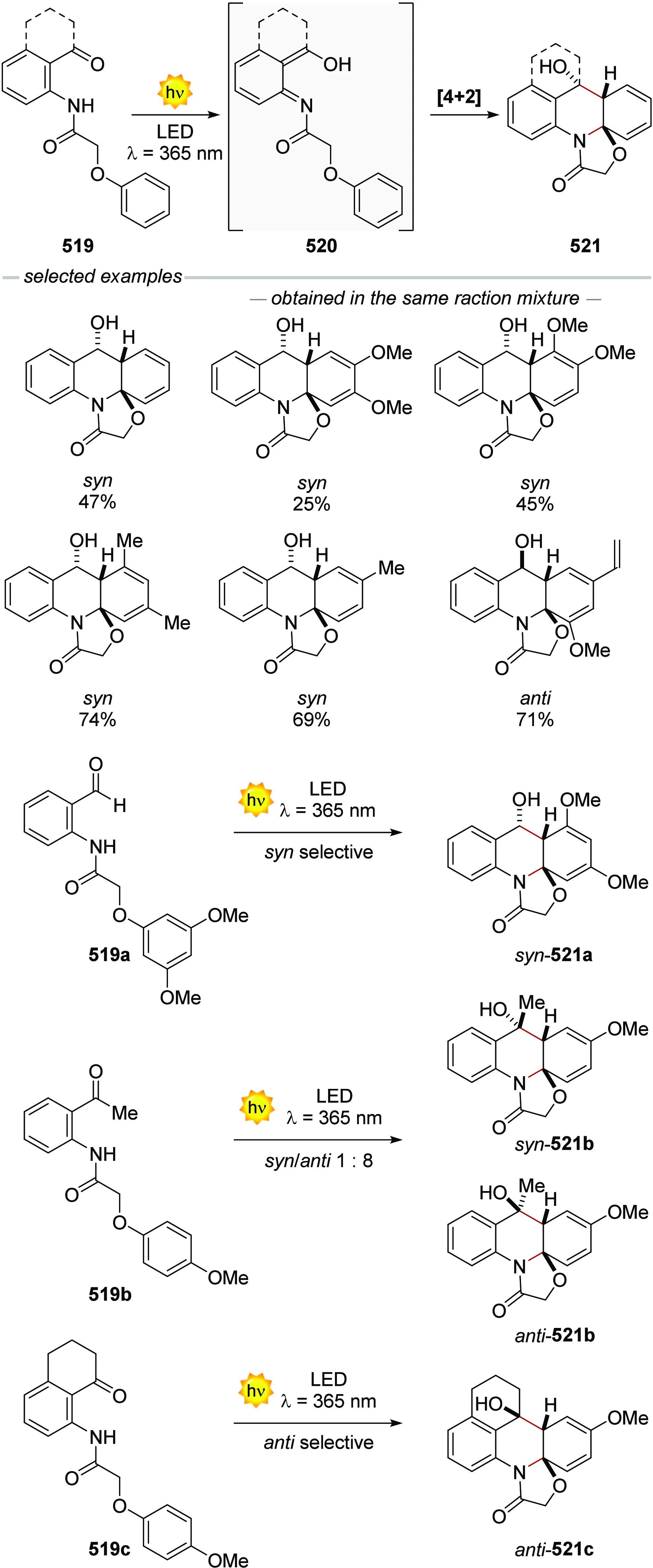
Photochemical Dearomatization of Arenes Mediated by in Situ Generated Aza-*o*-xylylenes

### 5.4 Povarov Cycloadditions

#### 5.4.1 Photocatalytic Methods

The Povarov cycloaddition, discovered in the early 1960s by Povarov and co-workers,^417,418^ is an aza-Diels–Alder cycloaddition between *N*-aryl imines and electron-rich olefins, representing one of the most straightforward synthetic entries to tetrahydroquinolines (THQs and to quinolines after oxidation), key structural elements in several natural products and/or medicinally relevant agents.^419,420^ This type of cycloaddition is an inverse-electron-demanding hetero-Diels–Alder reaction typically governed by LUMO-lowering effects promoted by either Lewis or Brønsted acids that can therefore catalyze the reaction. Traditionally, catalytic examples of the Povarov cycloaddition proceeds through a polar pathway depicted in Scheme 104a, in which the *N*-aryl imine is often generated in situ and activated by an acid additive to deliver iminium **522**. This intermediate can undergo nucleophilic attack by the electron-rich alkene in a Mannich-type reaction affording intermediate **523**. Then, an intramolecular electrophilic aromatic substitution of intermediate **523** (Friedel–Crafts alkylation) to provide intermediate **524**, followed by rearomatization through a [1,3]-hydrogen shift, affords the desired tetrahydroquinoline derivative. Alternatively, product formation through a concerted asynchronous transition state has also been proposed in some cases.^421^

**Scheme 104:**
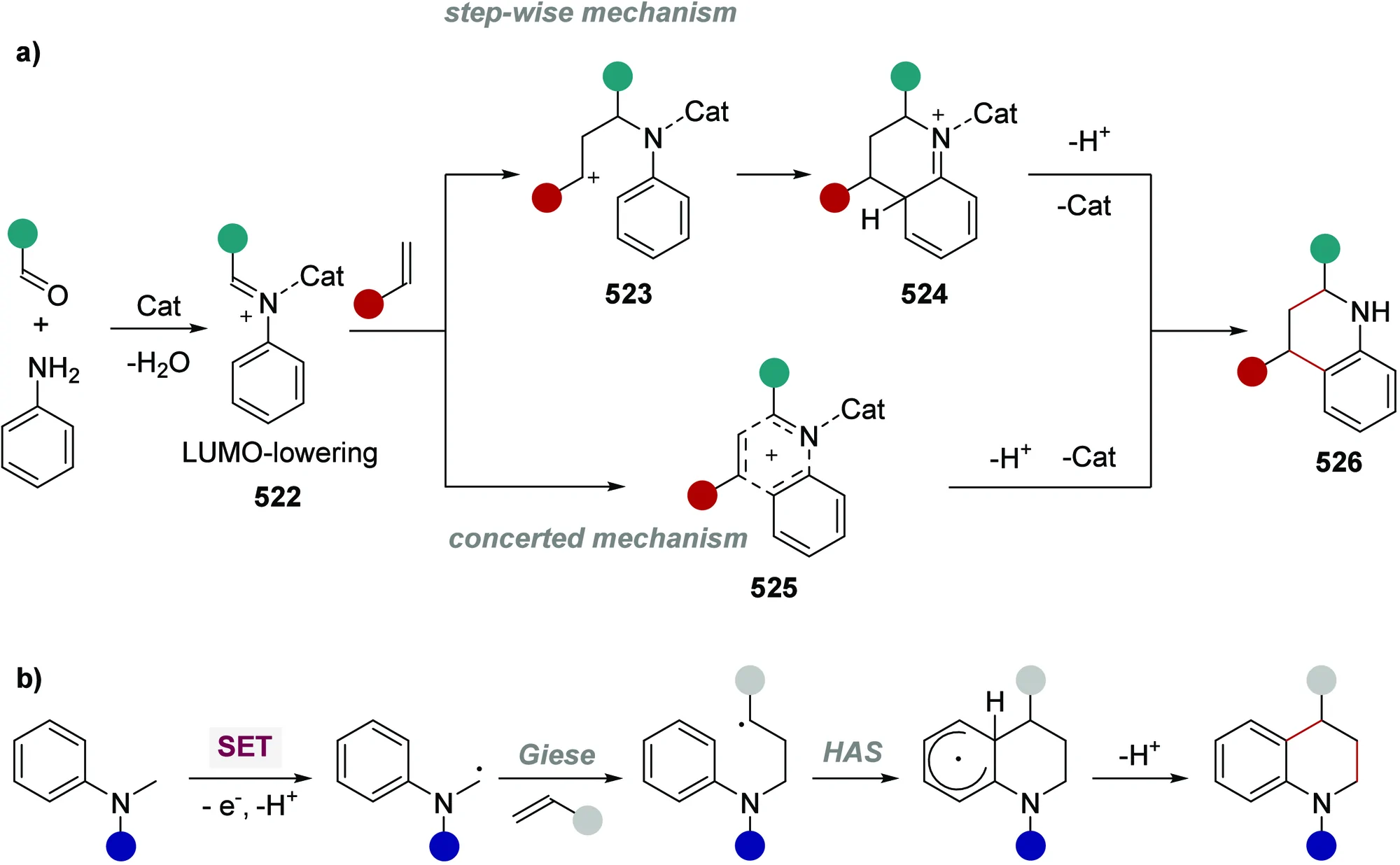
a) General Reaction Pathway for Polar Povarov Cycloadditions; b) Radical-Mediated Pathway

Iminium intermediates analogous to those invoked in the Povarov cycloaddition can be generated through single-electron oxidation of secondary *N*-alkylanilines or tertiary *N*-alkyl-*N*-methylanilines, with concomitant dehydrogenation, affording reactive species capable of engaging electron-poor alkenes as dienophiles. The advent of visible-light photoredox catalysis has provided an operationally mild and versatile platform for accessing such open-shell intermediates through SET with a suitable photocatalyst, thereby opening alternative reaction manifolds that circumvent the harsh conditions traditionally required. Within this context, photocatalytic Povarov-type cycloadditions, and visible-light-promoted cycloadditions more broadly, have attracted considerable interest, owing to their capacity to deliver densely functionalized tetrahydroquinoline scaffolds with high efficiency and stereochemical control. On the other hand, photocatalytic and/or photochemical Povarov cycloadditions are generally initiated by the formation of alfa-amino radicals via oxidation of the corresponding aniline precursors (Scheme 104b). Then the overall process can be rationalized as a tandem Giese-type addition to an alkene and subsequent homolytic aromatic substitution (HAS) with the aromatic moiety. Finally, the rearomatization of the aryl group affords the desired THQ (or quinoline) derivative.

The breadth of this field is reflected in the substantial amount of literature that has been reported over the past two decades, the scope and significance of which have been beautifully described in two comprehensive reviews published in 2024 and 2025.^422,423^

For this reason, the focus of this chapter will be to describe the most relevant strategies to generate the reactive intermediates that can participate in Povarov cycloadditions, thus providing useful guidelines on the general activation modes for reaction development. In addition to this, the few examples that have been reported after 2025 will be reviewed.

As shown in Table 2, a survey of the literature showed that several photocatalytic strategies have been employed to generate one of the activated species involved in the Povarov cycloaddition. One representative example for each activation mode is presented in each row of Table 2, illustrating a characteristic case for its respective category. Generally, they rely on the oxidation of *N*-dialkylanilines to the corresponding α-amino radical that is then trapped with a suitable dienophile (Table 2, entry 1). Alternatively, the same species can also be generated either via direct HAT process (Table 2, entry 2) or by EDA-complex photoexcitation (Table 2, entry 3). Similarly, *N*-aryl imines can be activated by PCET to afford alfa-amino radicals (Table 2, entry 4). Furthermore, photocatalytic dehydrogenation (Table 2, entry 5) or decarboxylation (Table 2, entry 6) of *N*-aryl glycine esters or *N*-aryl glycine derivatives respectively allow for in situ imine formation. Finally, activation of the dienophile by EnT (Table 2, entry 7) catalysis or SET-oxidation (Table 2, entry 8) have also been reported to develop alternative Povarov cycloadditions.

**Table 2:**
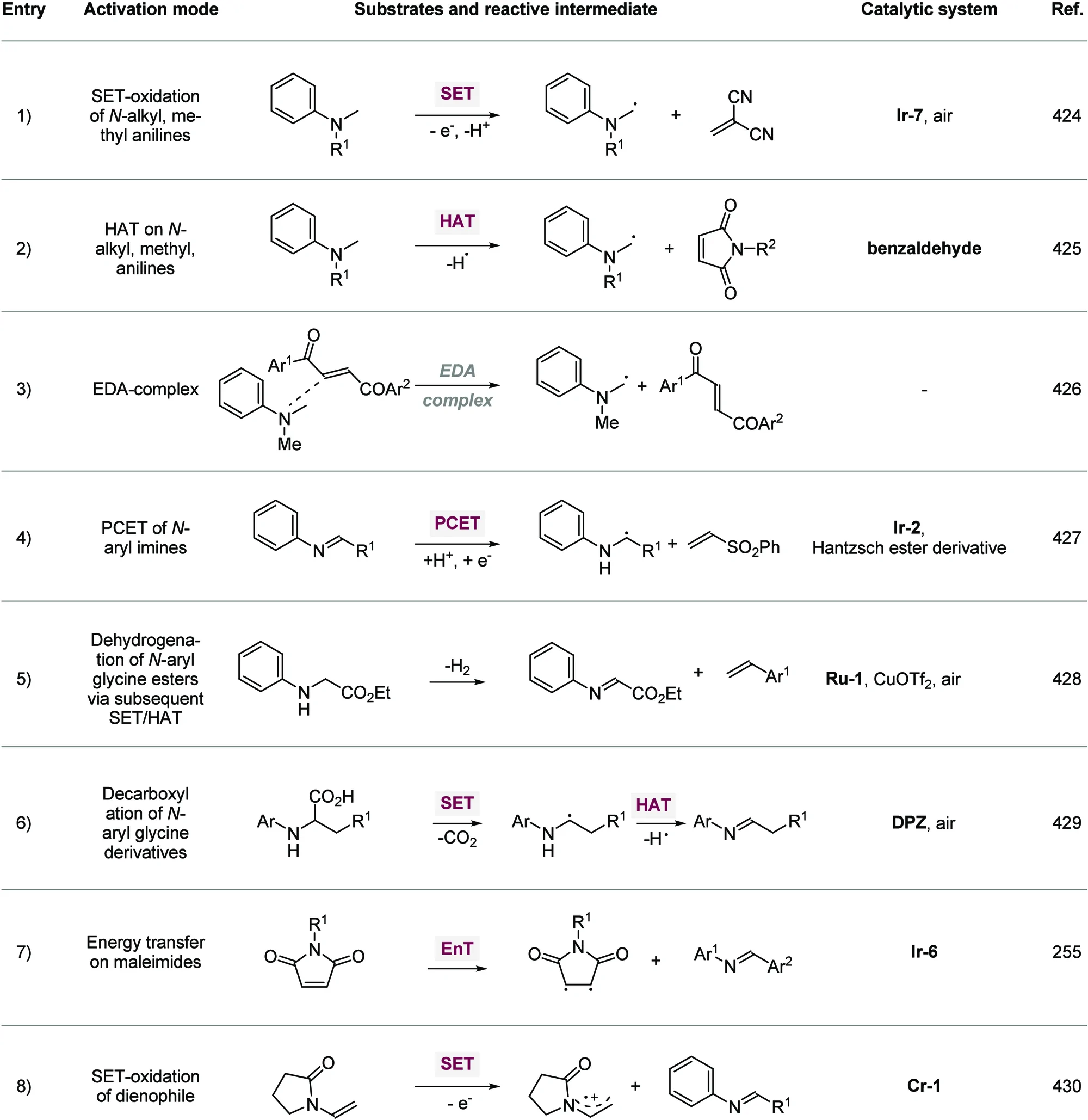
Representative Examples of Activation Modes and Corresponding Reactive Intermediates Involved in Photocatalytic Povarov Cycloadditions^255,424–430^

In 2025, Kim, Ryu et al. reported a catalytic enantio- and diastereoselective Povarov cycloaddition reaction for the synthesis of tetrahydroquinolines, employing a chiral oxazaborolidinium ion (**COBI**) Lewis acid catalyst in combination with an Ir-based photosensitizer (**Ir-9**) under visible-light photoredox conditions (Scheme 105).^431^ In the proposed reaction pathway, the observed reactivity is initiated by single-electron oxidation of the α-silyl amine substrate by the photoexcited Ir­(III) sensitizer (*E*_1/2_ = +0.66 V vs. SCE), which is thermodynamically feasible given the oxidation potential of the substrate (*E*_1/2_ = +0.60 V vs. SCE), as confirmed by Stern–Volmer luminescence quenching studies. Desilylation of the resulting radical cation delivers the reactive α-aminoalkyl radical, whose enantioselective Giese addition to the **COBI**-activated α,β-unsaturated ester **527** is directed by the bulky 3,5-di-*tert*-butylphenyl groups of the catalyst, resulting in the formation of α-carbonyl radical **531**. This species undergoes intramolecular homolytic aromatic substitution to afford the cyclohexadienyl radical intermediate **532**, which is oxidized by TMS- or proton-activated benzophenone to afford cationic intermediate **533**. The authors showed how, upon Lewis or Brønsted acid activation, the reduction potential of benzophenone can be significantly shifted from −1.83 V to approximately −0.95 V. Subsequently, rearomatization by deprotonation to deliver the THQ product **529**, with concomitant regeneration of the Ir­(III) photocatalyst, enables catalytic activity. The isolation of coproduct **537** in 57% yield is consistent with the proposed reaction mechanism.

**Scheme 105:**
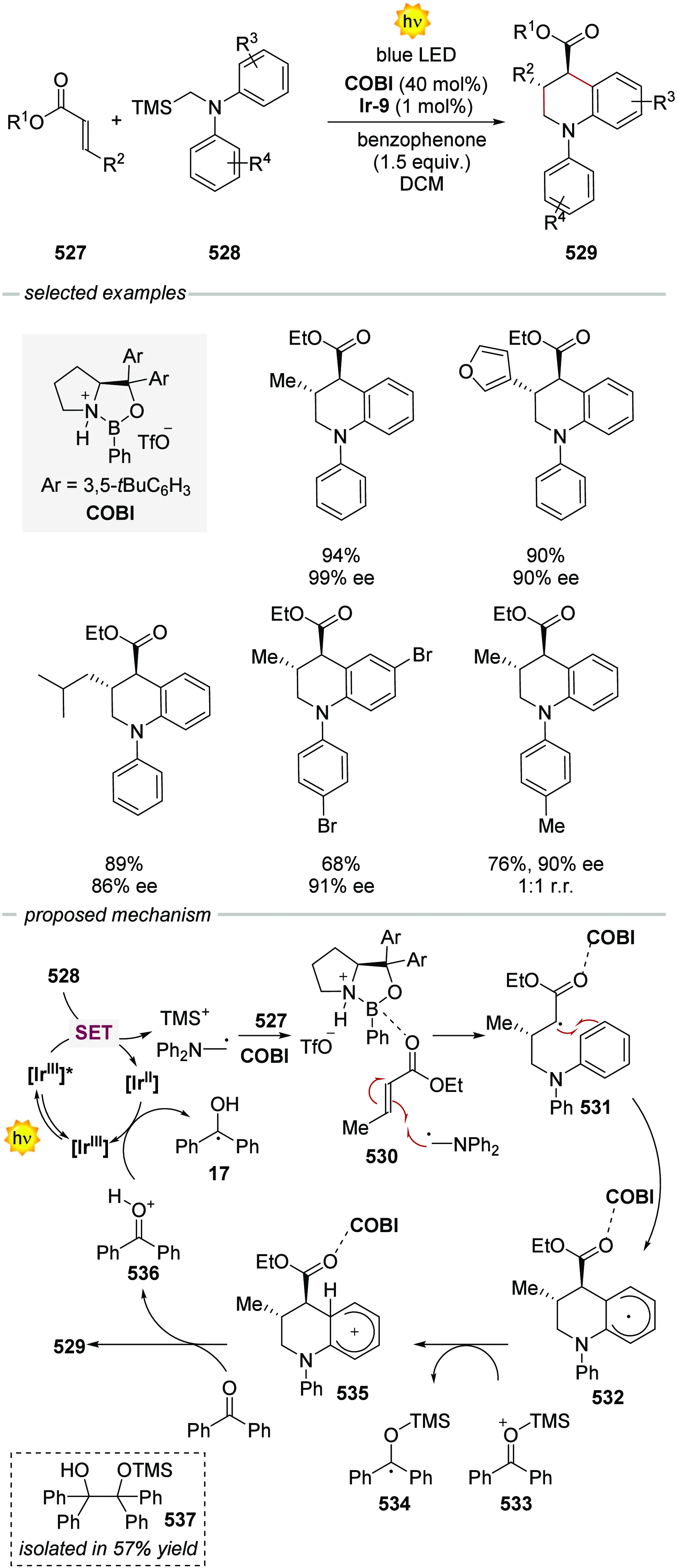
Photocatalytic Enantioselective Povarov Cycloaddition Promoted by a Chiral Oxazaborolidinium Ion Catalyst

The group of Das reported the photocatalytic aza-Diels–Alder reaction between the Danishefsky’s diene **538** and imines **430** for the synthesis of 2,3-dihydropyridin-4­(1*H*)-one derivatives **539** (Scheme 106).^432^ Compared to the previous related strategies on photocatalytic Diels–Alder reactions, the authors employed a cheaper and nontoxic organic dye that facilitate the desired reaction. The scope of the reaction is relatively broad, consistently providing high values of yield. While a series of Stern–Volmer quenching studies established that the excited state of fluorenone is quenched by the more electron-rich reaction partner, various control experiments revealed that molecular oxygen is required in the reaction to increase the overall yield.

**Scheme 106:**
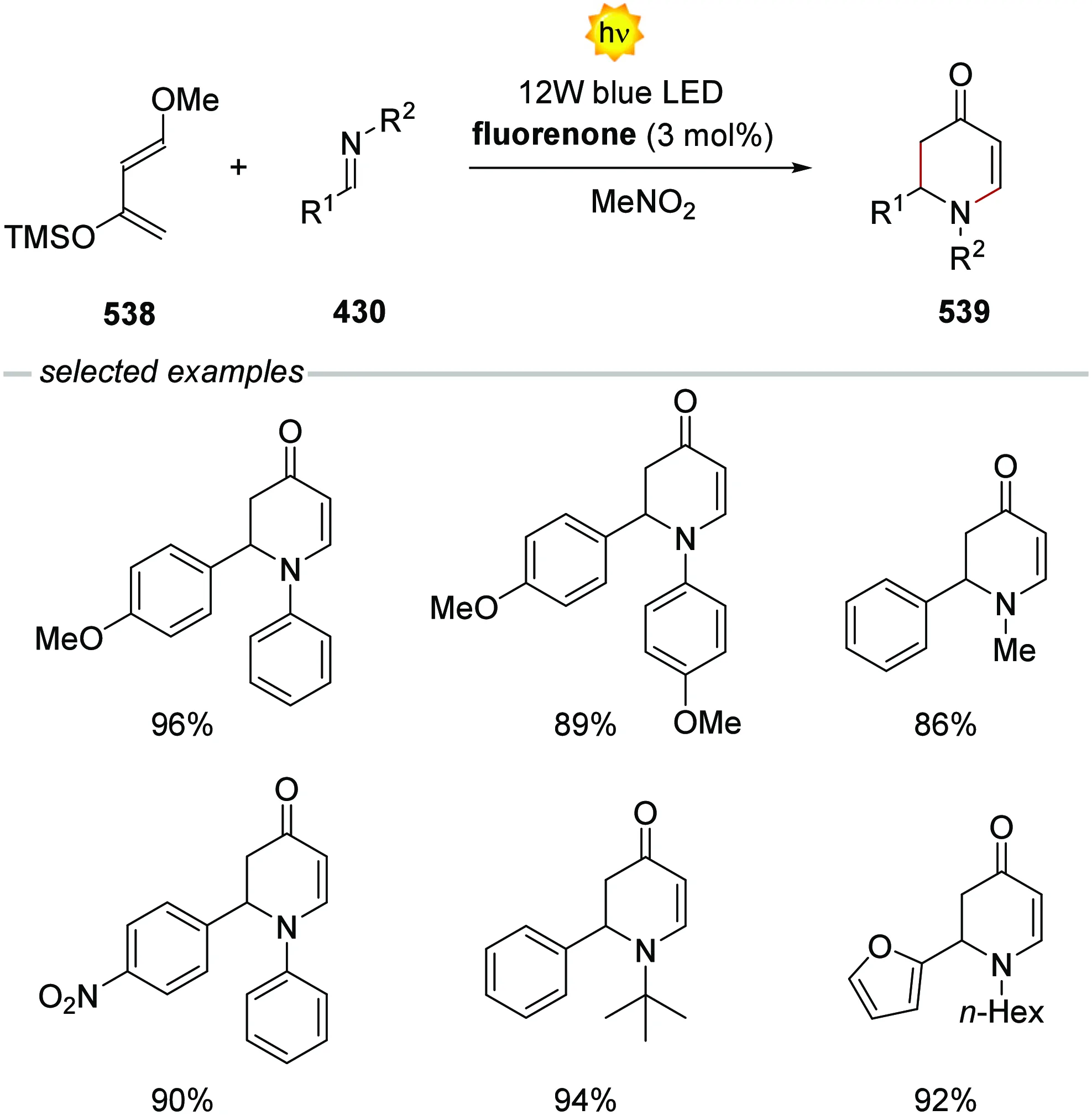
Fluorenone Mediated Aza-Diels–Alder Cycloaddition

### 5.5 *N,O*-Heterocycles

#### 5.5.1 Photocatalytic Methods

Nitrosobenzenes are highly reactive heterodienophiles that readily participate in hetero-Diels–Alder reactions. However, their widespread synthetic application is often limited by their intrinsic instability, as they readily undergo dimerization or overoxidation. To overcome these limitations, the development of photochemical strategies has enabled the in situ generation of nitrosobenzenes, allowing their employment in cycloaddition reactions. Indeed, the photochemical decomposition of 1,2,4,-oxadiazole-4-oxide derivatives affords the corresponding nitrosobenzene species that is then intercepted by cyclohexadiene affording the hetero-Diels–Alder product.^433^

More recently, in 2017, Masson et al. developed a visible-light-mediated tandem oxidation/cycloaddition strategy for the synthesis of 3,6-dihydro-1,2-oxazines **542**. The method generates *in situ* nitrosoarene compounds, unstable intermediates, directly from arylhydroxylamines **541** under photocatalytic conditions that undergo hetero-Diels–Alder reactions in the presence of electron rich dienes **540** (Scheme 107).^434^ The combination of **Ru-1**, blue LED irradiation and an oxygen atmosphere, provided a more sustainable oxidative protocol. Interestingly, the use of trifluoroethanol (TFE) and 2,6-lutidine were crucial for suppressing overoxidation of the nitroso intermediate and promoting efficient cycloaddition in the presence of various dienes. The authors carried out a series of mechanistic experiments to propose the reaction pathway shown in Scheme 15. Photoexcitation of Ru­(II) and subsequent oxidation of the phenylhydroxylamine **2** affords hydroxylammonium radical cation **543**. The reduced photocatalyst is subsequently reoxidized by molecular oxygen, regenerating the catalytic cycle while producing a superoxide radical anion. Deprotonation of species **543**, followed by a second oxidation mediated by superoxide, furnishes the reactive nitrosoarene **545**, which is rapidly trapped by the diene through a nitroso-Diels–Alder cycloaddition to afford the desired product **542**. The authors also acknowledge an alternative pathway involving singlet oxygen, generated by energy transfer from the excited photocatalyst to molecular oxygen, as a possible oxidant for hydroxylamine. Nevertheless, based on precedent and experimental observations, they propose that the photoredox SET mechanism is the dominant pathway.

**Scheme 107:**
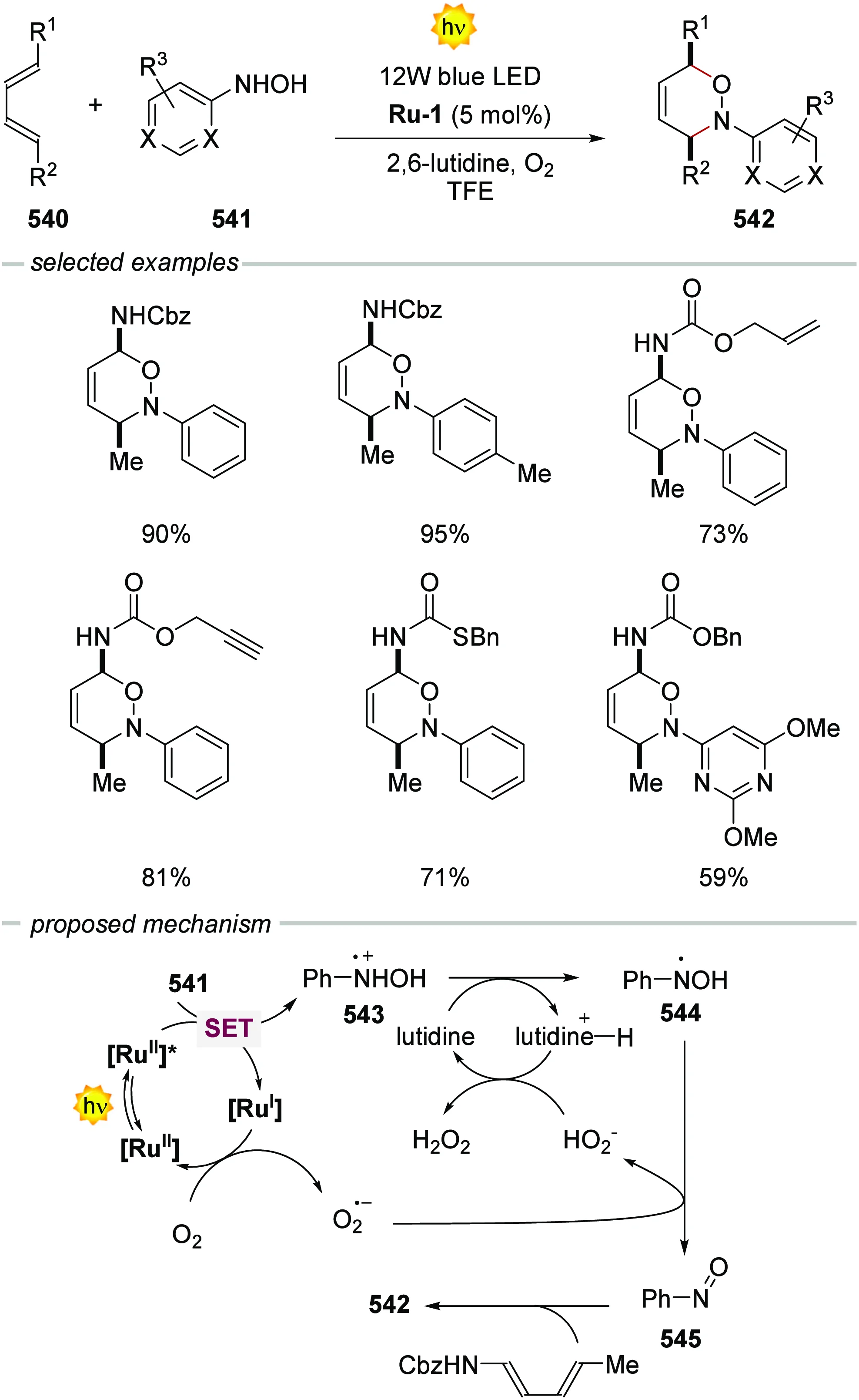
Visible-Light Promoted Nitroso Diels–Alder Reaction

More recently, Hou and co-workers reported a very similar methodology employing an Ir-based photocatalyst.^435^

In 2023, the group of Feng developed a visible-light-mediated [4π+2σ] cycloaddition between aryl cyclopropanes **546** and nitrones **318** using an acridinium photocatalyst **Acr-1** under mild conditions (Scheme 108).^436^ One interesting aspect of this methodology is the use of electron-rich aryl cyclopropanes, through the formation of a radical cation intermediate, in radical cycloaddition reactions, as opposed to the traditional donor–acceptor cyclopropanes typically used in polar cycloadditions. Mechanistic investigations suggest that the transformation proceeds through a stepwise radical pathway rather than a concerted cycloaddition. For example, the identical product obtained from both *cis*- and *trans*-cyclopropanes, together with complete inhibition by TEMPO, confirms the involvement of radical intermediates. Luminescence quenching and electrochemical studies indicate that the productive pathway begins with the oxidation of the aryl cyclopropane by the excited acridinium photocatalyst, generating a cyclopropane radical cation **548**. The resulting radical cation undergoes a nucleophilic attack by the nitrone oxygen, followed by ring opening of the cyclopropane to generate a benzylic radical **549**. This intermediate subsequently cyclizes to afford the cyclic species **550** that, upon reduction from the radical form of the photocatalyst, provides product **4**.

**Scheme 108:**
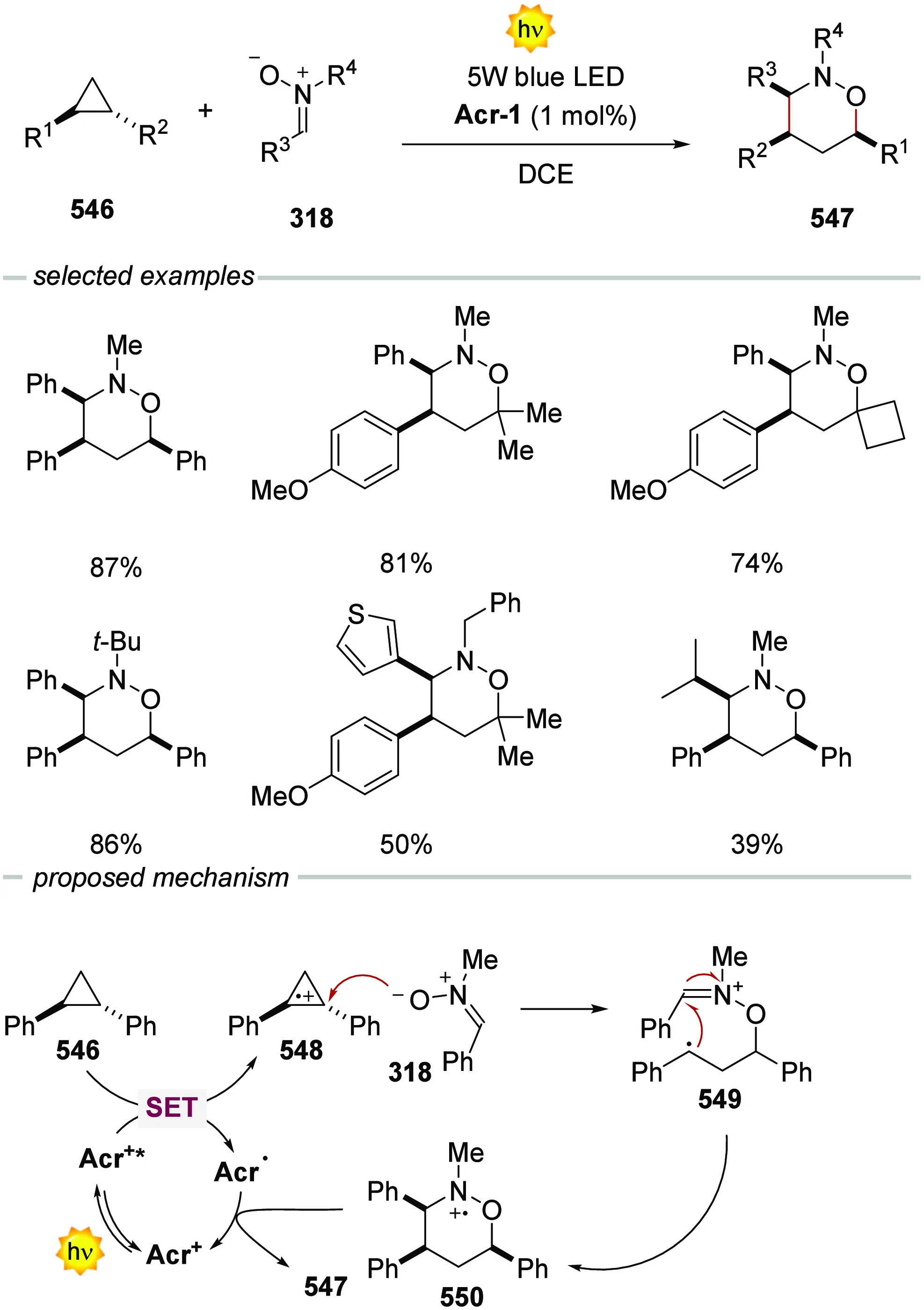
Photocatalytic Hetero-cycloaddition of Nitrones with Aryl Cyclopropanes

In 2021, Park and co-workers reported a visible-light photocatalyzed divergent cycloaddition methodology in which imino-alkyne and imino-alkene substrates undergo distinct reaction pathways under metal-free organophotocatalytic conditions. While the [2 + 2] pathway of imino-alkynes has been already described in this review (*vide supra*), the [4 + 2] cycloaddition pathway becomes predominant when imino-alkenes are employed under similar reaction conditions (Scheme 109).^173^ The scope of the [4 + 2] cycloaddition is quite broad and the reaction efficiency generally improves with electron-rich or neutral substrates, as opposed to the electron-withdrawing ones. Furthermore, the reaction tolerates heteroatom-containing tethers delivering dihydro-1,4-oxazines as single diastereomers in generally high yields. From a mechanistic perspective, the authors circumvented the classical limitations of excited-state reactivity of imines (see section 2.4) by operating under an electron-transfer instead of energy-transfer manifold. The working hypothesis was validated by correlating the oxidation potential of the substrate with the one of the photocatalyst in the excited state, rather than with its triplet energy. Stern–Volmer quenching studies showed that the styrene portion of the substrate effectively quenches the excited state of the photocatalyst. The reaction pathway is proposed to start with the reductive quenching of the excited-state acridine photocatalyst to afford radical cation **553**, which undergoes nucleophilic attack by the nitrogen atom to form intermediate **554**. Then, cyclization of the oxygen atom to afford the bicyclic scaffold **552** and single-electron-reduction to restore the cationic form of the photocatalyst provides the desired [4 + 2] cycloadduct.

**Scheme 109:**
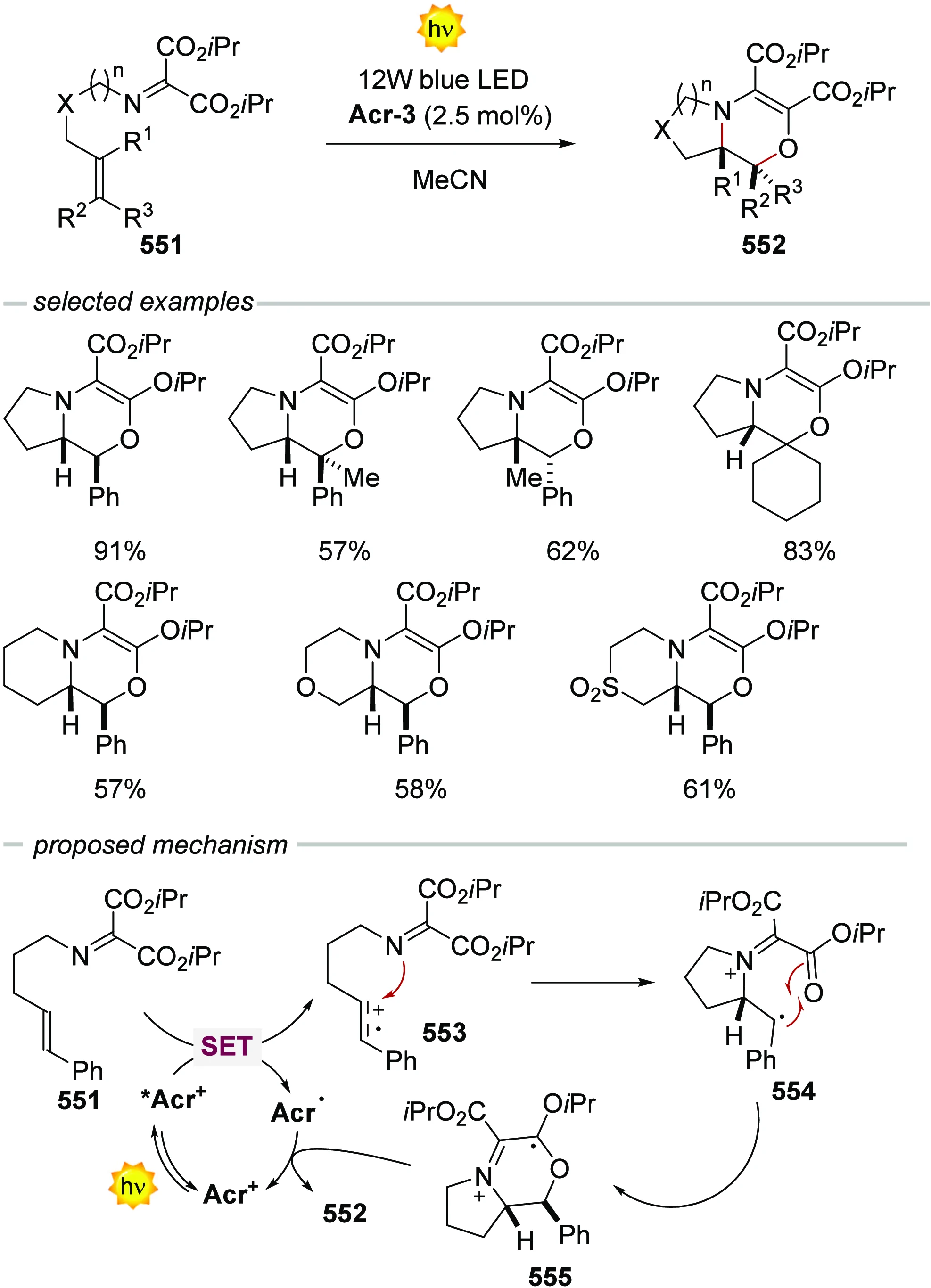
Imine-Alkyne Photocatalytic Intramolecular [4 + 2] Cycloadditions via Redox-Neutral Mechanism

### 5.6 Ketenes

#### 5.6.1 Direct Irradiation Methods

Ketenes, generated *in situ* through the visible-light-induced Wolff rearrangement of diazo compounds, have recently emerged as versatile intermediates for the development of hetero-[4 + 2] cycloaddition reactions. In recent years, this area has attracted considerable attention, culminating in several recent reviews that highlight the visible light-mediated reactivity of diazo compounds and their broad synthetic applications.^221,437,438^ In particular, these reviews provide an in-depth discussion of the tandem sequence involving photochemical ketene generation via the Wolff rearrangement followed by hetero-[4 + 2] cycloaddition, underscoring its utility as an efficient strategy for the rapid construction of heterocyclic frameworks. For this reason, this chapter will attempt to summarize the most relevant example for photochemical ketene generation and their use in hetero-cycloaddition reactions.

As summarized in Scheme 110, α-diazoketones have been predominantly employed as precursors for the visible-light-mediated generation of ketenes. Nevertheless, an alternative approach has been reported using α-ketoacylsilylanes, a subclass of acylsilanes, which furnish the corresponding siloxyketenes via a photochemically induced Brook-type rearrangement.^439^ In order to obtain *N*-containing heterocycles, these species have been reacted with a variety of reaction partners including saccharine-derived azadienes,^440^ Morita-Baylis-Hilmann carbonates,^441^ azadienes generated in situ from enynamides under gold catalysis,^442^ benzofuran-derived azadienes,^443^ and vinyl benzoxazinones.^444^ To obtain oxygenated heterocycles instead, ketenes have been used in combination with alkylidene oxindoles,^445^ or tropolone derivatives which however afforded [8 + 2] cycloaddition products.^446^

**Scheme 110:**
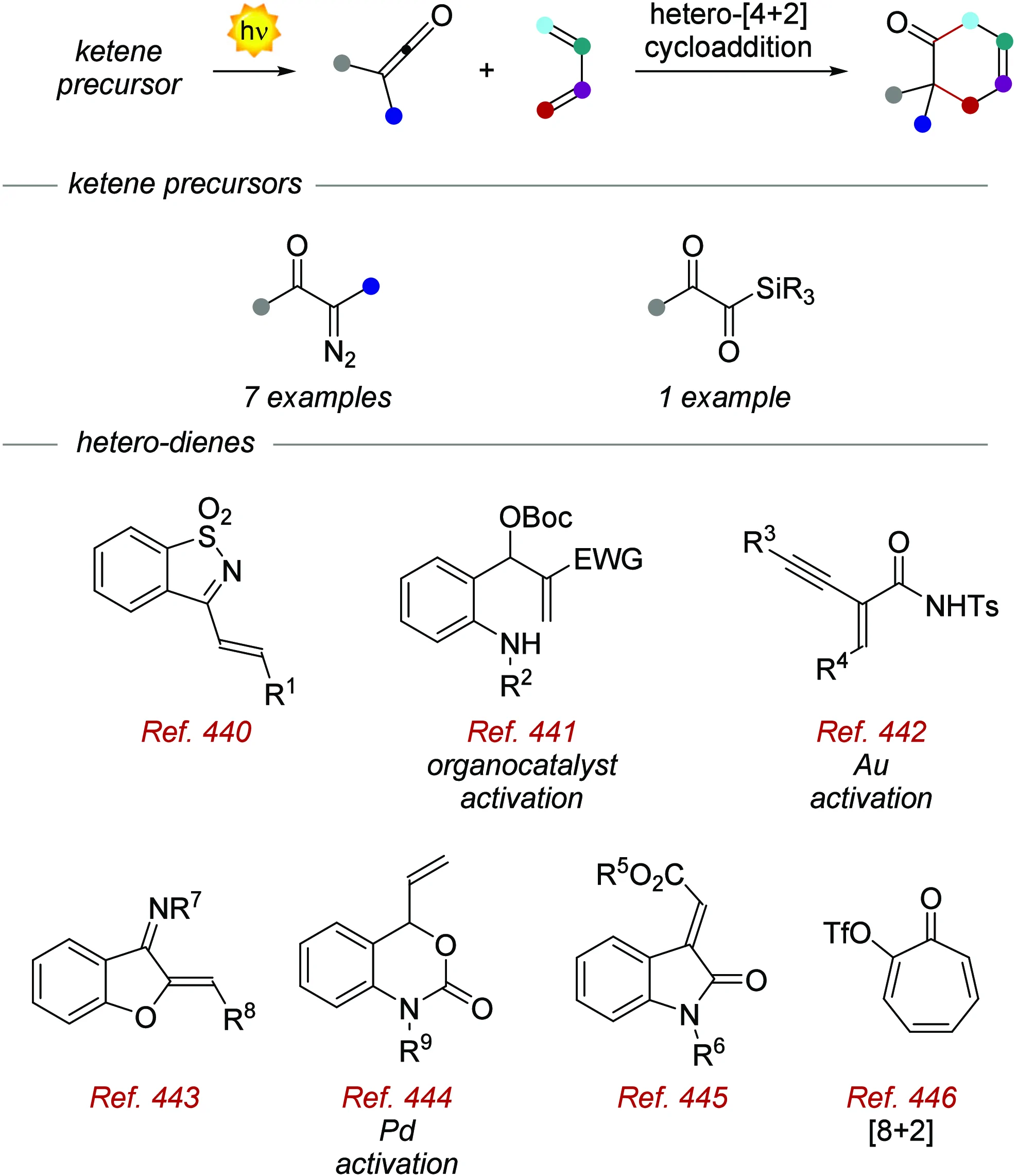
Summary of Photogenerated Ketenes Reactivity in Hetero-cycloaddition Reactions

## 6 Cheletropic Reactions

Cheletropic reactions are cycloadditions in which the terminal atoms of a fully conjugated polyene form two new σ-bonds to a single atom of a monocentric addend, resulting in the formal loss of one σ-bond from the polyene and an increase in the coordination number of the addend’s atom. These reactions also include those processes where the two sigma bonds are broken in concert from the monocentric addend (Scheme 111).

**Scheme 111:**
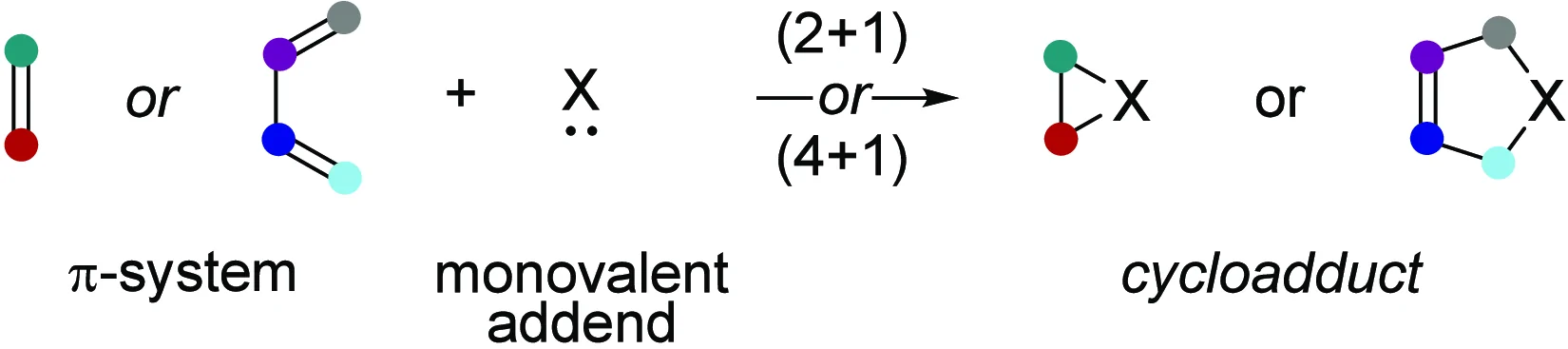
General Reaction of an Unsaturated Molecule and a Monovalent Addend

In the context of HPCs, one of the earliest examples of aziridination is the one reported by Lwowski and Mattingly who studied the photochemical decomposition of ethyl azidoformate to generate the corresponding nitrene intermediate, which was subsequently trapped in the presence of cyclohexene.^447^ Several years later, the cheletropic trapping of nitric oxides with butadiene derivatives was reported.^448^ The advent of visible light photocatalysis has led to the development of various reagents and activation strategies for the selective generation of reactive monovalent intermediates, including carbenes and nitrenes. By providing precise control over their formation and reactivity, these methodologies have significantly improved the selectivity and expanded the scope of cheletropic-type transformations.

### 6.1 Direct Irradiation Methods

In 2024, Parasram and co-workers introduced a metal- and photocatalyst-free aziridination of alkenes **52** based on the direct visible-light excitation of azoxy-triazenes **556**.^449^ Under irradiation at 390 nm, readily accessible phthalimido-substituted azoxy-triazenes serve as nitrogen-atom-transfer reagents, generating a free singlet nitrene that undergoes concerted cycloaddition to activated and unactivated alkenes to afford *N*-phthalimido aziridines **557** (Scheme 112). The method tolerates electronically diverse styrenes, cyclic and acyclic unactivated alkenes, multisubstituted olefins, terpenes, fatty-acid derivatives, and complex alkene-containing substrates, while avoiding competing allylic C–H amination. Mechanistic studies supported direct excitation of the azoxy-triazene precursor and singlet nitrene formation **558-S**_**1**_ via fragmentation of excited azoxy-triazene 556*, as indicated by nitrene trapping experiments, stereochemical probes, kinetic competition studies, and control experiments on azoxy-triazene photodecomposition. The resulting *N*-phthalimido aziridines can also be converted into free aziridines through a mild photoredox N–N bond cleavage or further derivatized through ring-opening chemistry, highlighting the synthetic utility of the protected aziridine products.

**Scheme 112:**
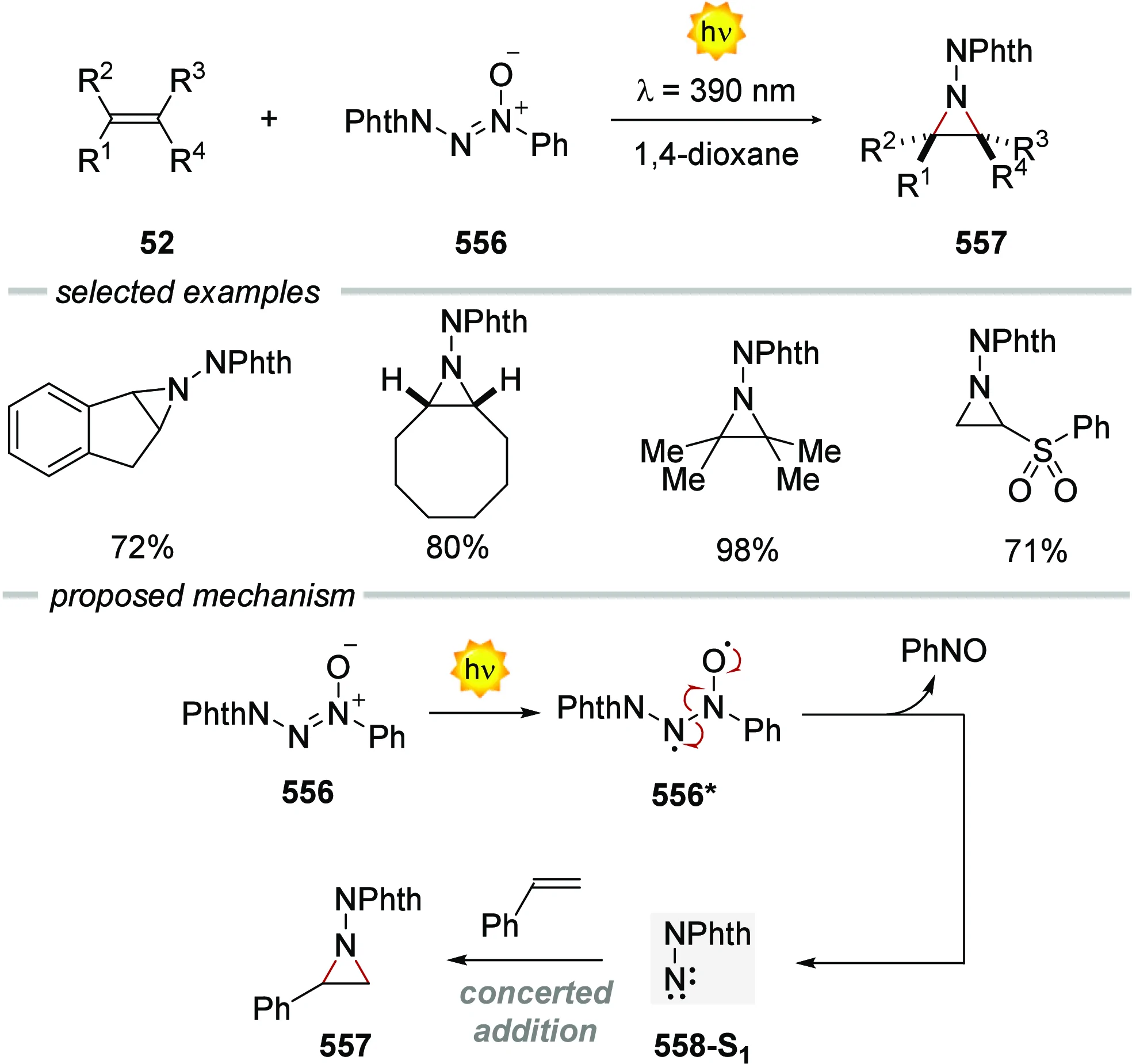
Direct Visible-Light Generation of Phthalimidonitrene from Azoxy-Triazenes for Metal- and Photocatalyst-Free Alkene Aziridination

An example of a (5 + 1) cycloaddition was reported by the group of Xu. Pyridinium 1,4-zwitterionic thiolate **559** reacted with phosphoryl diazo compound **560** under blue light irradiation to afford phosphoryl derivatives of dihydropyrido­[2,1-*c*]­[1,4]­thiazines **561** (Scheme 113).^450^ Mechanistically, direct photoexcitation of diazo compound **560** was proposed to trigger nitrogen extrusion, generating singlet carbene **562**. Subsequent nucleophilic addition of thiolate **559** affords zwitterionic intermediate **563**, which undergoes stereoselective cyclization to furnish predominantly the *trans* isomer. Notably, the corresponding thermal reaction, performed under microwave irradiation at 100 °C, afforded the product in only low yield, highlighting the crucial role of photochemical carbene generation in enabling this transformation.

**Scheme 113:**
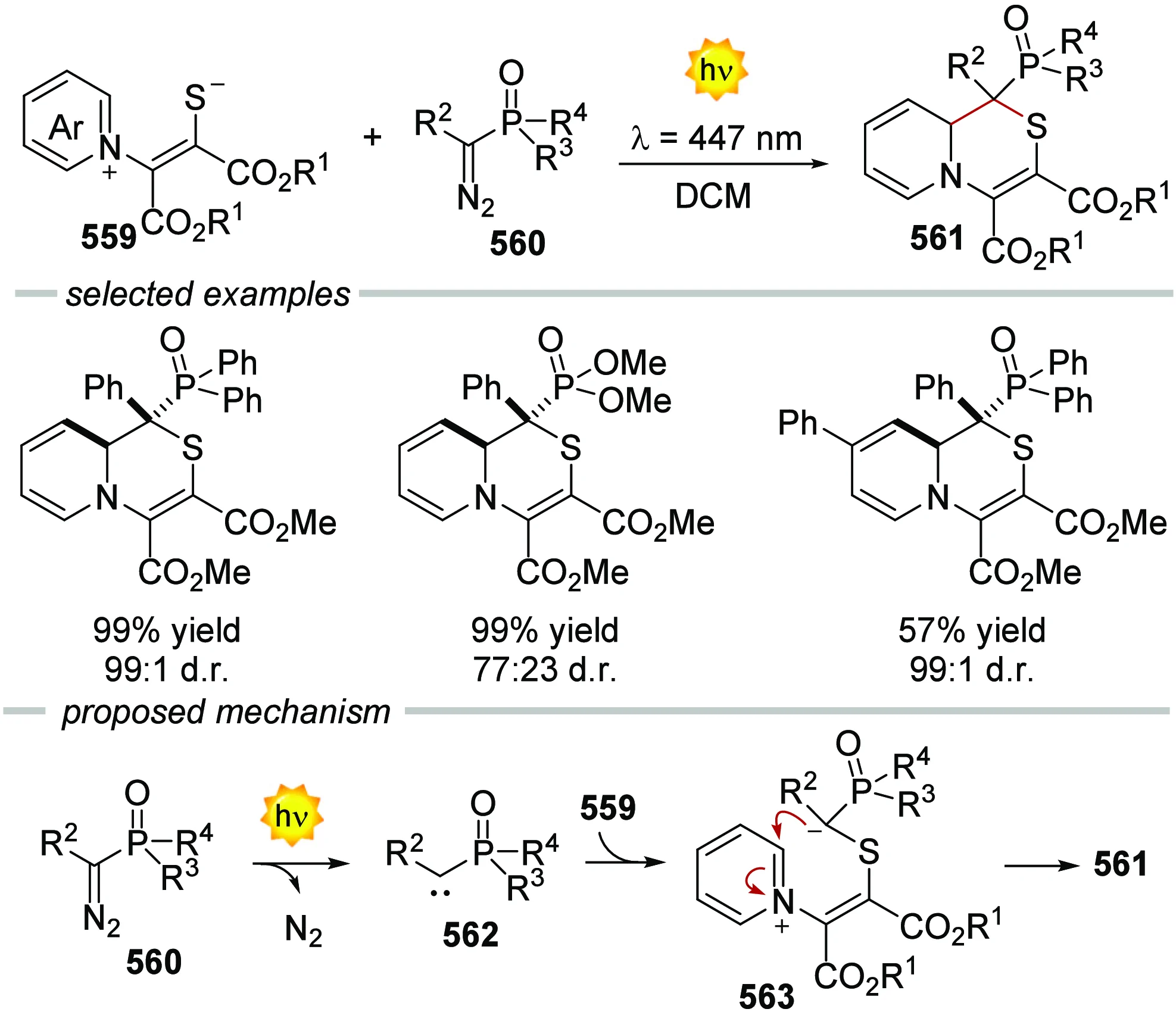
Reaction of Pyridinium Thiolate and Phosphoryl Diazo Compound through Visible-Light Driven Carbene Generation

### 6.2 Photocatalytic Methods

In 2016, Yoon and co-workers developed a visible-light-mediated aziridination of alkenes **52** based on the triplet sensitization of azidoformates **564** using an iridium photocatalyst (Scheme 114a).^451^ Rather than directly exciting the azide, the reaction proceeds through EnT from the photoexcited Ir catalyst (**Ir-9**), enabling the selective generation of triplet nitrenes under mild conditions. Conventional UV irradiation of azide species, which generates both singlet and triplet nitrenes, leads to mixtures of aziridination and allylic C–H amination products.^452,453^ The implementation of a photocatalytic protocol that selectively affords the triplet nitrene provides excellent chemoselectivity for alkene aziridination. The proposed mechanism involves the EnT process from the photexcited iridium photocatalyst to the azidoformate **564**, followed by nitrogen extrusion to produce a triplet carbamoyl nitrene. Then, a stepwise cycloaddition occurs in the presence of suitable alkenes to provide the desired aziridine. A series of mechanistic experiments, such as the loss of stereospecificity starting from *E*- or *Z*-alkenes as well as the stereochemical scrambling of a cyclopropyl radical clock derivative, support the proposed stepwise reaction pathway. More Recently, the same group developed a similar method to obtain reactive triplet nitrene from a simpler carbamate species **566** in combination with an organic photosensitizer (Scheme 114b).^454^

**Scheme 114:**
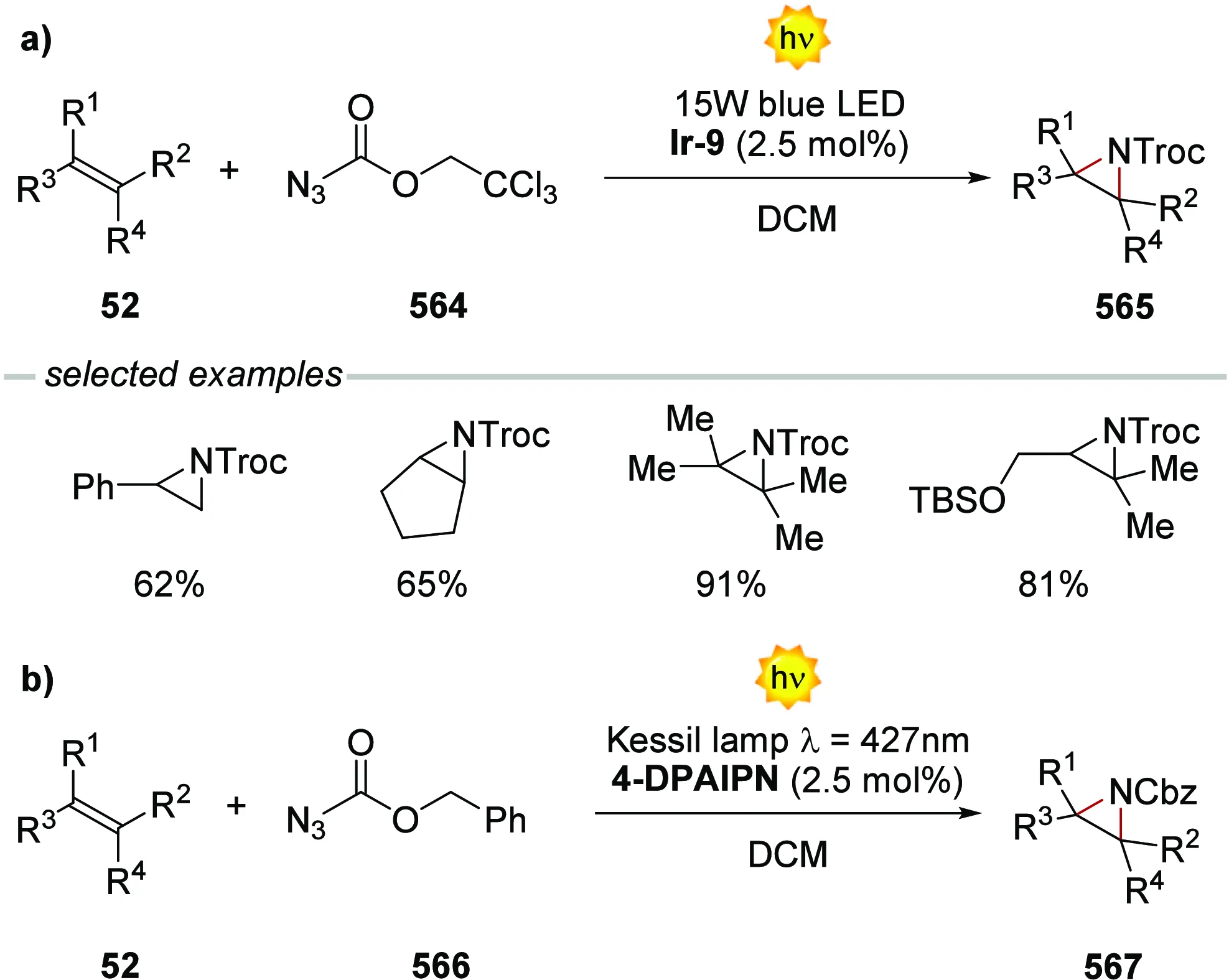
Photocatalytic Aziridination via EnT-Mediated Triplet Nitrene Formation

In 2024, the group of Beier reported a visible-light-mediated strategy for the generation of triplet trifluoromethyl nitrene from thermally stable CF_3_N_3_ **568** using *fac*-Ir­(ppy)_3_ **Ir-10** as a triplet sensitizer (Scheme 115).^455^ Unlike conventional nitrene generation by UV photolysis, the reaction proceeds under mild conditions through EnT, providing selective access to a highly reactive nitrene intermediate. The methodology enables the aziridination of mono-, di-, tri-, and tetrasubstituted alkenes **52**, providing access to the new molecular building block *N*-trifluoromethyl aziridine with broad substrate scope and good stereocontrol. Mechanistic studies strongly support an energy-transfer activation mode rather than a photoredox mechanism. Screening of photocatalysts revealed that catalytic activity correlates with triplet energy rather than excited-state redox potentials. Time-correlated single-photon counting, transient absorption spectroscopy, and EPR experiments provided compelling evidence for triplet energy transfer and the formation of a triplet nitrene intermediate. The proposed reaction pathway involves the excitation of CF_3_N_3_ to its triplet state **568-T**_**1**_ that rapidly collapses, by nearly barrierless N_2_ extrusion, to triplet CF_3_N **570-T**_**1**_. This nitrene adds stepwise to the alkene to form a triplet biradical (**571**, **571’**), which undergoes intersystem crossing and ring closure to afford the aziridine. Calculated biradical energies explain the observed regioselectivity, while alkene isomerization and facile C–C bond rotation account for the predominance of the *anti* diastereomer.

**Scheme 115:**
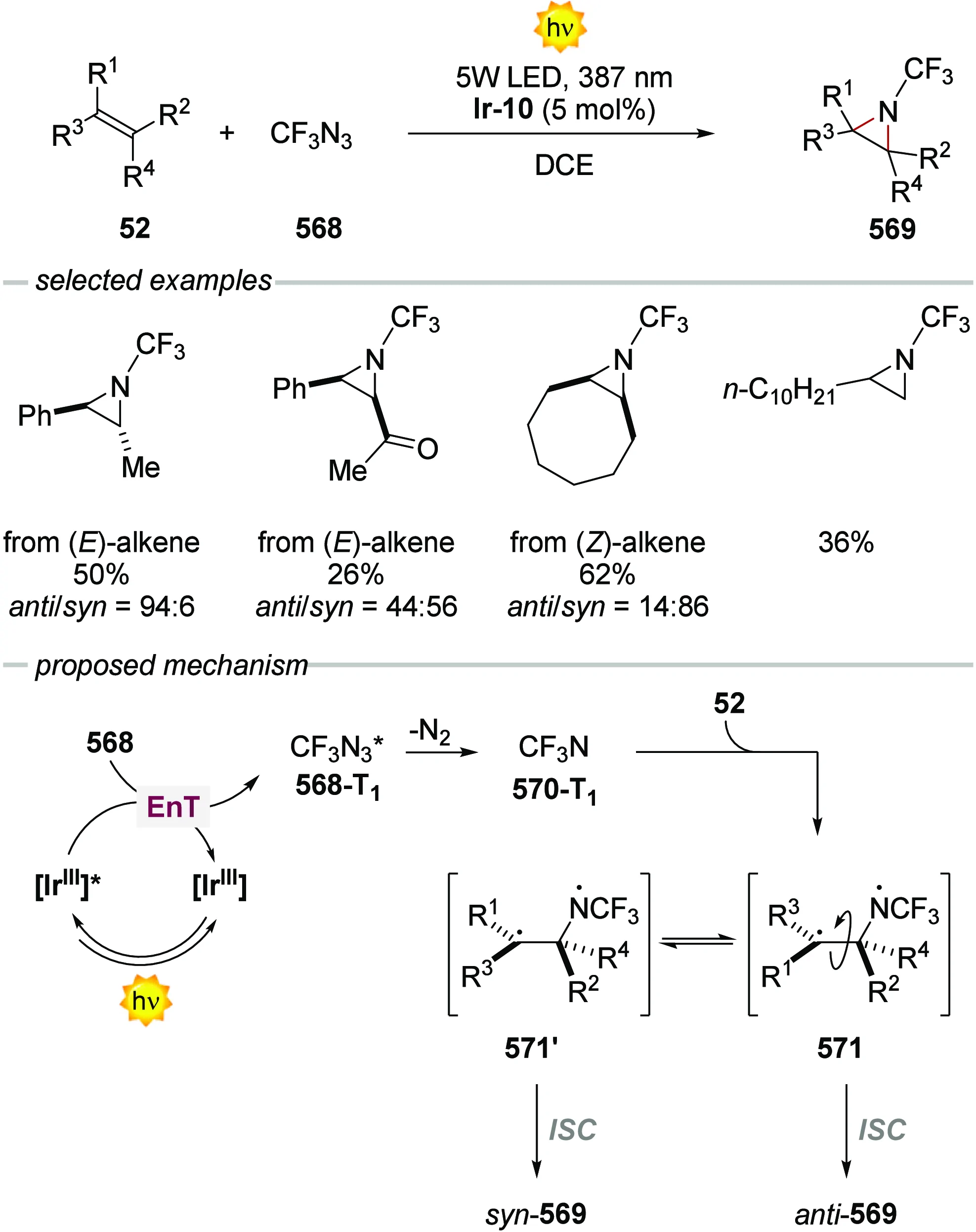
Visible-Light Mediated Aziridination Using Azidotrifluoromethane via EnT Process

In 2021, the group of Koenigs reported the use of iminoiodinanes **572** as bench-stable reagents that can be successfully employed in photocatalytic aziridination reactions of α-trifluoromethyl styrenes **573**, affording valuable trifluoromethylated aziridines **574** (Scheme 116).^456^ The formation of **574** was proposed to proceed *via* oxidative quenching of the ruthenium photocatalyst (**Ru**-**1**) by the iminoiodinane **572**, generating the nitrene radical anion **575**. Subsequent radical addition to **573** furnishes intermediate **576**, which undergoes SET oxidation by the Ru^3+^ photocatalyst to yield the zwitterionic intermediate **577**. According to DFT calculations, this intermediate then cyclizes in a barrierless process to deliver aziridine product **574**. In a subsequent combined theoretical and experimental study, the same group further expanded the scope of this transformation to include aziridination reactions of nontrifluoromethylated α-methyl styrenes.^457^

**Scheme 116:**
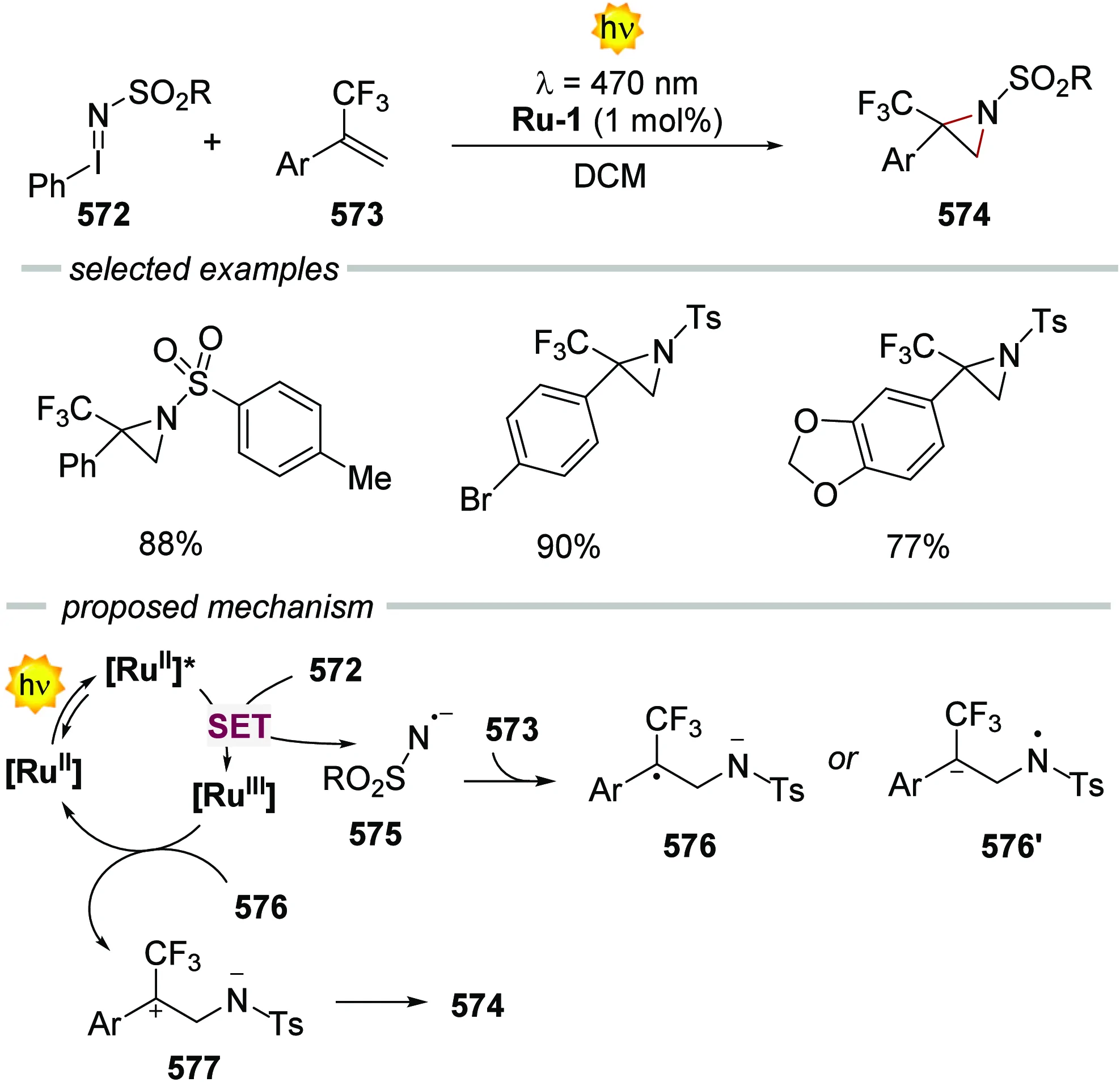
Photocatalytic Synthesis of Aziridines from Styrene Derivatives and Iminoiodinanes

Bouwman, Codée and co-workers developed an intermolecular aziridination of alkenes, including unactivated substrates, through organic dye-sensitized nitrene generation.^458^ In this protocol, benzenesulfonyl azides **578** are activated by visible-light energy transfer from a cyanoarene photosensitizer, generating triplet sulfonyl nitrenes that add to alkenes **52** to furnish *N*-sulfonyl aziridines **579** (Scheme 117). A key feature of the study is the use of the alkene as the limiting reagent, which is particularly advantageous for complex or valuable alkene substrates. By tuning the electronic properties of the sulfonyl azide and matching them with the triplet energy of the organic photosensitizer, the authors minimized photosensitizer degradation and enabled aziridination of simple aliphatic alkenes, styrenes, and a series of more complex olefins. Mechanistic investigations, including Stern–Volmer quenching, electrochemical studies, α-methylstyrene trapping, and kinetic monitoring, supported a photosensitized pathway in which triplet sulfonyl nitrenes, rather than nitrene radical anions, are the product-forming intermediates. Consistently, *cis*- and *trans*-4-octene afforded the same diastereomeric mixture of aziridines, supporting a stepwise triplet nitrene insertion pathway rather than a stereospecific concerted process.

**Scheme 117:**
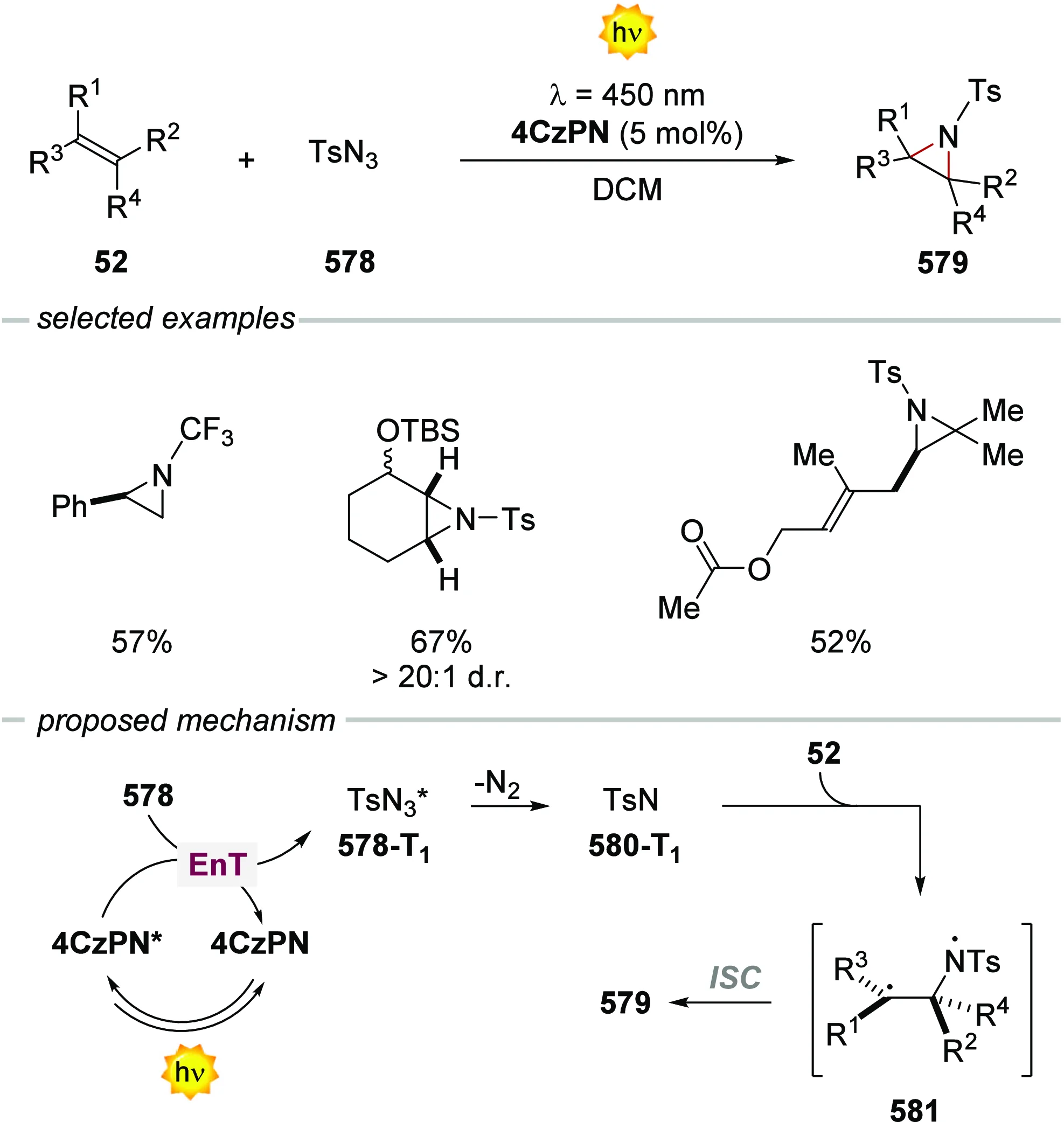
Organic Dye-Sensitized Generation of Triplet Sulfonyl Nitrenes for Intermolecular Aziridination of Unactivated Alkenes

## 7 Conclusions and Outlook

A distinctive feature of heterophotocycloaddition chemistry is its close relations to the construction of biorelevant cores by means of photocatalytic methods.^459,460^ In this Review, we have described the evolution of the field from the beginning of the new millennium to present, highlighting the tremendous ability of photochemical strategies to the construction of heterocyclic targets. *The overall picture that emerges from this work is that the evolution of the HPC chemistry closely mirrors that of modern photochemistry and photocatalysis*. Although direct photoexcitation laid the foundations of the field and continued to provide valuable synthetic transformations, technological advances in selective light-source irradiation (LEDs), together with the advent of visible-light photocatalysis, marked a turning point, by unlocking previously inaccessible hetero-cycloaddition manifolds. Moreover, rather than replacing classical direct photoexcitation strategies,^461,462^ photocatalysis has complemented and revitalized the field, thereby leading to the extraordinary momentum that currently characterizes HPC chemistry.

In this work, we have described how hetero-[2 + 2] photocycloadditions have remained the historical cornerstone of HPC chemistry, while simultaneously becoming one of its principal driving forces for innovation. In particular, classical Paternò–Büchi and aza-Paternò–Büchi reactions have evolved from substrate-driven photochemical processes into more sophisticated photocatalytic systems. Despite these remarkable advances, several challenges remain. In particular, the range of competent imine substrates remains comparatively narrow, and many intermolecular transformations still require one reaction partner to be employed in excess. Comparatively, thia-Paternò–Büchi reactions remain even more underdeveloped. New methods enabling the mild generation and excitation of unstable thiocarbonyls are sought after to increase the efficiency and generality of this class of transformation. Lastly, recent reports on [2 + 2] photocycloaddition of sulfinylamines suggest that new developments in the field will spur from identification of new classes of heteroatom-containing double bonds able to undergo this type of reactivity.

Despite remarkable progress, the examples of enantioselective transformations remain narrow. [2π+2σ]-HPCs have rapidly emerged as a distinct class of cycloadditions, providing access to highly strained bicyclic heterocyclic scaffolds that have emerged as medicinally relevant arene bioisosteres. *Given the infancy of this field, we expect that this class of HPCs will continue to flourish, driven by the growing demand for saturated, three-dimensional bioisosteric scaffolds in drug discovery*.^261,463^ We have shown how (3 + 2)-HPCs encompass a broad mechanistic landscape, involving various photochemical/photocatalytic activation strategies. These transformations share a common design principle based on the photochemical generation of transient 1,3-dipoles, which subsequently react with suitable dipolarophiles in polar or radical (3 + 2) cycloadditions. This modular strategy has enabled the development of a wide range of reactions, providing access to diverse nitrogen- and oxygen-containing five-membered heterocycles. Also in this case the examples of stereoselective processes are rare. On the other hand, [4 + 2]-HPCs enable access to oxygen- and nitrogen-containing six-membered heterocycles typically through the photochemical generation of reactive (hetero)­dienes. Despite their early origins, the current scope of these transformations remains comparatively narrow, thereby highlighting the need for new activation strategies and more general catalytic methods to further unlock their synthetic potential. Finally, the cheletropic (2 + 1) and (5 + 1) HPCs discussed in the final chapter underscore that the use of light can enable the incorporation of a single heteroatom into cyclic frameworks. These transformations represent a promising and still underdeveloped research topic for future developments in HPC chemistry.

### 7.1 Looking Ahead

The future development of HPC chemistry will require addressing challenges that are specific to each cycloaddition class. For [2 + 2]-HPCs, a main goal will engage the development of reactions involving nonactivated carbonyl, imine, and alkene partners under near-stoichiometric conditions. In particular, expanding the substrate scope of aza- and thia-Paternò–Büchi reactions, while achieving predictable chemo-, regio-, and stereoselectivity in structurally more complex substrates, would increase their synthetic usefulness. In the emerging area of [2π+2σ]-HPCs, future developments should extend beyond the currently dominant bicyclobutane-based substrates toward a wider range of strained σ-components. For (3 + 2)-HPCs, advances will be due to identification of novel precursors able of generating 1,3-dipoles under visible-light irradiation. Such developments would be especially valuable for photoclick and bioconjugation chemistry. Finally, as briefly discussed above, [4 + 2]- and cheletropic HPCs remain comparatively underdeveloped. Broadening the array of photogenerated heterodienes and monocentric addends, while replacing substrate-excitation with catalytic activation strategies, could unlock the access to unprecedented six-membered heterocycles and single-atom-transfer processes.

Across all these reaction classes, one of the most significant challenges facing the field remains the development of broadly applicable enantioselective heterophotocycloadditions. Although remarkable advances have been achieved in recent years, asymmetric examples are still scarce and largely confined to specific substrate classes and activation modes.^41–43^ The development of general catalytic asymmetric methodologies remains particularly challenging because stereochemical control must be exerted without altering the delicate excited-state processes responsible for reactivity. Traditional strategies have relied on chiral Lewis acids or organocatalysts possessing suitable photophysical properties to control the reaction outcome.^43,464^ While these approaches have provided elegant proof-of-concept studies, their generality remains limited, likely reflecting the exceptionally diverse mechanistic landscape of HPCs, which encompasses direct excitation, energy transfer (EnT), photoredox activation, and increasingly complex combinations thereof. A particularly promising yet still underexplored direction is the development of dual chiral photocatalytic systems.^465,466^ Although only a handful of examples have been reported to date, these systems have already demonstrated their potential to simultaneously recognize, activate, and stereochemically control the reacting partners through complementary activation modes, including EnT, SET, HAT, and related excited-state processes. *More broadly, we believe that an in-depth understanding of the reaction mechanisms governing HPCs, together with the development of new catalytic systems, will be key to unlocking new reactivity, broadening the scope of these transformations, and enabling the development of general stereoselective methodologies. Such integrated catalyst design may ultimately provide the level of substrate generality and stereochemical control required to transform asymmetric heterophotocycloadditions into broadly applicable synthetic methods, while increasingly contributing to medicinal chemistry and drug discovery through the rapid assembly of biorelevant and bioisosteric scaffolds*.
